# Epidemiological Trends in Alopecia Areata at the Global, Regional, and National Levels

**DOI:** 10.3389/fimmu.2022.874677

**Published:** 2022-07-14

**Authors:** Haifeng Wang, Lifang Pan, Yanfeng Wu

**Affiliations:** ^1^ Department of Hematology and Oncology, Beilun Branch of the First Affiliated Hospital of Medical College of Zhejiang University, Ningbo, China; ^2^ Department of Global Health, Ningbo Institute of Life and Health Industry, University of Chinese Academy of Sciences, Ningbo, China; ^3^ Department of Anesthesiology, Ningbo Hospital of Traditional Chinese Medicine, Zhejiang University of Traditional Chinese Medicine, Ningbo, China

**Keywords:** alopecia areata, incidence, disability-adjusted life-years (DALYs), Global Burden of Disease (GBD), trend

## Abstract

**Background:**

No comprehensive studies have been published on the global burden of alopecia areata since 2010.

**Objective:**

We aimed to measure the global, regional, and national incidence of alopecia areata and disability-adjusted life-years (DALYs) by age, sex, and socio-demographic index (SDI) value from 1990 to 2019.

**Methods:**

Data were extracted from the Global Burden of Disease Study 2019. Estimated annual percentage changes (EAPCs) were calculated to quantify temporal trends in the age-standardized rates of alopecia areata incidence and DALYs. The correlations between EAPCs in the age-standardized rates and SDI values were also analyzed.

**Results:**

From 1990 to 2019, the alopecia areata incidence number and the associated number of DALYs increased globally by 49.14%, and 49.51%, respectively. The global age-standardized incidence rate decreased (EAPC, −0.13; 95% confidence interval [CI], −0.13 to −0.12) and the age-standardized DALY rate showed a downward trend (EAPC, −0.12; 95% CI, −0.13 to −0.11). The largest increases in the age-standardized incidence rate and age-standardized DALY rate were observed in Low SDI quintile and Western Sub-Saharan Africa regions. The regions with the greatest changes in the incidence of alopecia areata were Central Sub-Saharan Africa and Western Sub-Saharan Africa. The three countries with the largest increases in alopecia areata incidence from 1990 to 2019 were Kuwait (EAPC, 0.15), South Sudan (EAPC, 0.12), and Nigeria (EAPC, 0.11). The age-standardized incidence rate was higher in females than in males.

**Conclusion:**

Globally, both the age-standardized incidence rate and age-standardized DALY rate of alopecia areata showed decreasing trends. Future preventive strategies should focus on low-income countries, Central Sub-Saharan Africa, Western Sub-Saharan Africa, Kuwait, South Sudan, Nigeria.

## Introduction

Alopecia areata is a common chronic tissue-specific autoimmune disease that causes patchy hair loss. It affects 2% of the general population (1). In some patients, it may be persistent, especially when hair loss is widespread, and it is an important cause of mental disorders, such as depression, anxiety, and psychosocial distress (2).

Recent studies have only presented the burden of alopecia areata based on regional and/or national data or data from the Global Burden of Disease (GBD) 2010 study (3), and have not provided comprehensive information for all countries and regions (4). Therefore, no comprehensive studies have been published on the global burden of alopecia areata since 2010.

In this study, to provide comprehensive and comparable information on the burden of alopecia areata, we analyzed the global, regional, and national incidence and disability-adjusted life-years (DALYs) data from the GBD 2019 study in terms of counts and age-standardized rates by sex, age, and socio-demographic index (SDI) value.

## Materials and Methods

### Data Source

Annual data on the incidence of alopecia areata and the associated number of DALYs were collected from the GBD 2019 study (http://ghdx.healthdata.org/gbd-results-tool). The data were from 204 countries and territories stratified by age and sex from 1990 to 2019 (5). The 204 countries and territories were classified into five regions, based on low, low-middle, middle, high-middle, and high SDI values, and into 21 geographical areas. The trends in alopecia areata estimates were also assessed according to the following age stratification: 15–19, 20–24, 25–29, 30–34, 35–39, 40–44, 45–49, 50–54, 55–59, 60–64, 65–69, 70–74, 75–79, ≥80, 80–84, 85–89, 90–94, and ≥95 years. We followed the recommendations provided in the Guidelines for Accurate and Transparent Health Estimates Reporting (6).

### Estimation Framework

The incidence of alopecia areata was estimated using DisMod-MR, a Bayesian meta-regression disease modeling tool (5). The number of years lived with disability was calculated as the product of the disability weight and the prevalence of alopecia areata. The number of DALYs due to alopecia areata were calculated as the sum of the number of years lived with disability and the years of life lost due to premature death. Final estimates were computed using the mean estimates across 1,000 draws, and 95% uncertainty intervals (UIs) were specified on the basis of the 25^th^ and 975^th^ values across all 1,000 draws.

### Statistical Analysis

Estimated annual percentage changes (EAPCs) were calculated to quantify trends in the incidence of alopecia areata and the number of DALYs. The natural logarithm of the regression line fitted to the age-standardized rate was y = a + bx + c, where x is the calendar year (7). The EAPC was calculated as 100 × (exp(b) − 1), and its 95% confidence interval (CI) was obtained using a linear regression model. If the EAPC and the lower bound of its 95% CI were both > 0, the age-standardized rate was considered to exhibit an increasing trend. In contrast, when both the EAPC and the upper bound of its 95% CI were < 0, the age-standardized rate was considered to exhibit a decreasing trend. Otherwise, the age-standardized rate was considered to be stable. We further evaluated the associations between EAPCs in the age-standardized rates and SDI values using Pearson’s correlation analysis.

## Results

### Global Alopecia Areata Data

Globally, the incidence number of alopecia areata increased from 21742836.45 (95% UI,20996994.59 to 22478104.50) in 1990 to 32426829.18 (95% UI, 31370861.58 to 33473493.05) in 2019, whereas the related number of DALYs increased from 401682.16 (95% UI, 251624.90 to 595419.64) in 1990 to 600570.37 (95% UI, 378239.38 to 891060.98) in 2019. Based on these values, the incidence of alopecia areata and the number of DALYs increased by 49.14%, and 49.51%, respectively, from 1990 to 2019.

**Figure 1:**
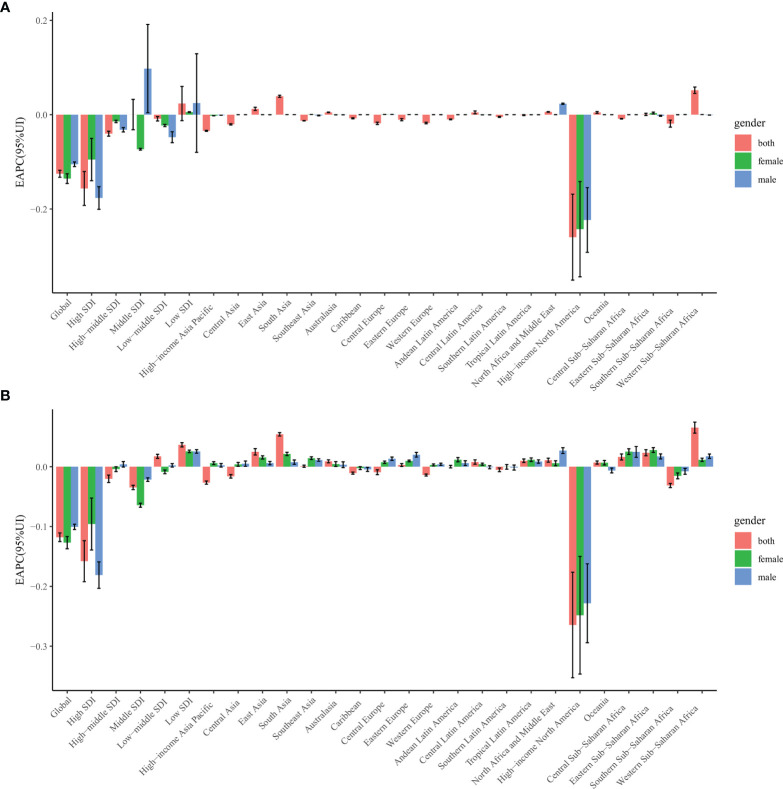
The estimated annual percentage change of alopecia areata age-standardized rates from 1990 to 2019, by sex and region. **(A)** The estimated annual percentage change of ASIR. **(B)** The estimated annual percentage change of age-standardized DALY rate. ASIR = age standardized incidence rate. DALY = disability adjusted life-year.

From 1990 to 2019, the age-standardized incidence rate (EAPC, −0.13; 95% CI, −0.13 to −0.12) of alopecia areata and the age-standardized DALY rate (EAPC, −0.12; 95% CI, −0.13 to −0.11) showed a downward trend globally (**Tables 1**, **2**, **Figures 1A, B**; **Supplementary Figure 1**). The age-standardized incidence rates for both females (EAPC, −0.14; 95% CI, −0.15 to −0.13) and males (EAPC, −0.11; 95% CI, −0.11 to −0.10) decreased during this period. The age-standardized DALY rates (EAPC, −0.13; 95% CI, −0.14 to −0.12) for both females and males (EAPC, −0.10; 95% CI, −0.10 to −0.10) also showed decreasing trends (**Tables 1**, **2**, **Figures 1A, B**; **Supplementary Figure 1**).

**Table 1:** The age-standardized incidence rate (ASIR) of alopecia areata in 1990 and 2019 and its temporal trends.

Characteristics	1990		2019		1990-2019	
	ASIR (per 100000)		ASIR (per 100000)		Percent change (%)	EAPC
	No. (95% UI)	Male/female ratio	No. (95% UI)	Male/female ratio		No. (95% CI)
**Global**	418.64 (405.28,432.10)	0.523217458	405.70 (392.72,418.82)	0.525081429	-0.03 (-0.03,-0.03)	-0.13 (-0.13,-0.12)
**Sex**	–	–	–	–	–	–
Male	287.26 (277.02,296.78)	–	279.18 (269.58,288.25)	–	-0.03 (-0.03,-0.03)	-0.11 (-0.11,-0.10)
Female	549.03 (531.31,567.35)	–	531.68 (514.17,548.87)	–	-0.03 (-0.03,-0.03)	-0.14 (-0.15,-0.13)
**Sociodemographic index**	–	–	–	–	–	–
Low SDI	354.25 (342.24,365.82)	0.537170835	343.59 (331.75,354.88)	0.483886803	-0.03 (-0.03,-0.03)	0.02 (-0.01,0.06)
Low-middle SDI	362.87 (350.86,375.18)	0.534427671	360.20 (348.33,372.44)	0.523401744	-0.01 (-0.01,-0.01)	-0.01 (-0.01,0.00)
Middle SDI	407.27 (393.65,421.23)	0.543357228	392.93 (380.00,406.41)	0.514560898	-0.04 (-0.04,-0.03)	0.00 (-0.03,0.03)
High-middle SDI	414.22 (400.17,428.16)	0.535859382	408.62 (394.86,422.37)	0.531364676	-0.01 (-0.02,-0.01)	-0.04 (-0.05,-0.04)
High SDI	530.84 (514.70,547.58)	0.528233225	518.91 (503.45,534.61)	0.50814547	-0.02 (-0.03,-0.02)	-0.16 (-0.19,-0.12)
**Region**	–	–	–	–	–	–
Andean Latin America	383.86 (369.74,397.25)	0.521621462	382.81 (368.66,396.19)	0.521621462	0.00 (0.00,0.00)	-0.01 (-0.01,-0.01)
Australasia	504.77 (486.42,522.50)	0.579198738	505.63 (487.01,523.40)	0.5792039	0.00 (0.00,0.00)	0.00 (0.00,0.01)
Caribbean	384.42 (370.28,397.86)	0.521621462	383.78 (369.64,397.19)	0.521621462	0.00 (0.00,0.00)	-0.01 (-0.01,-0.01)
Central Asia	386.39 (372.21,399.77)	0.521621462	384.18 (370.00,397.54)	0.521621462	-0.01 (-0.01,-0.01)	-0.02 (-0.02,-0.02)
Central Europe	384.04 (370.83,396.87)	0.521617167	382.28 (369.19,395.08)	0.521619608	0.00 (-0.01,0.00)	-0.02 (-0.02,-0.02)
Central Latin America	385.99 (372.26,398.80)	0.521630068	386.69 (373.13,399.48)	0.521644764	0.00 (0.00,0.00)	0.00 (0.00,0.01)
Central sub-Saharan Africa	364.72 (350.02,378.82)	0.535031465	363.93 (349.29,377.97)	0.535031465	0.00 (0.00,0.00)	-0.01 (-0.01,-0.01)
East Asia	420.25 (404.58,435.74)	0.501426801	420.92 (405.25,436.46)	0.501397506	0.00 (0.00,0.00)	0.01 (0.01,0.02)
Eastern Europe	389.49 (375.28,402.64)	0.521655122	387.84 (373.63,400.97)	0.52165053	0.00 (0.00,0.00)	-0.01 (-0.01,-0.01)
Eastern sub-Saharan Africa	363.78 (351.09,375.66)	0.548729167	363.94 (351.37,376.10)	0.547242338	0.00 (0.00,0.00)	0.00 (0.00,0.00)
High-income Asia Pacific	507.83 (490.73,524.97)	0.581285075	502.46 (485.60,519.49)	0.581439595	-0.01 (-0.01,-0.01)	-0.03 (-0.04,-0.03)
High-income North America	639.69 (620.86,658.03)	0.408138331	624.02 (606.60,641.27)	0.392111643	-0.02 (-0.03,-0.02)	-0.26 (-0.35,-0.17)
North Africa and middle East	339.55 (327.03,352.11)	0.527718588	338.48 (326.25,350.73)	0.531040905	0.00 (-0.01,0.00)	0.01 (0.01,0.01)
Oceania	439.98 (423.53,456.50)	0.554865943	441.02 (424.74,457.58)	0.554865943	0.00 (0.00,0.00)	0.00 (0.00,0.01)
South Asia	336.01 (323.93,348.16)	0.531350627	338.74 (326.52,350.98)	0.531359879	0.01 (0.01,0.01)	0.04 (0.04,0.04)
Southeast Asia	477.96 (461.87,493.28)	0.662434444	476.15 (460.12,491.59)	0.661543783	0.00 (0.00,0.00)	-0.01 (-0.01,-0.01)
Southern Latin America	508.42 (489.28,526.64)	0.579293957	507.87 (488.73,526.08)	0.579293957	0.00 (0.00,0.00)	0.00 (-0.01,0.00)
Southern sub-Saharan Africa	368.16 (355.28,380.97)	0.5352288	366.63 (353.95,379.33)	0.535280098	0.00 (0.00,0.00)	-0.02 (-0.03,-0.01)
Tropical Latin America	386.51 (372.20,400.07)	0.521654701	386.61 (372.32,400.09)	0.521653697	0.00 (0.00,0.00)	0.00 (0.00,0.00)
Western Europe	480.14 (463.65,496.74)	0.713554374	477.71 (461.22,494.16)	0.713507145	-0.01 (-0.01,0.00)	-0.02 (-0.02,-0.02)
Western sub-Saharan Africa	362.06 (349.13,374.76)	0.535260614	367.17 (354.01,380.14)	0.535107475	0.01 (0.01,0.02)	0.05 (0.05,0.06)

ASIR, age-standardized incidence rate; EAPC, estimated annual percentage change; UI, uncertainty interval.

**Table 2:** The age-standardized DALY rate of alopecia areata in 1990 and 2019 and its temporal trends.

Characteristics	1990		2019		1990-2019	
	age-standardized DALY rate (per 100000)		age-standardized DALY rate (per 100000)		Percent change(%)	EAPC
	No. (95% UI)	Male/female ratio	No. (95% UI)	Male/female ratio		No. (95% CI)
**Global**	7.74(4.86,11.47)	0.530912248	7.51(4.73,11.14)	0.532222484	-0.03(-0.04,-0.02)	-0.12(-0.13,-0.11)
**Sex**						
Male	5.36(3.36,7.97)	–	5.22(3.27,7.79)	–	-0.03(-0.04,-0.01)	-0.10(-0.10,-0.10)
Female	10.10(6.35,15.01)	–	9.80(6.18,14.51)	–	-0.03(-0.04,-0.02)	-0.13(-0.14,-0.12)
**Sociodemographic index**					
Low SDI	6.49(4.10,9.64)	0.543006758	6.55(4.15,9.74)	0.543072055	0.01(-0.01,0.03)	0.04(0.03,0.04)
Low-middle SDI	6.67(4.21,9.86)	0.540662598	6.70(4.20,9.96)	0.542335326	0.00(-0.02,0.03)	0.02(0.01,0.02)
Middle SDI	7.55(4.75,11.18)	0.549051019	7.47(4.71,11.04)	0.555377453	-0.01(-0.02,0.00)	-0.03(-0.04,-0.03)
High-middle SDI	7.68(4.83,11.41)	0.541810358	7.62(4.79,11.28)	0.543054668	-0.01(-0.02,0.01)	-0.02(-0.03,-0.01)
High SDI	9.81(6.16,14.62)	0.541213556	9.57(6.05,14.26)	0.520526917	-0.02(-0.04,-0.01)	-0.16(-0.19,-0.12)
**Region**					
Andean Latin America	7.11(4.53,10.71)	0.527259636	7.10(4.51,10.52)	0.526638444	0.00(-0.07,0.07)	0.00(0.00,0.00)
Australasia	9.31(5.83,13.82)	0.591846735	9.33(5.89,13.89)	0.590115226	0.00(-0.07,0.08)	0.01(0.01,0.01)
Caribbean	7.12(4.53,10.59)	0.525932691	7.10(4.47,10.50)	0.52628699	0.00(-0.05,0.04)	-0.01(-0.01,-0.01)
Central Asia	7.16(4.55,10.69)	0.525563456	7.13(4.47,10.68)	0.526978308	0.00(-0.05,0.04)	-0.02(-0.02,-0.01)
Central Europe	7.11(4.51,10.56)	0.522992222	7.10(4.47,10.55)	0.523280676	0.00(-0.03,0.02)	-0.01(-0.01,-0.01)
Central Latin America	7.14(4.53,10.61)	0.52618357	7.17(4.53,10.63)	0.525998862	0.00(-0.02,0.03)	0.01(0.00,0.01)
Central sub-Saharan Africa	6.67(4.23,10.06)	0.542227377	6.70(4.29,9.85)	0.540751603	0.00(-0.06,0.08)	0.02(0.01,0.02)
East Asia	7.82(4.91,11.66)	0.506744218	7.86(4.92,11.72)	0.504972971	0.01(-0.02,0.03)	0.02(0.02,0.03)
Eastern Europe	7.19(4.56,10.74)	0.523992203	7.19(4.53,10.66)	0.524676646	0.00(-0.03,0.03)	0.00(0.00,0.01)
Eastern sub-Saharan Africa	6.69(4.23,9.92)	0.553384698	6.72(4.26,10.06)	0.551618668	0.01(-0.02,0.03)	0.02(0.02,0.03)
High-income Asia Pacific	9.43(5.89,14.00)	0.594608927	9.35(5.88,13.90)	0.593931527	-0.01(-0.04,0.02)	-0.03(-0.03,-0.02)
High-income North America	11.76(7.42,17.55)	0.419121336	11.43(7.22,16.95)	0.403572633	-0.03(-0.05,-0.01)	-0.26(-0.35,-0.18)
North Africa and middle East	6.28(3.97,9.29)	0.534316185	6.26(3.97,9.31)	0.537688172	0.00(-0.03,0.03)	0.01(0.01,0.01)
Oceania	8.12(5.15,12.08)	0.560487924	8.14(5.09,12.12)	0.560656739	0.00(-0.06,0.07)	0.01(0.00,0.01)
South Asia	6.15(3.88,9.10)	0.538577928	6.23(3.94,9.22)	0.538785394	0.01(-0.01,0.04)	0.05(0.05,0.06)
Southeast Asia	8.85(5.57,13.20)	0.668210772	8.85(5.51,13.14)	0.666815944	0.00(-0.02,0.02)	0.00(0.00,0.00)
Southern Latin America	9.42(5.94,14.01)	0.591705936	9.41(5.96,14.00)	0.591308647	0.00(-0.06,0.06)	-0.01(-0.01,0.00)
Southern sub-Saharan Africa	6.79(4.28,10.17)	0.541298609	6.73(4.25,10.06)	0.542072019	-0.01(-0.04,0.03)	-0.03(-0.03,-0.03)
Tropical Latin America	7.12(4.47,10.58)	0.52725044	7.14(4.51,10.59)	0.526677162	0.00(-0.02,0.03)	0.01(0.01,0.01)
Western Europe	8.89(5.57,13.21)	0.731775711	8.85(5.55,13.27)	0.731705621	0.00(-0.03,0.02)	-0.01(-0.02,-0.01)
Western sub-Saharan Africa	6.67(4.22,9.93)	0.540360787	6.79(4.30,10.14)	0.540856144	0.02(0.00,0.04)	0.07(0.06,0.07)

DALY, disability adjusted life-years; UI, uncertainty interval.

### Alopecia Areata Incidence

The largest increase in the age-standardized incidence rate of alopecia areata from 1990 to 2019 was observed in countries in the low-SDI quintile (EAPC, 0.02; 95% CI, −0.01 to 0.06; **Table 1**). High-SDI countries had the highest age-standardized incidence rate (530.84 in 1990 and 518.91 in 2019), whereas low-SDI countries had the lowest age-standardized incidence rate (354.25 in 1990 and 343.59 in 2019; **Table 1**). The age-standardized incidence rates in countries in the low-middle, high-middle, and high SDI quintiles decreased with time (**Table 1**).

The regions with the greatest changes in the incidence of alopecia areata from 1990 to 2019 were Central Sub-Saharan Africa (1.47) and Western Sub-Saharan Africa (1.47), whereas the region with the smallest change was Central Europe (−0.04, **Supplementary Table 2**). However, the age-standardized incidence rate of alopecia areata from 1990 to 2019 was higher in females than in males, as demonstrated by male-to-female ratios of 0.52 and 0.53 in 1990 and 2019, respectively (**Table 1**).

The male-to-female ratio of alopecia areata incidence peaked in the 20–24-year age group globally and in high-SDI, high-middle-SDI, middle-SDI, and low-middle-SDI regions, but in the 90–94-year age group in low-SDI regions (**Supplementary Figure 2A**). The incidence of alopecia areata was highest in females aged 30–34 years across all SDI regions (**Supplementary Figure 3**).

Furthermore, the age-standardized alopecia areata incidence rates and their trends varied among different countries. The three countries with the largest increases in the incidence of alopecia areata from 1990 to 2019 were Kuwait (EAPC, 0.15), South Sudan (EAPC, 0.12), and Nigeria (EAPC, 0.11; **Supplementary Table 1**).

The EAPC in the age-standardized incidence rate of alopecia areata was found to be negatively correlated with the age-standardized incidence rate of alopecia areata (*ρ* = −0.146, *P* = 0.037, **Supplementary Figure 4A**) and the SDI value of the region (*ρ* = −0.141, *P* = 0.045, **Supplementary Figure 4B**). We also found that regions with higher SDI values had lower proportions of alopecia areata incidence in young people, whereas regions in which the SDI value increased from 1990 to 2019 had a lower proportion of alopecia areata incidence in older adults (**Supplementary Figures 5A, B**). The annual proportions of alopecia areata incidence in young people and older adults were relatively stable from year to year (**Supplementary Figure 6A**).

### Alopecia Areata-Related DALYs

The largest increase in the age-standardized DALY rate of alopecia areata from 1990 to 2019 was observed in countries in the low-SDI quintile (EAPC, 0.04; 95% CI, 0.03 to 0.04; **Table 2**). The highest age-standardized DALY rates of alopecia areata were observed in high-SDI regions in 1990 (9.81) and in 2019 (9.57). The lowest age-standardized DALY rates remained in countries in the low-SDI quintile during this period (6.49 in 1990 and 6.55 in 2019; **Table 2**).

The three regions with the highest age-standardized DALY rate of alopecia areata in 2019 were High-income North American (11.43), Southern Latin America (9.41), and High-income Asia Pacific (9.35, **Supplementary Table 2**), whereas those with the lowest age-standardized DALY rates were South Asia (6.23), North Africa and the Middle East (6.26), and Central Sub-Saharan Africa (6.70, **Supplementary Table 2**). The largest increase in the age-standardized DALY rate was in Western Sub-Saharan Africa (total: EAPC, 0.07), whereas the largest decrease was in the High-income North America (total: EAPC, −0.26; females: EAPC, −0.25; males: EAPC, −0.23). In females, the EAPC showed the largest increase in Eastern Sub-Saharan Africa (EAPC, 0.03) and Central Sub-Saharan Africa (EAPC, 0.03), whereas in males, the EAPC showed the largest decrease in high-income North American countries (EAPC, −0.23; **Supplementary Table 2**).

From 1990 to 2019, age-standardized DALYs associated with alopecia areata was lower in males than in females, as demonstrated by a male-to-female ratio of 0.53 in 1990 and 2019 (**Table 2**). The male-to-female ratio of the age standardized DALYs peaked in the 25–29-year age group globally, in the 20–24-year age group in high-SDI and high-middle-SDI regions, in the 40–44-year age group in low-middle-SDI regions (**Supplementary Figure 7**). The alopecia areata-associated DALYs was highest in females aged 30–34 years across all SDI regions (**Supplementary Figure 8**).

As shown in **Supplementary Tables 1, 4**, females in the United States of America (16.24) and males in Italy (7.47) had the highest age-standardized DALYs in 2019. The largest decrease in the age-standardized DALY rate was in the United States of America (total: EAPC, −0.29; females: EAPC, −0.27; males: EAPC, −0.25), whereas the largest increase was in Kuwait (total: EAPC, 0.15). In males, the largest increase in the age-standardized DALY rate was in Equatorial Guinea (EAPC, 0.04; **Supplementary Table 1**).

Negative correlations were found between the EAPC in the age-standardized DALY rate of alopecia areata and the age-standardized DALY rate (*ρ* = −0.164, *P* = 0.019, **Supplementary Figure 4C**). In 2019, high-middle-SDI regions had the highest proportion of alopecia areata-related DALYs in young people (15–49 years), whereas regions in which the SDI value increased from 1990 to 2019 had a lower proportion of DALYs in older adults (**Supplementary Figures 5C, D**). The annual proportions of alopecia areata-related DALYs in young people and older adults were relatively stable from year to year (**Supplementary Figure 6B**).

## Discussion

Skin conditions are a significant contributor to the burden of non-pathogenic disease in all countries and regions globally (3). Alopecia areata is associated with a higher incidence of other autoimmune diseases, such as autoimmune thyroid disease (8), pernicious anemia (9), and celiac disease (10). Numerous studies also have showed psychological correlates of alopecia areata, including anxiety (11, 12), depression (11, 12), stress (13), and social and functional deficits (14). We systematically analyzed the incidence and DALY rates of alopecia areata from 1990 to 2019 by sex, age, and SDI classification at the global, regional, and national levels. The global age-standardized incidence rate and age-standardized DALY rate decreased (−0.13% and −0.12%, respectively) during this period, although the decreases were small.

The burden of specific skin diseases has been shown to vary by country and socioeconomic status (4). Similarly, we found that the global burden of alopecia areata showed some regional differences according to SDI values. The SDI value of a region was inversely associated with the EAPC in the age-standardized incidence rate or age-standardized DALY rate in that region. Globally, the male-to-female ratios of the incidence of alopecia areata and the number of associated DALYs among different age groups were more affected in regions with high SDI values.

At the regional level, the age-standardized incidence rate and DALY rate increased significantly in Western Sub-Saharan Africa and South Asia. South Asia is the most densely populated region in the world and the poorest region after Sub-Saharan Africa. The poor living environment in these areas makes the local population prone to inflammation, and alopecia areata often coexists with autoimmune diseases, which may be secondary to infection or inflammation (15). In addition, deficiencies in micronutrients, such as vitamin D, are more widespread in these areas. Deficiencies in zinc and folic acid also affect the occurrence of alopecia areata (16). The increases in the incidence and DALYs in Western Sub-Saharan Africa and South Asia might be due to the increase in population growth, urbanization, increased household income, increased self-examination, and screening programs, improved access to early detection (17). Besides, in Asian societies, hair often represents an important factor in femininity, fertility, and female attractiveness, and thus may lead to higher disease awareness. However, the higher rate in Africa should be interpreted with caution, because the quality and accuracy of GBD data for such regions cannot be guaranteed (18).

Although the burden of alopecia areata in high-income North American countries declined over the 20-year study period, it was still high in high-income North American countries, Southern Latin America, and Australasia. The higher age-standardized incidence rate and DALY rate in 2019 in regions with higher SDI values, such as the Asia Pacific region, may be because people in these regions have a greater number of stressful life events (19). Many reasons may explain the low rate of alopecia areata in certain countries, such as, socioeconomic status, access to early detection, geographic distribution of dermatologists over time, insurance covrage, underdiagnosis (less screening for skin conditions), and disease awareness (12).

Alopecia areata can occur in people of any sex and age (20). However, our previous studies have shown that alopecia areata causes a higher level of stress in women than in men. Moreover, severe alopecia areata, in which the total area of hair loss is greater than 50% of the scalp area, is more common in women (21). Female alopecia areata patients are also more likely to have anxiety-related disorders, such as trait anxiety, social phobia, and social anxiety, and develop mental disorders, which have a greater adverse effect on the quality of life and increase the disease burden (2, 22). The global incidence and DALY rates of alopecia areata were higher in the 20–24- and 30–34-year age groups for males, females, and both sexes combined. This is consistent with previous research showing higher incidence rates of alopecia areata in the second and third decades (23). While patients with alopecia areata are receiving treatment, they also need to pay attention to their psychosocial needs. Planned media interventions and investments in health insurance funds for wigs are also necessary (24).

To the best of our knowledge, this is the first recent study of the global burden of alopecia areata since 2010. Here, we describe recent trends in the global epidemiology of alopecia areata at the national and regional levels according to sex, age, and SDI value. GBD data are updated annually, with improved methods as appropriate, and incorporated many newly acquired data sources (18). The studies used for alopecia areata in GBD 2010 were published between 1980 and 2010 (3). However, a systematic review of the literature was conducted using PubMed to expand the GBD 2019 dataset, with new epidemiological data for alopecia areata published since 2010 for both developed countries and developing countries (18). In GBD 2019, 7,333 national and 24,657 local vital registries, 16,984 published studies, and 1,654 household surveys were used in the GBD 2019 analysis (18), so there are significant advances that can be updated to a good estimate of the burden of alopecia areata (18).

However, this study has some limitations that should be noted. As the GBD study is a population-based study and the incidence of self-reported alopecia areata is low, the global burden of alopecia areata may have been underestimated. This may have been especially true in low-SDI regions with poor health awareness and in patients with less-severe alopecia areata that has not been diagnosed and treated (25). The GBD estimates were calculated based on an algorithm that was strongly dependent on the quality and quantity of data (retrospectively) used in the modeling (18). However, in many regions of the world, including Latin America, sub-Saharan Africa and Asia, data are lacking or extremely lacking (18). As a result, some estimates around the world may show unusual changes over some of the time periods analyzed (18). However, the annual update of GBD will allow each iteration to improve the method and include the latest data sources, especially in data-sparse locations (18). Last, due to the unavailability of data, emotional distress, and financial impact were not considered into the estimation of DALYs.

Although the global age-standardized incidence rate and DALY rate of alopecia areata decreased from 1990 to 2019, the decreases were small, and some low-income regions and countries, such as Central Sub-Saharan Africa, Western Sub-Saharan Africa, Kuwait, South Sudan, Nigeria had increased burden of alopecia areata. Focusing on these regions, especially on younger people in these regions, may be an effective strategy to reduce the global burden of alopecia areata.

## Data Availability Statement

The datasets generated and/or analysed during the current study are available in the Global Health Data Exchange GBD Results Tool repository, http://ghdx.healthdata.org/gbd-results-tool.

## Author Contributions

HW: drafted the manuscript. LP and HW: data analysis. YW: revision of the paper. All authors contributed to the article and approved the submitted version.

## Funding

This study was supported by Zhejiang Medical Association Clinical Research Fund project, Ningbo, East China blood products research project (2021ZYC-A116).

## Conflict of Interest

The authors declare that the research was conducted in the absence of any commercial or financial relationships that could be construed as a potential conflict of interest.

## Publisher’s Note

All claims expressed in this article are solely those of the authors and do not necessarily represent those of their affiliated organizations, or those of the publisher, the editors and the reviewers. Any product that may be evaluated in this article, or claim that may be made by its manufacturer, is not guaranteed or endorsed by the publisher.
