# Supplementary material for: Epidemiological Trends in Alopecia Areata at the Global, Regional, and National Levels
Source: Front Immunol. 2022 Jul 14;13:874677. doi: 10.3389/fimmu.2022.874677 (PMC9331164; doi:10.3389/fimmu.2022.874677)
Supplement: Supplementary file 1 [file DataSheet_1.doc]

**Supplemental Figure 1: The change trends of age-standardized alopecia areata incidence, and DALY rate among different SDI quintiles and gender from 1990 to 2019. (A) ASIR, age standardized incidence rate. (B) age-standardized DALY rate.** Abbreviations: DALY = disability adjusted life-year.


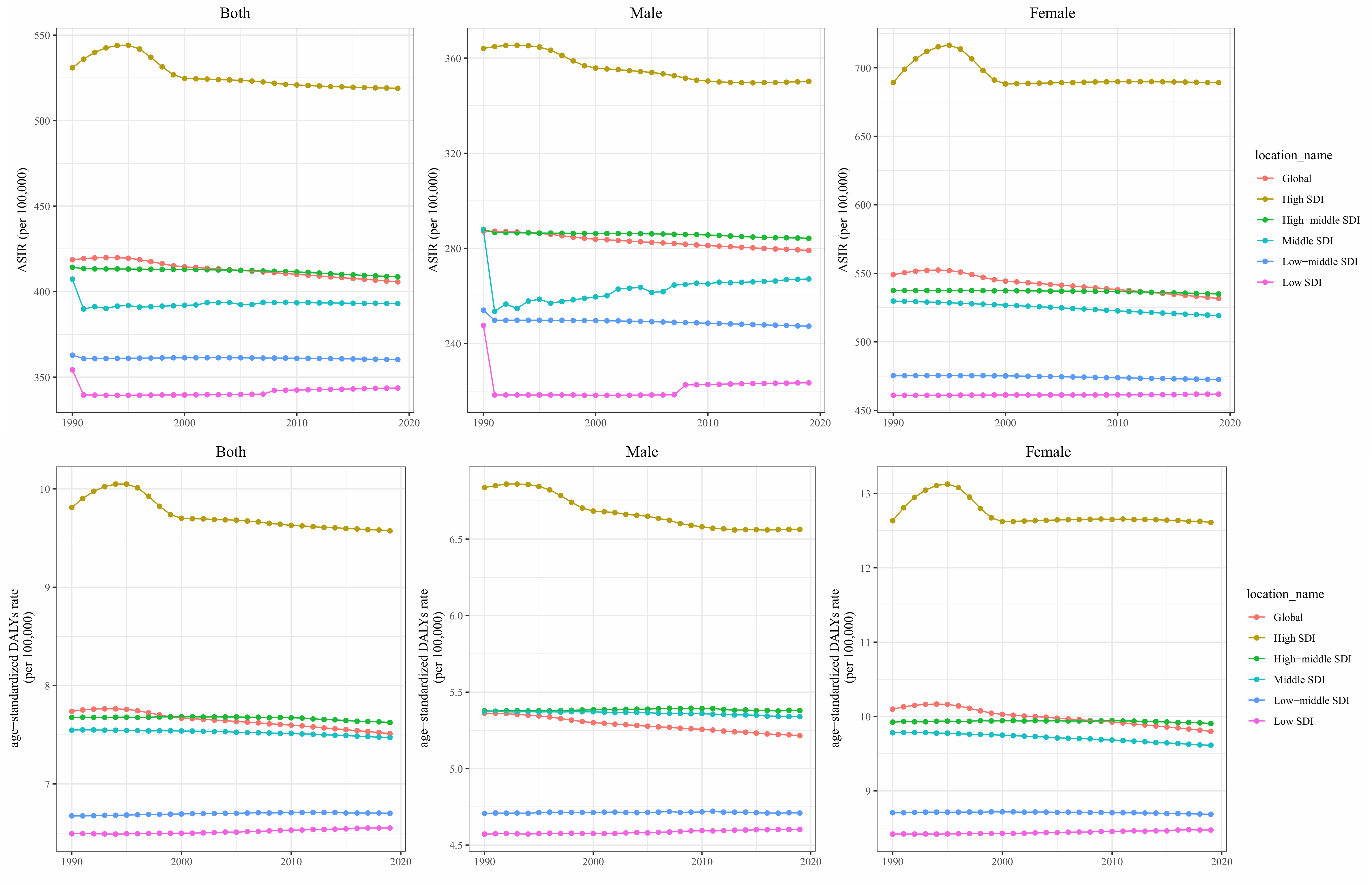


**Supplemental Figure 2: The ratio of male to female incidence among different age groups in 2019. (A) Global. (B) High SDI. (C) High-middle SDI. (D) Middle SDI. (E) Middle-low SDI. (F) Low SDI.** Abbreviations: SDI = socio-demographic index.


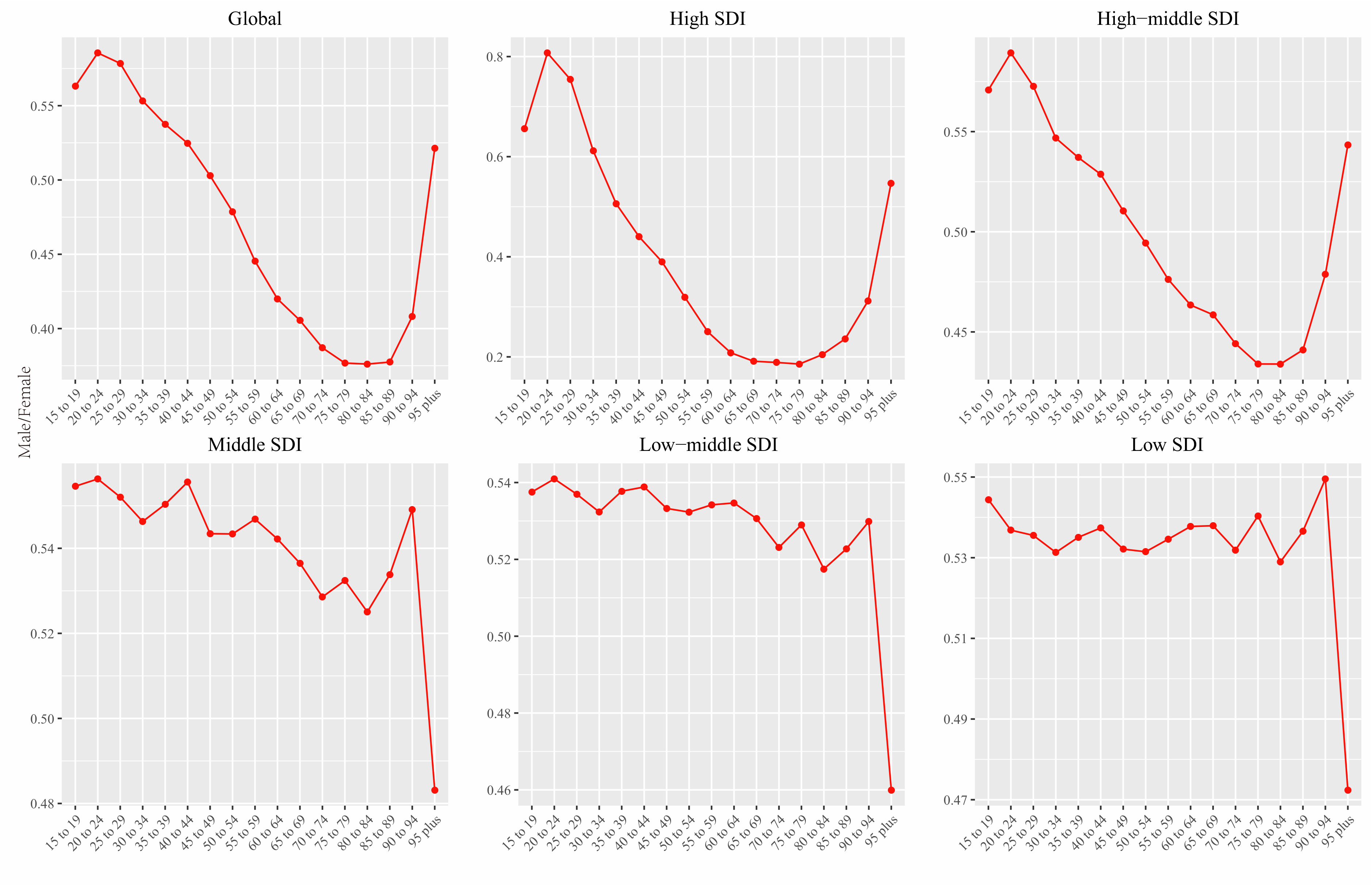


**Supplemental Figure 3. Distribution of different ages in alopecia areata incidence in global (A), high SDI (B), high-middle SDI (C), middle SDI (D), middle-low SDI (E), low SDI (F).** Abbreviations: SDI, socio-demographic index.

**
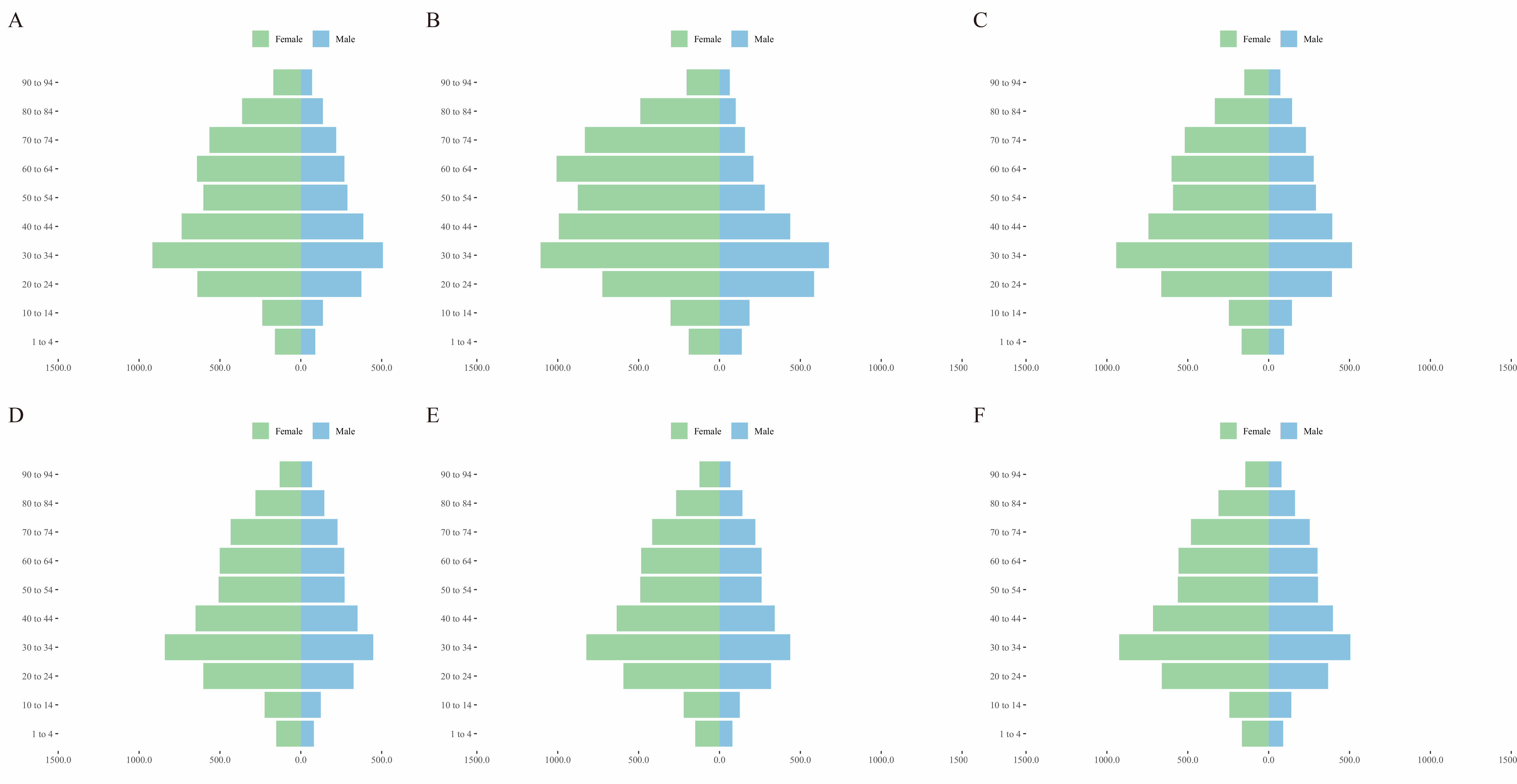
**

**Supplemental Figure 4: The correlation between EAPC and alopecia areata age-standardized rates in 1990 and SDI in 2019. The circles represent countries that were available on SDI data. The size of circle is increased with the cases of alopecia areata. The ρ indices Pearson’s correlation coefficient and p values were derived from Pearson’s correlation analysis. (A) EAPC and ASIR. (B) EAPC and SDI in incidence. (C) EAPC and age-standardized DALY rate. (D) EAPC and SDI in DALYs.** Abbreviations: EAPC = estimated annual percentage change. SDI = socio-demographic index. ASIR = age standardized incidence rate. DALY = disability adjusted life-year.


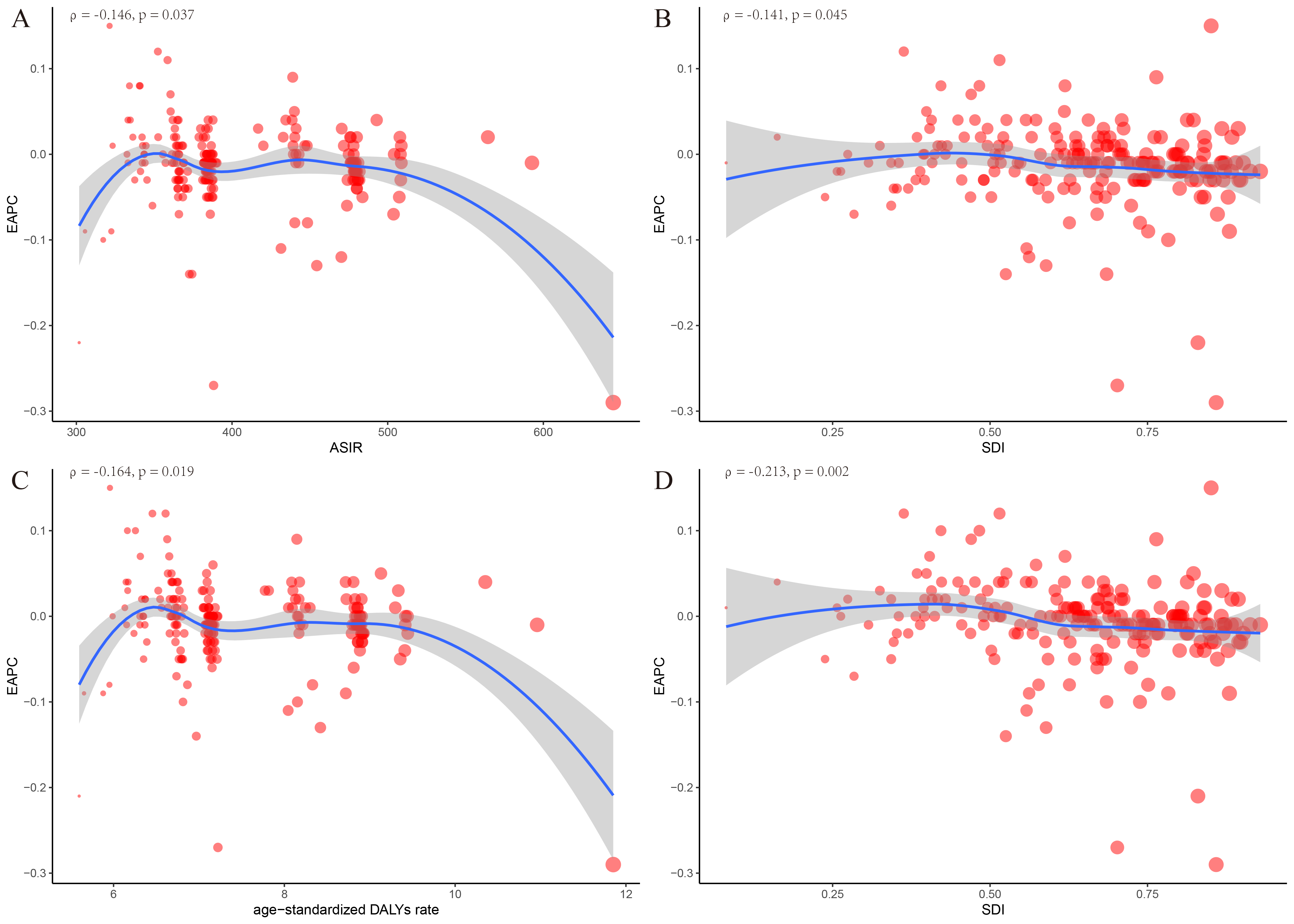


**Supplemental Figure 5: The incidence, and DALY rates of alopecia areata in different age groups. (A) incidence in 1990. (B) incidence in 2019. (C) DALY rate in 1990. (D) DALY rate in 2019.** Abbreviations: DALY = disability adjusted life-year.


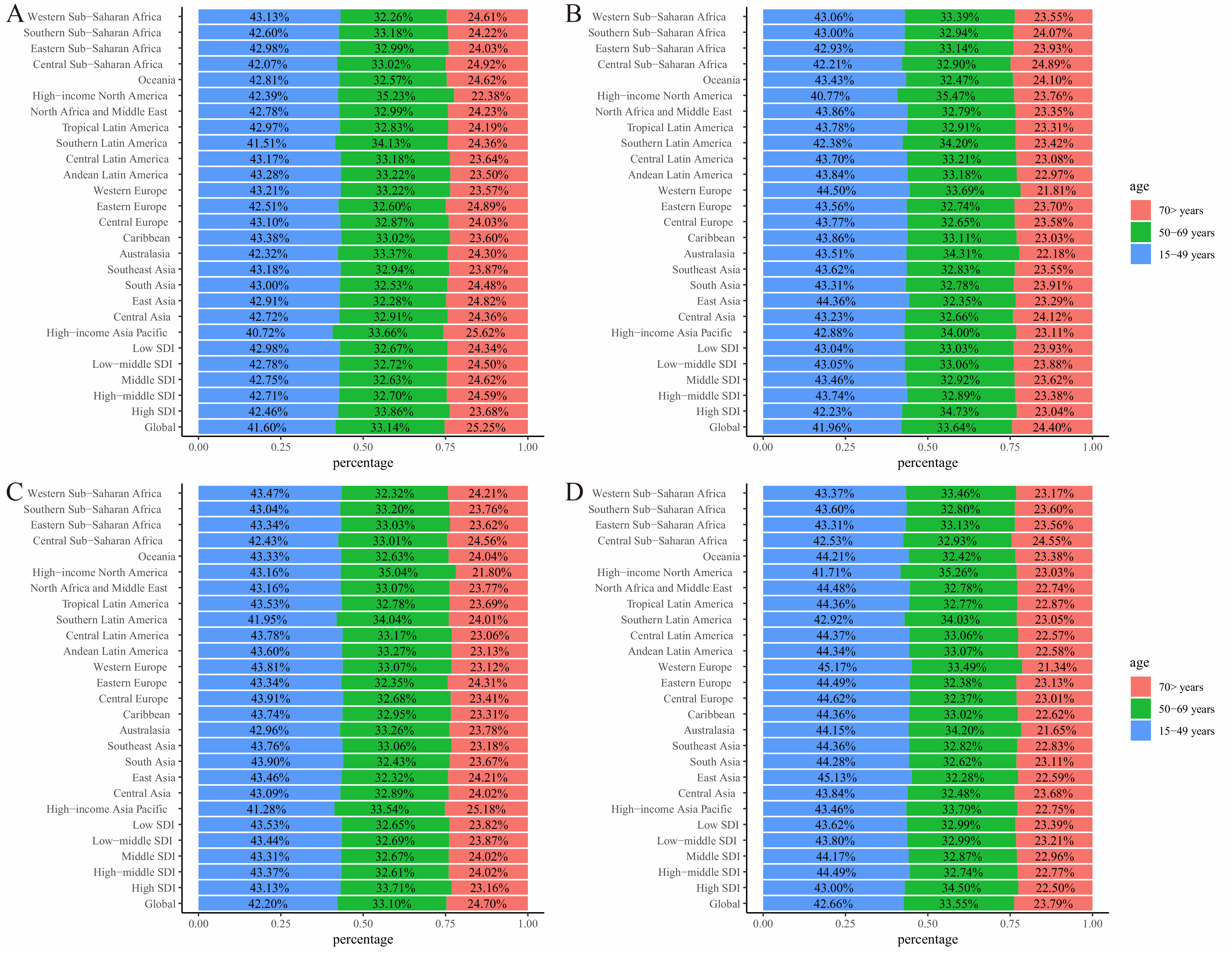


**Supplemental Figure 6: The proportion of different ages in alopecia areata incidence (A) and age-standardized DALY (B) by years.** Abbreviations: DALY = disability adjusted life-year.


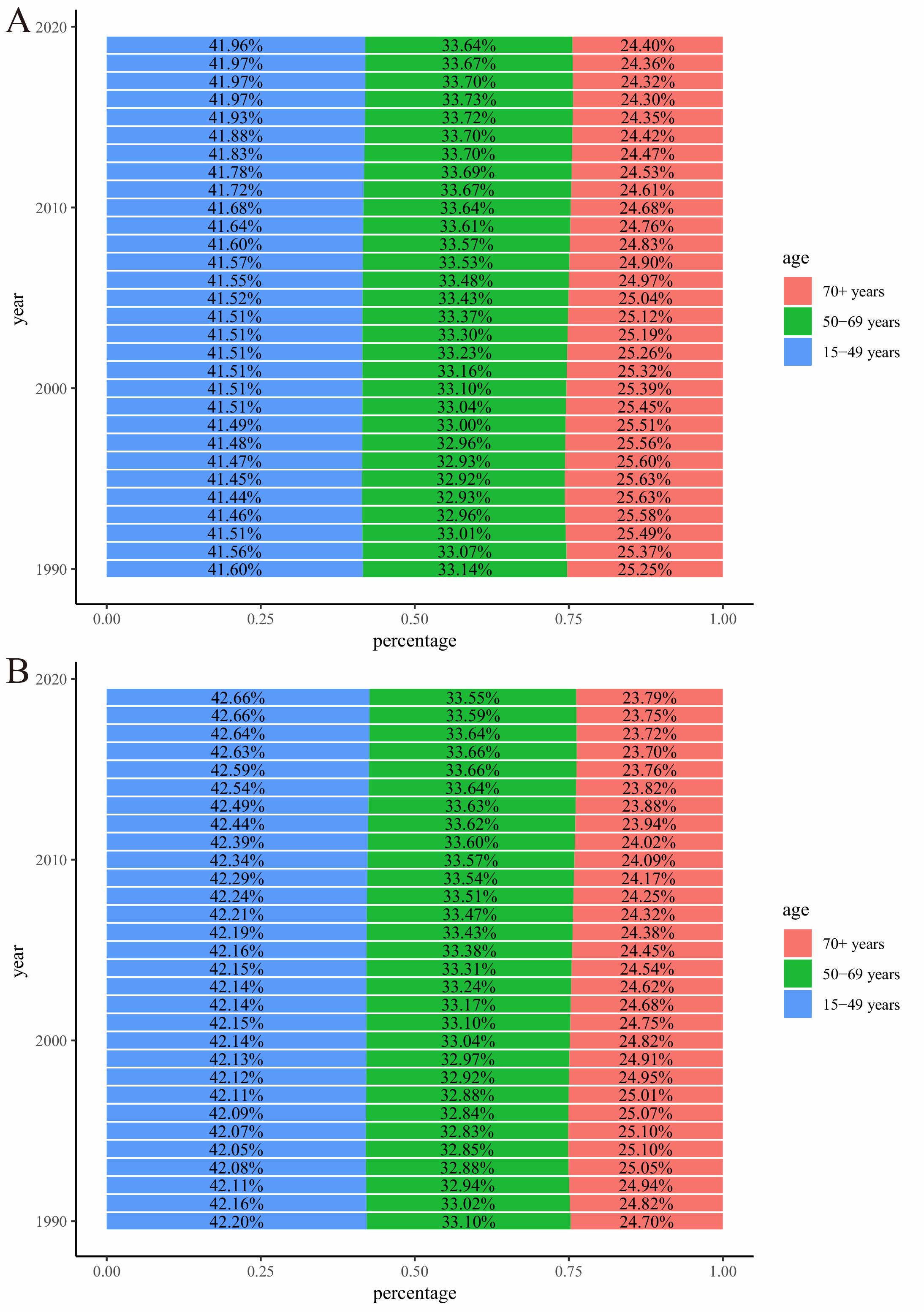


**Supplemental Figure 7: The ratio of male to female age standardized DALY rate among different age groups in 2019. (A) Global. (B) High SDI. (C) High-middle SDI. (D) Middle SDI. (E) Middle-low SDI. (F) Low SDI.** Abbreviations: SDI = socio-demographic index.


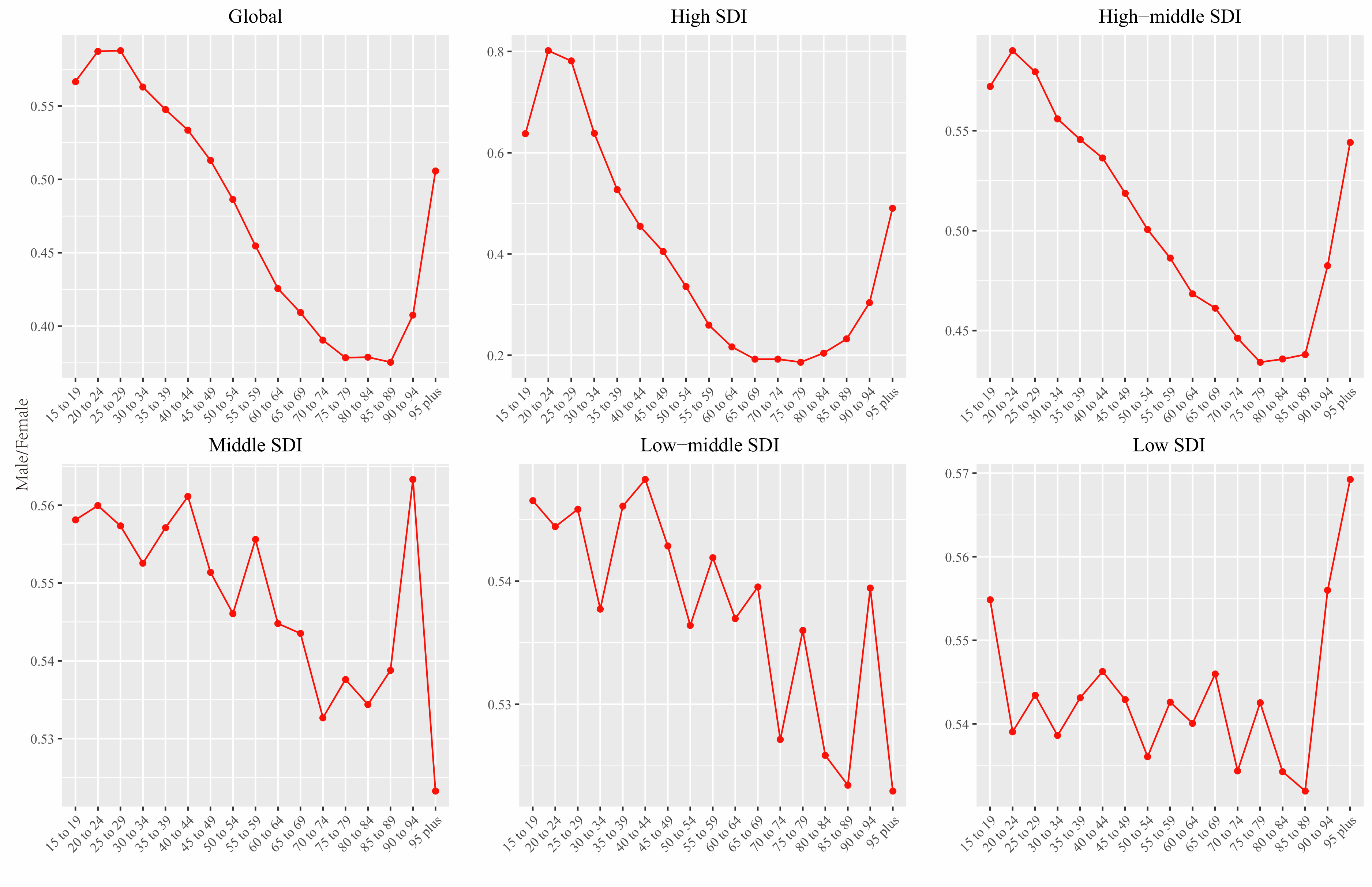


**Supplemental Figure 8. Distribution of different ages in alopecia areata DALYs in global (A), high SDI (B), high-middle SDI (C), middle SDI (D), middle-low SDI (E), low SDI (F).** Abbreviations: SDI, socio-demographic index, DALY = disability adjusted life-year.

**
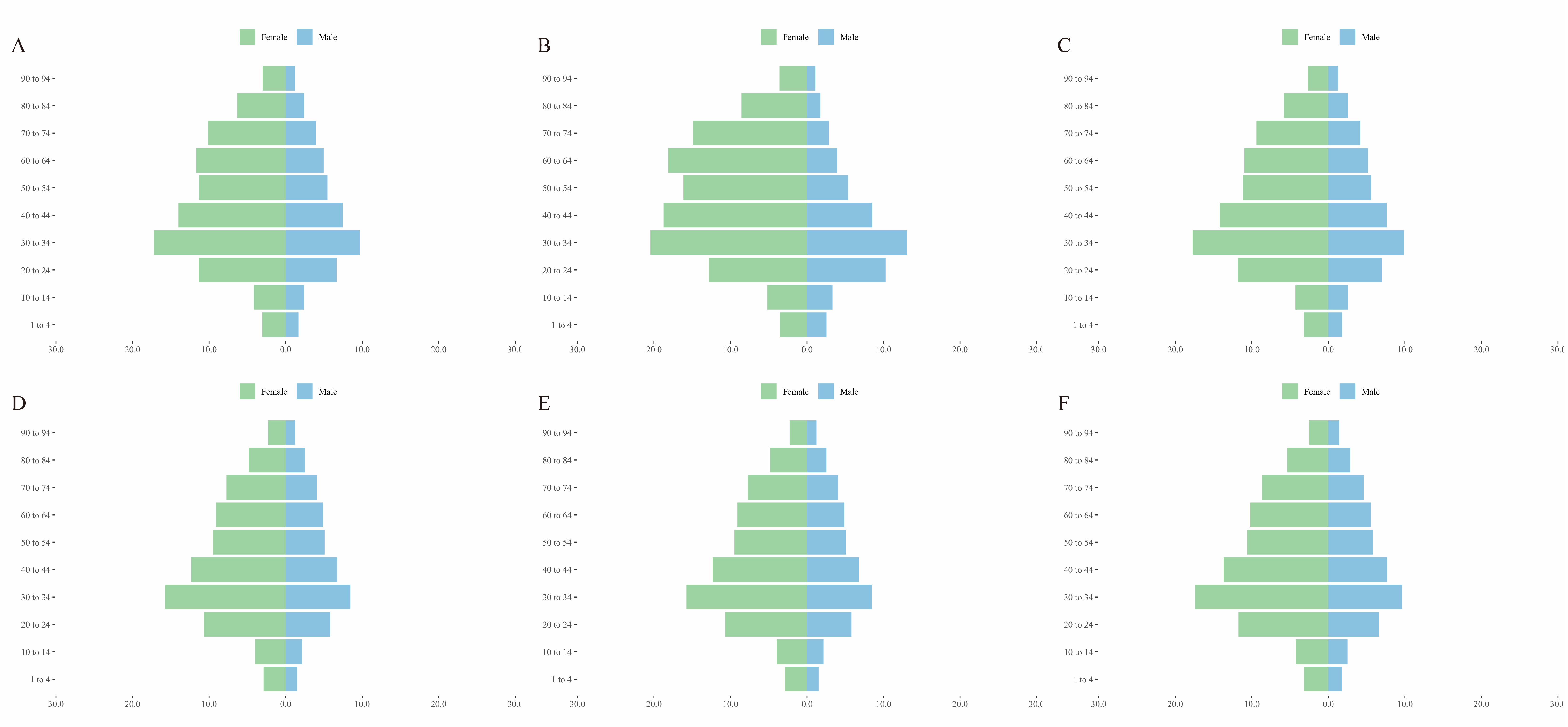
**
