# Supplementary material for: Epidemiological Trends in Alopecia Areata at the Global, Regional, and National Levels
Source: Front Immunol. 2022 Jul 14;13:874677. doi: 10.3389/fimmu.2022.874677 (PMC9331164; doi:10.3389/fimmu.2022.874677)
Supplement: Supplementary file 2 [file Table_1.doc]

**Supplementary table 1. The top three and the bottom three countries of alopecia areata incidence, or DALY.**

| **Measure** | **sex** | | **Top three countries** | | | | | **Bottom three countries** | | |
| --- | --- | --- | --- | --- | --- | --- | --- | --- | --- | --- |
| **2019 ASR (per 100,000 people)** | | | | | | | | | | |
| **ASIR** |  | |  | | |  |  |  |  |  |
|  | both | | Canada(591.35) | | | Georgia(601.43) | United States of America(627.64) | Qatar(290.74) | United Arab Emirates(302.52) | Oman(311.63) |
|  | female | | Canada(846.50) | | | Georgia(502.01) | United States of America(895.03) | Qatar(447.51) | United Arab Emirates(447.51) | Oman(447.51) |
|  | male | | Norway(397.29) | | | Sweden(397.29) | Italy(397.29) | Turkey(219.75) | Bhutan(235.00) | Bangladesh(235.00) |
| **Age-standardized DALY rate** | | | | | |  |  |  |  |  |
|  | both | | Greenland(10.51) | | | Canada(10.93) | United States of America(11.48) | Qatar(5.39) | United Arab Emirates(5.61) | Oman(5.79) |
|  | female | | Greenland(15.46) | | | Canada(15.55) | United States of America(16.24) | India(8.13) | Afghanistan(8.13) | Bangladesh(8.13) |
|  | male | | Israel(7.47) | | | Spain(7.47) | Italy(7.47) | Turkey(4.11) | Bhutan(4.38) | India(4.38) |
| **1990-2019 increase times** | | | | | | | | | | |
| **Incidence (cases)** | | | | | | | | | | |
|  | both | | Equatorial Guinea(2.54) | | United Arab Emirates(4.58) | | Qatar(6.04) | Georgia(-0.33) | Latvia(-0.28) | Bosnia and Herzegovina(-0.26) |
|  | female | | Jordan(2.46) | | United Arab Emirates(3.73) | | Qatar(4.76) | Georgia(-0.34) | Latvia(-0.28) | Bosnia and Herzegovina(-0.24) |
|  | male | | Equatorial Guinea(3.34) | | United Arab Emirates(5.28) | | Qatar(6.99) | Georgia(-0.32) | Bosnia and Herzegovina(-0.28) | Latvia(-0.27) |
| **DALY (cases)** | | |  | |  | |  |  |  |  |
|  | | both | Equatorial Guinea(2.57) | | United Arab Emirates(4.65) | | Qatar(6.04) | Georgia(-0.34) | Bosnia and Herzegovina(-0.26) | Latvia(-0.28) |
|  | | female | Jordan(2.49) | | United Arab Emirates(3.77) | | Qatar(4.77) | Georgia(-0.34) | Latvia(-0.28) | Bosnia and Herzegovina(-0.25) |
|  | | male | Equatorial Guinea(2.20) | | United Arab Emirates(3.77) | | Qatar(4.77) | Georgia(-0.34) | Bosnia and Herzegovina(-0.25) | Latvia(-0.28) |
| **EAPC** | | | | | | | | | | |
| **Incidence** |  | | |  |  | |  |  |  |  |
|  | both | | | Nigeria(0.11) | South Sudan(0.12) | | Kuwait(0.15) | United States of America(-0.29) | Georgia(-0.27) | Qatar(-0.22) |
|  | female | | | United Kingdom(0.00) | Netherlands(0.00) | | United Republic of Tanzania(0.03) | United States of America(-0.27) | Georgia(-0.22) | Sri Lanka(0.00) |
|  | male | | | United Kingdom(0.00) | Turkey(0.00) | | Egypt(0.00) | Georgia(-0.28) | United States of America(-0.25) | Sri Lanka(-0.03) |
| **DALY** |  | | |  |  | |  |  |  |  |
|  | both | | | South Sudan(0.12) | Nigeria(0.12) | | Kuwait(0.15) | United States of America(-0.29) | Georgia(-0.27) | Qatar(-0.21) |
|  | female | | | Maldives(0.03) | Uganda(0.04) | | United Republic of Tanzania(0.06) | United States of America(-0.27) | Georgia(-0.22) | Eswatini(-0.04) |
|  | male | | | Uganda(0.04) | Eritrea(0.04) | | Equatorial Guinea(0.04) | Georgia(-0.28) | United States of America(-0.25) | Eswatini(-0.04) |

**Supplementary table 2. The top three and the bottom three regions of alopecia areata incidence, or DALY.**

| **Measure** | **sex** | | **Top three regions** | | | **Bottom three regions** | | | |
| --- | --- | --- | --- | --- | --- | --- | --- | --- | --- |
| **2019 ASIR (per 100,000 people)** | | | | | | | | | |
| **ASIR** | | | | | | | | | |
|  | both | | High-income North America (624.02) | Southern Latin America (507.87) | Australasia(505.63) | North Africa and Middle East(338.48) | South Asia(338.74) | Central Sub-Saharan Africa (363.93) | |
|  | female | | Australasia (636.51) | Southern Latin America (635.96) | High-income North America(890.17) | North Africa and Middle East(447.93) | South Asia(444.44) | Eastern Sub-Saharan Africa (467.23) | |
|  | male | | Western Europe (396.17) | Southeast Asia (378.84) | High-income North America(349.05) | North Africa and Middle East(237.87) | South Asia(236.16) | Central Sub-Saharan Africa (252.12) | |
| **Age-standardized DALY rate** | | | |  |  |  |  |  | |
|  | both | | Southern Latin America (9.41) | High-income Asia Pacific (9.35) | High-income North America(11.43) | South Asia(6.23) | Central Sub-Saharan Africa (6.70) | North Africa and Middle East(6.26) | |
|  | female | | High-income Asia Pacific (11.73) | Southern Latin America (11.70) | High-income North America(16.17) | South Asia(8.13) | North Africa and Middle East (8.25) | Eastern Sub-Saharan Africa (8.61) | |
|  | male | | High-income Asia Pacific(6.97) | Southeast Asia(7.07) | Western Europe(7.45) | South Asia(4.38) | Central Sub-Saharan Africa (4.67) | North Africa and Middle East(4.44) | |
| **1990-2019 increase times** | | | |  |  |  |  |  | |
| **Incidence (cases)** | | | | | | | | | |
|  | both | | Eastern Sub-Saharan Africa (1.30) | Western Sub-Saharan Africa (1.47) | Central Sub-Saharan Africa (1.47) | Eastern Europe (-0.06) | Central Europe(-0.04) | High-income Asia Pacific (0.06) | |
|  | female | | Eastern Sub-Saharan Africa (1.30) | Central Sub-Saharan Africa (1.46) | Western Sub-Saharan Africa (1.52) | Eastern Europe (-0.05) | Central Europe(-0.05) | High-income Asia Pacific (0.12) | |
|  | male | | Eastern Sub-Saharan Africa (1.31) | Western Sub-Saharan Africa (1.37) | Central Sub-Saharan Africa (1.50) | Eastern Europe (-0.06) | Central Europe(-0.04) | High-income Asia Pacific(-0.04) | |
| **DALY (cases)** | | |  |  |  |  |  |  | |
|  | | both | Eastern Sub-Saharan Africa (1.31) | Western Sub-Saharan Africa (1.48) | Central Sub-Saharan Africa (1.48) | Eastern Europe (-0.05) | Central Europe(-0.04) | High-income Asia Pacific (0.07) | |
|  | | female | Eastern Sub-Saharan Africa (1.31) | Central Sub-Saharan Africa (1.47) | Western Sub-Saharan Africa (1.53) | Eastern Europe (-0.05) | Central Europe(-0.04) | High-income Asia Pacific (0.12) | |
|  | | male | Eastern Sub-Saharan Africa (1.32) | Western Sub-Saharan Africa (1.38) | Central Sub-Saharan Africa (1.51) | Eastern Europe (-0.05) | Central Europe (-0.04) | High-income Asia Pacific(-0.03) | |
| **EAPC** | | | | | | | | | |
| **Incidence** | |  |  |  |  |  |  | |  |
|  | | both | South Asia (0.04) | Western Sub-Saharan Africa (0.05) | East Asia (0.01) | High-income Asia Pacific (-0.03) | Central Asia (-0.02) | | High-income North America(-0.26) |
|  | | female | Eastern Sub-Saharan Africa (0.03) | Central Europe (0.01) | Southeast Asia (0.00) | High-income Asia Pacific (-0.02) | Central Latin America (-0.00) | | High-income North America(-0.24) |
|  | | male | North Africa and Middle East (0.02) | Central Europe (0.01) | Southern Sub-Saharan Africa (0.00) | High-income North America (-0.22) | Eastern Sub-Saharan Africa (-0.002) | | Southeast Asia (-0.002) |
| **DALY** | |  |  |  |  |  |  | |  |
|  | | both | South Asia(0.05) | Western Sub-Saharan Africa(0.07) | East Asia (0.03) | High-income Asia Pacific (-0.03) | Southern Sub-Saharan Africa (-0.03) | | High-income North America(-0.26) |
|  | | female | South Asia (0.02) | Central Sub-Saharan Africa (0.03) | Eastern Sub-Saharan Africa(0.03) | Southern Sub-Saharan Africa (-0.02) | Caribbean (-0.00) | | High-income North America(-0.25) |
|  | | male | Central Sub-Saharan Africa (0.03) | North Africa and Middle East (0.02) | Eastern Europe (0.02) | High-income North America(-0.23) | Southern Sub-Saharan Africa (-0.01) | | Oceania (-0.01) |

**Supplementary table 3. The incidence cases and age-standardized incidence rate of alopecia areata in 1990 and 2019, and its temporal trends from 1990 to 2019.**

| **Nation** | **Sex** | **Incident Cases No. (95% UI)** | | **Change in absolute number (%)** | **ASIR per 100,000 No.(95% UI)** | | **1990-2019 EAPC No. (95%CI)** |
| --- | --- | --- | --- | --- | --- | --- | --- |
|  |  | **1990** | **2019** |  | **1990** | **2019** |  |
| Afghanistan | both | 32745.26(31514.49,33951.18) | 112663.96(107978.71,117189.60) | 2.44 | 226.88(204.75,251.76) | 341.32(328.57,354.26) | -0.06(-0.07,-0.05) |
| Albania | both | 12265.04(11792.24,12734.06) | 11011.62(10611.71,11374.01) | -0.10 | 106.83(96.09,117.50) | 380.83(366.83,394.05) | 0.02(0.00,0.03) |
| Algeria | both | 76872.80(73675.04,79944.41) | 149260.72(143259.95,155021.23) | 0.94 | 211.89(191.35,234.05) | 341.88(329.10,354.79) | -0.01(-0.01,-0.01) |
| American Samoa | both | 199.17(191.02,207.35) | 239.50(230.73,247.89) | 0.20 | 541.12(501.30,586.69) | 444.52(428.23,461.14) | 0.03(0.03,0.03) |
| Andorra | both | 293.14(280.98,304.30) | 430.86(414.86,445.19) | 0.47 | 126.55(113.49,140.97) | 472.47(454.60,489.29) | 0.03(0.02,0.04) |
| Angola | both | 31963.67(30635.44,33245.67) | 94796.07(90916.32,98589.79) | 1.97 | 207.59(191.16,225.80) | 367.82(352.96,382.16) | 0.07(0.07,0.08) |
| Antigua and Barbuda | both | 233.29(224.40,241.89) | 370.90(357.37,383.70) | 0.59 | 313.99(292.29,338.96) | 384.91(370.70,398.37) | -0.03(-0.05,-0.01) |
| Argentina | both | 164684.65(158532.27,170542.26) | 239236.73(230050.43,247791.04) | 0.45 | 172.35(159.20,185.96) | 508.04(488.91,526.26) | 0.00(-0.01,0.00) |
| Armenia | both | 13325.67(12802.89,13848.56) | 12607.98(12120.40,13047.16) | -0.05 | 156.94(144.56,169.94) | 385.40(371.21,398.78) | -0.01(-0.02,0.00) |
| Australia | both | 90238.40(86761.03,93498.48) | 131492.67(126659.26,135870.17) | 0.46 | 504.16(485.21,522.29) | 504.77(485.73,522.97) | 0.00(0.00,0.00) |
| Austria | both | 40779.03(39274.85,42236.13) | 45508.38(43905.51,47068.90) | 0.12 | 481.78(463.92,498.74) | 477.23(459.33,494.11) | -0.03(-0.03,-0.03) |
| Azerbaijan | both | 27831.63(26744.19,28948.26) | 43098.60(41449.67,44706.32) | 0.55 | 387.67(373.45,401.06) | 382.81(368.66,396.08) | -0.05(-0.05,-0.05) |
| Bahamas | both | 999.06(959.52,1038.00) | 1567.70(1510.42,1621.59) | 0.57 | 386.12(371.95,399.55) | 386.05(371.87,399.50) | 0.00(0.00,0.01) |
| Bahrain | both | 1739.28(1657.10,1817.84) | 5352.27(5131.10,5577.09) | 2.08 | 322.45(310.69,334.21) | 320.56(308.79,332.21) | -0.09(-0.11,-0.06) |
| Bangladesh | both | 319262.70(306317.15,332189.68) | 559171.47(537522.23,580606.98) | 0.75 | 334.11(321.61,346.27) | 340.89(328.15,353.43) | 0.08(0.07,0.09) |
| Barbados | both | 1021.36(982.74,1058.17) | 1224.69(1180.38,1263.71) | 0.20 | 386.74(372.54,400.14) | 385.24(371.06,398.64) | -0.01(-0.01,-0.01) |
| Belarus | both | 42649.35(41068.51,44142.75) | 39644.49(38194.20,40976.28) | -0.07 | 387.42(373.23,400.78) | 385.29(371.15,398.62) | -0.01(-0.02,-0.01) |
| Belgium | both | 51527.58(49618.73,53334.23) | 56696.77(54738.88,58614.79) | 0.10 | 478.56(460.81,495.45) | 476.44(458.59,493.30) | -0.02(-0.02,-0.02) |
| Belize | both | 616.97(593.61,641.08) | 1586.30(1526.26,1644.79) | 1.57 | 379.76(365.68,392.90) | 383.04(368.93,396.50) | 0.03(0.03,0.03) |
| Benin | both | 14639.30(14035.09,15236.96) | 39909.84(38207.51,41534.61) | 1.73 | 369.83(354.80,384.29) | 365.76(350.95,379.98) | -0.04(-0.04,-0.03) |
| Bermuda | both | 255.16(245.37,264.70) | 262.78(253.38,271.10) | 0.03 | 385.11(370.94,398.52) | 384.14(370.02,397.45) | -0.01(-0.01,0.00) |
| Bhutan | both | 1829.70(1754.92,1904.78) | 2680.31(2571.93,2789.25) | 0.46 | 333.16(320.94,345.31) | 333.53(321.25,345.66) | -0.01(-0.02,0.00) |
| Bolivia (Plurinational State of) | both | 21896.53(21082.27,22720.18) | 45027.87(43343.79,46651.80) | 1.06 | 385.28(371.12,398.71) | 382.50(368.38,395.84) | -0.03(-0.03,-0.03) |
| Bosnia and Herzegovina | both | 18359.50(17678.07,19025.33) | 13654.40(13156.33,14082.13) | -0.26 | 381.99(368.01,395.33) | 382.50(368.36,395.81) | -0.01(-0.02,0.00) |
| Botswana | both | 4196.52(4019.86,4364.00) | 8843.35(8466.75,9211.63) | 1.11 | 369.68(354.74,384.10) | 364.99(350.30,378.95) | -0.04(-0.04,-0.04) |
| Brazil | both | 555623.12(533136.85,576904.51) | 895910.06(861218.27,928523.85) | 0.61 | 386.63(372.21,400.34) | 386.78(372.41,400.44) | 0.00(0.00,0.00) |
| Brunei Darussalam | both | 1276.57(1221.78,1330.70) | 2424.83(2325.78,2525.50) | 0.90 | 492.93(475.03,511.41) | 498.09(480.08,516.67) | 0.04(0.03,0.05) |
| Bulgaria | both | 35084.20(33796.74,36259.68) | 28343.66(27371.89,29248.83) | -0.19 | 382.81(368.66,396.11) | 380.84(366.72,394.11) | -0.02(-0.03,-0.02) |
| Burkina Faso | both | 28645.62(27526.26,29755.45) | 71540.68(68602.30,74389.38) | 1.50 | 371.49(356.39,385.96) | 368.85(353.91,383.26) | -0.02(-0.03,-0.02) |
| Burundi | both | 17212.93(16546.04,17888.52) | 37492.73(36053.68,38952.59) | 1.18 | 365.87(351.95,378.93) | 359.90(346.39,372.88) | -0.07(-0.08,-0.07) |
| Cabo Verde | both | 1105.35(1059.45,1149.78) | 2103.81(2015.52,2190.36) | 0.90 | 374.31(359.33,388.71) | 360.50(346.14,374.07) | -0.14(-0.14,-0.13) |
| Cambodia | both | 42848.33(41260.13,44524.57) | 80456.32(77401.06,83533.89) | 0.88 | 483.79(466.72,501.65) | 477.54(460.93,494.67) | -0.05(-0.05,-0.04) |
| Cameroon | both | 32121.03(30807.72,33398.19) | 96371.31(92266.56,100317.40) | 2.00 | 365.74(350.90,379.96) | 363.42(348.72,377.52) | -0.03(-0.03,-0.02) |
| Canada | both | 176153.17(169519.09,182405.85) | 233408.17(225087.40,241436.94) | 0.33 | 592.68(571.16,613.27) | 591.35(569.75,612.09) | -0.01(-0.01,-0.01) |
| Central African Republic | both | 8709.18(8337.54,9057.06) | 17312.86(16585.08,18007.79) | 0.99 | 365.93(351.22,380.16) | 366.41(351.59,380.62) | 0.00(-0.01,0.01) |
| Chad | both | 18106.79(17380.76,18821.74) | 47961.54(45945.56,49896.39) | 1.65 | 368.40(353.45,382.74) | 363.46(348.68,377.57) | -0.05(-0.05,-0.05) |
| Chile | both | 67813.90(65231.70,70382.73) | 99227.57(95477.57,102705.99) | 0.46 | 508.78(489.58,527.03) | 507.08(487.95,525.25) | -0.01(-0.01,-0.01) |
| China | both | 5151928.50(4941932.77,5360361.61) | 6667732.76(6414818.06,6920700.61) | 0.29 | 420.12(404.46,435.61) | 420.95(405.26,436.50) | 0.01(0.01,0.02) |
| Colombia | both | 120262.59(115611.61,124872.42) | 192566.80(185410.13,199214.05) | 0.60 | 384.58(370.41,397.94) | 384.92(370.72,398.28) | 0.00(0.00,0.00) |
| Comoros | both | 1431.02(1377.64,1483.03) | 2533.57(2438.37,2627.23) | 0.77 | 363.73(349.78,376.78) | 361.27(347.55,373.95) | -0.03(-0.03,-0.03) |
| Congo | both | 7676.80(7353.41,7978.51) | 18148.97(17403.19,18874.88) | 1.36 | 366.28(351.57,380.38) | 363.42(348.75,377.53) | -0.03(-0.03,-0.02) |
| Cook Islands | both | 79.18(76.11,82.13) | 81.34(78.51,84.10) | 0.03 | 438.98(422.59,455.53) | 449.64(433.39,466.67) | 0.09(0.08,0.10) |
| Costa Rica | both | 11100.42(10666.20,11529.66) | 19439.30(18713.36,20131.43) | 0.75 | 383.06(368.95,396.43) | 386.32(372.11,399.75) | 0.03(0.03,0.04) |
| Côte d'Ivoire | both | 37639.39(35991.90,39192.35) | 86607.55(82918.84,90171.76) | 1.30 | 357.32(343.18,371.04) | 357.80(343.46,371.63) | -0.01(-0.01,0.00) |
| Croatia | both | 20165.22(19411.30,20863.28) | 17328.93(16703.34,17873.65) | -0.14 | 383.56(369.43,396.89) | 381.67(367.52,394.91) | -0.02(-0.02,-0.02) |
| Cuba | both | 43567.39(41961.65,45100.70) | 46237.93(44604.40,47695.11) | 0.06 | 381.72(367.59,395.08) | 380.79(366.65,394.01) | -0.01(-0.01,-0.01) |
| Cyprus | both | 3868.32(3720.39,4008.46) | 7045.67(6775.17,7296.10) | 0.82 | 478.05(460.22,494.94) | 478.59(460.57,495.57) | 0.01(0.00,0.01) |
| Czechia | both | 41480.20(40011.11,42913.69) | 43160.97(41657.75,44581.35) | 0.04 | 383.57(369.40,396.92) | 380.53(366.41,393.74) | -0.03(-0.03,-0.03) |
| Democratic People's Republic of Korea | both | 89514.04(86044.67,92941.94) | 120116.37(115559.49,124669.90) | 0.34 | 431.45(415.34,447.31) | 419.73(404.18,435.19) | -0.11(-0.12,-0.11) |
| Democratic Republic of the Congo | both | 118435.86(113581.64,123139.35) | 281900.09(269993.08,293013.09) | 1.38 | 365.64(350.92,379.78) | 362.61(348.12,376.53) | -0.03(-0.03,-0.03) |
| Denmark | both | 26537.84(25582.13,27485.94) | 28783.23(27804.94,29750.47) | 0.08 | 478.03(460.26,494.93) | 475.96(458.07,492.79) | -0.01(-0.02,-0.01) |
| Djibouti | both | 1486.54(1428.64,1545.29) | 4238.70(4061.24,4403.92) | 1.85 | 352.55(339.18,364.69) | 354.82(341.15,367.36) | 0.02(0.02,0.03) |
| Dominica | both | 266.31(256.78,275.60) | 271.83(262.00,280.93) | 0.02 | 382.51(368.39,395.71) | 379.04(365.04,392.21) | -0.03(-0.03,-0.02) |
| Dominican Republic | both | 25834.53(24859.23,26825.91) | 42340.35(40745.32,43849.84) | 0.64 | 385.33(371.13,398.79) | 381.32(367.21,394.57) | -0.05(-0.06,-0.04) |
| Ecuador | both | 35471.37(34129.00,36795.04) | 67995.34(65458.55,70423.19) | 0.92 | 383.55(369.43,396.91) | 383.21(369.05,396.59) | 0.00(0.00,0.00) |
| Egypt | both | 173523.00(166088.80,180576.08) | 333669.98(319772.27,347278.97) | 0.92 | 341.21(327.27,354.17) | 339.36(325.91,352.58) | -0.02(-0.02,-0.02) |
| El Salvador | both | 18293.48(17619.46,18979.20) | 24804.34(23879.50,25665.56) | 0.36 | 387.71(373.39,401.17) | 392.43(377.96,406.13) | 0.04(0.04,0.05) |
| Equatorial Guinea | both | 1316.30(1263.01,1367.77) | 4665.37(4459.58,4860.55) | 2.54 | 372.55(357.45,387.04) | 359.56(345.36,373.26) | -0.14(-0.16,-0.12) |
| Eritrea | both | 9253.82(8899.03,9612.15) | 22529.03(21646.59,23413.20) | 1.43 | 364.94(351.14,377.82) | 361.99(348.35,374.75) | -0.03(-0.03,-0.03) |
| Estonia | both | 6373.91(6143.22,6588.94) | 5344.39(5153.37,5519.71) | -0.16 | 387.20(373.04,400.60) | 382.41(368.35,395.73) | -0.05(-0.05,-0.04) |
| Eswatini | both | 2492.39(2387.30,2594.24) | 4057.92(3886.12,4226.01) | 0.63 | 371.89(356.82,386.44) | 368.03(353.18,382.13) | -0.04(-0.04,-0.03) |
| Ethiopia | both | 156327.27(150284.61,162383.11) | 347844.79(334297.84,361736.33) | 1.23 | 364.40(351.57,377.52) | 362.78(350.04,375.77) | -0.01(-0.02,0.00) |
| Fiji | both | 3222.99(3092.65,3353.11) | 4088.18(3933.86,4244.12) | 0.27 | 442.87(426.44,459.39) | 442.45(425.96,458.92) | -0.01(-0.01,0.00) |
| Finland | both | 26064.80(25111.60,26991.23) | 27482.50(26535.33,28418.18) | 0.05 | 480.79(463.00,497.76) | 476.21(458.39,493.09) | -0.03(-0.03,-0.03) |
| France | both | 293118.48(282390.59,303664.84) | 323660.19(312638.12,334413.54) | 0.10 | 479.12(461.36,496.05) | 478.21(460.35,495.13) | -0.01(-0.01,-0.01) |
| Gabon | both | 3164.96(3034.18,3289.38) | 6304.47(6040.69,6562.39) | 0.99 | 361.90(347.59,375.51) | 366.23(351.42,380.44) | 0.04(0.04,0.04) |
| Gambia | both | 3045.08(2909.42,3171.78) | 7430.63(7105.66,7733.97) | 1.44 | 360.56(346.10,374.49) | 364.47(349.65,378.63) | 0.05(0.04,0.06) |
| Georgia | both | 22175.51(21358.13,22947.04) | 14763.21(14225.57,15244.87) | -0.33 | 388.16(373.94,401.59) | 601.43(582.38,620.67) | -0.27(-2.33,1.84) |
| Germany | both | 422910.42(407029.67,437876.38) | 427594.80(412298.79,442119.88) | 0.01 | 480.91(463.05,497.89) | 476.43(458.49,493.25) | -0.03(-0.03,-0.03) |
| Ghana | both | 47547.85(45562.18,49456.80) | 111827.01(107133.19,116433.16) | 1.35 | 364.89(350.04,379.09) | 367.87(353.00,382.20) | 0.04(0.03,0.04) |
| Greece | both | 52502.72(50649.91,54281.51) | 51708.44(49929.39,53425.97) | -0.02 | 478.15(460.32,495.10) | 478.02(460.18,494.93) | 0.00(0.00,0.00) |
| Greenland | both | 339.19(325.31,353.12) | 344.25(331.85,356.32) | 0.01 | 564.37(544.43,584.02) | 570.93(550.13,590.91) | 0.02(0.02,0.03) |
| Grenada | both | 300.42(289.34,311.34) | 413.88(398.69,427.97) | 0.38 | 384.59(370.39,397.96) | 378.74(364.76,391.91) | -0.05(-0.05,-0.05) |
| Guam | both | 611.98(586.90,636.93) | 749.12(722.58,776.09) | 0.22 | 434.50(418.31,450.85) | 439.08(422.66,455.44) | 0.04(0.02,0.06) |
| Guatemala | both | 26074.72(25125.50,27062.96) | 67910.74(65278.16,70480.04) | 1.60 | 384.67(370.47,398.06) | 388.51(374.21,402.05) | 0.04(0.03,0.05) |
| Guinea | both | 19248.03(18475.46,20017.19) | 40255.75(38580.53,41875.30) | 1.09 | 367.80(352.82,382.11) | 368.27(353.22,382.50) | 0.01(0.01,0.02) |
| Guinea-Bissau | both | 3119.25(2989.59,3244.74) | 6366.82(6090.35,6635.57) | 1.04 | 368.95(353.93,383.24) | 367.84(352.93,382.20) | -0.01(-0.01,-0.01) |
| Guyana | both | 2807.18(2699.51,2916.75) | 3032.98(2920.94,3138.74) | 0.08 | 383.82(369.69,397.24) | 384.19(370.05,397.64) | 0.00(0.00,0.01) |
| Haiti | both | 21877.34(21067.30,22707.76) | 47193.98(45363.89,48992.93) | 1.16 | 386.66(372.40,400.15) | 387.81(373.51,401.39) | 0.01(0.01,0.02) |
| Honduras | both | 15222.91(14652.28,15809.16) | 37011.99(35608.25,38396.33) | 1.43 | 384.71(370.51,398.10) | 387.90(373.57,401.42) | 0.03(0.03,0.04) |
| Hungary | both | 42244.13(40708.35,43687.78) | 39889.63(38464.48,41188.96) | -0.06 | 384.56(370.39,397.91) | 382.86(368.70,396.19) | -0.02(-0.02,-0.01) |
| Iceland | both | 1254.87(1206.72,1300.56) | 1720.08(1658.36,1778.68) | 0.37 | 476.19(458.38,493.08) | 474.84(456.97,491.69) | -0.01(-0.01,-0.01) |
| India | both | 2682756.39(2582315.97,2790366.03) | 4869486.98(4686454.96,5057346.15) | 0.82 | 336.28(324.03,348.77) | 338.29(325.89,350.92) | 0.02(0.02,0.03) |
| Indonesia | both | 844059.43(812219.77,875048.71) | 1303777.90(1257152.57,1351509.58) | 0.54 | 478.23(461.79,495.21) | 476.41(460.15,493.23) | -0.01(-0.01,-0.01) |
| Iran (Islamic Republic of) | both | 175566.81(168818.73,182175.19) | 315683.25(303194.75,327790.93) | 0.80 | 342.61(330.19,354.20) | 343.44(330.98,355.01) | 0.01(0.01,0.02) |
| Iraq | both | 53128.28(50912.15,55265.88) | 148922.12(142459.90,155023.48) | 1.80 | 355.59(341.92,368.88) | 355.20(341.53,368.46) | 0.00(0.00,0.01) |
| Ireland | both | 17166.83(16560.37,17771.13) | 24310.10(23465.05,25110.49) | 0.42 | 477.03(459.21,493.90) | 476.80(458.89,493.71) | -0.01(-0.01,0.00) |
| Israel | both | 23210.87(22366.74,24031.69) | 43805.71(42268.81,45343.74) | 0.89 | 479.82(461.98,496.81) | 477.75(459.92,494.65) | -0.02(-0.02,-0.02) |
| Italy | both | 296927.56(286946.45,307484.90) | 302569.48(292850.93,312845.94) | 0.02 | 481.97(464.70,499.62) | 479.86(462.58,497.43) | -0.02(-0.02,-0.01) |
| Jamaica | both | 8572.36(8248.24,8889.76) | 11468.60(11040.31,11875.13) | 0.34 | 385.17(371.00,398.52) | 383.19(369.05,396.64) | -0.02(-0.02,-0.02) |
| Japan | both | 688971.19(664271.41,712516.27) | 672933.62(649698.76,694960.19) | -0.02 | 508.41(490.91,526.65) | 504.31(487.01,522.42) | -0.03(-0.03,-0.03) |
| Jordan | both | 11022.14(10560.76,11467.27) | 38913.10(37381.54,40420.57) | 2.53 | 337.50(324.97,350.20) | 334.13(321.73,346.72) | -0.03(-0.05,-0.02) |
| Kazakhstan | both | 63069.91(60692.04,65467.25) | 73504.00(70745.91,76172.56) | 0.17 | 387.16(372.95,400.53) | 386.31(372.11,399.71) | -0.01(-0.01,0.00) |
| Kenya | both | 70060.35(67327.42,72811.36) | 171931.55(165232.83,178580.59) | 1.45 | 364.19(351.37,377.16) | 364.26(351.49,377.26) | 0.00(-0.01,0.00) |
| Kiribati | both | 307.50(295.10,320.01) | 516.47(496.17,536.22) | 0.68 | 448.22(432.02,464.94) | 449.82(433.59,466.57) | 0.01(0.01,0.01) |
| Kuwait | both | 6029.13(5755.46,6298.55) | 17608.53(16838.18,18368.04) | 1.92 | 321.35(309.51,333.08) | 334.76(322.26,347.29) | 0.15(0.11,0.19) |
| Kyrgyzstan | both | 16064.87(15458.36,16673.09) | 25108.12(24151.30,26026.85) | 0.56 | 386.25(372.09,399.60) | 384.72(370.52,398.10) | -0.01(-0.01,-0.01) |
| Lao People's Democratic Republic | both | 17246.51(16623.17,17898.05) | 34231.10(32932.42,35595.14) | 0.98 | 477.94(461.08,495.19) | 474.29(457.65,491.27) | -0.03(-0.03,-0.03) |
| Latvia | both | 10847.45(10450.98,11207.17) | 7815.20(7538.53,8066.58) | -0.28 | 387.74(373.54,401.14) | 384.33(370.20,397.63) | -0.03(-0.04,-0.03) |
| Lebanon | both | 10306.01(9917.56,10705.50) | 18648.32(17896.91,19382.64) | 0.81 | 344.66(331.67,357.74) | 345.37(332.32,358.50) | 0.01(0.01,0.02) |
| Lesotho | both | 5823.73(5585.12,6049.14) | 7651.98(7328.70,7960.77) | 0.31 | 365.94(351.12,380.08) | 364.05(349.62,377.70) | -0.02(-0.03,-0.01) |
| Liberia | both | 6051.42(5810.79,6285.17) | 16162.03(15475.11,16814.83) | 1.67 | 365.01(350.14,379.16) | 361.20(346.64,375.19) | -0.04(-0.05,-0.04) |
| Libya | both | 12271.34(11773.46,12756.17) | 25293.65(24260.32,26276.03) | 1.06 | 333.11(320.72,345.53) | 339.41(326.79,352.23) | 0.04(0.03,0.05) |
| Lithuania | both | 14909.21(14364.17,15417.84) | 11432.12(11027.68,11784.51) | -0.23 | 387.08(372.86,400.49) | 384.66(370.54,397.98) | -0.02(-0.03,-0.02) |
| Luxembourg | both | 2018.52(1940.30,2090.18) | 3196.31(3081.52,3304.04) | 0.58 | 478.97(461.19,495.92) | 474.65(456.76,491.48) | -0.03(-0.03,-0.03) |
| Madagascar | both | 36846.16(35438.94,38245.09) | 87981.09(84599.48,91390.09) | 1.39 | 361.48(347.70,374.36) | 361.76(347.95,374.62) | 0.00(0.00,0.00) |
| Malawi | both | 29581.75(28441.63,30696.55) | 59836.35(57515.41,62144.02) | 1.02 | 364.08(350.24,377.06) | 365.35(351.45,378.48) | 0.02(0.01,0.02) |
| Malaysia | both | 79685.59(76649.12,82886.81) | 157285.22(151336.40,163292.60) | 0.97 | 474.19(457.50,491.15) | 470.58(454.10,487.32) | -0.03(-0.03,-0.03) |
| Maldives | both | 875.76(842.71,909.98) | 2607.44(2493.64,2720.79) | 1.98 | 470.22(453.42,487.16) | 453.36(438.04,469.20) | -0.12(-0.16,-0.08) |
| Mali | both | 26416.89(25373.74,27461.19) | 66777.95(63977.12,69446.47) | 1.53 | 366.55(351.60,380.84) | 364.12(349.37,378.30) | -0.02(-0.03,-0.01) |
| Malta | both | 1870.81(1802.61,1934.84) | 2241.07(2160.25,2319.43) | 0.20 | 480.14(462.35,497.06) | 475.48(457.64,492.33) | -0.04(-0.04,-0.03) |
| Marshall Islands | both | 169.34(162.56,176.13) | 250.53(240.65,260.30) | 0.48 | 440.94(424.49,457.32) | 440.51(423.96,456.98) | -0.01(-0.01,-0.01) |
| Mauritania | both | 6443.35(6177.46,6697.32) | 13314.06(12753.70,13833.86) | 1.07 | 364.74(349.95,378.90) | 365.09(350.28,379.27) | 0.01(0.01,0.01) |
| Mauritius | both | 5389.98(5176.88,5601.05) | 6604.71(6368.41,6843.96) | 0.23 | 474.93(458.33,491.89) | 474.71(458.04,491.65) | 0.00(0.00,0.00) |
| Mexico | both | 303673.52(291595.70,315479.71) | 500754.46(481815.19,519034.20) | 0.65 | 387.40(372.93,401.23) | 387.49(373.07,401.26) | 0.00(0.00,0.01) |
| Micronesia (Federated States of) | both | 397.94(382.56,413.72) | 447.81(430.28,464.50) | 0.13 | 441.07(424.63,457.46) | 442.27(425.80,458.66) | 0.00(-0.01,0.01) |
| Monaco | both | 158.29(152.57,163.77) | 183.31(177.03,189.22) | 0.16 | 479.49(461.61,496.51) | 477.75(459.85,494.70) | -0.01(-0.02,-0.01) |
| Mongolia | both | 7395.82(7111.56,7694.75) | 13507.07(12990.22,14017.21) | 0.83 | 383.05(368.91,396.34) | 384.94(370.76,398.33) | 0.02(0.01,0.02) |
| Montenegro | both | 2467.95(2376.79,2554.92) | 2531.88(2439.65,2616.15) | 0.03 | 382.13(368.05,395.44) | 381.64(367.51,394.90) | 0.00(-0.01,0.00) |
| Morocco | both | 80658.78(77323.64,83877.17) | 128305.81(123326.69,133193.47) | 0.59 | 344.04(331.16,357.13) | 342.86(330.04,355.84) | -0.01(-0.02,0.00) |
| Mozambique | both | 40582.11(39049.40,42076.69) | 92021.78(88469.75,95488.58) | 1.27 | 368.85(354.81,382.27) | 367.65(353.64,380.89) | -0.01(-0.02,-0.01) |
| Myanmar | both | 182394.50(175603.74,189440.00) | 268693.14(259048.92,278526.96) | 0.47 | 476.31(459.55,493.43) | 479.23(462.40,496.56) | 0.02(0.02,0.02) |
| Namibia | both | 4543.57(4351.10,4722.97) | 8536.37(8177.26,8890.94) | 0.88 | 365.85(351.04,380.05) | 366.86(352.13,380.96) | 0.01(0.00,0.01) |
| Nauru | both | 40.52(38.91,42.12) | 45.04(43.22,46.83) | 0.11 | 440.05(423.69,456.49) | 445.72(429.31,462.23) | 0.05(0.04,0.06) |
| Nepal | both | 58527.61(56219.18,60768.93) | 105572.76(101464.80,109587.45) | 0.80 | 340.89(328.40,353.46) | 348.04(335.47,360.85) | 0.08(0.07,0.09) |
| Netherlands | both | 78005.38(74964.69,80701.74) | 85402.53(82515.70,88278.08) | 0.09 | 478.18(460.05,494.93) | 476.16(459.31,492.96) | -0.02(-0.02,-0.01) |
| New Zealand | both | 18178.36(17539.44,18835.56) | 23650.99(22849.62,24433.80) | 0.30 | 507.83(490.06,526.17) | 510.50(492.80,528.95) | 0.02(0.02,0.02) |
| Nicaragua | both | 12650.16(12167.90,13146.90) | 25263.55(24307.55,26201.68) | 1.00 | 386.65(372.43,400.06) | 385.11(370.91,398.44) | -0.01(-0.01,-0.01) |
| Niger | both | 23638.91(22664.62,24593.08) | 67019.83(64235.86,69668.92) | 1.84 | 362.76(348.06,376.79) | 364.45(349.84,378.56) | 0.02(0.02,0.03) |
| Nigeria | both | 283241.01(272382.40,294051.77) | 697074.30(670220.66,724116.14) | 1.46 | 358.59(345.37,371.30) | 369.99(356.22,383.29) | 0.11(0.10,0.13) |
| Niue | both | 9.42(9.09,9.76) | 7.45(7.19,7.71) | -0.21 | 443.03(426.71,459.56) | 443.26(426.91,459.81) | 0.00(-0.01,0.01) |
| North Macedonia | both | 7934.34(7636.77,8214.36) | 9014.06(8675.01,9323.13) | 0.14 | 381.15(367.01,394.42) | 378.80(364.79,391.90) | -0.03(-0.03,-0.02) |
| Northern Mariana Islands | both | 217.22(207.33,226.73) | 195.17(187.84,202.00) | -0.10 | 432.89(416.69,449.17) | 438.12(422.09,454.35) | 0.02(-0.02,0.06) |
| Norway | both | 21624.69(20885.36,22400.95) | 26826.35(25957.20,27750.64) | 0.24 | 479.39(462.11,496.85) | 476.44(459.16,493.85) | -0.02(-0.03,-0.02) |
| Oman | both | 5714.61(5468.48,5954.26) | 16723.77(15926.39,17493.91) | 1.93 | 317.24(305.53,328.78) | 311.63(300.09,322.94) | -0.10(-0.17,-0.02) |
| Pakistan | both | 321872.85(310157.12,334529.01) | 701807.14(675849.44,729814.17) | 1.18 | 334.65(322.67,346.96) | 338.52(326.23,351.17) | 0.04(0.04,0.05) |
| Palau | both | 68.78(65.98,71.54) | 83.89(80.79,86.79) | 0.22 | 440.32(423.89,456.76) | 428.06(412.27,443.85) | -0.08(-0.12,-0.05) |
| Palestine | both | 5858.93(5619.56,6092.49) | 16037.09(15371.68,16684.06) | 1.74 | 345.39(332.39,358.56) | 341.64(328.84,354.57) | -0.03(-0.03,-0.02) |
| Panama | both | 8732.97(8404.18,9057.96) | 15987.54(15395.63,16542.35) | 0.83 | 380.65(366.57,393.90) | 381.19(367.07,394.48) | 0.01(0.01,0.01) |
| Papua New Guinea | both | 16316.53(15661.10,16968.30) | 41419.57(39767.79,43063.60) | 1.54 | 439.42(422.97,455.90) | 440.25(424.01,456.83) | 0.00(0.00,0.00) |
| Paraguay | both | 13875.21(13369.40,14396.35) | 27019.77(26000.99,28021.61) | 0.95 | 381.75(367.65,395.02) | 380.73(366.61,393.95) | -0.01(-0.01,-0.01) |
| Peru | both | 76840.92(73959.51,79681.73) | 133423.18(128415.34,138158.02) | 0.74 | 383.61(369.50,396.99) | 382.66(368.50,396.06) | -0.01(-0.01,-0.01) |
| Philippines | both | 276186.98(265583.62,286350.86) | 529106.94(509766.36,548021.68) | 0.92 | 477.17(460.86,494.02) | 476.15(459.79,492.90) | -0.01(-0.01,0.00) |
| Poland | both | 153392.83(147472.28,159018.82) | 161384.51(155381.37,166935.07) | 0.05 | 386.11(371.70,399.68) | 384.27(369.97,397.74) | -0.02(-0.02,-0.02) |
| Portugal | both | 51158.86(49358.15,52914.47) | 54213.88(52332.52,56010.78) | 0.06 | 481.13(463.28,498.12) | 480.22(462.33,497.18) | -0.01(-0.01,0.00) |
| Puerto Rico | both | 14087.65(13567.86,14579.23) | 14353.32(13845.51,14804.18) | 0.02 | 387.19(372.94,400.65) | 386.20(372.02,399.61) | -0.01(-0.01,-0.01) |
| Qatar | both | 1507.71(1436.51,1577.90) | 10616.97(10113.46,11119.24) | 6.04 | 301.80(290.42,312.66) | 290.74(279.49,301.21) | -0.22(-0.27,-0.16) |
| Republic of Korea | both | 238072.98(228521.74,247612.50) | 298085.17(287509.99,308341.91) | 0.25 | 507.45(489.25,526.08) | 499.28(481.11,517.94) | -0.05(-0.06,-0.05) |
| Republic of Moldova | both | 17709.43(17042.47,18343.03) | 15752.17(15156.94,16304.31) | -0.11 | 388.14(373.92,401.60) | 384.09(369.91,397.42) | -0.04(-0.04,-0.04) |
| Romania | both | 92325.10(88982.72,95503.41) | 77913.33(75148.31,80458.27) | -0.16 | 383.08(368.93,396.38) | 381.39(367.26,394.69) | -0.02(-0.02,-0.01) |
| Russian Federation | both | 624685.37(600679.54,646723.21) | 614159.22(590789.30,635460.47) | -0.02 | 389.57(375.05,403.19) | 388.08(373.64,401.72) | -0.01(-0.01,-0.01) |
| Rwanda | both | 22140.00(21294.15,23014.23) | 43524.89(41822.47,45216.83) | 0.97 | 365.58(351.68,378.53) | 365.87(351.96,378.79) | 0.00(-0.02,0.02) |
| Saint Kitts and Nevis | both | 152.00(146.14,157.73) | 246.28(237.26,254.79) | 0.62 | 384.90(370.75,398.27) | 381.68(367.60,394.92) | -0.03(-0.04,-0.03) |
| Saint Lucia | both | 487.38(469.02,505.43) | 726.16(700.01,750.88) | 0.49 | 386.71(372.53,400.12) | 382.24(368.10,395.59) | -0.04(-0.04,-0.04) |
| Saint Vincent and the Grenadines | both | 388.41(373.57,403.14) | 448.49(432.44,463.71) | 0.15 | 382.97(368.83,396.29) | 379.54(365.46,392.77) | -0.03(-0.03,-0.02) |
| Samoa | both | 643.52(618.82,667.78) | 880.16(847.07,911.77) | 0.37 | 439.20(422.85,455.58) | 440.72(424.35,457.21) | 0.01(0.01,0.01) |
| San Marino | both | 119.15(114.87,123.35) | 165.46(159.76,170.98) | 0.39 | 475.83(458.19,492.73) | 478.40(460.57,495.40) | 0.02(0.02,0.02) |
| Sao Tome and Principe | both | 366.25(351.63,380.54) | 710.20(680.72,738.92) | 0.94 | 365.39(350.53,379.58) | 361.42(346.98,375.37) | -0.05(-0.05,-0.04) |
| Saudi Arabia | both | 47861.78(45860.14,49798.27) | 136397.31(130390.48,142108.18) | 1.85 | 323.29(311.33,335.24) | 324.00(312.03,336.00) | 0.01(-0.01,0.02) |
| Senegal | both | 23178.60(22226.08,24100.15) | 49983.10(47864.52,51961.77) | 1.16 | 365.69(350.82,379.88) | 363.97(349.23,378.13) | -0.02(-0.02,-0.02) |
| Serbia | both | 37789.36(36390.81,39062.50) | 35463.51(34185.98,36612.49) | -0.06 | 382.74(368.51,396.06) | 380.85(366.71,394.10) | -0.01(-0.02,-0.01) |
| Seychelles | both | 337.60(324.81,350.53) | 513.89(495.22,532.71) | 0.52 | 473.83(457.22,490.59) | 467.28(451.11,483.78) | -0.06(-0.07,-0.05) |
| Sierra Leone | both | 11693.17(11205.80,12171.72) | 27619.08(26419.29,28747.32) | 1.36 | 365.22(350.32,379.40) | 362.80(348.11,376.85) | -0.04(-0.06,-0.03) |
| Singapore | both | 17157.36(16445.03,17860.20) | 32459.86(31206.38,33716.53) | 0.89 | 503.76(485.71,522.32) | 496.01(478.06,514.22) | -0.07(-0.08,-0.06) |
| Slovakia | both | 21032.06(20241.59,21769.44) | 22757.12(21934.99,23529.16) | 0.08 | 383.99(369.79,397.35) | 381.69(367.55,394.95) | -0.02(-0.03,-0.02) |
| Slovenia | both | 8098.93(7800.16,8382.67) | 8336.08(8041.17,8603.64) | 0.03 | 383.64(369.52,396.95) | 379.07(365.02,392.19) | -0.05(-0.05,-0.05) |
| Solomon Islands | both | 1264.66(1215.69,1314.52) | 2691.19(2584.89,2795.28) | 1.13 | 438.60(422.14,455.22) | 443.10(426.84,459.72) | 0.04(0.04,0.04) |
| Somalia | both | 21404.95(20583.54,22219.40) | 62132.86(59761.50,64528.62) | 1.90 | 361.54(347.73,374.42) | 361.31(347.71,374.05) | -0.01(-0.01,-0.01) |
| South Africa | both | 129589.81(124454.77,134485.69) | 213784.94(205422.48,221888.87) | 0.65 | 368.83(355.17,382.09) | 366.16(352.60,379.31) | -0.03(-0.04,-0.02) |
| South Sudan | both | 17981.61(17284.73,18670.91) | 28705.45(27599.46,29779.38) | 0.60 | 352.35(339.00,364.62) | 364.07(350.08,377.40) | 0.12(0.10,0.13) |
| Spain | both | 196167.54(189148.02,203035.00) | 231951.07(223743.59,239697.80) | 0.18 | 479.40(461.60,496.36) | 477.20(459.38,494.08) | -0.02(-0.02,-0.02) |
| Sri Lanka | both | 82385.36(79270.34,85463.65) | 108716.80(104861.93,112536.60) | 0.32 | 480.23(463.72,497.07) | 477.83(461.03,495.04) | 0.02(0.00,0.03) |
| Sudan | both | 59654.14(57211.41,62044.25) | 131115.29(125566.40,136523.79) | 1.20 | 343.60(330.75,356.57) | 342.55(329.68,355.46) | 0.00(-0.01,0.00) |
| Suriname | both | 1418.59(1365.61,1471.12) | 2265.30(2183.19,2342.45) | 0.60 | 381.54(367.46,394.78) | 383.96(369.82,397.29) | 0.02(0.02,0.03) |
| Sweden | both | 43375.87(41939.02,44848.28) | 50479.33(48851.52,52207.98) | 0.16 | 479.22(461.93,496.69) | 477.08(459.82,494.49) | -0.02(-0.02,-0.02) |
| Switzerland | both | 36128.00(34760.96,37444.29) | 44608.44(43023.49,46108.57) | 0.23 | 478.72(460.98,495.61) | 475.72(457.82,492.56) | -0.02(-0.02,-0.02) |
| Syrian Arab Republic | both | 36822.83(35318.96,38288.36) | 49293.19(47413.88,51128.10) | 0.34 | 340.52(327.82,353.33) | 350.09(336.84,363.38) | 0.08(0.05,0.12) |
| Taiwan (Province of China) | both | 89973.37(86259.05,93245.76) | 110538.59(106666.35,114233.31) | 0.23 | 416.76(401.09,431.05) | 420.33(404.55,434.51) | 0.03(0.03,0.04) |
| Tajikistan | both | 18073.44(17368.14,18796.86) | 35769.00(34372.95,37130.96) | 0.98 | 383.89(369.79,397.28) | 381.84(367.69,395.13) | -0.02(-0.02,-0.02) |
| Thailand | both | 276955.04(266246.78,287992.75) | 367115.39(354000.92,380238.77) | 0.33 | 476.27(459.53,493.38) | 475.91(459.18,492.98) | -0.01(-0.01,0.00) |
| Timor-Leste | both | 3336.53(3206.79,3473.66) | 5787.22(5573.34,5998.76) | 0.73 | 471.82(455.25,488.67) | 474.22(457.45,491.18) | 0.01(0.01,0.02) |
| Togo | both | 11204.09(10724.71,11667.07) | 26911.03(25793.35,28018.04) | 1.40 | 368.42(353.42,382.70) | 368.30(353.42,382.59) | 0.01(0.00,0.01) |
| Tokelau | both | 6.53(6.30,6.78) | 5.89(5.69,6.10) | -0.10 | 448.47(432.07,465.23) | 440.65(424.49,457.08) | -0.08(-0.09,-0.06) |
| Tonga | both | 379.18(365.24,393.10) | 427.56(411.52,443.18) | 0.13 | 446.73(430.68,463.73) | 447.57(431.54,464.35) | 0.01(0.00,0.02) |
| Trinidad and Tobago | both | 4515.13(4342.99,4684.55) | 5703.67(5490.18,5907.47) | 0.26 | 382.25(368.12,395.55) | 380.97(366.87,394.25) | -0.01(-0.01,-0.01) |
| Tunisia | both | 26921.10(25824.74,27984.33) | 42432.34(40795.00,44040.07) | 0.58 | 342.21(329.38,355.19) | 344.19(331.23,357.26) | 0.02(0.02,0.03) |
| Turkey | both | 188150.78(180004.40,195665.97) | 296490.59(283984.19,308668.11) | 0.58 | 332.50(319.30,345.45) | 331.96(318.50,344.85) | 0.00(-0.01,0.00) |
| Turkmenistan | both | 12992.70(12484.00,13520.23) | 19560.64(18830.09,20269.15) | 0.51 | 386.04(371.89,399.43) | 379.14(365.30,392.31) | -0.07(-0.07,-0.07) |
| Tuvalu | both | 40.46(38.95,42.02) | 51.88(49.91,53.80) | 0.28 | 454.45(438.09,471.54) | 438.51(422.19,454.84) | -0.13(-0.14,-0.13) |
| Uganda | both | 51663.36(49651.22,53621.97) | 127647.55(122746.10,132519.20) | 1.47 | 363.27(349.46,376.28) | 366.29(352.31,379.41) | 0.03(0.02,0.03) |
| Ukraine | both | 216645.39(208496.18,224133.50) | 188071.42(180976.20,194512.92) | -0.13 | 390.23(375.66,403.84) | 388.40(373.94,402.02) | -0.01(-0.02,-0.01) |
| United Arab Emirates | both | 6211.53(5921.05,6497.26) | 34673.24(32933.13,36375.49) | 4.58 | 305.54(294.03,316.48) | 302.52(290.84,313.46) | -0.09(-0.12,-0.05) |
| United Kingdom | both | 293040.69(283606.10,303438.02) | 337845.31(326940.41,349142.33) | 0.15 | 480.37(463.63,497.64) | 478.33(461.50,495.54) | -0.02(-0.02,-0.01) |
| United Republic of Tanzania | both | 79202.82(76017.70,82269.49) | 183106.74(175744.71,190181.15) | 1.31 | 364.03(350.50,377.52) | 365.83(352.28,378.42) | 0.01(0.00,0.02) |
| United States of America | both | 1745911.11(1691489.59,1796500.92) | 2203662.30(2144283.10,2261351.45) | 0.26 | 644.91(626.03,663.34) | 627.64(610.14,644.64) | -0.29(-0.39,-0.18) |
| United States Virgin Islands | both | 409.38(394.48,424.18) | 413.70(398.93,426.48) | 0.01 | 387.81(373.50,401.38) | 387.95(373.67,401.42) | 0.00(0.00,0.01) |
| Uruguay | both | 16082.54(15488.61,16636.56) | 18255.29(17586.44,18858.08) | 0.14 | 508.74(489.57,526.96) | 509.53(490.30,527.76) | 0.01(0.01,0.01) |
| Uzbekistan | both | 72859.96(70013.52,75799.07) | 132196.38(127123.05,137130.56) | 0.81 | 385.12(370.97,398.55) | 384.06(369.87,397.42) | -0.01(-0.01,-0.01) |
| Vanuatu | both | 586.94(563.62,610.82) | 1231.05(1182.33,1279.00) | 1.10 | 440.33(424.04,457.01) | 443.37(427.07,460.07) | 0.02(0.02,0.03) |
| Venezuela (Bolivarian Republic of) | both | 68132.71(65551.49,70735.65) | 111983.59(107900.74,115887.81) | 0.64 | 383.74(369.62,397.14) | 385.20(370.99,398.60) | 0.00(-0.01,0.00) |
| Viet Nam | both | 302255.68(290536.68,313939.33) | 499423.18(480469.46,518269.42) | 0.65 | 480.22(463.31,497.62) | 475.37(458.75,492.35) | -0.04(-0.04,-0.03) |
| Yemen | both | 37546.86(36039.83,39069.44) | 99351.04(95209.32,103414.66) | 1.65 | 343.56(330.62,356.56) | 342.90(330.05,355.81) | 0.00(-0.01,0.00) |
| Zambia | both | 24018.80(23093.87,24916.24) | 59562.16(57209.93,61927.41) | 1.48 | 362.67(348.86,375.76) | 362.17(348.45,375.09) | -0.01(-0.01,0.00) |
| Zimbabwe | both | 31854.43(30531.92,33154.68) | 50955.59(48811.65,53027.32) | 0.60 | 365.56(350.70,379.79) | 369.44(354.54,383.68) | 0.04(0.04,0.04) |
| Afghanistan | male | 10833.85(10386.29,11263.81) | 40636.37(38775.07,42382.47) | 2.75 | 238.30(228.39,247.94) | 238.30(228.39,247.94) | 0.00(0.00,0.00) |
| Albania | male | 4351.57(4171.16,4526.65) | 3789.75(3639.93,3930.21) | -0.13 | 261.86(251.52,272.05) | 261.86(251.52,272.05) | 0.00(0.00,0.00) |
| Algeria | male | 27109.17(25920.81,28264.05) | 52443.83(50158.84,54682.62) | 0.93 | 238.30(228.39,247.94) | 238.30(228.39,247.94) | 0.00(0.00,0.00) |
| American Samoa | male | 72.76(69.56,75.99) | 85.28(81.83,88.58) | 0.17 | 316.80(303.94,329.45) | 316.80(303.94,329.45) | 0.00(0.00,0.00) |
| Andorra | male | 133.22(126.35,139.38) | 167.90(160.71,174.67) | 0.26 | 395.72(377.00,413.84) | 395.72(377.00,413.84) | 0.00(0.00,0.00) |
| Angola | male | 11310.04(10824.24,11819.64) | 31143.18(29800.19,32541.55) | 1.75 | 252.12(241.85,262.23) | 252.12(241.85,262.23) | 0.00(0.00,0.00) |
| Antigua and Barbuda | male | 75.61(72.47,78.65) | 122.53(117.68,127.23) | 0.62 | 261.86(251.52,272.05) | 261.86(251.52,272.05) | 0.00(0.00,0.00) |
| Argentina | male | 58709.62(56185.65,61327.76) | 83674.23(80070.52,87504.86) | 0.43 | 368.41(352.28,385.03) | 368.41(352.28,385.03) | 0.00(0.00,0.00) |
| Armenia | male | 4396.70(4208.42,4575.43) | 4087.59(3916.16,4245.18) | -0.07 | 261.86(251.52,272.05) | 261.86(251.52,272.05) | 0.00(0.00,0.00) |
| Australia | male | 32540.89(31123.48,34025.49) | 44086.71(42240.26,46038.28) | 0.35 | 368.41(352.28,385.03) | 368.41(352.28,385.03) | 0.00(0.00,0.00) |
| Austria | male | 15973.51(15214.96,16717.11) | 17169.31(16416.02,17887.82) | 0.07 | 395.72(377.00,413.84) | 395.72(377.00,413.84) | 0.00(0.00,0.00) |
| Azerbaijan | male | 9082.59(8695.63,9469.08) | 14626.50(14003.56,15217.57) | 0.61 | 261.86(251.52,272.05) | 261.86(251.52,272.05) | 0.00(0.00,0.00) |
| Bahamas | male | 330.73(316.53,344.33) | 513.14(492.71,533.36) | 0.55 | 261.86(251.52,272.05) | 261.86(251.52,272.05) | 0.00(0.00,0.00) |
| Bahrain | male | 792.07(753.77,835.68) | 2603.12(2482.82,2728.34) | 2.29 | 238.30(228.39,247.94) | 238.30(228.39,247.94) | 0.00(0.00,0.00) |
| Bangladesh | male | 115065.24(110164.15,119982.25) | 187885.46(180284.88,195832.03) | 0.63 | 235.00(225.60,244.58) | 235.00(225.60,244.58) | 0.00(0.00,0.00) |
| Barbados | male | 331.88(318.03,345.34) | 399.30(383.92,414.07) | 0.20 | 261.86(251.52,272.05) | 261.86(251.52,272.05) | 0.00(0.00,0.00) |
| Belarus | male | 13577.43(13016.63,14116.88) | 12646.26(12124.44,13135.21) | -0.07 | 261.86(251.52,272.05) | 261.86(251.52,272.05) | 0.00(0.00,0.00) |
| Belgium | male | 20290.15(19333.11,21186.97) | 21283.90(20376.92,22152.35) | 0.05 | 395.72(377.00,413.84) | 395.72(377.00,413.84) | 0.00(0.00,0.00) |
| Belize | male | 215.32(206.45,224.42) | 535.84(514.06,556.90) | 1.49 | 261.86(251.52,272.05) | 261.86(251.52,272.05) | 0.00(0.00,0.00) |
| Benin | male | 4667.34(4470.86,4876.48) | 13355.93(12773.77,13962.91) | 1.86 | 252.12(241.85,262.23) | 252.12(241.85,262.23) | 0.00(0.00,0.00) |
| Bermuda | male | 84.36(80.65,87.79) | 86.81(83.52,90.02) | 0.03 | 261.86(251.52,272.05) | 261.86(251.52,272.05) | 0.00(0.00,0.00) |
| Bhutan | male | 690.69(658.79,721.46) | 996.25(952.49,1043.33) | 0.44 | 235.00(225.60,244.58) | 235.00(225.60,244.58) | 0.00(0.00,0.00) |
| Bolivia (Plurinational State of) | male | 7289.81(6991.83,7593.52) | 15390.90(14760.28,16009.21) | 1.11 | 261.86(251.52,272.05) | 261.86(251.52,272.05) | 0.00(0.00,0.00) |
| Bosnia and Herzegovina | male | 6337.64(6076.20,6593.21) | 4568.96(4390.88,4737.62) | -0.28 | 261.86(251.52,272.05) | 261.86(251.52,272.05) | 0.00(0.00,0.00) |
| Botswana | male | 1344.08(1286.07,1403.95) | 3007.26(2875.91,3150.64) | 1.24 | 252.12(241.85,262.23) | 252.12(241.85,262.23) | 0.00(0.00,0.00) |
| Brazil | male | 185943.95(177452.29,193723.31) | 296872.46(284312.90,309205.46) | 0.60 | 263.23(252.42,274.06) | 263.23(252.42,274.06) | 0.00(0.00,0.00) |
| Brunei Darussalam | male | 562.22(533.59,590.11) | 981.20(934.66,1028.28) | 0.75 | 368.41(352.28,385.03) | 368.41(352.28,385.03) | 0.00(0.00,0.00) |
| Bulgaria | male | 11810.67(11351.89,12255.61) | 9533.80(9151.71,9881.95) | -0.19 | 261.86(251.52,272.05) | 261.86(251.52,272.05) | 0.00(0.00,0.00) |
| Burkina Faso | male | 9021.10(8650.80,9409.68) | 23260.43(22265.13,24302.66) | 1.58 | 252.12(241.85,262.23) | 252.12(241.85,262.23) | 0.00(0.00,0.00) |
| Burundi | male | 5801.82(5540.45,6054.22) | 13190.04(12595.02,13745.57) | 1.27 | 254.99(244.35,264.87) | 254.99(244.35,264.87) | 0.00(0.00,0.00) |
| Cabo Verde | male | 345.78(330.98,361.43) | 751.86(719.09,787.16) | 1.17 | 252.12(241.85,262.23) | 252.12(241.85,262.23) | 0.00(0.00,0.00) |
| Cambodia | male | 15511.29(14865.60,16171.98) | 31174.89(29929.97,32494.86) | 1.01 | 377.73(363.56,392.09) | 377.73(363.56,392.09) | 0.00(0.00,0.00) |
| Cameroon | male | 10684.23(10234.61,11159.92) | 33001.13(31554.33,34507.73) | 2.09 | 252.12(241.85,262.23) | 252.12(241.85,262.23) | 0.00(0.00,0.00) |
| Canada | male | 47674.07(45475.38,49856.85) | 56308.07(53892.08,58507.29) | 0.18 | 328.43(314.07,342.99) | 328.43(314.07,342.99) | 0.00(0.00,0.00) |
| Central African Republic | male | 2924.00(2797.94,3057.16) | 5780.80(5534.02,6038.03) | 0.98 | 252.12(241.85,262.23) | 252.12(241.85,262.23) | 0.00(0.00,0.00) |
| Chad | male | 5883.57(5638.85,6143.60) | 16192.06(15507.77,16922.64) | 1.75 | 252.12(241.85,262.23) | 252.12(241.85,262.23) | 0.00(0.00,0.00) |
| Chile | male | 25202.67(24034.14,26372.91) | 34296.07(32803.60,35862.75) | 0.36 | 368.41(352.28,385.03) | 368.41(352.28,385.03) | 0.00(0.00,0.00) |
| China | male | 1791873.62(1711724.41,1869863.92) | 2270456.09(2174473.48,2359634.03) | 0.27 | 283.18(271.53,294.58) | 283.18(271.53,294.58) | 0.00(0.00,0.00) |
| Colombia | male | 40023.59(38336.89,41663.33) | 63716.31(61138.13,66219.31) | 0.59 | 261.86(251.52,272.05) | 261.86(251.52,272.05) | 0.00(0.00,0.00) |
| Comoros | male | 492.62(471.38,512.82) | 895.47(856.71,931.16) | 0.82 | 254.99(244.35,264.87) | 254.99(244.35,264.87) | 0.00(0.00,0.00) |
| Congo | male | 2578.18(2466.08,2694.70) | 6211.63(5956.64,6483.38) | 1.41 | 252.12(241.85,262.23) | 252.12(241.85,262.23) | 0.00(0.00,0.00) |
| Cook Islands | male | 29.65(28.37,30.91) | 27.59(26.55,28.62) | -0.07 | 316.80(303.94,329.45) | 316.80(303.94,329.45) | 0.00(0.00,0.00) |
| Costa Rica | male | 3767.82(3608.74,3924.38) | 6330.30(6073.68,6578.46) | 0.68 | 261.86(251.52,272.05) | 261.86(251.52,272.05) | 0.00(0.00,0.00) |
| Côte d'Ivoire | male | 13574.91(12971.95,14191.30) | 31368.79(30005.68,32774.78) | 1.31 | 252.12(241.85,262.23) | 252.12(241.85,262.23) | 0.00(0.00,0.00) |
| Croatia | male | 6719.10(6445.72,6976.23) | 5810.22(5584.59,6031.61) | -0.14 | 261.86(251.52,272.05) | 261.86(251.52,272.05) | 0.00(0.00,0.00) |
| Cuba | male | 14989.96(14362.36,15574.63) | 15838.52(15219.52,16406.64) | 0.06 | 261.86(251.52,272.05) | 261.86(251.52,272.05) | 0.00(0.00,0.00) |
| Cyprus | male | 1618.97(1539.05,1692.92) | 2753.97(2625.01,2876.87) | 0.70 | 395.72(377.00,413.84) | 395.72(377.00,413.84) | 0.00(0.00,0.00) |
| Czechia | male | 13801.57(13247.63,14354.13) | 14742.65(14157.82,15296.50) | 0.07 | 261.86(251.52,272.05) | 261.86(251.52,272.05) | 0.00(0.00,0.00) |
| Democratic People's Republic of Korea | male | 27600.93(26373.76,28783.12) | 40771.91(38942.78,42504.27) | 0.48 | 281.76(269.74,293.05) | 281.76(269.74,293.05) | 0.00(0.00,0.00) |
| Democratic Republic of the Congo | male | 40010.09(38282.43,41816.37) | 98620.42(94327.02,103116.13) | 1.46 | 252.12(241.85,262.23) | 252.12(241.85,262.23) | 0.00(0.00,0.00) |
| Denmark | male | 10531.96(10045.94,10992.80) | 10964.73(10493.69,11422.47) | 0.04 | 395.72(377.00,413.84) | 395.72(377.00,413.84) | 0.00(0.00,0.00) |
| Djibouti | male | 588.50(560.91,614.43) | 1594.70(1524.14,1659.90) | 1.71 | 254.99(244.35,264.87) | 254.99(244.35,264.87) | 0.00(0.00,0.00) |
| Dominica | male | 91.34(87.72,94.97) | 96.25(92.56,99.83) | 0.05 | 261.86(251.52,272.05) | 261.86(251.52,272.05) | 0.00(0.00,0.00) |
| Dominican Republic | male | 8507.27(8151.94,8860.45) | 14635.56(14037.31,15221.88) | 0.72 | 261.86(251.52,272.05) | 261.86(251.52,272.05) | 0.00(0.00,0.00) |
| Ecuador | male | 11946.27(11449.80,12438.12) | 23031.37(22106.25,23951.69) | 0.93 | 261.86(251.52,272.05) | 261.86(251.52,272.05) | 0.00(0.00,0.00) |
| Egypt | male | 62126.12(59267.31,64927.09) | 121517.12(115948.10,127326.28) | 0.96 | 238.62(228.39,248.99) | 239.18(228.74,249.98) | 0.00(0.00,0.01) |
| El Salvador | male | 5927.01(5692.74,6169.40) | 7595.86(7289.78,7890.63) | 0.28 | 261.86(251.52,272.05) | 261.86(251.52,272.05) | 0.00(0.00,0.00) |
| Equatorial Guinea | male | 412.16(395.18,429.94) | 1786.79(1701.89,1872.29) | 3.34 | 252.12(241.85,262.23) | 252.12(241.85,262.23) | 0.00(0.00,0.00) |
| Eritrea | male | 3233.37(3088.49,3370.69) | 8054.92(7681.12,8397.57) | 1.49 | 254.99(244.35,264.87) | 254.99(244.35,264.87) | 0.00(0.00,0.00) |
| Estonia | male | 2021.90(1940.34,2100.26) | 1751.20(1681.18,1818.14) | -0.13 | 261.86(251.52,272.05) | 261.86(251.52,272.05) | 0.00(0.00,0.00) |
| Eswatini | male | 778.94(745.97,813.97) | 1341.02(1281.13,1402.69) | 0.72 | 252.12(241.85,262.23) | 252.12(241.85,262.23) | 0.00(0.00,0.00) |
| Ethiopia | male | 54185.08(51953.41,56457.71) | 123042.56(117732.28,128444.17) | 1.27 | 256.36(245.72,266.88) | 256.36(245.72,266.88) | 0.00(0.00,0.00) |
| Fiji | male | 1166.78(1113.95,1219.56) | 1487.73(1425.76,1548.05) | 0.28 | 316.80(303.94,329.45) | 316.80(303.94,329.45) | 0.00(0.00,0.00) |
| Finland | male | 10209.48(9742.69,10654.28) | 10286.38(9844.82,10708.13) | 0.01 | 395.72(377.00,413.84) | 395.72(377.00,413.84) | 0.00(0.00,0.00) |
| France | male | 115653.05(110275.10,120826.17) | 118687.75(113746.69,123336.10) | 0.03 | 395.72(377.00,413.84) | 395.72(377.00,413.84) | 0.00(0.00,0.00) |
| Gabon | male | 1105.99(1059.12,1156.78) | 2082.47(1994.50,2174.46) | 0.88 | 252.12(241.85,262.23) | 252.12(241.85,262.23) | 0.00(0.00,0.00) |
| Gambia | male | 1049.63(1004.49,1096.87) | 2498.04(2388.57,2612.02) | 1.38 | 252.12(241.85,262.23) | 252.12(241.85,262.23) | 0.00(0.00,0.00) |
| Georgia | male | 7067.23(6782.12,7342.97) | 4827.43(4634.43,5006.58) | -0.32 | 261.86(251.52,272.05) | 261.86(251.52,272.05) | -0.28(-1.34,0.79) |
| Germany | male | 164916.14(157126.31,172477.76) | 160838.67(153872.97,167485.29) | -0.02 | 395.72(377.00,413.84) | 395.72(377.00,413.84) | 0.00(0.00,0.00) |
| Ghana | male | 16016.09(15335.84,16734.57) | 36635.78(35049.96,38294.22) | 1.29 | 252.12(241.85,262.23) | 252.12(241.85,262.23) | 0.00(0.00,0.00) |
| Greece | male | 20434.99(19518.35,21325.67) | 18568.40(17806.46,19307.09) | -0.09 | 395.72(377.00,413.84) | 395.72(377.00,413.84) | 0.00(0.00,0.00) |
| Greenland | male | 117.47(111.64,123.30) | 98.01(93.72,102.12) | -0.17 | 328.43(314.07,342.99) | 328.43(314.07,342.99) | 0.00(0.00,0.00) |
| Grenada | male | 100.77(96.73,104.91) | 147.33(141.46,152.83) | 0.46 | 261.86(251.52,272.05) | 261.86(251.52,272.05) | 0.00(0.00,0.00) |
| Guam | male | 242.09(230.68,253.38) | 280.54(269.30,291.43) | 0.16 | 316.80(303.94,329.45) | 316.80(303.94,329.45) | 0.00(0.00,0.00) |
| Guatemala | male | 8678.87(8331.90,9034.94) | 21970.87(21036.12,22885.77) | 1.53 | 261.86(251.52,272.05) | 261.86(251.52,272.05) | 0.00(0.00,0.00) |
| Guinea | male | 6251.24(5996.84,6520.36) | 12935.50(12389.21,13506.85) | 1.07 | 252.12(241.85,262.23) | 252.12(241.85,262.23) | 0.00(0.00,0.00) |
| Guinea-Bissau | male | 1005.80(963.01,1051.22) | 2086.61(1993.64,2183.69) | 1.07 | 252.12(241.85,262.23) | 252.12(241.85,262.23) | 0.00(0.00,0.00) |
| Guyana | male | 944.42(904.00,983.90) | 1018.86(978.15,1059.42) | 0.08 | 261.86(251.52,272.05) | 261.86(251.52,272.05) | 0.00(0.00,0.00) |
| Haiti | male | 7111.94(6824.01,7401.79) | 15201.99(14565.48,15832.36) | 1.14 | 261.86(251.52,272.05) | 261.86(251.52,272.05) | 0.00(0.00,0.00) |
| Honduras | male | 5077.33(4874.57,5284.89) | 11935.44(11438.10,12411.76) | 1.35 | 261.86(251.52,272.05) | 261.86(251.52,272.05) | 0.00(0.00,0.00) |
| Hungary | male | 13844.15(13301.16,14384.03) | 13104.91(12590.25,13593.01) | -0.05 | 261.86(251.52,272.05) | 261.86(251.52,272.05) | 0.00(0.00,0.00) |
| Iceland | male | 529.62(503.31,554.08) | 692.83(661.65,722.64) | 0.31 | 395.72(377.00,413.84) | 395.72(377.00,413.84) | 0.00(0.00,0.00) |
| India | male | 980206.64(938801.75,1020945.57) | 1741332.91(1668497.90,1814962.32) | 0.78 | 236.24(226.85,245.95) | 236.24(226.85,245.95) | 0.00(0.00,0.00) |
| Indonesia | male | 331638.30(318346.57,345054.76) | 523977.24(502993.01,545071.86) | 0.58 | 379.73(365.31,394.52) | 379.73(365.31,394.52) | 0.00(0.00,0.00) |
| Iran (Islamic Republic of) | male | 62847.97(60282.99,65422.47) | 111586.30(106951.07,116404.38) | 0.78 | 239.53(230.12,248.90) | 239.53(230.12,248.90) | 0.00(0.00,0.00) |
| Iraq | male | 20419.04(19554.80,21334.58) | 57487.25(54815.77,60176.49) | 1.82 | 266.74(255.50,277.78) | 266.74(255.50,277.78) | 0.00(0.00,0.00) |
| Ireland | male | 7057.58(6738.84,7368.34) | 9295.93(8901.23,9665.35) | 0.32 | 395.72(377.00,413.84) | 395.72(377.00,413.84) | 0.00(0.00,0.00) |
| Israel | male | 9543.44(9093.29,9966.94) | 17726.67(16938.10,18493.88) | 0.86 | 395.72(377.00,413.84) | 395.72(377.00,413.84) | 0.00(0.00,0.00) |
| Italy | male | 114503.11(109674.06,119556.86) | 108328.91(104141.87,112737.61) | -0.05 | 397.29(380.79,414.68) | 397.29(380.79,414.68) | 0.00(0.00,0.00) |
| Jamaica | male | 2838.94(2724.00,2951.94) | 3878.91(3721.25,4027.52) | 0.37 | 261.86(251.52,272.05) | 261.86(251.52,272.05) | 0.00(0.00,0.00) |
| Japan | male | 235173.90(225172.01,244697.80) | 207645.21(199509.89,215338.31) | -0.12 | 370.17(353.50,385.64) | 370.17(353.50,385.64) | 0.00(0.00,0.00) |
| Jordan | male | 4136.04(3946.75,4320.68) | 15086.24(14416.52,15722.97) | 2.65 | 238.30(228.39,247.94) | 238.30(228.39,247.94) | 0.00(0.00,0.00) |
| Kazakhstan | male | 20723.73(19862.36,21585.52) | 24073.84(23073.79,25030.10) | 0.16 | 261.86(251.52,272.05) | 261.86(251.52,272.05) | 0.00(0.00,0.00) |
| Kenya | male | 24396.01(23363.95,25468.29) | 60105.29(57558.42,62740.02) | 1.46 | 256.36(245.72,266.88) | 256.36(245.72,266.88) | 0.00(0.00,0.00) |
| Kiribati | male | 106.51(101.70,111.31) | 176.51(168.60,184.37) | 0.66 | 316.80(303.94,329.45) | 316.80(303.94,329.45) | 0.00(0.00,0.00) |
| Kuwait | male | 2670.67(2544.29,2807.83) | 6677.88(6367.99,6980.75) | 1.50 | 238.30(228.39,247.94) | 238.30(228.39,247.94) | 0.00(0.00,0.00) |
| Kyrgyzstan | male | 5327.38(5106.42,5548.96) | 8442.61(8089.18,8784.33) | 0.58 | 261.86(251.52,272.05) | 261.86(251.52,272.05) | 0.00(0.00,0.00) |
| Lao People's Democratic Republic | male | 6604.16(6343.74,6877.38) | 13698.35(13148.49,14272.98) | 1.07 | 377.73(363.56,392.09) | 377.73(363.56,392.09) | 0.00(0.00,0.00) |
| Latvia | male | 3411.45(3274.34,3539.98) | 2480.51(2382.41,2575.03) | -0.27 | 261.86(251.52,272.05) | 261.86(251.52,272.05) | 0.00(0.00,0.00) |
| Lebanon | male | 3546.88(3396.49,3687.35) | 6272.83(6005.37,6542.47) | 0.77 | 238.30(228.39,247.94) | 238.30(228.39,247.94) | 0.00(0.00,0.00) |
| Lesotho | male | 1949.23(1868.53,2035.44) | 2649.05(2531.46,2773.83) | 0.36 | 252.12(241.85,262.23) | 252.12(241.85,262.23) | 0.00(0.00,0.00) |
| Liberia | male | 2016.23(1938.28,2099.33) | 5630.97(5390.09,5879.02) | 1.79 | 252.12(241.85,262.23) | 252.12(241.85,262.23) | 0.00(0.00,0.00) |
| Libya | male | 4725.86(4520.50,4924.73) | 9201.40(8791.99,9612.01) | 0.95 | 238.30(228.39,247.94) | 238.30(228.39,247.94) | 0.00(0.00,0.00) |
| Lithuania | male | 4772.78(4579.77,4954.15) | 3626.42(3484.77,3760.92) | -0.24 | 261.86(251.52,272.05) | 261.86(251.52,272.05) | 0.00(0.00,0.00) |
| Luxembourg | male | 802.36(763.85,838.68) | 1261.55(1204.66,1315.19) | 0.57 | 395.72(377.00,413.84) | 395.72(377.00,413.84) | 0.00(0.00,0.00) |
| Madagascar | male | 12922.90(12353.65,13472.32) | 30921.18(29533.68,32213.19) | 1.39 | 254.99(244.35,264.87) | 254.99(244.35,264.87) | 0.00(0.00,0.00) |
| Malawi | male | 10106.42(9657.52,10535.88) | 20228.33(19317.81,21077.48) | 1.00 | 254.99(244.35,264.87) | 254.99(244.35,264.87) | 0.00(0.00,0.00) |
| Malaysia | male | 31903.62(30592.75,33209.42) | 65780.79(63167.61,68483.35) | 1.06 | 377.73(363.56,392.09) | 377.73(363.56,392.09) | 0.00(0.00,0.00) |
| Maldives | male | 358.33(344.20,373.08) | 1385.27(1318.98,1453.61) | 2.87 | 377.73(363.56,392.09) | 377.73(363.56,392.09) | 0.00(0.00,0.00) |
| Mali | male | 8715.35(8356.45,9091.35) | 22547.95(21592.73,23541.75) | 1.59 | 252.12(241.85,262.23) | 252.12(241.85,262.23) | 0.00(0.00,0.00) |
| Malta | male | 750.90(716.73,783.55) | 852.11(814.63,887.84) | 0.13 | 395.72(377.00,413.84) | 395.72(377.00,413.84) | 0.00(0.00,0.00) |
| Marshall Islands | male | 62.31(59.54,65.08) | 92.44(88.32,96.50) | 0.48 | 316.80(303.94,329.45) | 316.80(303.94,329.45) | 0.00(0.00,0.00) |
| Mauritania | male | 2179.00(2088.22,2275.67) | 4457.36(4272.36,4656.13) | 1.05 | 252.12(241.85,262.23) | 252.12(241.85,262.23) | 0.00(0.00,0.00) |
| Mauritius | male | 2152.42(2062.73,2242.41) | 2606.30(2507.02,2702.57) | 0.21 | 377.73(363.56,392.09) | 377.73(363.56,392.09) | 0.00(0.00,0.00) |
| Mexico | male | 100493.33(95901.23,104719.38) | 165086.10(158243.01,171967.60) | 0.64 | 263.23(252.42,274.06) | 263.23(252.42,274.06) | 0.00(0.00,0.00) |
| Micronesia (Federated States of) | male | 146.56(140.18,153.01) | 163.83(156.70,170.71) | 0.12 | 316.80(303.94,329.45) | 316.80(303.94,329.45) | 0.00(0.00,0.00) |
| Monaco | male | 56.18(53.71,58.57) | 63.42(60.92,65.90) | 0.13 | 395.72(377.00,413.84) | 395.72(377.00,413.84) | 0.00(0.00,0.00) |
| Mongolia | male | 2523.35(2417.44,2631.58) | 4526.88(4326.88,4710.22) | 0.79 | 261.86(251.52,272.05) | 261.86(251.52,272.05) | 0.00(0.00,0.00) |
| Montenegro | male | 846.27(811.52,880.11) | 858.42(825.17,890.61) | 0.01 | 261.86(251.52,272.05) | 261.86(251.52,272.05) | 0.00(0.00,0.00) |
| Morocco | male | 27755.07(26553.05,28924.42) | 44625.03(42686.99,46466.32) | 0.61 | 238.30(228.39,247.94) | 238.30(228.39,247.94) | 0.00(0.00,0.00) |
| Mozambique | male | 13077.17(12513.92,13593.74) | 30307.13(28974.30,31606.20) | 1.32 | 254.99(244.35,264.87) | 254.99(244.35,264.87) | 0.00(0.00,0.00) |
| Myanmar | male | 71415.22(68495.22,74315.14) | 100911.48(97094.17,104906.33) | 0.41 | 377.73(363.56,392.09) | 377.73(363.56,392.09) | 0.00(0.00,0.00) |
| Namibia | male | 1519.32(1454.37,1586.83) | 2836.28(2713.34,2965.38) | 0.87 | 252.12(241.85,262.23) | 252.12(241.85,262.23) | 0.00(0.00,0.00) |
| Nauru | male | 14.96(14.30,15.63) | 16.11(15.36,16.85) | 0.08 | 316.80(303.94,329.45) | 316.80(303.94,329.45) | 0.00(0.00,0.00) |
| Nepal | male | 20162.06(19327.74,20959.53) | 33317.35(31960.36,34652.06) | 0.65 | 237.76(228.02,247.20) | 237.76(228.02,247.20) | 0.00(0.00,0.00) |
| Netherlands | male | 31754.04(30369.48,33165.68) | 32008.23(30832.27,33255.79) | 0.01 | 395.50(378.28,412.17) | 395.22(379.53,411.74) | 0.00(0.00,0.00) |
| New Zealand | male | 6520.02(6219.34,6795.93) | 7610.30(7294.51,7906.10) | 0.17 | 370.17(353.50,385.64) | 370.17(353.50,385.64) | 0.00(0.00,0.00) |
| Nicaragua | male | 4145.88(3976.70,4322.02) | 8440.71(8087.94,8785.46) | 1.04 | 261.86(251.52,272.05) | 261.86(251.52,272.05) | 0.00(0.00,0.00) |
| Niger | male | 8030.34(7694.55,8382.75) | 22818.11(21855.26,23867.28) | 1.84 | 252.12(241.85,262.23) | 252.12(241.85,262.23) | 0.00(0.00,0.00) |
| Nigeria | male | 101828.29(97822.36,105910.93) | 226120.16(217162.42,235331.63) | 1.22 | 253.47(243.84,263.27) | 253.47(243.84,263.27) | 0.00(0.00,0.00) |
| Niue | male | 3.40(3.26,3.53) | 2.67(2.57,2.77) | -0.21 | 316.80(303.94,329.45) | 316.80(303.94,329.45) | 0.00(0.00,0.00) |
| North Macedonia | male | 2746.19(2634.63,2856.60) | 3178.57(3050.13,3302.31) | 0.16 | 261.86(251.52,272.05) | 261.86(251.52,272.05) | 0.00(0.00,0.00) |
| Northern Mariana Islands | male | 83.99(79.94,87.96) | 72.91(70.01,75.70) | -0.13 | 316.80(303.94,329.45) | 316.80(303.94,329.45) | 0.00(0.00,0.00) |
| Norway | male | 8665.69(8287.99,9054.26) | 10574.23(10142.86,11025.68) | 0.22 | 397.29(380.79,414.68) | 397.29(380.79,414.68) | 0.00(0.00,0.00) |
| Oman | male | 2712.29(2585.91,2844.29) | 8954.15(8475.55,9476.30) | 2.30 | 238.30(228.39,247.94) | 238.30(228.39,247.94) | 0.00(0.00,0.00) |
| Pakistan | male | 119381.29(114544.79,124289.15) | 249373.71(238919.42,259565.69) | 1.09 | 236.24(226.85,245.95) | 236.24(226.85,245.95) | 0.00(0.00,0.00) |
| Palau | male | 25.60(24.42,26.79) | 34.71(33.30,36.11) | 0.36 | 316.80(303.94,329.45) | 316.80(303.94,329.45) | 0.00(0.00,0.00) |
| Palestine | male | 2042.07(1950.83,2131.21) | 5712.25(5457.64,5951.54) | 1.80 | 238.30(228.39,247.94) | 238.30(228.39,247.94) | 0.00(0.00,0.00) |
| Panama | male | 3031.87(2905.85,3152.01) | 5525.75(5310.18,5740.04) | 0.82 | 261.86(251.52,272.05) | 261.86(251.52,272.05) | 0.00(0.00,0.00) |
| Papua New Guinea | male | 6089.54(5822.12,6354.37) | 15274.77(14603.95,15953.94) | 1.51 | 316.80(303.94,329.45) | 316.80(303.94,329.45) | 0.00(0.00,0.00) |
| Paraguay | male | 4782.50(4581.91,4980.57) | 9412.08(9021.32,9792.67) | 0.97 | 261.86(251.52,272.05) | 261.86(251.52,272.05) | 0.00(0.00,0.00) |
| Peru | male | 25896.25(24834.34,26950.19) | 45376.86(43555.86,47158.52) | 0.75 | 261.86(251.52,272.05) | 261.86(251.52,272.05) | 0.00(0.00,0.00) |
| Philippines | male | 110179.28(105729.34,114681.44) | 214112.04(205633.98,222656.07) | 0.94 | 379.73(365.31,394.52) | 379.73(365.31,394.52) | 0.00(0.00,0.00) |
| Poland | male | 51069.64(48914.92,53224.96) | 53977.01(51809.72,56097.28) | 0.06 | 263.23(252.42,274.06) | 263.23(252.42,274.06) | 0.00(0.00,0.00) |
| Portugal | male | 19693.21(18801.26,20554.59) | 18956.56(18174.58,19702.98) | -0.04 | 395.72(377.00,413.84) | 395.72(377.00,413.84) | 0.00(0.00,0.00) |
| Puerto Rico | male | 4558.93(4380.86,4738.53) | 4607.08(4432.54,4773.96) | 0.01 | 261.86(251.52,272.05) | 261.86(251.52,272.05) | 0.00(0.00,0.00) |
| Qatar | male | 866.99(823.71,915.68) | 6928.49(6560.38,7330.88) | 6.99 | 238.30(228.39,247.94) | 238.30(228.39,247.94) | 0.00(0.00,0.00) |
| Republic of Korea | male | 92810.18(88340.59,97280.19) | 102454.43(98209.61,106848.42) | 0.10 | 368.41(352.28,385.03) | 368.41(352.28,385.03) | 0.00(0.00,0.00) |
| Republic of Moldova | male | 5664.29(5430.39,5896.34) | 5157.00(4938.93,5360.27) | -0.09 | 261.86(251.52,272.05) | 261.86(251.52,272.05) | 0.00(0.00,0.00) |
| Romania | male | 31090.46(29884.19,32273.80) | 26204.59(25186.15,27168.44) | -0.16 | 261.86(251.52,272.05) | 261.86(251.52,272.05) | 0.00(0.00,0.00) |
| Russian Federation | male | 199138.19(190714.46,207727.16) | 194833.75(186962.41,202882.93) | -0.02 | 263.23(252.42,274.06) | 263.23(252.42,274.06) | 0.00(0.00,0.00) |
| Rwanda | male | 7531.35(7187.77,7853.64) | 14766.58(14097.62,15392.80) | 0.96 | 254.99(244.35,264.87) | 254.99(244.35,264.87) | 0.00(0.00,0.00) |
| Saint Kitts and Nevis | male | 50.64(48.54,52.73) | 84.97(81.57,88.20) | 0.68 | 261.86(251.52,272.05) | 261.86(251.52,272.05) | 0.00(0.00,0.00) |
| Saint Lucia | male | 159.99(153.46,166.72) | 247.66(237.84,257.05) | 0.55 | 261.86(251.52,272.05) | 261.86(251.52,272.05) | 0.00(0.00,0.00) |
| Saint Vincent and the Grenadines | male | 132.94(127.45,138.55) | 158.24(152.22,164.18) | 0.19 | 261.86(251.52,272.05) | 261.86(251.52,272.05) | 0.00(0.00,0.00) |
| Samoa | male | 244.63(233.82,255.41) | 325.63(311.85,338.97) | 0.33 | 316.80(303.94,329.45) | 316.80(303.94,329.45) | 0.00(0.00,0.00) |
| San Marino | male | 48.07(45.82,50.26) | 59.57(57.08,61.97) | 0.24 | 395.72(377.00,413.84) | 395.72(377.00,413.84) | 0.00(0.00,0.00) |
| Sao Tome and Principe | male | 124.11(118.91,129.52) | 249.27(238.76,260.44) | 1.01 | 252.12(241.85,262.23) | 252.12(241.85,262.23) | 0.00(0.00,0.00) |
| Saudi Arabia | male | 20589.04(19654.53,21494.22) | 59818.08(57005.46,62692.63) | 1.91 | 238.30(228.39,247.94) | 238.30(228.39,247.94) | 0.00(0.00,0.00) |
| Senegal | male | 7677.32(7350.75,8020.82) | 17146.48(16414.41,17915.83) | 1.23 | 252.12(241.85,262.23) | 252.12(241.85,262.23) | 0.00(0.00,0.00) |
| Serbia | male | 12805.31(12299.23,13292.06) | 12074.11(11599.44,12522.24) | -0.06 | 261.86(251.52,272.05) | 261.86(251.52,272.05) | 0.00(0.00,0.00) |
| Seychelles | male | 135.39(129.79,140.98) | 223.86(214.98,232.59) | 0.65 | 377.73(363.56,392.09) | 377.73(363.56,392.09) | 0.00(0.00,0.00) |
| Sierra Leone | male | 3875.58(3720.14,4045.99) | 9426.48(9010.83,9854.27) | 1.43 | 252.12(241.85,262.23) | 252.12(241.85,262.23) | 0.00(0.00,0.00) |
| Singapore | male | 6664.19(6337.86,6993.47) | 11552.76(11022.87,12078.18) | 0.73 | 368.41(352.28,385.03) | 368.41(352.28,385.03) | 0.00(0.00,0.00) |
| Slovakia | male | 7023.50(6737.39,7304.60) | 7687.59(7368.25,7981.63) | 0.09 | 261.86(251.52,272.05) | 261.86(251.52,272.05) | 0.00(0.00,0.00) |
| Slovenia | male | 2703.47(2590.79,2811.50) | 2895.82(2782.54,3001.88) | 0.07 | 261.86(251.52,272.05) | 261.86(251.52,272.05) | 0.00(0.00,0.00) |
| Solomon Islands | male | 469.41(449.22,489.56) | 969.05(927.11,1011.62) | 1.06 | 316.80(303.94,329.45) | 316.80(303.94,329.45) | 0.00(0.00,0.00) |
| Somalia | male | 7700.87(7361.42,8022.35) | 22590.28(21525.24,23567.11) | 1.93 | 254.99(244.35,264.87) | 254.99(244.35,264.87) | 0.00(0.00,0.00) |
| South Africa | male | 42912.68(41135.28,44704.61) | 72873.36(69782.34,75945.10) | 0.70 | 253.47(243.84,263.27) | 253.47(243.84,263.27) | 0.00(0.00,0.00) |
| South Sudan | male | 6926.94(6620.44,7218.71) | 9831.80(9408.80,10236.59) | 0.42 | 254.99(244.35,264.87) | 254.99(244.35,264.87) | 0.00(0.00,0.00) |
| Spain | male | 77589.96(74014.12,81065.92) | 84977.27(81433.69,88349.12) | 0.10 | 395.72(377.00,413.84) | 395.72(377.00,413.84) | 0.00(0.00,0.00) |
| Sri Lanka | male | 33548.77(32162.22,34872.93) | 41285.62(39735.76,42808.75) | 0.23 | 390.00(374.68,404.51) | 377.73(363.56,392.09) | -0.03(-0.05,0.00) |
| Sudan | male | 20535.71(19652.49,21375.66) | 45484.97(43472.50,47389.95) | 1.21 | 238.30(228.39,247.94) | 238.30(228.39,247.94) | 0.00(0.00,0.00) |
| Suriname | male | 493.19(473.01,513.57) | 759.76(730.79,788.36) | 0.54 | 261.86(251.52,272.05) | 261.86(251.52,272.05) | 0.00(0.00,0.00) |
| Sweden | male | 16943.52(16246.67,17673.36) | 19561.84(18763.54,20378.44) | 0.15 | 397.29(380.79,414.68) | 397.29(380.79,414.68) | 0.00(0.00,0.00) |
| Switzerland | male | 14517.94(13832.47,15169.51) | 17031.38(16275.79,17746.14) | 0.17 | 395.72(377.00,413.84) | 395.72(377.00,413.84) | 0.00(0.00,0.00) |
| Syrian Arab Republic | male | 13193.02(12614.12,13751.13) | 15958.83(15302.27,16564.64) | 0.21 | 238.30(228.39,247.94) | 238.30(228.39,247.94) | 0.00(0.00,0.00) |
| Taiwan (Province of China) | male | 32131.64(30682.35,33550.57) | 37470.65(35991.89,38982.09) | 0.17 | 288.45(276.49,300.19) | 288.45(276.49,300.19) | 0.00(0.00,0.00) |
| Tajikistan | male | 6117.44(5864.06,6386.63) | 12363.68(11845.19,12877.35) | 1.02 | 261.86(251.52,272.05) | 261.86(251.52,272.05) | 0.00(0.00,0.00) |
| Thailand | male | 108404.03(103962.24,112871.82) | 141989.96(136408.48,147167.81) | 0.31 | 377.73(363.56,392.09) | 377.73(363.56,392.09) | 0.00(0.00,0.00) |
| Timor-Leste | male | 1372.43(1314.95,1430.41) | 2309.07(2217.97,2400.44) | 0.68 | 377.73(363.56,392.09) | 377.73(363.56,392.09) | 0.00(0.00,0.00) |
| Togo | male | 3654.03(3496.05,3822.32) | 8841.37(8468.00,9234.31) | 1.42 | 252.12(241.85,262.23) | 252.12(241.85,262.23) | 0.00(0.00,0.00) |
| Tokelau | male | 2.25(2.15,2.34) | 2.15(2.06,2.23) | -0.04 | 316.80(303.94,329.45) | 316.80(303.94,329.45) | 0.00(0.00,0.00) |
| Tonga | male | 133.79(128.08,139.37) | 148.15(141.96,154.08) | 0.11 | 316.80(303.94,329.45) | 316.80(303.94,329.45) | 0.00(0.00,0.00) |
| Trinidad and Tobago | male | 1548.09(1483.45,1610.16) | 1971.76(1891.19,2047.55) | 0.27 | 261.86(251.52,272.05) | 261.86(251.52,272.05) | 0.00(0.00,0.00) |
| Tunisia | male | 9462.52(9056.32,9854.15) | 14470.50(13875.06,15068.67) | 0.53 | 238.30(228.39,247.94) | 238.30(228.39,247.94) | 0.00(0.00,0.00) |
| Turkey | male | 63085.73(60205.94,66059.89) | 99435.08(94776.17,103744.99) | 0.58 | 219.40(209.98,228.76) | 219.75(209.99,229.09) | 0.00(0.00,0.00) |
| Turkmenistan | male | 4336.25(4154.17,4524.37) | 6983.04(6690.62,7269.31) | 0.61 | 261.86(251.52,272.05) | 261.86(251.52,272.05) | 0.00(0.00,0.00) |
| Tuvalu | male | 13.12(12.56,13.67) | 19.59(18.75,20.41) | 0.49 | 316.80(303.94,329.45) | 316.80(303.94,329.45) | 0.00(0.00,0.00) |
| Uganda | male | 17728.87(16941.56,18476.72) | 43192.59(41250.16,45039.82) | 1.44 | 254.99(244.35,264.87) | 254.99(244.35,264.87) | 0.00(0.00,0.00) |
| Ukraine | male | 67772.73(65004.11,70564.92) | 59020.97(56633.64,61431.23) | -0.13 | 263.23(252.42,274.06) | 263.23(252.42,274.06) | 0.00(0.00,0.00) |
| United Arab Emirates | male | 3415.18(3249.20,3601.75) | 21435.88(20260.63,22626.04) | 5.28 | 238.30(228.39,247.94) | 238.30(228.39,247.94) | 0.00(0.00,0.00) |
| United Kingdom | male | 113484.10(108857.13,118203.65) | 128978.95(123988.77,134224.21) | 0.14 | 397.03(380.99,413.51) | 397.05(380.94,413.56) | 0.00(0.00,0.00) |
| United Republic of Tanzania | male | 26941.83(25804.96,28097.63) | 61563.53(58952.16,64232.39) | 1.29 | 258.12(247.79,268.74) | 256.12(246.02,266.41) | -0.02(-0.02,-0.01) |
| United States of America | male | 489024.73(470817.17,508496.26) | 552015.45(535664.78,568114.76) | 0.13 | 370.18(356.90,384.12) | 351.26(340.63,362.05) | -0.25(-0.32,-0.17) |
| United States Virgin Islands | male | 131.87(126.69,137.00) | 131.02(126.13,135.62) | -0.01 | 261.86(251.52,272.05) | 261.86(251.52,272.05) | 0.00(0.00,0.00) |
| Uruguay | male | 5539.86(5303.37,5785.34) | 6046.22(5792.82,6311.93) | 0.09 | 368.41(352.28,385.03) | 368.41(352.28,385.03) | 0.00(0.00,0.00) |
| Uzbekistan | male | 24515.21(23482.86,25610.25) | 44832.77(42948.27,46670.54) | 0.83 | 261.86(251.52,272.05) | 261.86(251.52,272.05) | 0.00(0.00,0.00) |
| Vanuatu | male | 213.98(204.70,223.29) | 439.30(420.31,458.02) | 1.05 | 316.80(303.94,329.45) | 316.80(303.94,329.45) | 0.00(0.00,0.00) |
| Venezuela (Bolivarian Republic of) | male | 23030.58(22059.61,23996.05) | 37037.83(35589.55,38477.56) | 0.61 | 261.86(251.52,272.05) | 261.86(251.52,272.05) | 0.00(0.00,0.00) |
| Viet Nam | male | 113768.92(109003.14,118649.41) | 196883.48(189008.12,204648.79) | 0.73 | 377.73(363.56,392.09) | 377.73(363.56,392.09) | 0.00(0.00,0.00) |
| Yemen | male | 13143.00(12566.14,13695.27) | 34784.39(33236.40,36282.48) | 1.65 | 238.30(228.39,247.94) | 238.30(228.39,247.94) | 0.00(0.00,0.00) |
| Zambia | male | 8226.46(7868.46,8573.70) | 20685.23(19739.42,21569.99) | 1.51 | 254.99(244.35,264.87) | 254.99(244.35,264.87) | 0.00(0.00,0.00) |
| Zimbabwe | male | 10629.29(10175.04,11100.63) | 16493.29(15782.13,17224.89) | 0.55 | 252.12(241.85,262.23) | 252.12(241.85,262.23) | 0.00(0.00,0.00) |
| Afghanistan | female | 21911.40(20968.81,22787.50) | 72027.60(68560.12,75215.40) | 2.29 | 447.51(428.29,466.22) | 447.51(428.29,466.22) | 0.00(0.00,0.00) |
| Albania | female | 7913.46(7565.62,8245.86) | 7221.88(6931.21,7465.69) | -0.09 | 502.01(480.81,520.30) | 502.01(480.81,520.30) | 0.00(0.00,0.00) |
| Algeria | female | 49763.63(47431.20,51864.57) | 96816.89(92431.83,101083.33) | 0.95 | 447.51(428.29,466.22) | 447.51(428.29,466.22) | 0.00(0.00,0.00) |
| American Samoa | female | 126.41(120.89,131.92) | 154.23(148.27,160.07) | 0.22 | 570.95(549.03,592.87) | 570.95(549.03,592.87) | 0.00(0.00,0.00) |
| Andorra | female | 159.92(153.74,166.26) | 262.96(253.00,272.51) | 0.64 | 554.47(534.24,574.99) | 554.47(534.24,574.99) | 0.00(0.00,0.00) |
| Angola | female | 20653.63(19731.47,21575.24) | 63652.89(60835.16,66455.57) | 2.08 | 471.22(450.55,490.12) | 471.22(450.55,490.12) | 0.00(0.00,0.00) |
| Antigua and Barbuda | female | 157.69(150.79,163.97) | 248.37(237.87,257.46) | 0.58 | 502.01(480.81,520.30) | 502.01(480.81,520.30) | 0.00(0.00,0.00) |
| Argentina | female | 105975.03(101984.57,109796.68) | 155562.50(149667.81,161382.90) | 0.47 | 635.96(611.84,659.34) | 635.96(611.84,659.34) | 0.00(0.00,0.00) |
| Armenia | female | 8928.97(8533.92,9294.17) | 8520.40(8159.28,8836.60) | -0.05 | 502.01(480.81,520.30) | 502.01(480.81,520.30) | 0.00(0.00,0.00) |
| Australia | female | 57697.51(55490.28,59865.78) | 87405.96(84030.02,90513.60) | 0.51 | 635.96(611.84,659.34) | 635.96(611.84,659.34) | 0.00(0.00,0.00) |
| Austria | female | 24805.52(23912.67,25697.12) | 28339.06(27314.86,29311.52) | 0.14 | 554.47(534.24,574.99) | 554.47(534.24,574.99) | 0.00(0.00,0.00) |
| Azerbaijan | female | 18749.04(17924.08,19534.26) | 28472.09(27209.62,29600.24) | 0.52 | 502.01(480.81,520.30) | 502.01(480.81,520.30) | 0.00(0.00,0.00) |
| Bahamas | female | 668.33(638.63,697.07) | 1054.56(1010.10,1093.45) | 0.58 | 502.01(480.81,520.30) | 502.01(480.81,520.30) | 0.00(0.00,0.00) |
| Bahrain | female | 947.21(898.99,990.98) | 2749.15(2619.36,2871.83) | 1.90 | 447.51(428.29,466.22) | 447.51(428.29,466.22) | 0.00(0.00,0.00) |
| Bangladesh | female | 204197.46(195352.49,213253.85) | 371286.02(355682.08,386482.41) | 0.82 | 442.48(425.37,459.77) | 442.48(425.37,459.77) | 0.00(0.00,0.00) |
| Barbados | female | 689.48(659.35,715.95) | 825.39(792.98,853.60) | 0.20 | 502.01(480.81,520.30) | 502.01(480.81,520.30) | 0.00(0.00,0.00) |
| Belarus | female | 29071.92(27875.30,30136.86) | 26998.23(25927.70,27961.73) | -0.07 | 502.01(480.81,520.30) | 502.01(480.81,520.30) | 0.00(0.00,0.00) |
| Belgium | female | 31237.42(30099.25,32337.42) | 35412.87(34147.24,36619.46) | 0.13 | 554.47(534.24,574.99) | 554.47(534.24,574.99) | 0.00(0.00,0.00) |
| Belize | female | 401.65(384.19,418.39) | 1050.46(1005.03,1092.01) | 1.62 | 502.01(480.81,520.30) | 502.01(480.81,520.30) | 0.00(0.00,0.00) |
| Benin | female | 9971.96(9519.07,10418.22) | 26553.91(25357.52,27728.44) | 1.66 | 471.22(450.55,490.12) | 471.22(450.55,490.12) | 0.00(0.00,0.00) |
| Bermuda | female | 170.80(163.37,177.63) | 175.97(169.06,182.01) | 0.03 | 502.01(480.81,520.30) | 502.01(480.81,520.30) | 0.00(0.00,0.00) |
| Bhutan | female | 1139.01(1088.87,1188.53) | 1684.06(1611.73,1756.61) | 0.48 | 442.48(425.37,459.77) | 442.48(425.37,459.77) | 0.00(0.00,0.00) |
| Bolivia (Plurinational State of) | female | 14606.72(13987.13,15195.69) | 29636.97(28380.79,30781.58) | 1.03 | 502.01(480.81,520.30) | 502.01(480.81,520.30) | 0.00(0.00,0.00) |
| Bosnia and Herzegovina | female | 12021.86(11501.91,12478.25) | 9085.44(8734.19,9387.44) | -0.24 | 502.01(480.81,520.30) | 502.01(480.81,520.30) | 0.00(0.00,0.00) |
| Botswana | female | 2852.44(2723.17,2978.65) | 5836.09(5566.58,6100.83) | 1.05 | 471.22(450.55,490.12) | 471.22(450.55,490.12) | 0.00(0.00,0.00) |
| Brazil | female | 369679.17(354138.59,385195.02) | 599037.60(574540.00,622800.80) | 0.62 | 504.61(484.53,524.84) | 504.61(484.53,524.84) | 0.00(0.00,0.00) |
| Brunei Darussalam | female | 714.35(682.11,745.96) | 1443.63(1381.97,1503.75) | 1.02 | 632.20(607.89,655.84) | 632.20(607.89,655.84) | 0.00(0.00,0.00) |
| Bulgaria | female | 23273.53(22342.03,24098.63) | 18809.86(18086.91,19448.30) | -0.19 | 502.01(480.81,520.30) | 502.01(480.81,520.30) | 0.00(0.00,0.00) |
| Burkina Faso | female | 19624.52(18771.55,20464.47) | 48280.25(46112.85,50411.11) | 1.46 | 471.22(450.55,490.12) | 471.22(450.55,490.12) | 0.00(0.00,0.00) |
| Burundi | female | 11411.11(10941.35,11897.40) | 24302.69(23283.99,25338.29) | 1.13 | 466.25(447.04,484.40) | 466.25(447.04,484.40) | 0.00(0.00,0.00) |
| Cabo Verde | female | 759.57(726.87,791.62) | 1351.95(1289.86,1411.10) | 0.78 | 471.22(450.55,490.12) | 471.22(450.55,490.12) | 0.00(0.00,0.00) |
| Cambodia | female | 27337.05(26199.79,28444.65) | 49281.43(47285.04,51299.10) | 0.80 | 570.95(549.03,592.87) | 570.95(549.03,592.87) | 0.00(0.00,0.00) |
| Cameroon | female | 21436.81(20490.96,22373.26) | 63370.18(60434.71,66234.30) | 1.96 | 471.22(450.55,490.12) | 471.22(450.55,490.12) | 0.00(0.00,0.00) |
| Canada | female | 128479.10(123507.30,133429.53) | 177100.11(170522.05,183482.62) | 0.38 | 846.50(815.54,878.05) | 846.50(815.54,878.05) | 0.00(0.00,0.00) |
| Central African Republic | female | 5785.18(5528.49,6036.96) | 11532.06(11010.54,12036.58) | 0.99 | 471.22(450.55,490.12) | 471.22(450.55,490.12) | 0.00(0.00,0.00) |
| Chad | female | 12223.22(11690.14,12749.89) | 31769.48(30351.73,33187.47) | 1.60 | 471.22(450.55,490.12) | 471.22(450.55,490.12) | 0.00(0.00,0.00) |
| Chile | female | 42611.23(40907.27,44303.80) | 64931.50(62407.53,67280.08) | 0.52 | 635.96(611.84,659.34) | 635.96(611.84,659.34) | 0.00(0.00,0.00) |
| China | female | 3360054.88(3214174.44,3503103.10) | 4397276.68(4219224.87,4572097.49) | 0.31 | 565.13(541.69,587.11) | 565.13(541.69,587.11) | 0.00(0.00,0.00) |
| Colombia | female | 80239.00(76710.83,83644.50) | 128850.50(123407.20,133578.41) | 0.61 | 502.01(480.81,520.30) | 502.01(480.81,520.30) | 0.00(0.00,0.00) |
| Comoros | female | 938.41(900.75,976.84) | 1638.09(1570.46,1703.24) | 0.75 | 466.25(447.04,484.40) | 466.25(447.04,484.40) | 0.00(0.00,0.00) |
| Congo | female | 5098.62(4876.89,5321.78) | 11937.33(11393.44,12473.06) | 1.34 | 471.22(450.55,490.12) | 471.22(450.55,490.12) | 0.00(0.00,0.00) |
| Cook Islands | female | 49.52(47.49,51.46) | 53.75(51.73,55.83) | 0.09 | 570.95(549.03,592.87) | 570.95(549.03,592.87) | 0.00(0.00,0.00) |
| Costa Rica | female | 7332.60(7011.06,7641.06) | 13109.00(12541.70,13606.70) | 0.79 | 502.01(480.81,520.30) | 502.01(480.81,520.30) | 0.00(0.00,0.00) |
| Côte d'Ivoire | female | 24064.48(22962.69,25156.19) | 55238.76(52623.55,57789.17) | 1.30 | 471.22(450.55,490.12) | 471.22(450.55,490.12) | 0.00(0.00,0.00) |
| Croatia | female | 13446.12(12894.21,13924.99) | 11518.71(11073.19,11896.94) | -0.14 | 502.01(480.81,520.30) | 502.01(480.81,520.30) | 0.00(0.00,0.00) |
| Cuba | female | 28577.43(27353.60,29684.93) | 30399.41(29220.09,31380.56) | 0.06 | 502.01(480.81,520.30) | 502.01(480.81,520.30) | 0.00(0.00,0.00) |
| Cyprus | female | 2249.35(2166.84,2332.46) | 4291.70(4128.18,4449.82) | 0.91 | 554.47(534.24,574.99) | 554.47(534.24,574.99) | 0.00(0.00,0.00) |
| Czechia | female | 27678.62(26566.07,28655.10) | 28418.32(27320.59,29426.07) | 0.03 | 502.01(480.81,520.30) | 502.01(480.81,520.30) | 0.00(0.00,0.00) |
| Democratic People's Republic of Korea | female | 61913.10(59426.63,64408.04) | 79344.46(76242.60,82585.66) | 0.28 | 562.22(540.11,585.25) | 562.22(540.11,585.25) | 0.00(0.00,0.00) |
| Democratic Republic of the Congo | female | 78425.77(74990.95,81802.13) | 183279.67(175148.09,191280.97) | 1.34 | 471.22(450.55,490.12) | 471.22(450.55,490.12) | 0.00(0.00,0.00) |
| Denmark | female | 16005.88(15428.44,16565.12) | 17818.50(17164.73,18433.47) | 0.11 | 554.47(534.24,574.99) | 554.47(534.24,574.99) | 0.00(0.00,0.00) |
| Djibouti | female | 898.04(860.31,936.86) | 2644.00(2523.64,2757.63) | 1.94 | 466.25(447.04,484.40) | 466.25(447.04,484.40) | 0.00(0.00,0.00) |
| Dominica | female | 174.97(167.81,181.27) | 175.58(168.35,181.74) | 0.00 | 502.01(480.81,520.30) | 502.01(480.81,520.30) | 0.00(0.00,0.00) |
| Dominican Republic | female | 17327.26(16572.48,18052.46) | 27704.79(26530.90,28771.56) | 0.60 | 502.01(480.81,520.30) | 502.01(480.81,520.30) | 0.00(0.00,0.00) |
| Ecuador | female | 23525.10(22509.52,24479.13) | 44963.97(43064.41,46682.29) | 0.91 | 502.01(480.81,520.30) | 502.01(480.81,520.30) | 0.00(0.00,0.00) |
| Egypt | female | 111396.87(106292.39,116076.82) | 212152.86(202323.25,221220.79) | 0.90 | 447.51(428.29,466.22) | 447.51(428.29,466.22) | 0.00(0.00,0.00) |
| El Salvador | female | 12366.47(11850.89,12853.99) | 17208.48(16490.94,17846.40) | 0.39 | 502.01(480.81,520.30) | 502.01(480.81,520.30) | 0.00(0.00,0.00) |
| Equatorial Guinea | female | 904.14(864.41,942.58) | 2878.59(2743.80,3009.37) | 2.18 | 471.22(450.55,490.12) | 471.22(450.55,490.12) | 0.00(0.00,0.00) |
| Eritrea | female | 6020.45(5774.68,6271.23) | 14474.11(13867.37,15076.34) | 1.40 | 466.25(447.04,484.40) | 466.25(447.04,484.40) | 0.00(0.00,0.00) |
| Estonia | female | 4352.00(4178.13,4505.70) | 3593.19(3455.98,3714.89) | -0.17 | 502.01(480.81,520.30) | 502.01(480.81,520.30) | 0.00(0.00,0.00) |
| Eswatini | female | 1713.45(1636.38,1789.69) | 2716.90(2588.10,2839.95) | 0.59 | 471.22(450.55,490.12) | 471.22(450.55,490.12) | 0.00(0.00,0.00) |
| Ethiopia | female | 102142.19(97776.16,106654.50) | 224802.23(214926.75,234763.18) | 1.20 | 468.94(450.96,487.33) | 468.94(450.96,487.33) | 0.00(0.00,0.00) |
| Fiji | female | 2056.21(1967.75,2140.13) | 2600.45(2500.32,2702.21) | 0.26 | 570.95(549.03,592.87) | 570.95(549.03,592.87) | 0.00(0.00,0.00) |
| Finland | female | 15855.33(15282.42,16416.85) | 17196.13(16558.12,17779.27) | 0.08 | 554.47(534.24,574.99) | 554.47(534.24,574.99) | 0.00(0.00,0.00) |
| France | female | 177465.43(171078.34,183898.96) | 204972.44(197779.33,211904.65) | 0.15 | 554.47(534.24,574.99) | 554.47(534.24,574.99) | 0.00(0.00,0.00) |
| Gabon | female | 2058.98(1969.82,2144.75) | 4222.00(4030.69,4407.61) | 1.05 | 471.22(450.55,490.12) | 471.22(450.55,490.12) | 0.00(0.00,0.00) |
| Gambia | female | 1995.44(1901.54,2088.92) | 4932.59(4704.14,5156.19) | 1.47 | 471.22(450.55,490.12) | 471.22(450.55,490.12) | 0.00(0.00,0.00) |
| Georgia | female | 15108.28(14469.28,15675.15) | 9935.78(9546.36,10287.96) | -0.34 | 502.01(480.81,520.30) | 502.01(480.81,520.30) | -0.22(-2.73,2.36) |
| Germany | female | 257994.28(248550.23,267052.82) | 266756.13(257206.55,275626.77) | 0.03 | 554.47(534.24,574.99) | 554.47(534.24,574.99) | 0.00(0.00,0.00) |
| Ghana | female | 31531.76(30099.78,32943.37) | 75191.23(71721.65,78534.10) | 1.38 | 471.22(450.55,490.12) | 471.22(450.55,490.12) | 0.00(0.00,0.00) |
| Greece | female | 32067.74(30903.62,33199.62) | 33140.04(31957.33,34298.88) | 0.03 | 554.47(534.24,574.99) | 554.47(534.24,574.99) | 0.00(0.00,0.00) |
| Greenland | female | 221.72(212.30,231.05) | 246.24(236.94,255.53) | 0.11 | 846.50(815.54,878.05) | 846.50(815.54,878.05) | 0.00(0.00,0.00) |
| Grenada | female | 199.66(191.19,207.26) | 266.55(255.55,276.13) | 0.34 | 502.01(480.81,520.30) | 502.01(480.81,520.30) | 0.00(0.00,0.00) |
| Guam | female | 369.89(353.80,385.99) | 468.58(450.95,486.46) | 0.27 | 570.95(549.03,592.87) | 570.95(549.03,592.87) | 0.00(0.00,0.00) |
| Guatemala | female | 17395.85(16654.39,18099.45) | 45939.87(43928.15,47839.47) | 1.64 | 502.01(480.81,520.30) | 502.01(480.81,520.30) | 0.00(0.00,0.00) |
| Guinea | female | 12996.79(12412.23,13565.73) | 27320.26(26078.65,28559.31) | 1.10 | 471.22(450.55,490.12) | 471.22(450.55,490.12) | 0.00(0.00,0.00) |
| Guinea-Bissau | female | 2113.45(2018.26,2207.62) | 4280.21(4076.11,4475.74) | 1.03 | 471.22(450.55,490.12) | 471.22(450.55,490.12) | 0.00(0.00,0.00) |
| Guyana | female | 1862.77(1780.55,1942.02) | 2014.13(1929.05,2090.73) | 0.08 | 502.01(480.81,520.30) | 502.01(480.81,520.30) | 0.00(0.00,0.00) |
| Haiti | female | 14765.40(14129.74,15364.21) | 31991.99(30574.84,33314.03) | 1.17 | 502.01(480.81,520.30) | 502.01(480.81,520.30) | 0.00(0.00,0.00) |
| Honduras | female | 10145.58(9708.07,10559.75) | 25076.54(23991.96,26101.60) | 1.47 | 502.01(480.81,520.30) | 502.01(480.81,520.30) | 0.00(0.00,0.00) |
| Hungary | female | 28399.99(27259.53,29393.63) | 26784.73(25771.80,27710.40) | -0.06 | 502.01(480.81,520.30) | 502.01(480.81,520.30) | 0.00(0.00,0.00) |
| Iceland | female | 725.25(699.69,752.60) | 1027.24(989.72,1062.85) | 0.42 | 554.47(534.24,574.99) | 554.47(534.24,574.99) | 0.00(0.00,0.00) |
| India | female | 1702549.75(1630567.63,1776814.27) | 3128154.07(2995513.59,3261137.23) | 0.84 | 444.69(426.28,462.71) | 444.69(426.28,462.71) | 0.00(0.00,0.00) |
| Indonesia | female | 512421.13(490839.57,533396.71) | 779800.67(748684.25,810598.50) | 0.52 | 574.06(551.77,595.96) | 574.06(551.77,595.96) | 0.00(0.00,0.00) |
| Iran (Islamic Republic of) | female | 112718.84(107950.80,117261.46) | 204096.95(195386.50,212738.33) | 0.81 | 450.26(432.27,466.75) | 450.26(432.27,466.75) | 0.00(0.00,0.00) |
| Iraq | female | 32709.24(31184.87,34095.51) | 91434.87(87158.29,95327.99) | 1.80 | 447.51(428.29,466.22) | 447.51(428.29,466.22) | 0.00(0.00,0.00) |
| Ireland | female | 10109.25(9757.85,10481.48) | 15014.18(14467.55,15552.92) | 0.49 | 554.47(534.24,574.99) | 554.47(534.24,574.99) | 0.00(0.00,0.00) |
| Israel | female | 13667.42(13182.60,14171.06) | 26079.05(25123.05,26987.50) | 0.91 | 554.47(534.24,574.99) | 554.47(534.24,574.99) | 0.00(0.00,0.00) |
| Italy | female | 182424.45(175937.77,189336.88) | 194240.56(187458.75,201450.90) | 0.06 | 556.95(535.94,578.44) | 556.95(535.94,578.44) | 0.00(0.00,0.00) |
| Jamaica | female | 5733.42(5490.54,5957.08) | 7589.68(7267.22,7876.13) | 0.32 | 502.01(480.81,520.30) | 502.01(480.81,520.30) | 0.00(0.00,0.00) |
| Japan | female | 453797.28(437547.33,470204.85) | 465288.41(448711.27,482225.71) | 0.03 | 636.94(613.13,660.21) | 636.94(613.13,660.21) | 0.00(0.00,0.00) |
| Jordan | female | 6886.10(6559.33,7190.87) | 23826.86(22738.41,24822.56) | 2.46 | 447.51(428.29,466.22) | 447.51(428.29,466.22) | 0.00(0.00,0.00) |
| Kazakhstan | female | 42346.18(40539.54,44019.08) | 49430.16(47321.66,51294.73) | 0.17 | 502.01(480.81,520.30) | 502.01(480.81,520.30) | 0.00(0.00,0.00) |
| Kenya | female | 45664.34(43652.85,47708.83) | 111826.26(107030.52,116765.24) | 1.45 | 468.94(450.96,487.33) | 468.94(450.96,487.33) | 0.00(0.00,0.00) |
| Kiribati | female | 200.99(192.41,209.52) | 339.96(326.04,353.84) | 0.69 | 570.95(549.03,592.87) | 570.95(549.03,592.87) | 0.00(0.00,0.00) |
| Kuwait | female | 3358.47(3183.34,3516.84) | 10930.65(10367.87,11474.30) | 2.25 | 447.51(428.29,466.22) | 447.51(428.29,466.22) | 0.00(0.00,0.00) |
| Kyrgyzstan | female | 10737.50(10272.26,11173.28) | 16665.51(15950.30,17318.61) | 0.55 | 502.01(480.81,520.30) | 502.01(480.81,520.30) | 0.00(0.00,0.00) |
| Lao People's Democratic Republic | female | 10642.35(10213.35,11059.45) | 20532.75(19665.58,21386.55) | 0.93 | 570.95(549.03,592.87) | 570.95(549.03,592.87) | 0.00(0.00,0.00) |
| Latvia | female | 7436.00(7141.50,7691.17) | 5334.70(5129.95,5511.49) | -0.28 | 502.01(480.81,520.30) | 502.01(480.81,520.30) | 0.00(0.00,0.00) |
| Lebanon | female | 6759.12(6461.97,7043.56) | 12375.49(11815.65,12911.09) | 0.83 | 447.51(428.29,466.22) | 447.51(428.29,466.22) | 0.00(0.00,0.00) |
| Lesotho | female | 3874.49(3709.41,4040.81) | 5002.93(4769.05,5223.41) | 0.29 | 471.22(450.55,490.12) | 471.22(450.55,490.12) | 0.00(0.00,0.00) |
| Liberia | female | 4035.19(3857.22,4212.45) | 10531.07(10046.04,11011.60) | 1.61 | 471.22(450.55,490.12) | 471.22(450.55,490.12) | 0.00(0.00,0.00) |
| Libya | female | 7545.48(7191.53,7867.64) | 16092.25(15347.52,16806.84) | 1.13 | 447.51(428.29,466.22) | 447.51(428.29,466.22) | 0.00(0.00,0.00) |
| Lithuania | female | 10136.43(9725.14,10500.59) | 7805.70(7515.95,8059.02) | -0.23 | 502.01(480.81,520.30) | 502.01(480.81,520.30) | 0.00(0.00,0.00) |
| Luxembourg | female | 1216.16(1171.06,1259.95) | 1934.76(1863.95,2002.95) | 0.59 | 554.47(534.24,574.99) | 554.47(534.24,574.99) | 0.00(0.00,0.00) |
| Madagascar | female | 23923.26(22951.84,24932.24) | 57059.90(54665.60,59450.51) | 1.39 | 466.25(447.04,484.40) | 466.25(447.04,484.40) | 0.00(0.00,0.00) |
| Malawi | female | 19475.33(18674.71,20302.41) | 39608.02(37918.02,41275.73) | 1.03 | 466.25(447.04,484.40) | 466.25(447.04,484.40) | 0.00(0.00,0.00) |
| Malaysia | female | 47781.97(45773.24,49748.80) | 91504.43(87734.34,95243.85) | 0.92 | 570.95(549.03,592.87) | 570.95(549.03,592.87) | 0.00(0.00,0.00) |
| Maldives | female | 517.43(495.89,538.74) | 1222.16(1166.99,1277.53) | 1.36 | 570.95(549.03,592.87) | 570.95(549.03,592.87) | 0.00(0.00,0.00) |
| Mali | female | 17701.54(16921.82,18466.44) | 44230.00(42252.29,46207.16) | 1.50 | 471.22(450.55,490.12) | 471.22(450.55,490.12) | 0.00(0.00,0.00) |
| Malta | female | 1119.91(1077.78,1160.52) | 1388.96(1335.28,1438.64) | 0.24 | 554.47(534.24,574.99) | 554.47(534.24,574.99) | 0.00(0.00,0.00) |
| Marshall Islands | female | 107.03(102.55,111.43) | 158.09(151.55,164.44) | 0.48 | 570.95(549.03,592.87) | 570.95(549.03,592.87) | 0.00(0.00,0.00) |
| Mauritania | female | 4264.35(4075.62,4451.14) | 8856.69(8458.15,9238.83) | 1.08 | 471.22(450.55,490.12) | 471.22(450.55,490.12) | 0.00(0.00,0.00) |
| Mauritius | female | 3237.56(3100.22,3376.68) | 3998.41(3844.33,4152.09) | 0.24 | 570.95(549.03,592.87) | 570.95(549.03,592.87) | 0.00(0.00,0.00) |
| Mexico | female | 203180.19(194289.96,211715.85) | 335668.36(322063.66,349410.82) | 0.65 | 504.61(484.53,524.84) | 504.61(484.53,524.84) | 0.00(0.00,0.00) |
| Micronesia (Federated States of) | female | 251.37(241.16,261.52) | 283.98(272.52,295.10) | 0.13 | 570.95(549.03,592.87) | 570.95(549.03,592.87) | 0.00(0.00,0.00) |
| Monaco | female | 102.11(98.31,105.75) | 119.89(115.65,123.93) | 0.17 | 554.47(534.24,574.99) | 554.47(534.24,574.99) | 0.00(0.00,0.00) |
| Mongolia | female | 4872.47(4658.36,5077.66) | 8980.20(8585.22,9338.77) | 0.84 | 502.01(480.81,520.30) | 502.01(480.81,520.30) | 0.00(0.00,0.00) |
| Montenegro | female | 1621.68(1553.30,1680.86) | 1673.46(1607.56,1731.86) | 0.03 | 502.01(480.81,520.30) | 502.01(480.81,520.30) | 0.00(0.00,0.00) |
| Morocco | female | 52903.71(50467.84,55158.11) | 83680.78(79983.66,87272.76) | 0.58 | 447.51(428.29,466.22) | 447.51(428.29,466.22) | 0.00(0.00,0.00) |
| Mozambique | female | 27504.94(26382.74,28614.01) | 61714.64(59169.85,64296.60) | 1.24 | 466.25(447.04,484.40) | 466.25(447.04,484.40) | 0.00(0.00,0.00) |
| Myanmar | female | 110979.28(106426.82,115492.67) | 167781.67(161145.33,174455.58) | 0.51 | 570.95(549.03,592.87) | 570.95(549.03,592.87) | 0.00(0.00,0.00) |
| Namibia | female | 3024.25(2891.09,3156.30) | 5700.08(5438.12,5948.99) | 0.88 | 471.22(450.55,490.12) | 471.22(450.55,490.12) | 0.00(0.00,0.00) |
| Nauru | female | 25.56(24.46,26.63) | 28.94(27.67,30.12) | 0.13 | 570.95(549.03,592.87) | 570.95(549.03,592.87) | 0.00(0.00,0.00) |
| Nepal | female | 38365.56(36729.08,40010.10) | 72255.41(69175.73,75242.29) | 0.88 | 442.48(425.37,459.77) | 442.48(425.37,459.77) | 0.00(0.00,0.00) |
| Netherlands | female | 46251.34(44417.18,47979.64) | 53394.30(51399.47,55368.14) | 0.15 | 554.63(532.96,575.06) | 555.16(535.53,576.62) | 0.00(0.00,0.00) |
| New Zealand | female | 11658.34(11243.11,12102.30) | 16040.69(15488.55,16599.73) | 0.38 | 639.60(616.24,663.81) | 639.60(616.24,663.81) | 0.00(0.00,0.00) |
| Nicaragua | female | 8504.28(8131.69,8861.13) | 16822.84(16093.31,17500.63) | 0.98 | 502.01(480.81,520.30) | 502.01(480.81,520.30) | 0.00(0.00,0.00) |
| Niger | female | 15608.58(14909.80,16305.27) | 44201.72(42249.06,46122.19) | 1.83 | 471.22(450.55,490.12) | 471.22(450.55,490.12) | 0.00(0.00,0.00) |
| Nigeria | female | 181412.72(174162.40,188696.69) | 470954.14(451992.77,489585.31) | 1.60 | 473.51(455.35,491.12) | 473.51(455.35,491.12) | 0.00(0.00,0.00) |
| Niue | female | 6.03(5.80,6.25) | 4.78(4.60,4.96) | -0.21 | 570.95(549.03,592.87) | 570.95(549.03,592.87) | 0.00(0.00,0.00) |
| North Macedonia | female | 5188.16(4965.47,5379.26) | 5835.49(5600.57,6044.62) | 0.12 | 502.01(480.81,520.30) | 502.01(480.81,520.30) | 0.00(0.00,0.00) |
| Northern Mariana Islands | female | 133.24(126.71,139.87) | 122.26(117.48,126.83) | -0.08 | 570.95(549.03,592.87) | 570.95(549.03,592.87) | 0.00(0.00,0.00) |
| Norway | female | 12959.01(12492.88,13448.02) | 16252.12(15683.28,16868.23) | 0.25 | 556.95(535.94,578.44) | 556.95(535.94,578.44) | 0.00(0.00,0.00) |
| Oman | female | 3002.32(2860.08,3131.32) | 7769.62(7381.65,8135.55) | 1.59 | 447.51(428.29,466.22) | 447.51(428.29,466.22) | 0.00(0.00,0.00) |
| Pakistan | female | 202491.56(194240.44,211278.76) | 452433.43(433236.81,472658.52) | 1.23 | 444.69(426.28,462.71) | 444.69(426.28,462.71) | 0.00(0.00,0.00) |
| Palau | female | 43.18(41.35,44.99) | 49.18(47.30,51.08) | 0.14 | 570.95(549.03,592.87) | 570.95(549.03,592.87) | 0.00(0.00,0.00) |
| Palestine | female | 3816.87(3643.55,3980.65) | 10324.84(9846.38,10763.20) | 1.71 | 447.51(428.29,466.22) | 447.51(428.29,466.22) | 0.00(0.00,0.00) |
| Panama | female | 5701.10(5455.80,5934.24) | 10461.79(10022.69,10841.25) | 0.84 | 502.01(480.81,520.30) | 502.01(480.81,520.30) | 0.00(0.00,0.00) |
| Papua New Guinea | female | 10226.99(9788.10,10648.69) | 26144.80(25035.44,27222.31) | 1.56 | 570.95(549.03,592.87) | 570.95(549.03,592.87) | 0.00(0.00,0.00) |
| Paraguay | female | 9092.71(8703.35,9457.39) | 17607.69(16849.93,18299.63) | 0.94 | 502.01(480.81,520.30) | 502.01(480.81,520.30) | 0.00(0.00,0.00) |
| Peru | female | 50944.67(48742.04,52993.96) | 88046.31(84315.48,91349.93) | 0.73 | 502.01(480.81,520.30) | 502.01(480.81,520.30) | 0.00(0.00,0.00) |
| Philippines | female | 166007.70(158884.14,172819.63) | 314994.90(302364.14,327654.00) | 0.90 | 574.06(551.77,595.96) | 574.06(551.77,595.96) | 0.00(0.00,0.00) |
| Poland | female | 102323.19(98117.32,106236.30) | 107407.50(103101.68,111582.53) | 0.05 | 504.61(484.53,524.84) | 504.61(484.53,524.84) | 0.00(0.00,0.00) |
| Portugal | female | 31465.64(30354.26,32585.46) | 35257.32(33973.61,36501.32) | 0.12 | 554.47(534.24,574.99) | 554.47(534.24,574.99) | 0.00(0.00,0.00) |
| Puerto Rico | female | 9528.72(9130.12,9878.98) | 9746.25(9370.78,10080.13) | 0.02 | 502.01(480.81,520.30) | 502.01(480.81,520.30) | 0.00(0.00,0.00) |
| Qatar | female | 640.72(607.76,671.39) | 3688.48(3495.76,3873.43) | 4.76 | 447.51(428.29,466.22) | 447.51(428.29,466.22) | 0.00(0.00,0.00) |
| Republic of Korea | female | 145262.80(139238.01,151129.99) | 195630.74(188224.75,202855.40) | 0.35 | 632.20(607.89,655.84) | 632.20(607.89,655.84) | 0.00(0.00,0.00) |
| Republic of Moldova | female | 12045.14(11526.99,12502.08) | 10595.17(10169.84,10981.52) | -0.12 | 502.01(480.81,520.30) | 502.01(480.81,520.30) | 0.00(0.00,0.00) |
| Romania | female | 61234.64(58696.39,63339.87) | 51708.74(49729.22,53492.08) | -0.16 | 502.01(480.81,520.30) | 502.01(480.81,520.30) | 0.00(0.00,0.00) |
| Russian Federation | female | 425547.18(408503.01,441511.92) | 419325.47(402287.37,435582.95) | -0.01 | 504.61(484.53,524.84) | 504.61(484.53,524.84) | 0.00(0.00,0.00) |
| Rwanda | female | 14608.65(14012.14,15226.07) | 28758.31(27561.62,29953.70) | 0.97 | 466.25(447.04,484.40) | 466.25(447.04,484.40) | 0.00(0.00,0.00) |
| Saint Kitts and Nevis | female | 101.36(96.82,105.38) | 161.31(154.59,167.26) | 0.59 | 502.01(480.81,520.30) | 502.01(480.81,520.30) | 0.00(0.00,0.00) |
| Saint Lucia | female | 327.39(313.60,340.43) | 478.50(458.60,495.59) | 0.46 | 502.01(480.81,520.30) | 502.01(480.81,520.30) | 0.00(0.00,0.00) |
| Saint Vincent and the Grenadines | female | 255.48(244.65,265.63) | 290.26(278.15,300.57) | 0.14 | 502.01(480.81,520.30) | 502.01(480.81,520.30) | 0.00(0.00,0.00) |
| Samoa | female | 398.90(383.16,414.39) | 554.53(532.95,575.71) | 0.39 | 570.95(549.03,592.87) | 570.95(549.03,592.87) | 0.00(0.00,0.00) |
| San Marino | female | 71.09(68.56,73.69) | 105.88(101.99,109.68) | 0.49 | 554.47(534.24,574.99) | 554.47(534.24,574.99) | 0.00(0.00,0.00) |
| Sao Tome and Principe | female | 242.14(231.71,252.47) | 460.93(439.98,481.40) | 0.90 | 471.22(450.55,490.12) | 471.22(450.55,490.12) | 0.00(0.00,0.00) |
| Saudi Arabia | female | 27272.74(25965.66,28454.41) | 76579.24(72735.98,80246.32) | 1.81 | 447.51(428.29,466.22) | 447.51(428.29,466.22) | 0.00(0.00,0.00) |
| Senegal | female | 15501.28(14814.87,16177.82) | 32836.63(31361.32,34285.75) | 1.12 | 471.22(450.55,490.12) | 471.22(450.55,490.12) | 0.00(0.00,0.00) |
| Serbia | female | 24984.05(23954.40,25880.96) | 23389.40(22482.76,24195.51) | -0.06 | 502.01(480.81,520.30) | 502.01(480.81,520.30) | 0.00(0.00,0.00) |
| Seychelles | female | 202.21(193.61,210.42) | 290.03(278.94,301.62) | 0.43 | 570.95(549.03,592.87) | 570.95(549.03,592.87) | 0.00(0.00,0.00) |
| Sierra Leone | female | 7817.59(7461.22,8165.91) | 18192.60(17343.31,19014.01) | 1.33 | 471.22(450.55,490.12) | 471.22(450.55,490.12) | 0.00(0.00,0.00) |
| Singapore | female | 10493.17(10033.29,10939.52) | 20907.10(20069.00,21746.70) | 0.99 | 632.20(607.89,655.84) | 632.20(607.89,655.84) | 0.00(0.00,0.00) |
| Slovakia | female | 14008.56(13416.45,14518.63) | 15069.53(14478.84,15614.72) | 0.08 | 502.01(480.81,520.30) | 502.01(480.81,520.30) | 0.00(0.00,0.00) |
| Slovenia | female | 5395.47(5169.04,5588.73) | 5440.27(5229.00,5628.24) | 0.01 | 502.01(480.81,520.30) | 502.01(480.81,520.30) | 0.00(0.00,0.00) |
| Solomon Islands | female | 795.25(762.28,827.08) | 1722.14(1648.93,1790.43) | 1.17 | 570.95(549.03,592.87) | 570.95(549.03,592.87) | 0.00(0.00,0.00) |
| Somalia | female | 13704.07(13121.40,14256.02) | 39542.58(37880.75,41170.37) | 1.89 | 466.25(447.04,484.40) | 466.25(447.04,484.40) | 0.00(0.00,0.00) |
| South Africa | female | 86677.14(83169.88,90053.00) | 140911.58(135036.40,146634.52) | 0.63 | 473.51(455.35,491.12) | 473.51(455.35,491.12) | 0.00(0.00,0.00) |
| South Sudan | female | 11054.68(10577.98,11530.48) | 18873.65(18085.94,19637.97) | 0.71 | 466.25(447.04,484.40) | 466.25(447.04,484.40) | 0.00(0.00,0.00) |
| Spain | female | 118577.58(114395.01,122813.16) | 146973.80(141616.81,152220.79) | 0.24 | 554.47(534.24,574.99) | 554.47(534.24,574.99) | 0.00(0.00,0.00) |
| Sri Lanka | female | 48836.59(46819.56,50863.64) | 67431.18(64852.94,70063.70) | 0.38 | 570.95(549.03,592.87) | 570.95(549.03,592.87) | 0.00(0.00,0.00) |
| Sudan | female | 39118.43(37271.23,40776.59) | 85630.32(81469.03,89280.44) | 1.19 | 447.51(428.29,466.22) | 447.51(428.29,466.22) | 0.00(0.00,0.00) |
| Suriname | female | 925.40(886.16,961.34) | 1505.54(1443.22,1560.36) | 0.63 | 502.01(480.81,520.30) | 502.01(480.81,520.30) | 0.00(0.00,0.00) |
| Sweden | female | 26432.35(25504.30,27409.32) | 30917.49(29856.93,32077.32) | 0.17 | 556.95(535.94,578.44) | 556.95(535.94,578.44) | 0.00(0.00,0.00) |
| Switzerland | female | 21610.07(20814.99,22401.19) | 27577.06(26559.44,28539.32) | 0.28 | 554.47(534.24,574.99) | 554.47(534.24,574.99) | 0.00(0.00,0.00) |
| Syrian Arab Republic | female | 23629.81(22518.21,24651.46) | 33334.36(31870.81,34711.35) | 0.41 | 447.51(428.29,466.22) | 447.51(428.29,466.22) | 0.00(0.00,0.00) |
| Taiwan (Province of China) | female | 57841.73(55312.23,60193.18) | 73067.95(70397.93,75686.45) | 0.26 | 553.64(531.50,574.25) | 553.64(531.50,574.25) | 0.00(0.00,0.00) |
| Tajikistan | female | 11956.00(11433.63,12457.34) | 23405.32(22374.78,24374.41) | 0.96 | 502.01(480.81,520.30) | 502.01(480.81,520.30) | 0.00(0.00,0.00) |
| Thailand | female | 168551.02(161440.95,175554.48) | 225125.43(216391.04,234034.15) | 0.34 | 570.95(549.03,592.87) | 570.95(549.03,592.87) | 0.00(0.00,0.00) |
| Timor-Leste | female | 1964.10(1878.02,2051.94) | 3478.16(3336.99,3615.86) | 0.77 | 570.95(549.03,592.87) | 570.95(549.03,592.87) | 0.00(0.00,0.00) |
| Togo | female | 7550.06(7209.98,7888.79) | 18069.66(17235.30,18887.48) | 1.39 | 471.22(450.55,490.12) | 471.22(450.55,490.12) | 0.00(0.00,0.00) |
| Tokelau | female | 4.28(4.12,4.45) | 3.74(3.60,3.88) | -0.13 | 570.95(549.03,592.87) | 570.95(549.03,592.87) | 0.00(0.00,0.00) |
| Tonga | female | 245.39(236.01,254.86) | 279.40(268.37,289.88) | 0.14 | 570.95(549.03,592.87) | 570.95(549.03,592.87) | 0.00(0.00,0.00) |
| Trinidad and Tobago | female | 2967.04(2838.76,3086.06) | 3731.91(3576.24,3869.55) | 0.26 | 502.01(480.81,520.30) | 502.01(480.81,520.30) | 0.00(0.00,0.00) |
| Tunisia | female | 17458.58(16648.23,18197.85) | 27961.84(26726.58,29138.64) | 0.60 | 447.51(428.29,466.22) | 447.51(428.29,466.22) | 0.00(0.00,0.00) |
| Turkey | female | 125065.05(119440.73,130257.25) | 197055.52(188353.08,205317.46) | 0.58 | 447.51(428.29,466.22) | 447.51(428.29,466.22) | 0.00(0.00,0.00) |
| Turkmenistan | female | 8656.46(8276.63,9028.89) | 12577.60(12041.36,13048.00) | 0.45 | 502.01(480.81,520.30) | 502.01(480.81,520.30) | 0.00(0.00,0.00) |
| Tuvalu | female | 27.34(26.25,28.44) | 32.29(31.03,33.55) | 0.18 | 570.95(549.03,592.87) | 570.95(549.03,592.87) | 0.00(0.00,0.00) |
| Uganda | female | 33934.49(32532.03,35399.65) | 84454.95(80882.76,88036.80) | 1.49 | 466.25(447.04,484.40) | 466.25(447.04,484.40) | 0.00(0.00,0.00) |
| Ukraine | female | 148872.66(143047.20,154416.00) | 129050.45(123831.48,134047.01) | -0.13 | 504.61(484.53,524.84) | 504.61(484.53,524.84) | 0.00(0.00,0.00) |
| United Arab Emirates | female | 2796.36(2648.31,2929.52) | 13237.35(12471.99,13941.38) | 3.73 | 447.51(428.29,466.22) | 447.51(428.29,466.22) | 0.00(0.00,0.00) |
| United Kingdom | female | 179556.59(173408.75,186092.75) | 208866.36(201858.75,216424.39) | 0.16 | 556.53(536.44,577.26) | 556.56(536.43,577.48) | 0.00(0.00,0.00) |
| United Republic of Tanzania | female | 52260.99(50001.39,54424.15) | 121543.22(116377.46,126806.61) | 1.33 | 460.22(441.94,478.83) | 465.85(446.53,483.50) | 0.03(0.01,0.04) |
| United States of America | female | 1256886.38(1220072.39,1291807.85) | 1651646.85(1605215.51,1695379.56) | 0.31 | 902.66(876.63,927.49) | 895.03(869.74,919.42) | -0.27(-0.38,-0.16) |
| United States Virgin Islands | female | 277.51(265.97,287.81) | 282.68(271.81,292.22) | 0.02 | 502.01(480.81,520.30) | 502.01(480.81,520.30) | 0.00(0.00,0.00) |
| Uruguay | female | 10542.68(10144.41,10913.67) | 12209.07(11748.83,12643.62) | 0.16 | 635.96(611.84,659.34) | 635.96(611.84,659.34) | 0.00(0.00,0.00) |
| Uzbekistan | female | 48344.75(46230.33,50388.12) | 87363.61(83545.91,90839.02) | 0.81 | 502.01(480.81,520.30) | 502.01(480.81,520.30) | 0.00(0.00,0.00) |
| Vanuatu | female | 372.96(356.92,388.87) | 791.75(758.58,823.95) | 1.12 | 570.95(549.03,592.87) | 570.95(549.03,592.87) | 0.00(0.00,0.00) |
| Venezuela (Bolivarian Republic of) | female | 45102.13(43128.41,46970.95) | 74945.77(71803.93,77699.37) | 0.66 | 502.01(480.81,520.30) | 502.01(480.81,520.30) | 0.00(0.00,0.00) |
| Viet Nam | female | 188486.76(180430.50,196384.85) | 302539.71(290556.45,314913.72) | 0.61 | 570.95(549.03,592.87) | 570.95(549.03,592.87) | 0.00(0.00,0.00) |
| Yemen | female | 24403.86(23252.55,25455.68) | 64566.65(61490.58,67294.92) | 1.65 | 447.51(428.29,466.22) | 447.51(428.29,466.22) | 0.00(0.00,0.00) |
| Zambia | female | 15792.34(15134.71,16479.27) | 38876.93(37222.21,40562.49) | 1.46 | 466.25(447.04,484.40) | 466.25(447.04,484.40) | 0.00(0.00,0.00) |
| Zimbabwe | female | 21225.13(20271.51,22170.55) | 34462.30(32886.00,36026.76) | 0.62 | 471.22(450.55,490.12) | 471.22(450.55,490.12) | 0.00(0.00,0.00) |

**Supplementary table 4. The DALYs and age-standardized DALY rate of alopecia areata in 1990 and 2019, and its temporal trends from 1990 to 2019.**

| **Nation** | **Sex** | **DALY Cases No. (95% UI)** | | **Change in absolute number (%)** | **Age-standardized DALY rate per 100,000 No.(95% UI)** | | **1990-2019 EAPC No. (95%CI)** |
| --- | --- | --- | --- | --- | --- | --- | --- |
|  |  | **1990** | **2019** |  | **1990** | **2019** |  |
| Afghanistan | both | 594.01(372.07,903.33) | 2058.32(1303.32,3048.60) | 2.47 | 6.35(4.03,9.64) | 6.23(3.98,9.07) | -0.05(-0.06,-0.04) |
| Albania | both | 227.73(145.17,341.34) | 204.33(128.89,304.82) | -0.10 | 7.04(4.53,10.52) | 7.08(4.48,10.60) | 0.03(0.01,0.04) |
| Algeria | both | 1421.74(894.39,2125.42) | 2779.45(1760.93,4150.23) | 0.95 | 6.35(4.02,9.36) | 6.35(4.02,9.47) | 0.00(-0.01,0.00) |
| American Samoa | both | 3 .69(2.36,5.55) | 4.41(2.80,6.60) | 0.20 | 8.17(5.22,12.22) | 8.20(5.21,12.29) | 0.02(0.01,0.02) |
| Andorra | both | 5.45(3.43,8.18) | 8.01(5.12,11.85) | 0.47 | 8.73(5.52,13.06) | 8.76(5.58,13.10) | 0.02(0.01,0.03) |
| Angola | both | 587.03(373.64,887.16) | 1746.67(1104.77,2633.42) | 1.98 | 6.63(4.25,9.90) | 6.79(4.30,10.19) | 0.09(0.09,0.10) |
| Antigua and Barbuda | both | 4.32(2.74,6.40) | 6.88(4.29,10.36) | 0.59 | 7.19(4.58,10.61) | 7.13(4.44,10.73) | -0.04(-0.05,-0.02) |
| Argentina | both | 3050.39(1939.49,4569.92) | 4432.10(2811.62,6645.08) | 0.45 | 9.42(5.98,14.11) | 9.41(5.97,14.12) | 0.00(-0.01,0.00) |
| Armenia | both | 247.46(156.33,367.87) | 234.89(147.41,347.10) | -0.05 | 7.17(4.52,10.68) | 7.18(4.52,10.64) | 0.00(-0.01,0.01) |
| Australia | both | 1665.44(1046.25,2481.68) | 2426.03(1528.79,3635.38) | 0.46 | 9.30(5.84,13.88) | 9.32(5.87,13.90) | 0.01(0.00,0.01) |
| Austria | both | 753.84(476.23,1128.49) | 844.40(532.55,1265.19) | 0.12 | 8.92(5.63,13.41) | 8.86(5.57,13.37) | -0.02(-0.03,-0.02) |
| Azerbaijan | both | 516.80(329.45,774.53) | 801.99(503.16,1201.67) | 0.55 | 7.21(4.57,10.82) | 7.11(4.47,10.69) | -0.05(-0.05,-0.04) |
| Bahamas | both | 18.52(11.85,28.10) | 29.10(18.43,43.46) | 0.57 | 7.17(4.60,10.84) | 7.16(4.56,10.66) | 0.00(-0.01,0.01) |
| Bahrain | both | 32.27(20.34,48.38) | 100.25(62.69,151.31) | 2.11 | 5.95(3.78,8.86) | 5.93(3.73,8.96) | -0.08(-0.11,-0.05) |
| Bangladesh | both | 5886.64(3661.63,8892.69) | 10329.43(6525.33,15562.18) | 0.75 | 6.16(3.88,9.19) | 6.30(3.99,9.48) | 0.10(0.09,0.11) |
| Barbados | both | 18.91(12.01,28.27) | 22.74(14.54,34.06) | 0.20 | 7.17(4.54,10.79) | 7.16(4.56,10.66) | -0.01(-0.02,-0.01) |
| Belarus | both | 790.75(505.42,1174.46) | 737.17(469.46,1100.07) | -0.07 | 7.19(4.59,10.67) | 7.16(4.52,10.72) | -0.01(-0.01,0.00) |
| Belgium | both | 953.94(607.75,1429.52) | 1047.13(654.27,1551.96) | 0.10 | 8.86(5.69,13.30) | 8.81(5.49,13.23) | -0.03(-0.03,-0.02) |
| Belize | both | 11.42(7.28,17.29) | 29.36(18.33,44.50) | 1.57 | 7.06(4.45,10.67) | 7.10(4.49,10.71) | 0.03(0.02,0.03) |
| Benin | both | 270.08(169.74,401.44) | 737.31(466.38,1113.48) | 1.73 | 6.83(4.29,10.13) | 6.77(4.30,10.11) | -0.02(-0.03,-0.02) |
| Bermuda | both | 4.75(2.99,7.11) | 4.88(3.09,7.28) | 0.03 | 7.16(4.48,10.77) | 7.14(4.54,10.64) | -0.01(-0.02,0.00) |
| Bhutan | both | 33.57(21.46,50.71) | 49.58(31.89,74.44) | 0.48 | 6.13(3.90,9.27) | 6.17(3.98,9.30) | 0.01(-0.01,0.02) |
| Bolivia (Plurinational State of) | both | 404.44(257.63,602.67) | 835.66(538.54,1264.35) | 1.07 | 7.12(4.57,10.67) | 7.10(4.56,10.67) | -0.01(-0.02,-0.01) |
| Bosnia and Herzegovina | both | 340.88(216.29,505.89) | 252.52(162.49,382.08) | -0.26 | 7.09(4.50,10.60) | 7.09(4.52,10.70) | -0.01(-0.03,0.00) |
| Botswana | both | 77.12(48.98,115.69) | 162.27(103.63,237.70) | 1.10 | 6.81(4.32,10.11) | 6.68(4.27,9.78) | -0.05(-0.06,-0.05) |
| Brazil | both | 10223.59(6453.03,15180.39) | 16568.07(10492.02,24457.41) | 0.62 | 7.12(4.48,10.57) | 7.15(4.52,10.59) | 0.01(0.01,0.01) |
| Brunei Darussalam | both | 23.74(14.96,36.23) | 45.27(28.26,66.76) | 0.91 | 9.13(5.79,13.72) | 9.26(5.77,13.68) | 0.05(0.03,0.06) |
| Bulgaria | both | 650.75(409.56,980.73) | 525.83(331.30,783.61) | -0.19 | 7.11(4.50,10.71) | 7.07(4.47,10.56) | -0.02(-0.03,-0.01) |
| Burkina Faso | both | 524.96(329.87,781.18) | 1321.03(828.78,2009.27) | 1.52 | 6.82(4.29,10.16) | 6.83(4.33,10.32) | 0.01(0.00,0.01) |
| Burundi | both | 316.75(199.14,478.52) | 687.70(434.36,1034.55) | 1.17 | 6.74(4.26,10.08) | 6.61(4.24,9.80) | -0.07(-0.08,-0.06) |
| Cabo Verde | both | 20.46(12.90,30.49) | 39.17(24.78,58.31) | 0.91 | 6.97(4.47,10.34) | 6.71(4.23,9.98) | -0.14(-0.15,-0.13) |
| Cambodia | both | 788.63(498.21,1175.26) | 1492.07(945.81,2255.93) | 0.89 | 8.92(5.69,13.24) | 8.85(5.58,13.34) | -0.02(-0.02,-0.02) |
| Cameroon | both | 589.58(375.87,873.55) | 1779.12(1122.84,2702.64) | 2.02 | 6.72(4.33,9.89) | 6.72(4.26,10.23) | 0.00(-0.01,0.00) |
| Canada | both | 3262.49(2068.83,4913.11) | 4308.55(2715.15,6417.31) | 0.32 | 10.96(6.91,16.51) | 10.93(6.91,16.23) | -0.01(-0.02,0.00) |
| Central African Republic | both | 159.28(101.00,239.84) | 318.10(202.47,477.98) | 1.00 | 6.70(4.28,9.96) | 6.74(4.28,10.12) | 0.02(0.01,0.03) |
| Chad | both | 333.19(209.01,501.25) | 882.76(564.70,1313.12) | 1.65 | 6.79(4.27,10.20) | 6.71(4.27,10.01) | -0.05(-0.05,-0.04) |
| Chile | both | 1254.48(786.92,1878.02) | 1836.20(1164.94,2702.56) | 0.46 | 9.41(5.92,14.08) | 9.38(5.98,13.91) | -0.01(-0.01,0.00) |
| China | both | 95739.88(60121.53,142944.05) | 124821.98(78771.44,185268.44) | 0.30 | 7.82(4.91,11.66) | 7.86(4.92,11.71) | 0.03(0.02,0.03) |
| Colombia | both | 2223.21(1403.12,3330.32) | 3580.41(2296.09,5371.94) | 0.61 | 7.12(4.48,10.62) | 7.16(4.59,10.73) | 0.01(0.01,0.02) |
| Comoros | both | 26.34(16.43,40.11) | 46.86(29.55,69.45) | 0.78 | 6.72(4.21,10.00) | 6.68(4.20,9.93) | -0.01(-0.02,-0.01) |
| Congo | both | 140.75(88.82,212.95) | 335.07(208.01,504.60) | 1.38 | 6.73(4.27,10.00) | 6.70(4.19,10.04) | -0.02(-0.03,-0.01) |
| Cook Islands | both | 1.47(0.92,2.20) | 1.50(0.94,2.24) | 0.02 | 8.15(5.13,12.28) | 8.33(5.18,12.50) | 0.09(0.07,0.10) |
| Costa Rica | both | 205.46(130.34,307.82) | 360.57(227.44,537.93) | 0.75 | 7.10(4.53,10.61) | 7.16(4.53,10.72) | 0.04(0.03,0.04) |
| Côte d'Ivoire | both | 688.62(431.86,1041.07) | 1599.25(1018.64,2379.51) | 1.32 | 6.55(4.13,9.80) | 6.61(4.20,9.80) | 0.02(0.02,0.03) |
| Croatia | both | 373.28(234.45,556.70) | 320.24(202.75,470.85) | -0.14 | 7.10(4.47,10.67) | 7.08(4.42,10.53) | -0.01(-0.02,-0.01) |
| Cuba | both | 807.13(508.52,1215.43) | 855.98(535.60,1278.54) | 0.06 | 7.09(4.48,10.70) | 7.05(4.42,10.60) | -0.01(-0.01,-0.01) |
| Cyprus | both | 71.72(46.19,106.04) | 131.26(82.35,196.20) | 0.83 | 8.86(5.70,13.14) | 8.89(5.58,13.22) | 0.01(0.01,0.02) |
| Czechia | both | 768.14(485.65,1138.22) | 798.64(503.14,1198.38) | 0.04 | 7.11(4.49,10.63) | 7.04(4.50,10.56) | -0.04(-0.04,-0.03) |
| Democratic People's Republic of Korea | both | 1668.57(1054.00,2498.69) | 2239.21(1433.42,3362.01) | 0.34 | 8.04(5.10,12.00) | 7.82(4.99,11.76) | -0.11(-0.12,-0.10) |
| Democratic Republic of the Congo | both | 2162.16(1375.02,3245.69) | 5174.10(3267.94,7683.13) | 1.39 | 6.68(4.24,10.09) | 6.67(4.29,9.87) | 0.00(-0.01,0.00) |
| Denmark | both | 490.93(307.24,729.32) | 531.16(338.13,795.10) | 0.08 | 8.85(5.55,13.10) | 8.80(5.59,13.26) | -0.01(-0.01,0.00) |
| Djibouti | both | 27.41(17.21,41.80) | 78.64(49.44,117.72) | 1.87 | 6.52(4.13,9.86) | 6.57(4.13,9.87) | 0.03(0.02,0.04) |
| Dominica | both | 4.93(3.10,7.40) | 5.03(3.21,7.57) | 0.02 | 7.10(4.49,10.74) | 7.03(4.46,10.56) | -0.03(-0.04,-0.02) |
| Dominican Republic | both | 478.17(303.00,715.45) | 785.50(500.46,1167.66) | 0.64 | 7.15(4.52,10.74) | 7.08(4.51,10.49) | -0.05(-0.05,-0.04) |
| Ecuador | both | 655.26(414.46,986.61) | 1262.02(799.96,1886.91) | 0.93 | 7.10(4.46,10.73) | 7.12(4.52,10.66) | 0.00(0.00,0.01) |
| Egypt | both | 3204.75(2023.44,4795.74) | 6209.41(3990.06,9320.73) | 0.94 | 6.31(3.97,9.38) | 6.31(4.04,9.44) | -0.01(-0.02,0.00) |
| El Salvador | both | 337.11(215.38,500.36) | 458.63(287.84,678.75) | 0.36 | 7.16(4.55,10.57) | 7.27(4.55,10.74) | 0.06(0.05,0.07) |
| Equatorial Guinea | both | 24.07(15.28,36.10) | 85.92(53.91,129.52) | 2.57 | 6.81(4.36,10.19) | 6.64(4.23,9.94) | -0.10(-0.13,-0.08) |
| Eritrea | both | 169.24(104.85,254.56) | 415.87(261.94,624.88) | 1.46 | 6.69(4.17,10.07) | 6.69(4.21,10.01) | 0.00(-0.01,0.01) |
| Estonia | both | 117.98(75.07,175.97) | 99.26(62.67,146.50) | -0.16 | 7.17(4.55,10.70) | 7.11(4.54,10.57) | -0.03(-0.04,-0.03) |
| Eswatini | both | 45.90(28.76,69.13) | 74.34(46.80,110.45) | 0.62 | 6.86(4.28,10.19) | 6.74(4.25,10.04) | -0.08(-0.09,-0.07) |
| Ethiopia | both | 2872.68(1805.65,4248.12) | 6421.60(4064.56,9685.01) | 1.24 | 6.71(4.23,9.88) | 6.72(4.26,10.02) | 0.02(0.01,0.03) |
| Fiji | both | 59.58(37.41,89.01) | 75.55(48.14,112.86) | 0.27 | 8.18(5.17,12.17) | 8.16(5.21,12.18) | -0.01(-0.02,-0.01) |
| Finland | both | 483.71(310.25,718.36) | 506.94(321.97,757.51) | 0.05 | 8.91(5.69,13.27) | 8.80(5.56,13.17) | -0.03(-0.04,-0.02) |
| France | both | 5437.75(3464.01,8196.15) | 5992.46(3780.08,9012.63) | 0.10 | 8.89(5.66,13.39) | 8.86(5.60,13.43) | 0.00(-0.01,0.00) |
| Gabon | both | 58.25(36.88,87.98) | 115.98(73.30,173.09) | 0.99 | 6.68(4.21,10.04) | 6.74(4.26,10.06) | 0.04(0.04,0.05) |
| Gambia | both | 55.88(35.38,84.03) | 136.97(87.30,207.39) | 1.45 | 6.64(4.22,9.94) | 6.73(4.36,10.05) | 0.05(0.04,0.06) |
| Georgia | both | 412.49(261.08,621.33) | 273.74(173.28,410.25) | -0.34 | 7.22(4.57,10.86) | 7.12(4.51,10.71) | -0.27(-2.27,1.78) |
| Germany | both | 7831.86(4940.49,11719.87) | 7892.68(4932.27,11746.68) | 0.01 | 8.91(5.64,13.34) | 8.82(5.53,13.20) | -0.03(-0.04,-0.03) |
| Ghana | both | 877.01(557.89,1320.42) | 2071.22(1293.21,3112.83) | 1.36 | 6.74(4.32,10.09) | 6.82(4.29,10.13) | 0.04(0.04,0.05) |
| Greece | both | 972.89(608.65,1468.32) | 958.12(616.24,1426.35) | -0.02 | 8.87(5.56,13.45) | 8.85(5.68,13.25) | -0.01(-0.01,0.00) |
| Greenland | both | 6.24(3.92,9.19) | 6.33(3.95,9.38) | 0.02 | 10.35(6.49,15.31) | 10.51(6.60,15.60) | 0.04(0.03,0.04) |
| Grenada | both | 5.55(3.54,8.32) | 7.65(4.83,11.42) | 0.38 | 7.13(4.52,10.65) | 7.01(4.41,10.50) | -0.05(-0.06,-0.05) |
| Guam | both | 11.39(7.22,17.17) | 13.92(8.90,20.64) | 0.22 | 8.08(5.09,12.20) | 8.17(5.18,12.16) | 0.04(0.01,0.06) |
| Guatemala | both | 480.04(304.71,721.34) | 1254.78(790.98,1875.26) | 1.61 | 7.09(4.55,10.55) | 7.18(4.54,10.83) | 0.05(0.04,0.06) |
| Guinea | both | 354.03(224.44,531.50) | 742.53(467.86,1109.55) | 1.10 | 6.77(4.32,10.13) | 6.81(4.32,10.17) | 0.03(0.02,0.03) |
| Guinea-Bissau | both | 57.32(36.28,86.13) | 117.69(73.84,177.97) | 1.05 | 6.80(4.35,10.20) | 6.81(4.32,10.23) | 0.00(-0.01,0.01) |
| Guyana | both | 51.53(32.83,76.54) | 55.75(35.08,83.29) | 0.08 | 7.06(4.52,10.49) | 7.07(4.50,10.53) | 0.01(0.00,0.02) |
| Haiti | both | 402.11(254.69,602.92) | 868.62(556.14,1291.01) | 1.16 | 7.11(4.51,10.64) | 7.13(4.59,10.60) | 0.01(0.01,0.02) |
| Honduras | both | 281.84(174.29,421.31) | 685.09(432.14,1027.40) | 1.43 | 7.13(4.45,10.67) | 7.19(4.51,10.76) | 0.03(0.03,0.04) |
| Hungary | both | 783.29(503.92,1164.41) | 739.73(469.35,1098.51) | -0.06 | 7.14(4.56,10.66) | 7.11(4.49,10.67) | 0.00(-0.01,0.00) |
| Iceland | both | 23.23(14.72,34.98) | 31.93(20.28,47.46) | 0.37 | 8.82(5.58,13.21) | 8.82(5.59,13.19) | -0.01(-0.01,0.00) |
| India | both | 49106.45(31097.01,72596.61) | 89471.34(56475.19,132428.68) | 0.82 | 6.15(3.90,9.07) | 6.21(3.93,9.18) | 0.04(0.04,0.05) |
| Indonesia | both | 15608.43(9847.92,23212.39) | 24244.98(15155.09,36201.38) | 0.55 | 8.85(5.57,13.13) | 8.84(5.53,13.18) | 0.00(0.00,0.00) |
| Iran (Islamic Republic of) | both | 3239.25(2026.90,4841.16) | 5866.80(3705.23,8684.51) | 0.81 | 6.33(4.00,9.44) | 6.36(4.00,9.39) | 0.02(0.01,0.02) |
| Iraq | both | 979.80(619.37,1449.37) | 2751.36(1729.80,4098.32) | 1.81 | 6.56(4.18,9.76) | 6.57(4.15,9.76) | 0.01(0.01,0.02) |
| Ireland | both | 317.37(199.34,475.65) | 450.13(284.20,665.85) | 0.42 | 8.83(5.55,13.17) | 8.82(5.55,13.08) | 0.00(-0.01,0.00) |
| Israel | both | 429.52(271.98,644.58) | 813.05(516.91,1231.52) | 0.89 | 8.88(5.65,13.34) | 8.87(5.63,13.51) | -0.01(-0.02,-0.01) |
| Italy | both | 5475.71(3444.54,8143.86) | 5604.20(3531.58,8310.64) | 0.02 | 8.91(5.59,13.31) | 8.90(5.60,13.26) | 0.00(0.00,0.00) |
| Jamaica | both | 159.08(98.68,239.63) | 212.60(135.84,320.39) | 0.34 | 7.17(4.50,10.75) | 7.11(4.54,10.66) | -0.02(-0.03,-0.02) |
| Japan | both | 12802.91(8067.80,19149.63) | 12514.64(7870.62,18507.08) | -0.02 | 9.45(5.90,14.09) | 9.39(5.87,13.88) | -0.02(-0.02,-0.02) |
| Jordan | both | 202.66(127.20,308.85) | 722.80(456.72,1092.71) | 2.57 | 6.24(3.97,9.44) | 6.20(3.90,9.36) | -0.02(-0.04,0.00) |
| Kazakhstan | both | 1166.74(742.80,1731.75) | 1364.50(860.56,2035.56) | 0.17 | 7.16(4.61,10.63) | 7.16(4.52,10.65) | -0.01(-0.01,0.00) |
| Kenya | both | 1290.31(810.22,1924.20) | 3176.15(2000.56,4730.50) | 1.46 | 6.72(4.22,9.98) | 6.74(4.25,10.04) | 0.01(0.01,0.01) |
| Kiribati | both | 5.65(3.59,8.41) | 9.55(6.08,14.18) | 0.69 | 8.25(5.25,12.36) | 8.31(5.28,12.40) | 0.01(0.01,0.02) |
| Kuwait | both | 112.18(70.60,167.60) | 329.61(205.58,495.93) | 1.94 | 5.96(3.78,8.97) | 6.22(3.91,9.42) | 0.15(0.11,0.19) |
| Kyrgyzstan | both | 297.42(187.29,453.78) | 467.07(295.57,701.46) | 0.57 | 7.16(4.55,10.83) | 7.15(4.55,10.74) | 0.00(-0.01,0.00) |
| Lao People's Democratic Republic | both | 319.11(204.40,473.67) | 636.60(401.53,950.42) | 0.99 | 8.85(5.66,13.14) | 8.82(5.57,13.21) | -0.01(-0.01,-0.01) |
| Latvia | both | 200.21(126.66,298.68) | 144.76(94.10,215.57) | -0.28 | 7.16(4.52,10.74) | 7.13(4.57,10.69) | -0.01(-0.02,0.00) |
| Lebanon | both | 190.29(119.28,288.57) | 346.14(219.96,515.16) | 0.82 | 6.37(4.00,9.66) | 6.40(4.05,9.48) | 0.02(0.01,0.03) |
| Lesotho | both | 107.36(67.77,163.56) | 140.15(89.84,211.10) | 0.31 | 6.76(4.30,10.07) | 6.66(4.32,9.98) | -0.05(-0.05,-0.04) |
| Liberia | both | 110.48(69.54,166.77) | 297.28(188.39,443.22) | 1.69 | 6.66(4.16,10.01) | 6.64(4.23,9.89) | -0.02(-0.03,-0.01) |
| Libya | both | 226.49(144.20,334.94) | 468.14(296.63,696.78) | 1.07 | 6.16(3.93,9.12) | 6.26(3.96,9.32) | 0.03(0.01,0.05) |
| Lithuania | both | 275.21(173.60,407.10) | 211.28(135.16,316.54) | -0.23 | 7.15(4.52,10.61) | 7.14(4.57,10.73) | -0.01(-0.02,-0.01) |
| Luxembourg | both | 37.37(23.54,56.00) | 59.23(36.86,88.17) | 0.58 | 8.86(5.60,13.27) | 8.78(5.44,13.13) | -0.03(-0.03,-0.02) |
| Madagascar | both | 676.78(431.59,1006.97) | 1625.21(1000.38,2482.20) | 1.40 | 6.66(4.27,9.92) | 6.69(4.18,10.13) | 0.02(0.02,0.03) |
| Malawi | both | 541.17(344.66,810.73) | 1101.75(695.47,1669.39) | 1.04 | 6.68(4.18,9.97) | 6.74(4.27,10.14) | 0.05(0.04,0.06) |
| Malaysia | both | 1476.46(922.69,2225.47) | 2923.00(1852.90,4370.77) | 0.98 | 8.79(5.49,13.14) | 8.74(5.56,13.11) | -0.02(-0.03,-0.02) |
| Maldives | both | 16.19(10.28,24.19) | 48.98(31.03,73.57) | 2.02 | 8.72(5.50,12.96) | 8.46(5.40,12.70) | -0.09(-0.13,-0.06) |
| Mali | both | 484.44(301.78,725.62) | 1228.98(756.29,1864.90) | 1.54 | 6.74(4.22,10.09) | 6.73(4.23,10.16) | 0.00(-0.01,0.01) |
| Malta | both | 34.70(22.15,51.70) | 41.53(26.96,62.05) | 0.20 | 8.89(5.65,13.27) | 8.82(5.67,13.11) | -0.04(-0.04,-0.03) |
| Marshall Islands | both | 3.14(1.99,4.66) | 4.63(2.91,6.93) | 0.47 | 8.17(5.19,12.07) | 8.12(5.13,12.12) | -0.02(-0.02,-0.01) |
| Mauritania | both | 118.85(77.41,177.44) | 246.79(156.62,371.42) | 1.08 | 6.74(4.40,10.04) | 6.79(4.33,10.18) | 0.01(0.01,0.02) |
| Mauritius | both | 99.85(63.27,149.75) | 122.03(77.69,181.38) | 0.22 | 8.80(5.58,13.11) | 8.78(5.55,13.20) | -0.01(-0.01,0.00) |
| Mexico | both | 5612.50(3529.07,8339.95) | 9276.45(5841.17,13690.55) | 0.65 | 7.17(4.53,10.61) | 7.18(4.51,10.58) | 0.00(-0.01,0.00) |
| Micronesia (Federated States of) | both | 7.36(4.58,10.99) | 8.27(5.20,12.64) | 0.12 | 8.17(5.09,12.12) | 8.18(5.17,12.53) | 0.00(-0.01,0.01) |
| Monaco | both | 2.94(1.86,4.40) | 3.39(2.15,5.03) | 0.15 | 8.91(5.66,13.30) | 8.85(5.62,13.20) | -0.02(-0.02,-0.01) |
| Mongolia | both | 136.86(87.62,203.24) | 251.88(157.43,377.63) | 0.84 | 7.11(4.51,10.54) | 7.15(4.46,10.70) | 0.02(0.02,0.03) |
| Montenegro | both | 45.93(29.50,67.96) | 46.93(29.61,70.18) | 0.02 | 7.11(4.57,10.52) | 7.08(4.42,10.66) | 0.00(-0.01,0.00) |
| Morocco | both | 1489.93(917.69,2227.29) | 2373.31(1472.88,3566.07) | 0.59 | 6.37(3.94,9.46) | 6.34(3.93,9.49) | -0.01(-0.02,0.00) |
| Mozambique | both | 741.39(471.46,1116.80) | 1690.52(1072.96,2555.53) | 1.28 | 6.75(4.29,10.11) | 6.75(4.28,10.15) | -0.01(-0.01,0.00) |
| Myanmar | both | 3366.95(2135.74,5018.10) | 4990.40(3146.02,7448.82) | 0.48 | 8.81(5.58,13.10) | 8.90(5.62,13.24) | 0.04(0.03,0.04) |
| Namibia | both | 83.53(53.52,124.97) | 156.95(99.86,234.03) | 0.88 | 6.74(4.31,10.08) | 6.74(4.29,10.09) | 0.00(-0.01,0.01) |
| Nauru | both | 0.75(0.48,1.14) | 0.84(0.53,1.27) | 0.11 | 8.18(5.19,12.32) | 8.25(5.22,12.48) | 0.04(0.03,0.05) |
| Nepal | both | 1074.47(672.26,1603.78) | 1947.39(1218.18,2912.33) | 0.81 | 6.26(3.94,9.35) | 6.43(4.02,9.60) | 0.10(0.09,0.12) |
| Netherlands | both | 1451.09(924.17,2191.14) | 1581.88(1010.01,2372.56) | 0.09 | 8.89(5.63,13.40) | 8.83(5.60,13.36) | -0.01(-0.02,-0.01) |
| New Zealand | both | 334.02(211.52,495.88) | 434.83(277.17,641.08) | 0.30 | 9.33(5.90,13.82) | 9.40(5.99,13.89) | 0.03(0.03,0.04) |
| Nicaragua | both | 232.61(148.15,346.46) | 468.03(298.08,697.92) | 1.01 | 7.13(4.55,10.63) | 7.13(4.54,10.58) | 0.00(-0.01,0.00) |
| Niger | both | 435.75(272.99,649.34) | 1236.76(783.83,1863.57) | 1.84 | 6.70(4.27,9.96) | 6.75(4.28,10.01) | 0.04(0.03,0.04) |
| Nigeria | both | 5208.02(3307.42,7817.90) | 12839.46(8054.39,19213.87) | 1.47 | 6.61(4.20,9.84) | 6.83(4.33,10.27) | 0.12(0.11,0.14) |
| Niue | both | 0.17(0.11,0.26) | 0.14(0.09,0.21) | -0.21 | 8.22(5.14,12.25) | 8.21(5.17,12.17) | -0.01(-0.02,0.00) |
| North Macedonia | both | 147.21(93.04,220.61) | 167.34(106.38,248.16) | 0.14 | 7.07(4.45,10.60) | 7.03(4.52,10.44) | -0.02(-0.03,-0.02) |
| Northern Mariana Islands | both | 4.05(2.59,6.09) | 3.62(2.30,5.32) | -0.11 | 8.05(5.10,12.03) | 8.14(5.11,12.06) | 0.01(-0.03,0.05) |
| Norway | both | 399.82(251.73,591.64) | 496.35(313.32,735.13) | 0.24 | 8.87(5.56,13.20) | 8.82(5.56,13.08) | -0.01(-0.02,-0.01) |
| Oman | both | 106.28(66.51,158.72) | 313.74(198.14,473.47) | 1.95 | 5.88(3.70,8.82) | 5.79(3.67,8.69) | -0.09(-0.16,-0.02) |
| Pakistan | both | 5928.97(3725.18,8771.21) | 12956.36(8160.23,19130.43) | 1.19 | 6.17(3.92,9.03) | 6.25(3.96,9.25) | 0.04(0.04,0.05) |
| Palau | both | 1.27(0.82,1.93) | 1.55(1.00,2.33) | 0.22 | 8.15(5.19,12.31) | 7.91(5.10,11.91) | -0.10(-0.13,-0.06) |
| Palestine | both | 108.13(67.92,161.78) | 295.93(186.28,447.26) | 1.74 | 6.39(4.05,9.43) | 6.30(4.02,9.42) | -0.03(-0.04,-0.02) |
| Panama | both | 161.89(102.73,242.07) | 296.24(186.54,448.65) | 0.83 | 7.07(4.51,10.54) | 7.07(4.45,10.70) | 0.01(0.01,0.02) |
| Papua New Guinea | both | 300.43(191.44,451.74) | 765.87(477.14,1138.45) | 1.55 | 8.09(5.11,12.05) | 8.13(5.06,12.02) | 0.01(0.01,0.01) |
| Paraguay | both | 256.79(162.56,383.07) | 499.98(315.81,745.92) | 0.95 | 7.07(4.50,10.46) | 7.05(4.44,10.56) | -0.01(-0.01,0.00) |
| Peru | both | 1420.91(913.89,2150.93) | 2475.35(1566.18,3671.99) | 0.74 | 7.11(4.58,10.71) | 7.10(4.50,10.53) | 0.00(0.00,0.01) |
| Philippines | both | 5100.01(3194.81,7621.91) | 9817.22(6157.15,14692.90) | 0.92 | 8.82(5.54,13.09) | 8.83(5.54,13.23) | 0.01(0.01,0.01) |
| Poland | both | 2838.35(1786.76,4203.31) | 2996.78(1886.64,4431.91) | 0.06 | 7.14(4.51,10.58) | 7.14(4.48,10.58) | 0.00(0.00,0.00) |
| Portugal | both | 942.44(593.17,1404.21) | 1002.02(633.82,1507.34) | 0.06 | 8.88(5.58,13.29) | 8.89(5.53,13.43) | 0.00(0.00,0.00) |
| Puerto Rico | both | 260.74(165.85,395.36) | 264.88(169.42,392.17) | 0.02 | 7.17(4.55,10.87) | 7.15(4.56,10.61) | -0.02(-0.02,-0.01) |
| Qatar | both | 28.25(17.87,42.67) | 198.96(125.41,295.82) | 6.04 | 5.60(3.52,8.38) | 5.39(3.41,8.06) | -0.21(-0.26,-0.16) |
| Republic of Korea | both | 4416.18(2774.47,6569.25) | 5551.74(3585.22,8193.82) | 0.26 | 9.41(5.89,14.06) | 9.28(5.92,13.83) | -0.04(-0.05,-0.03) |
| Republic of Moldova | both | 328.11(209.68,490.06) | 292.39(186.01,437.68) | -0.11 | 7.18(4.57,10.68) | 7.13(4.53,10.68) | -0.03(-0.03,-0.03) |
| Romania | both | 1710.03(1103.39,2537.52) | 1447.97(921.71,2139.67) | -0.15 | 7.11(4.53,10.56) | 7.09(4.53,10.50) | -0.01(-0.01,0.00) |
| Russian Federation | both | 11536.92(7351.43,17106.82) | 11379.03(7195.83,16660.21) | -0.01 | 7.19(4.60,10.71) | 7.19(4.52,10.70) | 0.01(0.00,0.01) |
| Rwanda | both | 406.78(256.26,604.27) | 801.24(506.77,1189.40) | 0.97 | 6.74(4.23,10.05) | 6.74(4.29,9.98) | 0.02(0.01,0.03) |
| Saint Kitts and Nevis | both | 2.81(1.76,4.21) | 4.56(2.91,6.80) | 0.62 | 7.14(4.46,10.67) | 7.06(4.46,10.52) | -0.03(-0.04,-0.02) |
| Saint Lucia | both | 8.99(5.64,13.30) | 13.42(8.43,19.84) | 0.49 | 7.16(4.51,10.55) | 7.06(4.45,10.43) | -0.04(-0.05,-0.04) |
| Saint Vincent and the Grenadines | both | 7.17(4.51,10.63) | 8.29(5.23,12.45) | 0.16 | 7.10(4.45,10.58) | 7.02(4.42,10.54) | -0.03(-0.04,-0.03) |
| Samoa | both | 11.91(7.43,17.74) | 16.29(10.41,24.63) | 0.37 | 8.15(5.10,12.05) | 8.17(5.24,12.33) | 0.00(0.00,0.01) |
| San Marino | both | 2.21(1.40,3.27) | 3.06(1.93,4.62) | 0.39 | 8.83(5.58,13.10) | 8.86(5.58,13.45) | 0.02(0.01,0.02) |
| Sao Tome and Principe | both | 6.77(4.36,10.07) | 13.17(8.26,19.63) | 0.95 | 6.79(4.38,10.19) | 6.71(4.22,9.97) | -0.04(-0.05,-0.04) |
| Saudi Arabia | both | 888.42(547.32,1333.25) | 2544.09(1611.43,3836.18) | 1.86 | 5.99(3.74,8.89) | 6.00(3.79,9.08) | 0.00(-0.02,0.01) |
| Senegal | both | 425.52(265.99,637.73) | 921.66(584.92,1384.57) | 1.17 | 6.73(4.26,10.06) | 6.73(4.30,10.08) | -0.01(-0.02,0.00) |
| Serbia | both | 701.09(444.50,1057.18) | 656.44(419.19,986.57) | -0.06 | 7.10(4.50,10.67) | 7.06(4.46,10.72) | -0.01(-0.02,-0.01) |
| Seychelles | both | 6.26(3.94,9.38) | 9.58(6.06,14.32) | 0.53 | 8.81(5.56,13.26) | 8.69(5.46,12.95) | -0.06(-0.07,-0.05) |
| Sierra Leone | both | 214.96(137.34,323.81) | 509.09(320.66,763.24) | 1.37 | 6.72(4.29,10.11) | 6.71(4.24,10.10) | -0.03(-0.04,-0.02) |
| Singapore | both | 319.22(202.13,475.61) | 609.80(385.67,910.12) | 0.91 | 9.36(5.93,13.91) | 9.27(5.87,13.76) | -0.05(-0.06,-0.04) |
| Slovakia | both | 389.75(245.92,573.91) | 422.48(269.78,625.80) | 0.08 | 7.11(4.51,10.46) | 7.08(4.53,10.55) | -0.02(-0.03,-0.01) |
| Slovenia | both | 149.79(95.74,221.74) | 154.43(96.52,230.03) | 0.03 | 7.10(4.53,10.58) | 7.03(4.39,10.63) | -0.04(-0.05,-0.04) |
| Solomon Islands | both | 23.28(14.60,34.27) | 49.91(31.75,75.14) | 1.14 | 8.10(5.08,12.00) | 8.21(5.20,12.28) | 0.03(0.03,0.04) |
| Somalia | both | 393.19(249.90,594.51) | 1143.70(723.26,1728.05) | 1.91 | 6.64(4.21,10.03) | 6.66(4.19,10.01) | 0.01(0.00,0.01) |
| South Africa | both | 2385.01(1502.53,3584.40) | 3927.54(2493.11,5898.90) | 0.65 | 6.80(4.27,10.22) | 6.72(4.26,10.11) | -0.05(-0.05,-0.04) |
| South Sudan | both | 328.69(211.61,493.90) | 525.52(331.89,790.51) | 0.60 | 6.45(4.15,9.64) | 6.67(4.24,10.01) | 0.12(0.11,0.13) |
| Spain | both | 3632.81(2314.02,5392.13) | 4314.26(2769.06,6387.47) | 0.19 | 8.90(5.66,13.27) | 8.86(5.63,13.21) | -0.02(-0.03,-0.01) |
| Sri Lanka | both | 1527.61(954.57,2278.16) | 2016.50(1277.61,2990.51) | 0.32 | 8.90(5.58,13.26) | 8.86(5.59,13.17) | 0.02(0.01,0.03) |
| Sudan | both | 1099.21(702.05,1661.88) | 2424.14(1528.17,3665.74) | 1.21 | 6.35(4.02,9.43) | 6.34(4.00,9.51) | 0.00(-0.01,0.00) |
| Suriname | both | 26.14(16.50,38.61) | 41.79(26.63,62.66) | 0.60 | 7.05(4.49,10.42) | 7.08(4.50,10.64) | 0.01(0.01,0.02) |
| Sweden | both | 805.16(504.41,1201.32) | 936.25(593.42,1369.88) | 0.16 | 8.90(5.59,13.33) | 8.85(5.61,12.97) | -0.01(-0.02,-0.01) |
| Switzerland | both | 667.15(420.25,992.97) | 827.69(523.52,1223.08) | 0.24 | 8.84(5.55,13.23) | 8.82(5.51,13.04) | -0.01(-0.02,-0.01) |
| Syrian Arab Republic | both | 680.61(426.52,1018.63) | 908.49(580.23,1372.36) | 0.33 | 6.31(3.97,9.29) | 6.46(4.14,9.80) | 0.07(0.05,0.10) |
| Taiwan (Province of China) | both | 1677.81(1046.58,2554.27) | 2066.56(1317.07,3106.29) | 0.23 | 7.77(4.85,11.84) | 7.85(4.94,11.74) | 0.03(0.03,0.04) |
| Tajikistan | both | 335.65(210.71,511.40) | 665.14(420.12,1008.17) | 0.98 | 7.15(4.50,10.77) | 7.10(4.51,10.71) | -0.02(-0.02,-0.01) |
| Thailand | both | 5145.75(3270.53,7731.43) | 6844.67(4307.33,10164.39) | 0.33 | 8.85(5.62,13.33) | 8.87(5.55,13.25) | 0.01(0.00,0.01) |
| Timor-Leste | both | 61.63(38.87,93.35) | 106.99(68.01,160.19) | 0.74 | 8.72(5.52,13.01) | 8.80(5.58,13.07) | 0.04(0.03,0.06) |
| Togo | both | 206.39(128.11,306.39) | 496.55(321.63,737.44) | 1.41 | 6.80(4.30,10.11) | 6.80(4.39,10.09) | 0.02(0.01,0.02) |
| Tokelau | both | 0.12(0.08,0.18) | 0.11(0.07,0.16) | -0.10 | 8.33(5.27,12.56) | 8.17(5.17,12.12) | -0.08(-0.10,-0.07) |
| Tonga | both | 7.02(4.40,10.56) | 7.92(5.02,11.85) | 0.13 | 8.30(5.26,12.49) | 8.30(5.24,12.40) | 0.01(0.00,0.02) |
| Trinidad and Tobago | both | 83.59(52.97,126.39) | 105.60(66.94,158.78) | 0.26 | 7.08(4.50,10.62) | 7.05(4.47,10.64) | -0.01(-0.01,0.00) |
| Tunisia | both | 499.84(314.42,754.46) | 788.75(492.81,1165.69) | 0.58 | 6.37(3.98,9.54) | 6.39(4.02,9.44) | 0.02(0.02,0.03) |
| Turkey | both | 3475.84(2192.06,5208.92) | 5492.45(3514.36,8217.89) | 0.58 | 6.15(3.87,9.20) | 6.14(3.95,9.16) | -0.01(-0.01,0.00) |
| Turkmenistan | both | 240.32(151.37,359.39) | 364.63(227.44,545.41) | 0.52 | 7.15(4.51,10.75) | 7.06(4.40,10.55) | -0.06(-0.07,-0.05) |
| Tuvalu | both | 0.75(0.47,1.13) | 0.96(0.60,1.44) | 0.28 | 8.42(5.32,12.71) | 8.13(5.12,12.20) | -0.13(-0.14,-0.13) |
| Uganda | both | 943.44(589.39,1422.44) | 2352.39(1490.42,3588.00) | 1.49 | 6.65(4.22,9.97) | 6.77(4.34,10.18) | 0.07(0.06,0.07) |
| Ukraine | both | 4003.97(2523.53,6062.30) | 3490.42(2237.73,5152.22) | -0.13 | 7.22(4.53,10.97) | 7.21(4.58,10.70) | 0.00(-0.01,0.01) |
| United Arab Emirates | both | 115.93(72.91,173.08) | 654.86(406.00,972.95) | 4.65 | 5.65(3.57,8.45) | 5.61(3.47,8.35) | -0.09(-0.12,-0.05) |
| United Kingdom | both | 5402.30(3408.44,7978.28) | 6227.66(3927.54,9210.75) | 0.15 | 8.86(5.57,13.12) | 8.83(5.58,13.09) | -0.01(-0.01,-0.01) |
| United Republic of Tanzania | both | 1449.91(908.65,2181.86) | 3380.89(2139.66,5146.86) | 1.33 | 6.69(4.21,9.99) | 6.76(4.30,10.23) | 0.04(0.03,0.06) |
| United States of America | both | 32115.43(20290.90,47642.37) | 40153.78(25431.07,59137.75) | 0.25 | 11.85(7.47,17.63) | 11.48(7.25,16.94) | -0.29(-0.39,-0.19) |
| United States Virgin Islands | both | 7.63(4.86,11.15) | 7.66(4.83,11.25) | 0.00 | 7.21(4.60,10.58) | 7.20(4.52,10.60) | 0.00(-0.01,0.00) |
| Uruguay | both | 298.09(189.44,442.25) | 337.83(214.68,502.04) | 0.13 | 9.44(6.02,13.98) | 9.44(5.98,14.11) | 0.00(0.00,0.01) |
| Uzbekistan | both | 1345.78(849.74,2019.19) | 2454.97(1542.11,3745.11) | 0.82 | 7.13(4.51,10.68) | 7.12(4.48,10.87) | 0.00(0.00,0.01) |
| Vanuatu | both | 10.82(6.71,16.31) | 22.74(14.37,34.77) | 1.10 | 8.13(5.03,12.18) | 8.19(5.20,12.41) | 0.02(0.02,0.03) |
| Venezuela (Bolivarian Republic of) | both | 1262.41(816.51,1869.14) | 2081.49(1312.73,3140.81) | 0.65 | 7.12(4.56,10.57) | 7.15(4.51,10.81) | 0.00(-0.01,0.01) |
| Viet Nam | both | 5600.34(3580.65,8405.13) | 9353.68(5892.30,13947.81) | 0.67 | 8.93(5.71,13.33) | 8.88(5.60,13.25) | -0.02(-0.03,-0.02) |
| Yemen | both | 689.23(436.10,1026.68) | 1826.57(1180.19,2729.86) | 1.65 | 6.31(4.02,9.34) | 6.30(4.08,9.33) | 0.00(0.00,0.01) |
| Zambia | both | 439.22(282.46,660.34) | 1094.61(693.92,1635.49) | 1.49 | 6.67(4.31,9.95) | 6.67(4.22,9.87) | 0.00(-0.01,0.00) |
| Zimbabwe | both | 586.85(376.09,888.96) | 937.99(590.80,1389.69) | 0.60 | 6.75(4.31,10.14) | 6.80(4.30,9.98) | 0.04(0.03,0.04) |
| Afghanistan | male | 198.18(122.88,305.20) | 747.51(459.69,1130.80) | 2.77 | 4.37(2.73,6.73) | 4.38(2.72,6.56) | 0.03(0.02,0.04) |
| Albania | male | 81.09(51.48,123.64) | 70.37(44.24,107.00) | -0.13 | 4.88(3.10,7.39) | 4.88(3.03,7.44) | 0.02(0.00,0.03) |
| Algeria | male | 503.50(313.30,764.02) | 981.92(609.06,1476.43) | 0.95 | 4.45(2.77,6.62) | 4.45(2.78,6.69) | 0.00(-0.01,0.00) |
| American Samoa | male | 1.35(0.84,2.06) | 1.58(0.99,2.41) | 0.17 | 5.90(3.69,8.87) | 5.89(3.71,8.96) | 0.00(-0.01,0.01) |
| Andorra | male | 2.51(1.59,3.77) | 3.18(2.03,4.76) | 0.26 | 7.44(4.70,11.13) | 7.43(4.73,11.04) | -0.01(-0.02,0.00) |
| Angola | male | 208.93(130.34,325.70) | 577.30(355.40,881.75) | 1.76 | 4.66(2.91,7.17) | 4.68(2.89,7.22) | 0.02(0.01,0.03) |
| Antigua and Barbuda | male | 1.41(0.86,2.11) | 2.29(1.42,3.45) | 0.62 | 4.89(2.98,7.28) | 4.88(3.03,7.40) | -0.01(-0.02,0.00) |
| Argentina | male | 1101.76(699.69,1676.34) | 1572.20(992.35,2360.70) | 0.43 | 6.92(4.40,10.54) | 6.92(4.36,10.38) | 0.00(-0.01,0.00) |
| Armenia | male | 81.76(51.18,123.72) | 76.60(46.64,116.56) | -0.06 | 4.87(3.08,7.31) | 4.90(2.98,7.44) | 0.03(0.02,0.03) |
| Australia | male | 609.20(379.81,924.32) | 825.92(518.62,1245.39) | 0.36 | 6.88(4.31,10.43) | 6.88(4.30,10.35) | 0.00(0.00,0.01) |
| Austria | male | 299.97(186.02,452.34) | 323.85(205.76,484.91) | 0.08 | 7.43(4.62,11.18) | 7.44(4.70,11.22) | 0.01(0.00,0.01) |
| Azerbaijan | male | 170.12(106.00,260.43) | 274.03(170.58,416.09) | 0.61 | 4.91(3.08,7.58) | 4.89(3.07,7.43) | 0.00(-0.01,0.01) |
| Bahamas | male | 6.18(3.80,9.56) | 9.58(5.99,14.50) | 0.55 | 4.90(3.07,7.53) | 4.89(3.07,7.38) | 0.00(-0.01,0.01) |
| Bahrain | male | 14.82(9.24,22.06) | 49.38(30.55,76.58) | 2.33 | 4.43(2.77,6.54) | 4.46(2.80,6.78) | 0.01(0.00,0.01) |
| Bangladesh | male | 2137.83(1325.34,3214.88) | 3505.01(2176.29,5430.46) | 0.64 | 4.36(2.73,6.52) | 4.38(2.71,6.83) | 0.02(0.01,0.03) |
| Barbados | male | 6.18(3.87,9.49) | 7.45(4.73,11.33) | 0.20 | 4.89(3.05,7.50) | 4.89(3.11,7.39) | -0.01(-0.02,-0.01) |
| Belarus | male | 253.35(160.17,383.82) | 236.24(149.55,354.52) | -0.07 | 4.88(3.07,7.36) | 4.88(3.07,7.36) | 0.01(0.00,0.02) |
| Belgium | male | 382.37(239.79,579.36) | 399.75(250.43,603.16) | 0.05 | 7.44(4.66,11.28) | 7.41(4.65,11.26) | -0.01(-0.02,-0.01) |
| Belize | male | 4.01(2.48,6.04) | 9.95(6.17,15.20) | 1.48 | 4.89(3.08,7.35) | 4.87(3.04,7.44) | -0.01(-0.01,0.00) |
| Benin | male | 86.17(54.08,130.39) | 248.70(155.07,381.56) | 1.89 | 4.67(2.95,7.12) | 4.70(2.96,7.19) | 0.02(0.01,0.03) |
| Bermuda | male | 1.58(0.99,2.43) | 1.63(1.03,2.45) | 0.03 | 4.89(3.07,7.49) | 4.90(3.09,7.40) | 0.00(-0.01,0.00) |
| Bhutan | male | 12.81(7.90,19.56) | 18.58(11.57,27.96) | 0.45 | 4.37(2.71,6.61) | 4.38(2.74,6.55) | 0.02(0.01,0.03) |
| Bolivia (Plurinational State of) | male | 135.43(85.13,204.27) | 287.16(183.17,431.01) | 1.12 | 4.87(3.06,7.33) | 4.88(3.12,7.33) | 0.01(0.01,0.02) |
| Bosnia and Herzegovina | male | 117.98(73.90,180.50) | 84.52(53.67,127.78) | -0.28 | 4.87(3.04,7.42) | 4.85(3.06,7.43) | 0.00(-0.01,0.00) |
| Botswana | male | 24.84(15.16,37.71) | 55.74(34.82,84.39) | 1.24 | 4.67(2.92,7.02) | 4.66(2.91,7.02) | 0.00(-0.01,0.01) |
| Brazil | male | 3445.60(2190.50,5179.98) | 5526.93(3479.74,8237.08) | 0.60 | 4.88(3.09,7.36) | 4.89(3.08,7.29) | 0.01(0.01,0.01) |
| Brunei Darussalam | male | 10.58(6.65,16.08) | 18.65(11.85,27.97) | 0.76 | 6.92(4.37,10.45) | 6.97(4.41,10.49) | 0.01(0.00,0.02) |
| Bulgaria | male | 218.79(138.21,329.32) | 177.32(111.59,267.38) | -0.19 | 4.86(3.02,7.28) | 4.87(3.01,7.49) | 0.01(0.00,0.02) |
| Burkina Faso | male | 166.41(104.19,252.77) | 431.78(268.92,677.94) | 1.59 | 4.67(2.92,7.05) | 4.70(3.00,7.23) | 0.03(0.02,0.04) |
| Burundi | male | 107.36(67.29,165.87) | 242.96(150.33,372.53) | 1.26 | 4.72(2.97,7.19) | 4.70(2.94,7.01) | -0.02(-0.03,0.00) |
| Cabo Verde | male | 6.40(3.95,9.72) | 14.09(8.91,21.68) | 1.20 | 4.71(2.98,7.04) | 4.72(2.99,7.21) | -0.01(-0.02,0.00) |
| Cambodia | male | 286.35(181.45,436.50) | 581.21(364.56,891.57) | 1.03 | 7.00(4.46,10.63) | 7.04(4.44,10.76) | 0.03(0.02,0.04) |
| Cameroon | male | 197.14(122.90,295.83) | 613.42(381.63,942.30) | 2.11 | 4.66(2.93,6.92) | 4.70(2.98,7.20) | 0.03(0.02,0.04) |
| Canada | male | 897.49(555.42,1358.05) | 1060.44(658.38,1590.03) | 0.18 | 6.16(3.77,9.29) | 6.17(3.84,9.29) | 0.00(-0.01,0.01) |
| Central African Republic | male | 53.69(33.50,80.72) | 107.10(67.84,161.96) | 0.99 | 4.63(2.94,6.86) | 4.67(2.95,7.04) | 0.02(0.02,0.03) |
| Chad | male | 108.54(68.16,163.40) | 299.57(184.45,451.00) | 1.76 | 4.67(2.93,6.98) | 4.68(2.95,7.11) | 0.01(0.00,0.02) |
| Chile | male | 472.27(291.64,723.34) | 644.48(407.43,969.93) | 0.36 | 6.92(4.29,10.53) | 6.91(4.37,10.34) | 0.00(-0.01,0.01) |
| China | male | 33527.73(21122.09,49874.36) | 42736.50(27029.64,63770.90) | 0.27 | 5.31(3.36,7.90) | 5.31(3.35,8.00) | 0.01(0.00,0.01) |
| Colombia | male | 744.82(460.22,1116.72) | 1190.97(749.00,1829.76) | 0.60 | 4.88(3.04,7.34) | 4.90(3.08,7.53) | 0.01(0.00,0.02) |
| Comoros | male | 9.09(5.55,14.08) | 16.63(10.18,25.28) | 0.83 | 4.73(2.93,7.19) | 4.74(2.94,7.16) | 0.03(0.02,0.03) |
| Congo | male | 47.57(29.45,71.91) | 115.98(71.37,177.08) | 1.44 | 4.67(2.90,6.95) | 4.70(2.96,7.11) | 0.02(0.00,0.03) |
| Cook Islands | male | 0.55(0.34,0.82) | 0.51(0.32,0.77) | -0.07 | 5.93(3.66,8.79) | 5.90(3.67,8.86) | -0.02(-0.02,-0.01) |
| Costa Rica | male | 70.14(43.52,107.96) | 117.93(75.00,178.43) | 0.68 | 4.88(3.05,7.47) | 4.88(3.09,7.41) | 0.00(-0.01,0.01) |
| Côte d'Ivoire | male | 250.78(154.66,386.00) | 582.84(364.78,872.95) | 1.32 | 4.66(2.89,7.12) | 4.68(2.93,6.95) | 0.02(0.01,0.03) |
| Croatia | male | 125.08(79.80,189.73) | 107.95(67.72,163.73) | -0.14 | 4.87(3.09,7.41) | 4.88(3.08,7.46) | 0.01(0.00,0.01) |
| Cuba | male | 279.22(177.61,423.13) | 294.61(182.70,443.17) | 0.06 | 4.89(3.10,7.35) | 4.87(3.03,7.34) | -0.01(-0.02,0.00) |
| Cyprus | male | 30.52(19.43,45.24) | 52.28(32.83,78.75) | 0.71 | 7.45(4.72,11.03) | 7.47(4.67,11.23) | 0.01(0.00,0.01) |
| Czechia | male | 257.23(157.61,389.51) | 273.72(172.82,413.67) | 0.06 | 4.88(3.00,7.41) | 4.86(3.08,7.41) | 0.00(0.00,0.01) |
| Democratic People's Republic of Korea | male | 520.39(316.86,794.00) | 767.72(474.13,1171.17) | 0.48 | 5.31(3.23,8.00) | 5.29(3.26,8.03) | 0.00(-0.01,0.01) |
| Democratic Republic of the Congo | male | 736.53(450.95,1129.02) | 1823.14(1143.68,2775.53) | 1.48 | 4.65(2.91,7.08) | 4.67(2.97,7.15) | 0.03(0.01,0.04) |
| Denmark | male | 198.23(122.47,299.51) | 205.97(129.46,309.84) | 0.04 | 7.43(4.65,11.31) | 7.42(4.67,11.22) | 0.00(0.00,0.01) |
| Djibouti | male | 10.91(6.84,16.48) | 29.78(18.60,44.86) | 1.73 | 4.75(2.98,7.17) | 4.75(2.98,7.17) | 0.00(-0.01,0.01) |
| Dominica | male | 1.70(1.04,2.58) | 1.79(1.13,2.71) | 0.05 | 4.90(3.03,7.40) | 4.88(3.08,7.51) | -0.01(-0.02,0.00) |
| Dominican Republic | male | 158.09(97.83,245.07) | 272.75(169.20,407.22) | 0.73 | 4.88(3.04,7.46) | 4.88(3.03,7.28) | 0.01(0.00,0.02) |
| Ecuador | male | 222.40(136.69,345.16) | 430.65(269.99,651.77) | 0.94 | 4.89(3.02,7.55) | 4.90(3.07,7.44) | 0.01(0.00,0.01) |
| Egypt | male | 1157.26(714.45,1810.63) | 2277.97(1415.86,3457.71) | 0.97 | 4.46(2.80,6.84) | 4.48(2.80,6.78) | 0.02(0.01,0.03) |
| El Salvador | male | 109.46(69.33,162.72) | 140.73(89.01,212.50) | 0.29 | 4.85(3.08,7.17) | 4.86(3.08,7.34) | 0.01(0.01,0.02) |
| Equatorial Guinea | male | 7.57(4.82,11.61) | 33.15(20.50,50.51) | 3.38 | 4.64(2.95,7.05) | 4.69(2.94,7.05) | 0.04(0.04,0.05) |
| Eritrea | male | 59.28(36.15,91.54) | 149.65(93.64,225.25) | 1.52 | 4.69(2.89,7.19) | 4.73(3.01,7.11) | 0.04(0.03,0.05) |
| Estonia | male | 37.65(23.64,57.13) | 32.70(20.64,48.74) | -0.13 | 4.87(3.05,7.39) | 4.89(3.06,7.36) | 0.02(0.01,0.03) |
| Eswatini | male | 14.40(8.90,22.27) | 24.77(15.45,37.41) | 0.72 | 4.68(2.91,7.09) | 4.65(2.92,6.99) | -0.04(-0.05,-0.03) |
| Ethiopia | male | 999.30(627.51,1497.15) | 2278.24(1418.15,3461.06) | 1.28 | 4.74(2.98,7.02) | 4.76(2.99,7.21) | 0.02(0.02,0.03) |
| Fiji | male | 21.66(13.67,32.50) | 27.61(17.41,41.81) | 0.27 | 5.88(3.71,8.82) | 5.87(3.71,8.93) | 0.00(-0.01,0.00) |
| Finland | male | 192.95(122.87,290.38) | 193.39(121.89,292.48) | 0.00 | 7.44(4.76,11.23) | 7.42(4.70,11.12) | 0.00(-0.01,0.01) |
| France | male | 2180.20(1370.22,3327.02) | 2235.74(1396.43,3407.84) | 0.03 | 7.45(4.67,11.37) | 7.44(4.62,11.23) | 0.01(0.00,0.01) |
| Gabon | male | 20.53(12.67,31.24) | 38.70(24.22,59.93) | 0.88 | 4.69(2.91,7.12) | 4.69(2.96,7.24) | 0.01(0.00,0.02) |
| Gambia | male | 19.46(12.09,29.94) | 46.32(29.45,69.22) | 1.38 | 4.68(2.97,7.27) | 4.69(3.01,6.98) | 0.00(-0.01,0.00) |
| Georgia | male | 132.21(83.06,199.34) | 89.88(55.32,137.59) | -0.32 | 4.90(3.06,7.43) | 4.88(3.00,7.46) | -0.28(-1.34,0.80) |
| Germany | male | 3115.83(1928.95,4713.46) | 3032.04(1890.16,4566.07) | -0.03 | 7.45(4.61,11.36) | 7.44(4.64,11.30) | 0.00(0.00,0.00) |
| Ghana | male | 297.79(187.12,450.80) | 684.12(423.73,1050.16) | 1.30 | 4.69(2.92,7.07) | 4.71(2.92,7.14) | 0.00(0.00,0.01) |
| Greece | male | 385.38(238.84,589.00) | 351.08(224.66,524.77) | -0.09 | 7.46(4.64,11.42) | 7.44(4.77,11.15) | 0.00(-0.01,0.01) |
| Greenland | male | 2.20(1.38,3.29) | 1.84(1.14,2.77) | -0.16 | 6.14(3.84,9.20) | 6.15(3.82,9.26) | 0.01(0.00,0.02) |
| Grenada | male | 1.88(1.18,2.85) | 2.74(1.71,4.21) | 0.46 | 4.89(3.10,7.47) | 4.87(3.04,7.55) | -0.01(-0.02,0.00) |
| Guam | male | 4.54(2.83,6.91) | 5.24(3.27,7.83) | 0.15 | 5.94(3.71,9.06) | 5.93(3.69,8.88) | -0.01(-0.01,0.00) |
| Guatemala | male | 159.90(100.57,242.03) | 408.43(256.75,630.30) | 1.55 | 4.83(3.07,7.26) | 4.88(3.04,7.46) | 0.02(0.01,0.03) |
| Guinea | male | 115.93(71.80,177.73) | 239.59(149.64,368.26) | 1.07 | 4.68(2.98,7.16) | 4.69(2.97,7.10) | 0.02(0.01,0.02) |
| Guinea-Bissau | male | 18.54(11.52,27.85) | 38.80(24.16,59.20) | 1.09 | 4.67(2.92,7.06) | 4.70(2.97,7.14) | 0.02(0.01,0.02) |
| Guyana | male | 17.42(10.85,26.25) | 18.81(11.90,28.46) | 0.08 | 4.83(3.01,7.29) | 4.84(3.09,7.35) | 0.00(-0.01,0.01) |
| Haiti | male | 131.75(82.64,200.36) | 282.25(176.16,424.20) | 1.14 | 4.86(3.04,7.30) | 4.86(3.07,7.22) | 0.00(-0.01,0.00) |
| Honduras | male | 94.55(59.20,142.77) | 222.35(141.55,337.14) | 1.35 | 4.89(3.06,7.40) | 4.89(3.11,7.32) | -0.01(-0.02,0.00) |
| Hungary | male | 257.17(163.31,391.11) | 243.73(150.88,364.66) | -0.05 | 4.86(3.10,7.35) | 4.87(3.08,7.36) | 0.02(0.01,0.03) |
| Iceland | male | 9.94(6.13,15.17) | 13.10(8.17,20.03) | 0.32 | 7.43(4.61,11.37) | 7.46(4.63,11.33) | 0.00(-0.01,0.01) |
| India | male | 18082.01(11433.05,26732.13) | 32292.20(20406.23,47976.33) | 0.79 | 4.35(2.77,6.42) | 4.38(2.77,6.49) | 0.02(0.02,0.02) |
| Indonesia | male | 6173.01(3877.06,9154.63) | 9794.05(6109.26,14717.13) | 0.59 | 7.07(4.46,10.43) | 7.08(4.41,10.63) | 0.01(0.01,0.01) |
| Iran (Islamic Republic of) | male | 1166.13(729.59,1750.21) | 2089.03(1323.73,3120.33) | 0.79 | 4.45(2.82,6.67) | 4.46(2.81,6.67) | 0.01(0.00,0.01) |
| Iraq | male | 380.02(238.22,583.92) | 1071.45(674.37,1587.61) | 1.82 | 4.97(3.17,7.38) | 4.98(3.12,7.35) | 0.01(0.00,0.01) |
| Ireland | male | 132.56(83.88,200.63) | 175.31(109.24,266.59) | 0.32 | 7.44(4.70,11.22) | 7.43(4.60,11.30) | 0.00(0.00,0.01) |
| Israel | male | 178.99(110.50,274.25) | 334.48(210.75,509.90) | 0.87 | 7.44(4.59,11.35) | 7.47(4.71,11.37) | 0.00(-0.01,0.01) |
| Italy | male | 2149.18(1335.14,3241.55) | 2044.80(1286.04,3060.50) | -0.05 | 7.46(4.65,11.29) | 7.47(4.71,11.18) | 0.01(0.01,0.01) |
| Jamaica | male | 52.88(32.53,82.21) | 72.29(45.32,109.24) | 0.37 | 4.90(3.03,7.56) | 4.89(3.06,7.37) | 0.00(-0.01,0.01) |
| Japan | male | 4445.14(2817.99,6626.61) | 3935.74(2465.87,5837.18) | -0.11 | 6.98(4.44,10.41) | 6.99(4.39,10.43) | 0.00(0.00,0.00) |
| Jordan | male | 76.79(47.63,120.39) | 283.20(175.46,431.69) | 2.69 | 4.45(2.80,6.80) | 4.47(2.78,6.79) | 0.01(0.00,0.01) |
| Kazakhstan | male | 385.62(237.53,594.31) | 450.53(285.43,682.03) | 0.17 | 4.87(3.01,7.49) | 4.88(3.07,7.37) | 0.00(-0.01,0.00) |
| Kenya | male | 451.30(284.40,682.91) | 1116.72(701.24,1665.47) | 1.47 | 4.76(3.00,7.13) | 4.77(2.99,7.11) | 0.02(0.01,0.02) |
| Kiribati | male | 1.97(1.23,3.00) | 3.29(2.05,4.90) | 0.67 | 5.87(3.70,8.84) | 5.89(3.68,8.85) | 0.01(0.00,0.02) |
| Kuwait | male | 50.11(30.99,75.58) | 126.32(78.58,190.36) | 1.52 | 4.45(2.79,6.72) | 4.46(2.82,6.75) | 0.00(0.00,0.01) |
| Kyrgyzstan | male | 99.18(61.78,151.25) | 158.39(99.33,241.79) | 0.60 | 4.88(3.07,7.45) | 4.91(3.07,7.44) | 0.01(0.00,0.02) |
| Lao People's Democratic Republic | male | 122.68(77.01,187.91) | 255.85(162.15,390.04) | 1.09 | 7.03(4.45,10.70) | 7.05(4.48,10.78) | 0.02(0.01,0.02) |
| Latvia | male | 63.24(39.32,96.61) | 46.30(29.26,70.20) | -0.27 | 4.85(3.05,7.41) | 4.89(3.08,7.51) | 0.03(0.02,0.04) |
| Lebanon | male | 65.76(41.30,100.45) | 116.86(71.54,177.56) | 0.78 | 4.43(2.79,6.70) | 4.43(2.73,6.70) | 0.00(-0.01,0.01) |
| Lesotho | male | 36.20(22.27,54.77) | 48.85(30.65,73.62) | 0.35 | 4.69(2.93,7.15) | 4.64(2.96,6.95) | -0.03(-0.03,-0.02) |
| Liberia | male | 37.04(22.97,55.70) | 103.95(64.54,157.84) | 1.81 | 4.63(2.88,6.99) | 4.65(2.94,6.96) | 0.02(0.01,0.03) |
| Libya | male | 88.08(55.67,134.50) | 171.38(106.18,255.06) | 0.95 | 4.45(2.82,6.76) | 4.42(2.74,6.63) | -0.01(-0.02,0.00) |
| Lithuania | male | 88.68(55.54,134.22) | 67.56(43.01,103.52) | -0.24 | 4.87(3.06,7.36) | 4.89(3.12,7.47) | 0.01(0.01,0.02) |
| Luxembourg | male | 15.09(9.55,23.04) | 23.83(15.14,35.92) | 0.58 | 7.41(4.69,11.34) | 7.43(4.67,11.28) | 0.01(0.00,0.02) |
| Madagascar | male | 237.97(150.40,362.06) | 575.41(355.87,863.55) | 1.42 | 4.71(3.04,7.13) | 4.75(3.01,7.15) | 0.03(0.02,0.03) |
| Malawi | male | 185.93(116.01,279.37) | 375.67(241.01,573.09) | 1.02 | 4.70(2.93,6.99) | 4.75(3.05,7.20) | 0.03(0.02,0.05) |
| Malaysia | male | 593.60(374.83,902.22) | 1227.99(772.68,1845.33) | 1.07 | 7.03(4.49,10.70) | 7.04(4.44,10.57) | 0.00(0.00,0.01) |
| Maldives | male | 6.66(4.19,10.09) | 26.19(16.36,39.65) | 2.93 | 7.05(4.48,10.64) | 7.08(4.51,10.70) | 0.02(0.01,0.03) |
| Mali | male | 161.47(99.70,249.75) | 418.09(255.42,644.37) | 1.59 | 4.68(2.92,7.14) | 4.70(2.93,7.13) | 0.02(0.01,0.03) |
| Malta | male | 14.15(8.95,21.06) | 16.10(10.25,24.22) | 0.14 | 7.43(4.72,11.09) | 7.45(4.72,11.30) | 0.00(-0.01,0.01) |
| Marshall Islands | male | 1.16(0.73,1.76) | 1.72(1.06,2.58) | 0.48 | 5.91(3.67,8.82) | 5.88(3.65,8.85) | -0.01(-0.02,-0.01) |
| Mauritania | male | 40.38(25.51,61.76) | 82.77(51.02,126.60) | 1.05 | 4.69(3.00,7.12) | 4.70(2.93,7.20) | 0.00(-0.01,0.01) |
| Mauritius | male | 40.00(25.39,60.55) | 48.30(30.36,71.81) | 0.21 | 7.02(4.42,10.48) | 7.00(4.42,10.46) | -0.01(-0.02,-0.01) |
| Mexico | male | 1868.00(1171.91,2792.92) | 3076.51(1921.52,4585.44) | 0.65 | 4.90(3.09,7.31) | 4.90(3.07,7.31) | -0.01(-0.01,0.00) |
| Micronesia (Federated States of) | male | 2.72(1.70,4.08) | 3.05(1.89,4.65) | 0.12 | 5.89(3.69,8.70) | 5.90(3.68,8.94) | 0.00(-0.01,0.01) |
| Monaco | male | 1.06(0.68,1.61) | 1.19(0.75,1.78) | 0.12 | 7.46(4.71,11.28) | 7.43(4.67,11.11) | -0.01(-0.02,-0.01) |
| Mongolia | male | 46.87(29.86,71.65) | 84.43(52.82,129.42) | 0.80 | 4.88(3.14,7.40) | 4.86(3.06,7.42) | -0.01(-0.02,0.00) |
| Montenegro | male | 15.78(9.96,23.60) | 15.96(10.00,24.34) | 0.01 | 4.88(3.08,7.30) | 4.87(3.06,7.42) | 0.01(0.00,0.02) |
| Morocco | male | 516.40(319.86,779.44) | 832.51(506.36,1259.99) | 0.61 | 4.44(2.79,6.74) | 4.44(2.70,6.75) | 0.00(-0.01,0.01) |
| Mozambique | male | 239.64(149.27,367.29) | 560.00(344.85,829.47) | 1.34 | 4.68(2.94,7.00) | 4.72(2.97,7.05) | 0.01(0.00,0.02) |
| Myanmar | male | 1327.26(825.18,1975.96) | 1882.73(1207.71,2831.95) | 0.42 | 7.03(4.38,10.47) | 7.04(4.51,10.64) | 0.01(0.01,0.02) |
| Namibia | male | 28.02(17.80,42.58) | 52.48(32.85,80.04) | 0.87 | 4.67(2.97,6.99) | 4.66(2.95,7.05) | 0.00(-0.01,0.01) |
| Nauru | male | 0.28(0.17,0.43) | 0.30(0.19,0.46) | 0.07 | 5.93(3.70,8.87) | 5.91(3.70,8.99) | -0.01(-0.01,0.00) |
| Nepal | male | 371.69(226.04,564.08) | 617.81(379.99,945.35) | 0.66 | 4.38(2.68,6.61) | 4.42(2.72,6.74) | 0.03(0.02,0.03) |
| Netherlands | male | 598.77(376.99,919.18) | 604.66(381.12,895.66) | 0.01 | 7.44(4.70,11.44) | 7.45(4.72,11.13) | 0.01(0.00,0.01) |
| New Zealand | male | 121.92(76.53,182.39) | 141.77(88.69,210.76) | 0.16 | 6.92(4.36,10.34) | 6.90(4.31,10.28) | 0.01(0.00,0.02) |
| Nicaragua | male | 76.33(47.52,116.43) | 157.29(96.66,241.43) | 1.06 | 4.84(3.04,7.24) | 4.88(3.02,7.47) | 0.01(0.00,0.02) |
| Niger | male | 149.45(90.00,229.50) | 422.24(261.61,638.37) | 1.83 | 4.70(2.86,7.06) | 4.69(2.92,7.10) | 0.01(0.00,0.02) |
| Nigeria | male | 1882.62(1199.85,2805.44) | 4193.65(2647.63,6237.27) | 1.23 | 4.69(2.99,7.00) | 4.72(2.98,7.03) | 0.02(0.02,0.03) |
| Niue | male | 0.06(0.04,0.09) | 0.05(0.03,0.07) | -0.21 | 5.90(3.71,8.85) | 5.91(3.73,8.86) | 0.00(-0.01,0.01) |
| North Macedonia | male | 51.14(32.00,76.77) | 59.19(36.86,90.13) | 0.16 | 4.87(3.05,7.33) | 4.87(3.06,7.44) | -0.01(-0.02,0.00) |
| Northern Mariana Islands | male | 1.58(0.98,2.38) | 1.36(0.84,2.02) | -0.14 | 5.91(3.72,8.82) | 5.93(3.70,8.85) | 0.00(-0.01,0.00) |
| Norway | male | 162.78(103.24,242.34) | 199.13(127.38,299.95) | 0.22 | 7.46(4.71,11.16) | 7.46(4.72,11.30) | 0.01(0.00,0.01) |
| Oman | male | 50.89(31.59,77.85) | 169.61(104.98,258.03) | 2.33 | 4.45(2.78,6.66) | 4.46(2.78,6.73) | 0.01(0.00,0.02) |
| Pakistan | male | 2220.97(1417.48,3345.13) | 4637.64(2929.65,6936.48) | 1.09 | 4.41(2.81,6.58) | 4.40(2.80,6.52) | 0.00(-0.01,0.00) |
| Palau | male | 0.48(0.30,0.73) | 0.65(0.41,0.99) | 0.36 | 5.90(3.75,9.01) | 5.88(3.72,8.90) | -0.02(-0.03,-0.01) |
| Palestine | male | 37.99(23.36,57.79) | 106.21(65.44,162.52) | 1.80 | 4.45(2.74,6.70) | 4.43(2.77,6.77) | -0.02(-0.03,-0.01) |
| Panama | male | 56.47(35.96,86.39) | 102.99(64.70,158.04) | 0.82 | 4.89(3.12,7.49) | 4.88(3.07,7.50) | 0.00(-0.01,0.01) |
| Papua New Guinea | male | 112.77(69.66,171.52) | 284.09(171.29,425.58) | 1.52 | 5.88(3.66,9.02) | 5.89(3.56,8.84) | -0.01(-0.01,0.00) |
| Paraguay | male | 89.35(56.22,135.99) | 175.03(111.56,264.23) | 0.96 | 4.90(3.08,7.39) | 4.87(3.08,7.37) | -0.01(-0.01,0.00) |
| Peru | male | 482.08(304.38,736.87) | 846.32(534.35,1276.82) | 0.76 | 4.89(3.09,7.36) | 4.88(3.08,7.38) | 0.00(0.00,0.01) |
| Philippines | male | 2043.61(1278.43,3046.15) | 3995.53(2494.67,5996.06) | 0.96 | 7.05(4.42,10.53) | 7.08(4.43,10.66) | 0.02(0.02,0.02) |
| Poland | male | 947.33(602.03,1411.31) | 1003.29(629.23,1496.87) | 0.06 | 4.87(3.09,7.26) | 4.89(3.05,7.32) | 0.01(0.01,0.02) |
| Portugal | male | 368.16(228.71,564.58) | 357.36(226.68,540.15) | -0.03 | 7.41(4.60,11.36) | 7.44(4.65,11.18) | 0.01(0.00,0.01) |
| Puerto Rico | male | 84.60(53.35,128.43) | 85.47(54.15,128.59) | 0.01 | 4.87(3.08,7.40) | 4.87(3.09,7.27) | -0.01(-0.01,0.00) |
| Qatar | male | 16.39(10.05,25.32) | 130.50(80.10,195.51) | 6.96 | 4.45(2.80,6.74) | 4.44(2.78,6.64) | -0.01(-0.01,0.00) |
| Republic of Korea | male | 1744.35(1096.08,2626.83) | 1942.54(1206.68,2878.95) | 0.11 | 6.93(4.37,10.36) | 6.94(4.29,10.41) | 0.01(0.00,0.01) |
| Republic of Moldova | male | 105.52(66.63,158.74) | 96.46(59.97,145.60) | -0.09 | 4.87(3.08,7.29) | 4.89(3.01,7.38) | 0.01(0.00,0.01) |
| Romania | male | 576.55(360.62,869.12) | 489.63(306.18,743.64) | -0.15 | 4.86(3.03,7.35) | 4.89(3.10,7.44) | 0.03(0.02,0.04) |
| Russian Federation | male | 3695.24(2352.69,5506.52) | 3631.23(2287.90,5355.71) | -0.02 | 4.87(3.08,7.27) | 4.90(3.08,7.28) | 0.02(0.02,0.03) |
| Rwanda | male | 139.08(85.91,214.40) | 273.86(168.77,409.60) | 0.97 | 4.72(2.95,7.22) | 4.73(2.94,7.02) | 0.02(0.01,0.03) |
| Saint Kitts and Nevis | male | 0.94(0.59,1.43) | 1.58(0.98,2.38) | 0.67 | 4.89(3.04,7.38) | 4.85(3.04,7.30) | 0.00(-0.01,0.00) |
| Saint Lucia | male | 2.97(1.86,4.56) | 4.61(2.87,6.97) | 0.55 | 4.88(3.06,7.41) | 4.87(3.03,7.36) | 0.00(-0.01,0.01) |
| Saint Vincent and the Grenadines | male | 2.46(1.52,3.80) | 2.95(1.83,4.48) | 0.20 | 4.88(3.05,7.40) | 4.89(3.04,7.46) | -0.01(-0.01,0.00) |
| Samoa | male | 4.55(2.80,6.82) | 6.07(3.85,9.09) | 0.34 | 5.91(3.66,8.84) | 5.92(3.80,8.87) | -0.01(-0.02,0.00) |
| San Marino | male | 0.90(0.57,1.36) | 1.12(0.70,1.71) | 0.24 | 7.44(4.63,11.20) | 7.43(4.60,11.38) | 0.00(0.00,0.01) |
| Sao Tome and Principe | male | 2.30(1.47,3.50) | 4.64(2.90,7.11) | 1.02 | 4.71(3.03,7.17) | 4.69(2.95,7.18) | 0.00(-0.01,0.01) |
| Saudi Arabia | male | 384.21(232.96,589.82) | 1123.66(704.59,1711.86) | 1.92 | 4.44(2.74,6.72) | 4.43(2.77,6.64) | -0.02(-0.03,-0.01) |
| Senegal | male | 142.21(88.62,213.80) | 318.13(198.92,478.58) | 1.24 | 4.69(2.93,7.02) | 4.70(2.92,7.07) | 0.01(0.00,0.01) |
| Serbia | male | 238.55(149.12,366.84) | 224.12(141.05,338.14) | -0.06 | 4.88(3.06,7.50) | 4.87(3.06,7.37) | 0.00(-0.01,0.00) |
| Seychelles | male | 2.52(1.61,3.81) | 4.20(2.71,6.32) | 0.66 | 7.05(4.48,10.57) | 7.06(4.52,10.58) | 0.00(-0.01,0.01) |
| Sierra Leone | male | 71.51(45.81,110.18) | 174.67(107.05,270.72) | 1.44 | 4.66(2.95,7.15) | 4.68(2.95,7.15) | 0.02(0.01,0.03) |
| Singapore | male | 125.73(78.40,189.10) | 220.41(137.99,338.72) | 0.75 | 6.94(4.35,10.39) | 6.95(4.44,10.76) | 0.01(0.00,0.02) |
| Slovakia | male | 130.31(81.02,195.06) | 142.85(89.24,210.64) | 0.10 | 4.85(3.02,7.27) | 4.86(3.03,7.19) | 0.00(-0.01,0.01) |
| Slovenia | male | 50.18(31.58,75.88) | 53.89(33.90,80.96) | 0.07 | 4.85(3.06,7.33) | 4.88(3.01,7.36) | 0.01(0.00,0.02) |
| Solomon Islands | male | 8.69(5.41,13.23) | 18.08(11.26,27.25) | 1.08 | 5.89(3.71,8.98) | 5.91(3.72,8.94) | -0.01(-0.01,0.00) |
| Somalia | male | 142.83(90.64,212.00) | 417.68(256.75,639.21) | 1.92 | 4.73(2.94,7.02) | 4.72(2.96,7.21) | 0.00(0.00,0.01) |
| South Africa | male | 796.74(501.93,1204.74) | 1352.22(849.18,2025.90) | 0.70 | 4.71(2.99,7.09) | 4.69(2.94,7.02) | -0.01(-0.01,0.00) |
| South Sudan | male | 127.39(78.98,191.38) | 180.87(114.29,274.51) | 0.42 | 4.71(2.98,7.03) | 4.71(2.97,7.07) | -0.01(-0.02,0.00) |
| Spain | male | 1457.28(910.69,2206.14) | 1615.18(1028.06,2408.37) | 0.11 | 7.45(4.65,11.26) | 7.47(4.73,11.21) | 0.00(0.00,0.01) |
| Sri Lanka | male | 625.30(391.26,946.59) | 769.20(484.67,1154.24) | 0.23 | 7.27(4.61,10.91) | 7.03(4.43,10.54) | -0.02(-0.05,0.00) |
| Sudan | male | 380.86(236.34,578.41) | 846.40(524.36,1261.53) | 1.22 | 4.43(2.77,6.67) | 4.45(2.78,6.70) | -0.01(-0.01,0.00) |
| Suriname | male | 9.14(5.60,14.00) | 14.14(8.77,21.06) | 0.55 | 4.87(3.04,7.38) | 4.87(3.02,7.29) | 0.00(-0.01,0.00) |
| Sweden | male | 320.22(202.31,478.57) | 368.76(234.09,544.92) | 0.15 | 7.49(4.69,11.22) | 7.46(4.73,11.17) | 0.00(-0.01,0.00) |
| Switzerland | male | 272.48(172.82,410.52) | 321.55(200.90,490.72) | 0.18 | 7.41(4.66,11.12) | 7.43(4.66,11.21) | 0.01(0.01,0.02) |
| Syrian Arab Republic | male | 245.62(151.41,374.00) | 296.21(184.20,451.62) | 0.21 | 4.46(2.74,6.74) | 4.43(2.80,6.78) | -0.02(-0.03,-0.01) |
| Taiwan (Province of China) | male | 604.50(373.64,926.85) | 704.43(447.86,1059.43) | 0.17 | 5.43(3.35,8.29) | 5.41(3.43,8.25) | -0.01(-0.02,0.00) |
| Tajikistan | male | 114.07(69.77,174.98) | 231.41(141.53,358.87) | 1.03 | 4.89(2.99,7.40) | 4.90(3.05,7.63) | 0.01(0.00,0.02) |
| Thailand | male | 2021.12(1263.35,3073.44) | 2662.24(1673.48,4019.33) | 0.32 | 7.05(4.44,10.60) | 7.08(4.47,10.72) | 0.01(0.01,0.02) |
| Timor-Leste | male | 25.45(15.79,38.78) | 42.83(27.31,65.04) | 0.68 | 7.01(4.41,10.54) | 7.03(4.48,10.61) | 0.04(0.02,0.06) |
| Togo | male | 67.70(41.89,101.52) | 164.10(103.58,246.58) | 1.42 | 4.69(2.96,7.13) | 4.69(2.98,7.04) | 0.01(0.00,0.02) |
| Tokelau | male | 0.04(0.03,0.06) | 0.04(0.03,0.06) | -0.04 | 5.94(3.78,9.02) | 5.92(3.74,8.90) | -0.01(-0.01,0.00) |
| Tonga | male | 2.49(1.58,3.73) | 2.77(1.75,4.15) | 0.11 | 5.92(3.78,8.87) | 5.93(3.77,8.93) | 0.00(0.00,0.01) |
| Trinidad and Tobago | male | 28.90(18.10,43.55) | 36.77(23.30,55.90) | 0.27 | 4.89(3.05,7.33) | 4.88(3.05,7.46) | 0.00(-0.01,0.00) |
| Tunisia | male | 176.97(109.78,272.83) | 270.89(164.19,403.07) | 0.53 | 4.47(2.81,6.86) | 4.45(2.70,6.64) | -0.01(-0.02,0.00) |
| Turkey | male | 1174.30(727.61,1761.46) | 1861.85(1189.88,2833.59) | 0.59 | 4.09(2.52,6.19) | 4.11(2.60,6.24) | 0.00(-0.01,0.01) |
| Turkmenistan | male | 80.60(50.93,122.95) | 130.70(82.13,200.00) | 0.62 | 4.88(3.05,7.41) | 4.89(3.07,7.44) | 0.01(0.00,0.01) |
| Tuvalu | male | 0.25(0.15,0.37) | 0.36(0.23,0.54) | 0.49 | 5.92(3.69,9.00) | 5.90(3.65,8.74) | -0.01(-0.01,0.00) |
| Uganda | male | 325.00(200.00,486.97) | 800.35(495.62,1244.67) | 1.46 | 4.69(2.91,6.97) | 4.74(2.93,7.27) | 0.04(0.03,0.05) |
| Ukraine | male | 1260.56(787.92,1896.75) | 1101.53(691.90,1645.87) | -0.13 | 4.89(3.05,7.38) | 4.90(3.09,7.34) | 0.02(0.01,0.02) |
| United Arab Emirates | male | 64.21(39.27,95.51) | 408.08(243.20,620.86) | 5.36 | 4.43(2.70,6.70) | 4.45(2.69,6.71) | 0.00(-0.01,0.01) |
| United Kingdom | male | 2126.36(1331.26,3134.33) | 2419.99(1530.41,3607.47) | 0.14 | 7.43(4.64,11.00) | 7.43(4.68,11.08) | 0.00(0.00,0.00) |
| United Republic of Tanzania | male | 497.31(300.89,761.63) | 1141.95(720.52,1778.13) | 1.30 | 4.78(2.96,7.18) | 4.76(2.96,7.33) | 0.01(0.00,0.02) |
| United States of America | male | 9194.42(5778.15,13702.89) | 10323.68(6486.74,15276.88) | 0.12 | 6.94(4.34,10.34) | 6.57(4.13,9.73) | -0.25(-0.32,-0.18) |
| United States Virgin Islands | male | 2.47(1.51,3.78) | 2.44(1.55,3.63) | -0.01 | 4.90(3.00,7.44) | 4.90(3.08,7.27) | 0.00(-0.01,0.01) |
| Uruguay | male | 104.07(65.70,155.91) | 113.60(71.34,172.86) | 0.09 | 6.93(4.36,10.39) | 6.92(4.37,10.48) | -0.01(-0.01,0.00) |
| Uzbekistan | male | 455.26(284.04,680.43) | 839.86(517.84,1264.82) | 0.84 | 4.87(3.07,7.32) | 4.90(3.04,7.38) | 0.01(0.00,0.02) |
| Vanuatu | male | 3.96(2.46,5.99) | 8.17(5.06,12.72) | 1.06 | 5.87(3.68,8.90) | 5.89(3.65,9.10) | 0.01(0.00,0.01) |
| Venezuela (Bolivarian Republic of) | male | 428.83(266.75,635.86) | 691.39(430.18,1046.07) | 0.61 | 4.88(3.04,7.25) | 4.88(3.04,7.43) | 0.00(-0.01,0.01) |
| Viet Nam | male | 2119.73(1352.93,3160.64) | 3709.43(2307.22,5547.63) | 0.75 | 7.06(4.51,10.49) | 7.09(4.45,10.58) | 0.01(0.00,0.02) |
| Yemen | male | 242.61(149.29,369.31) | 647.67(403.76,991.43) | 1.67 | 4.42(2.79,6.67) | 4.44(2.81,6.70) | 0.02(0.01,0.03) |
| Zambia | male | 150.92(94.55,228.31) | 381.56(236.41,582.10) | 1.53 | 4.70(2.99,7.04) | 4.71(2.95,7.08) | -0.01(-0.02,0.00) |
| Zimbabwe | male | 196.61(122.19,298.79) | 305.75(193.49,470.66) | 0.56 | 4.68(2.90,7.04) | 4.67(2.94,7.06) | 0.00(-0.01,0.01) |
| Afghanistan | female | 395.83(245.75,603.71) | 1310.81(810.31,1965.66) | 2.31 | 8.11(5.09,12.29) | 8.13(5.16,11.96) | 0.00(-0.01,0.01) |
| Albania | female | 146.64(93.00,219.40) | 133.96(85.42,200.98) | -0.09 | 9.32(5.92,13.90) | 9.33(5.94,14.13) | 0.00(0.00,0.01) |
| Algeria | female | 918.24(576.68,1379.11) | 1797.53(1135.48,2681.09) | 0.96 | 8.27(5.24,12.24) | 8.29(5.23,12.37) | 0.00(0.00,0.01) |
| American Samoa | female | 2.33(1.49,3.53) | 2.83(1.80,4.28) | 0.21 | 10.55(6.69,15.81) | 10.50(6.71,15.84) | -0.01(-0.02,-0.01) |
| Andorra | female | 2.93(1.86,4.40) | 4.83(3.06,7.22) | 0.65 | 10.17(6.46,15.22) | 10.18(6.40,15.34) | 0.00(-0.01,0.00) |
| Angola | female | 378.10(242.18,562.31) | 1169.37(741.92,1754.31) | 2.09 | 8.63(5.54,12.70) | 8.67(5.49,12.78) | 0.02(0.01,0.03) |
| Antigua and Barbuda | female | 2.91(1.84,4.36) | 4.59(2.90,6.88) | 0.58 | 9.29(5.89,13.90) | 9.28(5.96,13.90) | -0.01(-0.01,0.00) |
| Argentina | female | 1948.63(1246.05,2894.35) | 2859.89(1789.24,4304.55) | 0.47 | 11.70(7.48,17.42) | 11.70(7.34,17.66) | 0.00(0.00,0.01) |
| Armenia | female | 165.71(104.83,247.96) | 158.29(100.66,236.89) | -0.04 | 9.32(5.88,13.90) | 9.32(5.92,13.93) | 0.00(0.00,0.01) |
| Australia | female | 1056.24(670.52,1577.76) | 1600.11(1009.20,2377.00) | 0.51 | 11.64(7.37,17.47) | 11.66(7.37,17.42) | 0.00(0.00,0.01) |
| Austria | female | 453.87(285.73,680.15) | 520.55(329.16,777.94) | 0.15 | 10.17(6.38,15.34) | 10.21(6.35,15.33) | 0.01(0.00,0.01) |
| Azerbaijan | female | 346.68(219.52,523.37) | 527.96(330.14,796.96) | 0.52 | 9.30(5.90,13.94) | 9.30(5.89,14.17) | 0.00(-0.01,0.00) |
| Bahamas | female | 12.34(7.85,18.44) | 19.52(12.32,29.22) | 0.58 | 9.28(5.89,13.82) | 9.29(5.83,13.82) | 0.00(-0.01,0.00) |
| Bahrain | female | 17.45(11.11,26.43) | 50.87(32.06,75.33) | 1.91 | 8.23(5.17,12.39) | 8.22(5.16,12.17) | 0.00(0.00,0.01) |
| Bangladesh | female | 3748.82(2354.14,5633.16) | 6824.42(4385.81,10174.81) | 0.82 | 8.13(5.14,12.23) | 8.13(5.22,12.11) | 0.01(0.00,0.02) |
| Barbados | female | 12.73(8.01,18.90) | 15.29(9.66,22.79) | 0.20 | 9.28(5.83,13.90) | 9.31(5.96,13.90) | 0.00(0.00,0.01) |
| Belarus | female | 537.40(344.82,806.59) | 500.94(319.05,746.59) | -0.07 | 9.29(5.98,13.99) | 9.32(5.90,13.90) | 0.01(0.00,0.01) |
| Belgium | female | 571.56(362.50,853.64) | 647.37(405.14,969.37) | 0.13 | 10.16(6.49,15.28) | 10.16(6.33,15.14) | -0.01(-0.01,0.00) |
| Belize | female | 7.41(4.76,11.47) | 19.41(12.26,29.22) | 1.62 | 9.30(5.92,14.10) | 9.28(5.88,13.92) | -0.01(-0.01,0.00) |
| Benin | female | 183.91(113.28,276.08) | 488.60(304.73,734.13) | 1.66 | 8.68(5.40,12.85) | 8.69(5.44,12.96) | 0.01(0.00,0.02) |
| Bermuda | female | 3.18(1.98,4.73) | 3.26(2.07,4.85) | 0.03 | 9.32(5.80,13.88) | 9.30(5.92,13.92) | 0.00(-0.01,0.01) |
| Bhutan | female | 20.76(13.07,31.25) | 31.00(19.86,46.47) | 0.49 | 8.09(5.09,12.12) | 8.14(5.19,12.16) | 0.01(0.01,0.02) |
| Bolivia (Plurinational State of) | female | 269.01(170.81,404.91) | 548.49(346.60,829.83) | 1.04 | 9.25(5.94,13.84) | 9.29(5.86,14.03) | 0.01(0.01,0.02) |
| Bosnia and Herzegovina | female | 222.90(142.70,332.48) | 168.00(107.02,253.23) | -0.25 | 9.31(5.96,13.90) | 9.31(5.94,14.08) | 0.00(-0.01,0.01) |
| Botswana | female | 52.28(33.16,78.80) | 106.54(67.61,158.80) | 1.04 | 8.66(5.49,12.94) | 8.59(5.45,12.77) | -0.02(-0.02,-0.01) |
| Brazil | female | 6777.99(4284.37,10050.88) | 11041.14(7009.59,16286.33) | 0.63 | 9.26(5.82,13.67) | 9.29(5.90,13.74) | 0.01(0.01,0.01) |
| Brunei Darussalam | female | 13.16(8.28,19.82) | 26.62(16.85,39.77) | 1.02 | 11.61(7.38,17.43) | 11.62(7.34,17.40) | 0.01(0.00,0.01) |
| Bulgaria | female | 431.95(271.93,643.91) | 348.51(220.89,521.45) | -0.19 | 9.33(5.84,13.98) | 9.31(5.90,14.05) | 0.00(-0.01,0.00) |
| Burkina Faso | female | 358.55(224.47,539.11) | 889.25(554.55,1350.29) | 1.48 | 8.62(5.48,12.87) | 8.69(5.47,13.18) | 0.03(0.02,0.04) |
| Burundi | female | 209.39(133.43,314.54) | 444.73(280.51,665.58) | 1.12 | 8.56(5.44,12.81) | 8.55(5.40,12.64) | 0.01(0.00,0.02) |
| Cabo Verde | female | 14.06(8.96,21.02) | 25.08(15.86,37.38) | 0.78 | 8.76(5.58,13.01) | 8.74(5.58,13.03) | 0.00(-0.01,0.01) |
| Cambodia | female | 502.28(315.86,752.77) | 910.86(578.50,1376.94) | 0.81 | 10.50(6.64,15.70) | 10.55(6.74,15.99) | 0.02(0.02,0.03) |
| Cameroon | female | 392.44(249.44,584.10) | 1165.70(722.56,1759.98) | 1.97 | 8.64(5.54,12.68) | 8.68(5.43,13.12) | 0.02(0.01,0.02) |
| Canada | female | 2365.00(1517.08,3551.80) | 3248.10(2059.63,4815.42) | 0.37 | 15.57(9.89,23.52) | 15.55(9.92,23.21) | 0.00(-0.01,0.00) |
| Central African Republic | female | 105.60(66.60,160.08) | 210.99(134.61,317.74) | 1.00 | 8.61(5.48,12.97) | 8.63(5.52,12.95) | 0.02(0.01,0.02) |
| Chad | female | 224.65(138.81,339.64) | 583.19(368.06,877.58) | 1.60 | 8.67(5.41,13.09) | 8.67(5.51,13.04) | 0.00(-0.01,0.01) |
| Chile | female | 782.21(494.32,1173.84) | 1191.72(751.91,1751.21) | 0.52 | 11.68(7.39,17.53) | 11.68(7.37,17.27) | 0.00(0.00,0.00) |
| China | female | 62212.15(39024.95,93019.43) | 82085.48(51862.67,121540.65) | 0.32 | 10.48(6.57,15.68) | 10.53(6.64,15.66) | 0.02(0.01,0.02) |
| Colombia | female | 1478.39(923.61,2228.95) | 2389.44(1517.39,3607.10) | 0.62 | 9.26(5.81,13.80) | 9.31(5.92,14.01) | 0.01(0.01,0.02) |
| Comoros | female | 17.25(10.83,26.08) | 30.23(19.18,45.13) | 0.75 | 8.59(5.41,12.86) | 8.61(5.46,12.86) | 0.01(0.00,0.02) |
| Congo | female | 93.18(59.39,140.37) | 219.09(136.63,327.84) | 1.35 | 8.63(5.50,12.71) | 8.64(5.38,12.77) | 0.01(0.00,0.01) |
| Cook Islands | female | 0.91(0.58,1.38) | 0.99(0.61,1.50) | 0.08 | 10.54(6.75,15.70) | 10.54(6.58,16.01) | 0.00(-0.01,0.00) |
| Costa Rica | female | 135.32(86.50,202.10) | 242.64(151.62,363.80) | 0.79 | 9.27(5.96,13.83) | 9.29(5.82,14.04) | 0.01(0.00,0.01) |
| Côte d'Ivoire | female | 437.84(279.89,667.85) | 1016.40(637.28,1516.61) | 1.32 | 8.59(5.50,12.88) | 8.67(5.50,12.97) | 0.03(0.02,0.04) |
| Croatia | female | 248.21(155.75,369.59) | 212.29(136.26,313.47) | -0.14 | 9.27(5.80,13.91) | 9.30(5.87,13.79) | 0.00(0.00,0.01) |
| Cuba | female | 527.91(325.32,791.74) | 561.37(350.49,845.21) | 0.06 | 9.29(5.73,13.87) | 9.27(5.77,13.91) | 0.00(0.00,0.01) |
| Cyprus | female | 41.20(26.14,60.89) | 78.98(49.89,116.89) | 0.92 | 10.16(6.43,15.12) | 10.20(6.44,15.13) | 0.01(0.00,0.01) |
| Czechia | female | 510.91(323.03,759.63) | 524.92(333.48,792.86) | 0.03 | 9.28(5.85,13.87) | 9.28(5.88,14.02) | -0.01(-0.02,0.00) |
| Democratic People's Republic of Korea | female | 1148.18(729.84,1706.45) | 1471.50(949.19,2250.05) | 0.28 | 10.44(6.64,15.54) | 10.43(6.68,16.00) | 0.00(-0.01,0.01) |
| Democratic Republic of the Congo | female | 1425.63(911.63,2150.79) | 3350.96(2109.34,5025.73) | 1.35 | 8.57(5.41,12.85) | 8.63(5.48,12.85) | 0.03(0.02,0.03) |
| Denmark | female | 292.70(184.13,433.06) | 325.19(206.42,486.54) | 0.11 | 10.15(6.39,15.10) | 10.15(6.44,15.12) | 0.01(0.00,0.01) |
| Djibouti | female | 16.50(10.36,25.01) | 48.86(30.83,73.23) | 1.96 | 8.59(5.39,12.85) | 8.60(5.53,12.90) | 0.00(0.00,0.01) |
| Dominica | female | 3.23(2.02,4.87) | 3.24(2.07,4.88) | 0.00 | 9.29(5.83,14.03) | 9.28(5.89,14.11) | 0.00(-0.01,0.01) |
| Dominican Republic | female | 320.08(199.75,483.26) | 512.75(327.11,764.60) | 0.60 | 9.30(5.78,14.00) | 9.30(5.94,13.87) | 0.00(0.00,0.01) |
| Ecuador | female | 432.85(274.47,646.17) | 831.37(526.85,1228.55) | 0.92 | 9.26(5.87,13.82) | 9.29(5.89,13.71) | 0.01(0.00,0.01) |
| Egypt | female | 2047.49(1287.87,3063.71) | 3931.44(2521.56,5900.51) | 0.92 | 8.23(5.19,12.27) | 8.28(5.32,12.34) | 0.01(0.00,0.02) |
| El Salvador | female | 227.65(145.47,341.65) | 317.91(198.94,471.64) | 0.40 | 9.27(5.92,13.68) | 9.28(5.78,13.83) | 0.01(0.01,0.02) |
| Equatorial Guinea | female | 16.50(10.43,24.76) | 52.77(33.21,79.45) | 2.20 | 8.59(5.47,12.82) | 8.65(5.45,12.99) | 0.02(0.02,0.03) |
| Eritrea | female | 109.96(69.22,165.02) | 266.22(167.68,402.67) | 1.42 | 8.53(5.32,12.84) | 8.59(5.39,12.89) | 0.02(0.02,0.03) |
| Estonia | female | 80.33(51.78,120.53) | 66.56(42.08,98.89) | -0.17 | 9.28(5.96,13.85) | 9.33(5.89,14.01) | 0.02(0.01,0.02) |
| Eswatini | female | 31.50(19.33,47.77) | 49.57(30.91,74.80) | 0.57 | 8.68(5.30,13.02) | 8.60(5.35,12.94) | -0.04(-0.05,-0.03) |
| Ethiopia | female | 1873.38(1175.98,2748.06) | 4143.36(2668.22,6220.82) | 1.21 | 8.60(5.46,12.62) | 8.66(5.60,12.95) | 0.03(0.03,0.03) |
| Fiji | female | 37.92(23.92,57.37) | 47.94(30.16,72.03) | 0.26 | 10.52(6.66,15.80) | 10.51(6.63,15.78) | 0.00(-0.01,0.00) |
| Finland | female | 290.75(185.04,437.89) | 313.55(198.97,464.12) | 0.08 | 10.17(6.45,15.38) | 10.14(6.46,15.04) | 0.00(-0.01,0.01) |
| France | female | 3257.55(2091.33,4835.34) | 3756.72(2359.09,5563.82) | 0.15 | 10.19(6.49,15.14) | 10.17(6.37,15.15) | 0.00(0.00,0.01) |
| Gabon | female | 37.72(24.08,56.94) | 77.28(48.75,114.04) | 1.05 | 8.66(5.48,13.12) | 8.64(5.47,12.79) | 0.00(-0.01,0.01) |
| Gambia | female | 36.43(23.03,54.59) | 90.65(57.29,136.38) | 1.49 | 8.63(5.43,12.93) | 8.67(5.44,13.04) | 0.01(0.00,0.02) |
| Georgia | female | 280.29(176.71,426.31) | 183.86(117.74,274.77) | -0.34 | 9.32(5.86,14.14) | 9.30(5.88,13.86) | -0.22(-2.65,2.27) |
| Germany | female | 4716.03(2999.59,7085.23) | 4860.64(3112.19,7155.30) | 0.03 | 10.15(6.42,15.31) | 10.14(6.43,15.08) | 0.00(-0.01,0.00) |
| Ghana | female | 579.22(362.74,871.92) | 1387.10(878.93,2092.31) | 1.39 | 8.67(5.46,13.09) | 8.70(5.49,13.01) | 0.01(0.01,0.02) |
| Greece | female | 587.51(368.56,873.95) | 607.04(383.02,902.41) | 0.03 | 10.17(6.39,15.16) | 10.16(6.44,15.16) | -0.01(-0.01,0.00) |
| Greenland | female | 4.04(2.58,6.03) | 4.49(2.83,6.69) | 0.11 | 15.40(9.74,22.95) | 15.46(9.72,22.98) | 0.01(0.01,0.02) |
| Grenada | female | 3.68(2.32,5.55) | 4.92(3.12,7.32) | 0.34 | 9.27(5.90,13.93) | 9.27(5.89,13.87) | 0.00(-0.01,0.01) |
| Guam | female | 6.85(4.40,10.29) | 8.68(5.54,13.12) | 0.27 | 10.57(6.80,15.88) | 10.59(6.73,15.99) | 0.01(0.00,0.01) |
| Guatemala | female | 320.13(201.92,483.21) | 846.35(532.03,1267.24) | 1.64 | 9.24(5.86,14.00) | 9.25(5.78,13.95) | 0.01(0.01,0.02) |
| Guinea | female | 238.10(150.16,354.88) | 502.94(314.45,764.33) | 1.11 | 8.64(5.48,12.79) | 8.69(5.50,13.07) | 0.02(0.01,0.02) |
| Guinea-Bissau | female | 38.77(24.75,58.50) | 78.88(49.44,118.49) | 1.03 | 8.66(5.54,12.95) | 8.69(5.47,13.00) | 0.00(0.00,0.01) |
| Guyana | female | 34.11(21.83,51.36) | 36.94(23.52,55.23) | 0.08 | 9.21(5.88,13.90) | 9.22(5.85,13.76) | 0.01(0.00,0.01) |
| Haiti | female | 270.36(171.35,399.02) | 586.37(373.64,878.38) | 1.17 | 9.20(5.88,13.56) | 9.19(5.85,13.77) | 0.00(-0.01,0.01) |
| Honduras | female | 187.30(117.33,283.96) | 462.74(286.98,700.90) | 1.47 | 9.27(5.87,13.78) | 9.27(5.83,13.95) | 0.00(0.00,0.01) |
| Hungary | female | 526.12(337.00,787.17) | 496.00(317.01,738.60) | -0.06 | 9.31(5.92,13.99) | 9.32(5.91,14.11) | 0.01(0.00,0.01) |
| Iceland | female | 13.29(8.43,19.96) | 18.83(12.01,28.42) | 0.42 | 10.17(6.45,15.26) | 10.18(6.49,15.41) | 0.01(0.00,0.01) |
| India | female | 31024.43(19646.66,45997.90) | 57179.14(36394.16,84849.89) | 0.84 | 8.09(5.14,11.99) | 8.13(5.18,12.06) | 0.02(0.02,0.02) |
| Indonesia | female | 9435.42(5947.58,14074.94) | 14450.94(9087.69,21470.78) | 0.53 | 10.58(6.66,15.85) | 10.62(6.68,15.75) | 0.01(0.01,0.02) |
| Iran (Islamic Republic of) | female | 2073.11(1296.89,3086.50) | 3777.77(2386.71,5574.10) | 0.82 | 8.29(5.21,12.29) | 8.30(5.22,12.27) | 0.00(0.00,0.01) |
| Iraq | female | 599.79(372.40,896.87) | 1679.92(1061.85,2543.22) | 1.80 | 8.21(5.19,12.28) | 8.23(5.20,12.42) | 0.01(0.00,0.01) |
| Ireland | female | 184.81(116.63,273.59) | 274.82(176.42,411.51) | 0.49 | 10.15(6.36,15.06) | 10.15(6.47,15.20) | 0.00(0.00,0.01) |
| Israel | female | 250.53(159.80,371.70) | 478.57(302.34,727.85) | 0.91 | 10.17(6.49,15.13) | 10.18(6.45,15.54) | 0.01(0.00,0.01) |
| Italy | female | 3326.53(2114.08,4926.42) | 3559.40(2266.13,5268.89) | 0.07 | 10.19(6.47,15.16) | 10.23(6.42,15.18) | 0.02(0.02,0.02) |
| Jamaica | female | 106.20(66.06,160.67) | 140.31(89.68,207.96) | 0.32 | 9.32(5.82,14.14) | 9.29(5.92,13.84) | -0.01(-0.01,0.00) |
| Japan | female | 8357.77(5302.59,12470.83) | 8578.90(5449.25,12712.15) | 0.03 | 11.74(7.39,17.53) | 11.76(7.41,17.60) | 0.01(0.01,0.01) |
| Jordan | female | 125.87(79.93,190.96) | 439.60(279.18,667.85) | 2.49 | 8.22(5.28,12.36) | 8.25(5.23,12.54) | 0.01(0.00,0.02) |
| Kazakhstan | female | 781.12(495.92,1160.84) | 913.97(573.24,1379.86) | 0.17 | 9.26(5.88,13.74) | 9.27(5.84,13.90) | 0.00(0.00,0.01) |
| Kenya | female | 839.01(527.50,1243.55) | 2059.43(1294.56,3059.21) | 1.45 | 8.64(5.44,12.77) | 8.65(5.45,12.90) | 0.01(0.01,0.02) |
| Kiribati | female | 3.68(2.33,5.45) | 6.26(3.99,9.26) | 0.70 | 10.47(6.66,15.63) | 10.51(6.70,15.58) | 0.00(0.00,0.01) |
| Kuwait | female | 62.07(39.47,93.47) | 203.29(127.60,303.53) | 2.28 | 8.26(5.25,12.26) | 8.26(5.19,12.39) | 0.00(-0.01,0.00) |
| Kyrgyzstan | female | 198.23(124.52,302.05) | 308.68(196.03,465.92) | 0.56 | 9.29(5.88,14.08) | 9.30(5.92,13.92) | 0.01(0.00,0.01) |
| Lao People's Democratic Republic | female | 196.43(125.20,293.32) | 380.75(241.88,572.06) | 0.94 | 10.54(6.76,15.74) | 10.59(6.72,15.82) | 0.02(0.01,0.02) |
| Latvia | female | 136.97(86.26,203.15) | 98.45(63.44,146.71) | -0.28 | 9.26(5.81,13.83) | 9.29(5.98,13.97) | 0.02(0.01,0.02) |
| Lebanon | female | 124.53(78.66,190.14) | 229.27(145.03,344.95) | 0.84 | 8.24(5.20,12.60) | 8.27(5.25,12.35) | 0.01(0.00,0.01) |
| Lesotho | female | 71.16(45.23,108.46) | 91.30(58.25,138.27) | 0.28 | 8.68(5.49,13.07) | 8.60(5.55,12.90) | -0.03(-0.04,-0.03) |
| Liberia | female | 73.45(46.27,110.71) | 193.33(121.27,289.79) | 1.63 | 8.56(5.39,12.81) | 8.64(5.43,12.96) | 0.02(0.02,0.03) |
| Libya | female | 138.41(87.42,208.26) | 296.77(187.56,444.17) | 1.14 | 8.24(5.24,12.36) | 8.23(5.15,12.35) | -0.01(-0.01,0.00) |
| Lithuania | female | 186.53(119.04,275.65) | 143.71(91.39,215.76) | -0.23 | 9.25(5.87,13.74) | 9.28(5.83,13.83) | 0.01(0.00,0.01) |
| Luxembourg | female | 22.28(14.13,33.20) | 35.40(21.88,52.69) | 0.59 | 10.17(6.42,15.17) | 10.15(6.26,15.09) | 0.00(0.00,0.01) |
| Madagascar | female | 438.81(279.64,662.30) | 1049.80(649.29,1615.40) | 1.39 | 8.57(5.52,12.92) | 8.59(5.33,13.00) | 0.01(0.01,0.02) |
| Malawi | female | 355.24(224.74,540.15) | 726.08(456.90,1092.36) | 1.04 | 8.53(5.37,12.78) | 8.57(5.43,12.72) | 0.03(0.02,0.03) |
| Malaysia | female | 882.86(552.48,1327.04) | 1695.01(1082.12,2525.64) | 0.92 | 10.55(6.57,15.82) | 10.58(6.75,15.77) | 0.01(0.00,0.01) |
| Maldives | female | 9.53(6.02,14.28) | 22.79(14.56,34.27) | 1.39 | 10.53(6.55,15.61) | 10.60(6.73,15.94) | 0.03(0.02,0.04) |
| Mali | female | 322.97(202.37,479.47) | 810.90(498.99,1228.11) | 1.51 | 8.61(5.44,12.71) | 8.67(5.46,13.10) | 0.03(0.02,0.03) |
| Malta | female | 20.54(13.09,30.74) | 25.43(16.45,37.73) | 0.24 | 10.16(6.45,15.24) | 10.17(6.50,15.01) | 0.00(-0.01,0.00) |
| Marshall Islands | female | 1.98(1.24,2.92) | 2.91(1.85,4.34) | 0.47 | 10.54(6.63,15.43) | 10.48(6.64,15.59) | -0.01(-0.02,-0.01) |
| Mauritania | female | 78.47(50.01,118.11) | 164.02(103.68,248.90) | 1.09 | 8.69(5.57,13.02) | 8.74(5.55,13.13) | 0.01(0.00,0.01) |
| Mauritius | female | 59.85(37.93,90.42) | 73.73(47.32,108.86) | 0.23 | 10.56(6.70,15.83) | 10.54(6.69,15.65) | 0.00(-0.01,0.00) |
| Mexico | female | 3744.51(2350.34,5549.18) | 6199.94(3913.05,9211.00) | 0.66 | 9.31(5.85,13.71) | 9.32(5.88,13.84) | 0.00(0.00,0.00) |
| Micronesia (Federated States of) | female | 4.64(2.89,6.94) | 5.23(3.29,7.88) | 0.13 | 10.55(6.61,15.70) | 10.52(6.63,15.82) | 0.00(-0.01,0.00) |
| Monaco | female | 1.88(1.19,2.78) | 2.20(1.38,3.27) | 0.17 | 10.20(6.45,15.37) | 10.17(6.33,15.21) | 0.00(-0.01,0.01) |
| Mongolia | female | 89.99(56.48,133.74) | 167.46(104.33,248.11) | 0.86 | 9.30(5.83,13.76) | 9.32(5.83,13.76) | 0.01(0.01,0.02) |
| Montenegro | female | 30.16(19.45,44.35) | 30.97(19.68,46.51) | 0.03 | 9.34(5.99,13.74) | 9.30(5.85,13.89) | -0.01(-0.01,0.00) |
| Morocco | female | 973.53(595.78,1463.44) | 1540.80(960.63,2293.40) | 0.58 | 8.25(5.12,12.36) | 8.23(5.14,12.20) | 0.00(-0.01,0.00) |
| Mozambique | female | 501.75(316.48,751.83) | 1130.52(715.63,1698.78) | 1.25 | 8.51(5.43,12.81) | 8.54(5.39,12.80) | 0.01(0.00,0.02) |
| Myanmar | female | 2039.70(1291.70,3080.28) | 3107.67(1954.68,4671.70) | 0.52 | 10.52(6.66,15.62) | 10.57(6.64,15.85) | 0.01(0.01,0.02) |
| Namibia | female | 55.51(34.73,83.00) | 104.47(66.76,157.57) | 0.88 | 8.67(5.44,12.94) | 8.63(5.52,12.94) | 0.00(-0.01,0.00) |
| Nauru | female | 0.47(0.30,0.73) | 0.54(0.34,0.81) | 0.13 | 10.57(6.61,16.04) | 10.53(6.74,15.82) | -0.01(-0.01,0.00) |
| Nepal | female | 702.78(446.50,1054.37) | 1329.58(840.50,1990.22) | 0.89 | 8.10(5.15,12.19) | 8.15(5.12,12.29) | 0.02(0.01,0.03) |
| Netherlands | female | 852.31(538.94,1286.29) | 977.22(627.22,1456.68) | 0.15 | 10.22(6.46,15.44) | 10.18(6.49,15.47) | 0.00(-0.01,0.01) |
| New Zealand | female | 212.10(133.56,315.32) | 293.06(184.17,434.16) | 0.38 | 11.64(7.34,17.30) | 11.70(7.38,17.54) | 0.02(0.01,0.02) |
| Nicaragua | female | 156.28(98.65,233.81) | 310.74(197.38,461.73) | 0.99 | 9.25(5.85,13.68) | 9.27(5.91,13.77) | 0.01(0.00,0.01) |
| Niger | female | 286.30(178.94,428.71) | 814.53(517.82,1224.19) | 1.85 | 8.66(5.50,12.95) | 8.71(5.57,12.92) | 0.01(0.01,0.02) |
| Nigeria | female | 3325.39(2085.94,4996.39) | 8645.81(5484.16,13001.19) | 1.60 | 8.71(5.52,12.97) | 8.71(5.57,13.06) | 0.01(0.00,0.01) |
| Niue | female | 0.11(0.07,0.16) | 0.09(0.06,0.13) | -0.21 | 10.57(6.64,15.62) | 10.53(6.69,15.79) | -0.01(-0.02,-0.01) |
| North Macedonia | female | 96.07(59.43,145.73) | 108.15(68.47,160.71) | 0.13 | 9.30(5.74,14.07) | 9.31(5.87,13.91) | 0.01(0.00,0.01) |
| Northern Mariana Islands | female | 2.48(1.55,3.74) | 2.26(1.42,3.38) | -0.09 | 10.59(6.71,15.89) | 10.56(6.63,15.80) | -0.01(-0.02,-0.01) |
| Norway | female | 237.04(149.08,350.20) | 297.22(188.59,436.42) | 0.25 | 10.21(6.40,15.14) | 10.21(6.47,15.03) | 0.01(0.00,0.01) |
| Oman | female | 55.40(34.42,84.52) | 144.13(91.86,219.98) | 1.60 | 8.25(5.15,12.48) | 8.26(5.25,12.56) | 0.00(0.00,0.01) |
| Pakistan | female | 3708.00(2328.92,5536.88) | 8318.72(5322.50,12411.74) | 1.24 | 8.14(5.15,12.04) | 8.17(5.22,12.14) | 0.01(0.00,0.01) |
| Palau | female | 0.80(0.51,1.21) | 0.91(0.58,1.36) | 0.14 | 10.54(6.77,16.01) | 10.52(6.72,15.95) | -0.01(-0.02,0.00) |
| Palestine | female | 70.14(44.24,106.16) | 189.73(119.46,288.75) | 1.71 | 8.24(5.21,12.30) | 8.22(5.27,12.39) | 0.00(-0.01,0.00) |
| Panama | female | 105.42(67.34,157.90) | 193.25(121.52,293.02) | 0.83 | 9.29(5.94,13.84) | 9.27(5.82,14.05) | 0.01(0.00,0.01) |
| Papua New Guinea | female | 187.66(118.31,282.45) | 481.79(304.84,727.46) | 1.57 | 10.48(6.60,15.66) | 10.49(6.72,15.75) | 0.01(0.01,0.02) |
| Paraguay | female | 167.44(106.32,249.29) | 324.95(204.28,484.47) | 0.94 | 9.25(5.85,13.70) | 9.27(5.83,13.82) | 0.01(0.00,0.01) |
| Peru | female | 938.82(596.37,1406.40) | 1629.03(1028.55,2403.97) | 0.74 | 9.27(5.89,13.87) | 9.28(5.86,13.71) | 0.01(0.01,0.02) |
| Philippines | female | 3056.40(1932.33,4558.57) | 5821.69(3683.62,8696.41) | 0.90 | 10.58(6.64,15.73) | 10.61(6.68,15.81) | 0.01(0.01,0.01) |
| Poland | female | 1891.02(1194.35,2799.73) | 1993.49(1263.97,2942.07) | 0.05 | 9.32(5.91,13.82) | 9.37(5.92,13.83) | 0.02(0.02,0.02) |
| Portugal | female | 574.28(361.53,852.77) | 644.66(405.26,967.65) | 0.12 | 10.14(6.41,15.05) | 10.16(6.36,15.42) | 0.01(0.00,0.01) |
| Puerto Rico | female | 176.14(112.10,266.64) | 179.41(115.13,265.31) | 0.02 | 9.28(5.93,14.08) | 9.27(5.95,13.63) | 0.00(-0.01,0.00) |
| Qatar | female | 11.86(7.51,17.98) | 68.46(42.12,102.88) | 4.77 | 8.23(5.22,12.41) | 8.23(5.16,12.25) | 0.00(-0.01,0.01) |
| Republic of Korea | female | 2671.83(1659.25,3965.64) | 3609.20(2323.75,5372.30) | 0.35 | 11.63(7.17,17.27) | 11.66(7.52,17.58) | 0.01(0.00,0.02) |
| Republic of Moldova | female | 222.59(141.86,334.40) | 195.93(125.22,288.76) | -0.12 | 9.27(5.88,13.86) | 9.29(5.89,13.80) | 0.01(0.00,0.01) |
| Romania | female | 1133.48(723.11,1694.23) | 958.35(606.24,1409.06) | -0.15 | 9.31(5.89,13.86) | 9.30(5.91,13.78) | 0.00(0.00,0.01) |
| Russian Federation | female | 7841.68(4994.22,11590.10) | 7747.80(4897.65,11383.82) | -0.01 | 9.30(5.92,13.81) | 9.33(5.85,13.86) | 0.01(0.01,0.01) |
| Rwanda | female | 267.70(168.59,400.85) | 527.39(336.72,788.54) | 0.97 | 8.57(5.42,12.84) | 8.56(5.49,12.77) | 0.02(0.01,0.04) |
| Saint Kitts and Nevis | female | 1.87(1.16,2.80) | 2.98(1.88,4.43) | 0.60 | 9.28(5.75,13.89) | 9.29(5.86,13.94) | 0.00(0.00,0.01) |
| Saint Lucia | female | 6.02(3.81,8.94) | 8.82(5.65,13.09) | 0.46 | 9.26(5.85,13.65) | 9.25(5.91,13.74) | 0.00(-0.01,0.00) |
| Saint Vincent and the Grenadines | female | 4.71(2.97,7.01) | 5.34(3.36,8.02) | 0.14 | 9.28(5.81,13.77) | 9.24(5.80,13.86) | -0.01(-0.02,0.00) |
| Samoa | female | 7.36(4.62,10.93) | 10.21(6.51,15.52) | 0.39 | 10.56(6.70,15.57) | 10.53(6.73,15.94) | -0.01(-0.01,0.00) |
| San Marino | female | 1.30(0.81,1.94) | 1.94(1.22,2.88) | 0.49 | 10.18(6.36,15.12) | 10.17(6.41,15.15) | 0.00(-0.01,0.01) |
| Sao Tome and Principe | female | 4.47(2.87,6.67) | 8.53(5.32,12.78) | 0.91 | 8.72(5.60,13.04) | 8.73(5.44,13.07) | 0.00(0.00,0.01) |
| Saudi Arabia | female | 504.21(314.03,759.43) | 1420.43(887.40,2154.37) | 1.82 | 8.26(5.19,12.29) | 8.25(5.18,12.40) | -0.01(-0.01,0.00) |
| Senegal | female | 283.30(178.88,426.23) | 603.53(381.87,913.33) | 1.13 | 8.64(5.47,12.92) | 8.68(5.52,13.06) | 0.01(0.00,0.02) |
| Serbia | female | 462.54(291.84,698.76) | 432.32(270.24,642.97) | -0.07 | 9.30(5.89,13.92) | 9.30(5.80,13.83) | 0.00(0.00,0.01) |
| Seychelles | female | 3.74(2.39,5.56) | 5.38(3.41,8.02) | 0.44 | 10.59(6.76,15.76) | 10.58(6.72,15.82) | 0.00(-0.01,0.01) |
| Sierra Leone | female | 143.45(91.29,215.26) | 334.42(212.49,505.14) | 1.33 | 8.66(5.43,12.85) | 8.69(5.52,12.94) | 0.01(0.01,0.02) |
| Singapore | female | 193.49(123.62,290.60) | 389.39(245.04,572.40) | 1.01 | 11.65(7.41,17.57) | 11.74(7.44,17.29) | 0.02(0.01,0.02) |
| Slovakia | female | 259.44(163.91,385.98) | 279.63(179.36,416.11) | 0.08 | 9.30(5.89,13.89) | 9.32(6.04,13.86) | 0.01(0.00,0.01) |
| Slovenia | female | 99.61(62.91,148.20) | 100.55(64.16,150.79) | 0.01 | 9.28(5.85,13.81) | 9.29(5.87,13.90) | 0.01(0.00,0.01) |
| Solomon Islands | female | 14.59(9.05,21.71) | 31.83(20.40,47.77) | 1.18 | 10.50(6.68,15.50) | 10.53(6.74,15.70) | 0.00(-0.01,0.01) |
| Somalia | female | 250.35(159.10,384.38) | 726.02(462.09,1099.85) | 1.90 | 8.51(5.43,12.98) | 8.56(5.43,12.84) | 0.02(0.01,0.03) |
| South Africa | female | 1588.27(1003.52,2376.63) | 2575.32(1635.12,3831.42) | 0.62 | 8.69(5.48,12.99) | 8.65(5.51,12.95) | -0.02(-0.02,-0.01) |
| South Sudan | female | 201.30(127.79,303.22) | 344.65(217.57,518.38) | 0.71 | 8.50(5.42,12.60) | 8.51(5.36,12.77) | 0.01(0.01,0.02) |
| Spain | female | 2175.54(1370.51,3238.24) | 2699.09(1729.08,3986.71) | 0.24 | 10.20(6.44,15.13) | 10.19(6.54,15.12) | -0.01(-0.01,0.00) |
| Sri Lanka | female | 902.31(562.77,1359.18) | 1247.30(792.33,1864.84) | 0.38 | 10.55(6.56,15.87) | 10.56(6.65,15.79) | 0.01(0.00,0.01) |
| Sudan | female | 718.36(457.65,1085.57) | 1577.74(999.38,2363.87) | 1.20 | 8.23(5.26,12.25) | 8.24(5.19,12.42) | 0.00(0.00,0.01) |
| Suriname | female | 17.00(10.75,25.09) | 27.66(17.40,41.24) | 0.63 | 9.24(5.84,13.64) | 9.22(5.76,13.68) | -0.01(-0.02,0.00) |
| Sweden | female | 484.94(305.95,723.27) | 567.49(365.00,840.58) | 0.17 | 10.23(6.51,15.38) | 10.24(6.57,15.18) | 0.01(0.00,0.01) |
| Switzerland | female | 394.66(252.12,587.84) | 506.15(323.78,761.65) | 0.28 | 10.14(6.43,15.25) | 10.19(6.45,15.27) | 0.01(0.00,0.01) |
| Syrian Arab Republic | female | 435.00(276.30,656.85) | 612.28(387.51,938.08) | 0.41 | 8.25(5.21,12.34) | 8.23(5.26,12.63) | 0.00(-0.01,0.01) |
| Taiwan (Province of China) | female | 1073.31(685.17,1618.33) | 1362.13(856.01,2042.66) | 0.27 | 10.27(6.55,15.46) | 10.31(6.48,15.44) | 0.01(0.00,0.01) |
| Tajikistan | female | 221.59(140.58,334.75) | 433.73(272.69,654.82) | 0.96 | 9.34(5.93,13.91) | 9.30(5.87,14.01) | 0.00(-0.01,0.00) |
| Thailand | female | 3124.63(1997.29,4751.84) | 4182.43(2628.40,6207.91) | 0.34 | 10.59(6.83,16.04) | 10.60(6.64,15.71) | 0.01(0.01,0.02) |
| Timor-Leste | female | 36.18(22.93,54.39) | 64.16(40.38,96.06) | 0.77 | 10.52(6.71,15.71) | 10.56(6.61,15.68) | 0.03(0.01,0.04) |
| Togo | female | 138.69(85.89,208.01) | 332.45(213.21,494.41) | 1.40 | 8.67(5.43,12.95) | 8.67(5.53,12.76) | 0.01(0.00,0.02) |
| Tokelau | female | 0.08(0.05,0.12) | 0.07(0.04,0.10) | -0.13 | 10.56(6.70,15.92) | 10.54(6.59,15.65) | 0.00(-0.01,0.00) |
| Tonga | female | 4.53(2.84,6.79) | 5.15(3.28,7.70) | 0.14 | 10.57(6.63,15.81) | 10.54(6.69,15.72) | 0.00(-0.01,0.01) |
| Trinidad and Tobago | female | 54.69(34.43,82.50) | 68.83(43.68,102.71) | 0.26 | 9.25(5.84,13.93) | 9.26(5.89,13.94) | 0.01(0.00,0.01) |
| Tunisia | female | 322.87(199.35,484.35) | 517.86(327.73,766.67) | 0.60 | 8.29(5.14,12.43) | 8.27(5.20,12.20) | 0.00(0.00,0.01) |
| Turkey | female | 2301.54(1437.32,3461.30) | 3630.60(2326.87,5453.60) | 0.58 | 8.25(5.15,12.31) | 8.24(5.28,12.47) | 0.00(-0.01,0.00) |
| Turkmenistan | female | 159.72(100.18,238.29) | 233.93(147.28,353.03) | 0.46 | 9.28(5.87,13.84) | 9.33(5.89,14.05) | 0.01(0.00,0.01) |
| Tuvalu | female | 0.51(0.32,0.75) | 0.60(0.38,0.90) | 0.18 | 10.54(6.70,15.78) | 10.56(6.65,15.98) | 0.00(0.00,0.01) |
| Uganda | female | 618.44(388.04,931.21) | 1552.03(972.40,2356.51) | 1.51 | 8.52(5.39,12.85) | 8.59(5.48,12.97) | 0.04(0.03,0.05) |
| Ukraine | female | 2743.40(1727.03,4147.43) | 2388.89(1521.73,3585.18) | -0.13 | 9.32(5.87,14.23) | 9.35(5.97,14.08) | 0.01(0.00,0.02) |
| United Arab Emirates | female | 51.72(33.27,77.22) | 246.78(155.06,369.47) | 3.77 | 8.24(5.28,12.28) | 8.25(5.26,12.29) | 0.00(-0.01,0.00) |
| United Kingdom | female | 3275.94(2077.70,4861.60) | 3807.67(2420.34,5632.49) | 0.16 | 10.17(6.43,15.08) | 10.17(6.45,15.05) | 0.00(0.00,0.01) |
| United Republic of Tanzania | female | 952.60(592.51,1451.56) | 2238.95(1418.24,3356.85) | 1.35 | 8.42(5.34,12.75) | 8.59(5.44,12.89) | 0.06(0.05,0.08) |
| United States of America | female | 22921.02(14507.66,34073.35) | 29830.11(18934.89,43979.38) | 0.30 | 16.46(10.39,24.54) | 16.24(10.35,24.02) | -0.27(-0.38,-0.17) |
| United States Virgin Islands | female | 5.16(3.32,7.56) | 5.21(3.27,7.64) | 0.01 | 9.31(5.96,13.67) | 9.27(5.82,13.65) | -0.01(-0.01,0.00) |
| Uruguay | female | 194.01(123.80,289.92) | 224.23(140.77,332.32) | 0.16 | 11.72(7.44,17.56) | 11.69(7.40,17.42) | 0.00(-0.01,0.00) |
| Uzbekistan | female | 890.52(556.89,1327.31) | 1615.11(1028.12,2443.07) | 0.81 | 9.27(5.81,13.82) | 9.27(5.91,14.05) | 0.01(0.01,0.02) |
| Vanuatu | female | 6.86(4.25,10.37) | 14.57(9.17,22.12) | 1.12 | 10.52(6.47,15.86) | 10.50(6.66,16.00) | -0.01(-0.01,0.00) |
| Venezuela (Bolivarian Republic of) | female | 833.58(534.14,1254.74) | 1390.10(872.29,2084.10) | 0.67 | 9.28(5.94,13.89) | 9.30(5.84,14.01) | 0.01(0.00,0.01) |
| Viet Nam | female | 3480.61(2204.03,5237.76) | 5644.25(3562.03,8411.12) | 0.62 | 10.58(6.65,15.85) | 10.63(6.68,15.86) | 0.02(0.01,0.02) |
| Yemen | female | 446.62(282.58,673.49) | 1178.89(760.68,1764.05) | 1.64 | 8.18(5.23,12.23) | 8.17(5.26,12.23) | 0.00(-0.01,0.01) |
| Zambia | female | 288.29(183.48,437.26) | 713.05(447.58,1063.04) | 1.47 | 8.55(5.57,12.84) | 8.56(5.40,12.59) | 0.01(0.00,0.01) |
| Zimbabwe | female | 390.24(245.37,596.28) | 632.24(393.32,931.31) | 0.62 | 8.68(5.56,13.12) | 8.64(5.38,12.71) | 0.00(-0.01,0.01) |

**Supplementary table 6. Age distribution of incidence rate for alopecia areata in different countries in 2019.**

| **2019incidence rate** | **15 to 19** | **20 to 24** | **25 to 29** | **30 to 34** | **35 to 39** | **40 to 44** | **45 to 49** | **50 to 54** | **55 to 59** | **60 to 64** | **65 to 69** | **70 to 74** | **75 to 79** | **80 plus** | **80-84** | **85-89** | **90-94** | **all ages** |
| --- | --- | --- | --- | --- | --- | --- | --- | --- | --- | --- | --- | --- | --- | --- | --- | --- | --- | --- |
| Afghanistan | 272.9929 | 436.4981 | 578.9564 | 598.5966 | 551.1805 | 461.7959 | 405.4432 | 359.2481 | 364.0655 | 372.4044 | 355.7899 | 321.2423 | 248.809 | 177.6424 | 200.802 | 148.5609 | 91.91183 | 294.3344 |
| Albania | 304.206 | 484.6208 | 639.2817 | 666.9698 | 625.6145 | 534.0463 | 467.5687 | 410.1578 | 402.0848 | 404.641 | 383.4603 | 351.5369 | 280.2867 | 202.2479 | 231.4989 | 178.7321 | 118.8991 | 404.7865 |
| Algeria | 273.7554 | 437.7099 | 582.0428 | 604.7559 | 556.8496 | 469.915 | 411.8129 | 363.0116 | 354.9797 | 358.3421 | 339.1875 | 311.0019 | 244.5899 | 180.6693 | 201.9492 | 146.8088 | 81.00818 | 356.6795 |
| American Samoa | 356.5495 | 572.1325 | 758.0017 | 797.2573 | 726.225 | 611.2857 | 533.8867 | 471.5106 | 468.3043 | 471.9792 | 445.6634 | 404.1015 | 322.2906 | 230.8535 | 266.2517 | 205.1804 | 132.7169 | 431.4991 |
| Andorra | 380.4756 | 622.7647 | 823.2789 | 840.3245 | 768.6377 | 650.7026 | 558.9896 | 498.7732 | 497.5086 | 495.2246 | 457.3015 | 401.2188 | 333.0141 | 210.8625 | 269.3975 | 205.7145 | 129.5315 | 518.7028 |
| Angola | 295.588 | 469.5783 | 628.3457 | 653.9728 | 601.3767 | 505.3314 | 439.596 | 389.0613 | 387.5518 | 389.297 | 369.7084 | 342.6411 | 270.6634 | 198.497 | 222.9292 | 170.0813 | 109.4244 | 314.5346 |
| Antigua and Barbuda | 306.5074 | 488.9496 | 650.5907 | 687.8778 | 633.4966 | 533.4442 | 466.8596 | 409.9229 | 405.1542 | 407.0151 | 384.899 | 352.7148 | 279.9581 | 199.5999 | 233.5527 | 180.8835 | 116.3517 | 419.1437 |
| Argentina | 397.8878 | 638.2281 | 850.1083 | 882.064 | 819.0423 | 702.9987 | 614.0705 | 558.0585 | 569.2567 | 587.958 | 549.0245 | 494.2959 | 398.551 | 269.0211 | 323.3661 | 246.738 | 151.9432 | 530.2787 |
| Armenia | 302.2454 | 486.7592 | 652.8658 | 684.9503 | 629.2694 | 532.2366 | 467.6409 | 415.5977 | 410.7152 | 416.4519 | 397.8397 | 368.5918 | 295.5624 | 212.2401 | 243.5795 | 181.6734 | 115.5726 | 417.528 |
| Australia | 397.1247 | 637.9803 | 849.9573 | 881.9921 | 816.5516 | 700.5554 | 613.1337 | 554.9636 | 563.1604 | 578.2585 | 532.8116 | 468.0981 | 370.1412 | 242.9949 | 297.6338 | 226.9979 | 144.1982 | 535.2168 |
| Austria | 380.2219 | 621.9441 | 823.1092 | 840.261 | 766.4508 | 655.6182 | 567.2514 | 506.2276 | 510.2471 | 522.4209 | 487.3799 | 437.7383 | 351.6819 | 232.2592 | 283.7308 | 219.4103 | 136.213 | 510.4019 |
| Azerbaijan | 302.6251 | 487.6024 | 649.616 | 677.343 | 621.4877 | 526.0319 | 463.2248 | 411.677 | 406.8534 | 412.7742 | 395.417 | 366.7102 | 293.2935 | 224.9958 | 241.7482 | 181.0439 | 112.2203 | 419.3011 |
| Bahamas | 309.1941 | 491.7873 | 653.2964 | 686.1334 | 630.9667 | 531.3601 | 463.5753 | 409.9379 | 406.6604 | 412.9941 | 391.8983 | 362.1943 | 288.6618 | 199.248 | 235.8001 | 179.2101 | 117.0414 | 415.9016 |
| Bahrain | 273.0643 | 433.2293 | 564.7277 | 562.8467 | 482.7511 | 406.835 | 367.1228 | 327.6216 | 323.4271 | 326.498 | 311.3972 | 300.417 | 246.9893 | 188.8472 | 206.6972 | 153.4831 | 97.56978 | 370.9918 |
| Bangladesh | 275.4216 | 443.4481 | 586.2037 | 608.2101 | 557.3604 | 473.7642 | 411.9797 | 359.3645 | 352.3719 | 351.7685 | 331.9459 | 301.1749 | 237.3471 | 169.8317 | 194.2066 | 144.4866 | 90.14125 | 351.1064 |
| Barbados | 306.6495 | 490.953 | 652.5504 | 684.3103 | 630.8594 | 530.4324 | 465.2908 | 411.7056 | 407.9538 | 411.986 | 390.5493 | 359.827 | 286.8938 | 197.5462 | 234.9723 | 178.2173 | 113.5663 | 411.2848 |
| Belarus | 305.5127 | 486.545 | 644.0549 | 676.1142 | 624.1877 | 528.5713 | 465.4582 | 413.6638 | 411.5801 | 421.3198 | 407.6156 | 379.8947 | 310.2176 | 219.6515 | 258.7109 | 195.9071 | 125.3658 | 417.2759 |
| Belgium | 380.535 | 620.9216 | 822.111 | 841.3988 | 767.6209 | 654.8888 | 564.5961 | 504.9671 | 508.9743 | 518.3091 | 480.8121 | 432.2022 | 349.6878 | 232.1112 | 283.4635 | 218.8649 | 136.3355 | 496.5053 |
| Belize | 309.3543 | 493.6918 | 651.8686 | 683.5646 | 629.2556 | 529.4457 | 461.5847 | 405.47 | 397.819 | 401.6768 | 377.789 | 343.4679 | 270.0688 | 186.0004 | 222.4348 | 172.2168 | 110.4333 | 386.815 |
| Benin | 295.6825 | 470.2144 | 624.2226 | 648.6261 | 600.988 | 505.602 | 437.7599 | 384.1648 | 379.6345 | 385.1508 | 363.6438 | 335.9518 | 267.3884 | 194.6964 | 221.809 | 169.2548 | 108.1906 | 315.1004 |
| Bermuda | 308.1223 | 494.5749 | 654.0578 | 684.4981 | 626.1379 | 522.931 | 455.6475 | 404.163 | 403.1892 | 411.1895 | 392.1687 | 360.2163 | 288.8548 | 198.0141 | 236.3906 | 181.8531 | 120.2978 | 410.4044 |
| Bhutan | 271.1718 | 426.6412 | 562.9413 | 591.4579 | 542.6992 | 456.4729 | 399.4893 | 352.2826 | 350.0037 | 353.8318 | 336.3079 | 307.1903 | 241.8005 | 170.1525 | 196.3568 | 147.4957 | 93.2211 | 355.3608 |
| Bolivia (Plurinational State of) | 307.5121 | 489.8953 | 647.815 | 679.2065 | 624.3671 | 525.7773 | 459.0602 | 405.7832 | 402.0885 | 407.5564 | 389.0259 | 356.0158 | 281.4619 | 200.374 | 230.4695 | 173.9343 | 110.9674 | 374.8652 |
| Bosnia and Herzegovina | 306.2051 | 487.3556 | 646.1271 | 679.5921 | 622.6332 | 524.7267 | 457.5697 | 405.824 | 403.3568 | 409.7973 | 392.6384 | 363.8764 | 292.5354 | 213.8899 | 241.4875 | 182.2719 | 116.6707 | 413.7719 |
| Botswana | 293.2704 | 465.2692 | 620.5577 | 644.6165 | 589.7619 | 494.6809 | 435.1676 | 389.8769 | 390.7625 | 395.7105 | 372.421 | 345.0825 | 277.5582 | 208.0747 | 233.5871 | 179.5618 | 115.087 | 378.1276 |
| Brazil | 308.9202 | 493.8331 | 653.4928 | 685.1125 | 631.8228 | 534.2259 | 465.4535 | 412.6386 | 407.5184 | 414.0463 | 394.5532 | 361.441 | 288.8334 | 199.9003 | 240.0942 | 181.6917 | 115.4776 | 413.5005 |
| Brunei Darussalam | 392.743 | 635.0243 | 840.5788 | 865.1478 | 793.8388 | 677.5206 | 597.9499 | 542.071 | 545.4394 | 566.8854 | 534.4758 | 482.6936 | 395.0045 | 321.7148 | 342.3727 | 295.0121 | 179.0072 | 554.7296 |
| Bulgaria | 305.5361 | 486.3663 | 642.7723 | 672.4951 | 615.914 | 519.7558 | 454.9553 | 403.6399 | 401.7253 | 410.9206 | 396.2078 | 368.2119 | 295.3551 | 212.5841 | 244.816 | 185.0137 | 118.0966 | 408.7267 |
| Burkina Faso | 294.7665 | 471.1644 | 631.8157 | 659.0324 | 604.6137 | 507.2739 | 444.3142 | 391.8525 | 387.702 | 391.278 | 367.9084 | 339.7223 | 266.3508 | 192.3351 | 219.8117 | 166.6093 | 106.6376 | 315.2714 |
| Burundi | 298.3026 | 469.3591 | 622.0764 | 642.9698 | 581.2065 | 489.5018 | 424.7495 | 374.5751 | 369.5633 | 369.893 | 349.8208 | 318.1649 | 252.6599 | 186.8929 | 210.7431 | 161.9244 | 104.1707 | 314.1579 |
| Cabo Verde | 293.0649 | 460.445 | 608.3542 | 630.257 | 578.801 | 488.0699 | 429.347 | 382.6743 | 383.575 | 394.866 | 376.7404 | 347.7524 | 276.4983 | 190.2647 | 228.3772 | 170.1344 | 105.848 | 373.3054 |
| Cambodia | 381.9865 | 611.6903 | 807.5497 | 846.6398 | 774.2741 | 657.8765 | 576.7316 | 510.4448 | 507.2015 | 517.8223 | 493.9247 | 447.2089 | 356.7653 | 255.1796 | 290.7454 | 220.3329 | 140.6282 | 484.5856 |
| Cameroon | 293.9557 | 467.2399 | 623.1856 | 648.2689 | 593.8005 | 495.8562 | 433.0906 | 383.5731 | 379.2491 | 381.6522 | 360.058 | 331.8287 | 264.2857 | 194.9437 | 220.4296 | 167.6207 | 107.317 | 331.1516 |
| Canada | 445.5739 | 706.3829 | 950.3927 | 1004.822 | 963.486 | 835.4677 | 741.515 | 685.6044 | 706.797 | 735.3408 | 678.2973 | 598.3743 | 473.9054 | 305.1486 | 377.1528 | 287.1023 | 181.4452 | 639.127 |
| Central African Republic | 294.9893 | 470.2581 | 628.9532 | 654.7777 | 597.3369 | 496.9721 | 429.431 | 380.8325 | 380.506 | 390.1201 | 372.6643 | 349.1469 | 281.2672 | 211.3799 | 234.5592 | 176.9774 | 111.3266 | 326.6662 |
| Chad | 295.287 | 470.0767 | 628.3739 | 657.7055 | 604.1425 | 502.7126 | 434.2637 | 376.2462 | 368.1036 | 367.7844 | 344.4042 | 315.4684 | 249.4307 | 182.0668 | 206.7802 | 157.0164 | 104.4114 | 292.4688 |
| Chile | 397.2856 | 637.9742 | 849.138 | 879.1366 | 816.6545 | 702.1522 | 614.944 | 559.6762 | 570.8011 | 588.9022 | 546.2283 | 486.6454 | 390.6087 | 259.3732 | 313.2767 | 235.2973 | 142.7522 | 545.2556 |
| China | 335.9231 | 539.7649 | 719.9852 | 755.0303 | 689.138 | 581.7235 | 508.548 | 449.3632 | 442.381 | 447.3931 | 420.5392 | 382.3751 | 306.1867 | 228.4168 | 254.3815 | 201.2475 | 139.4227 | 468.7827 |
| Colombia | 306.3765 | 489.7951 | 650.158 | 681.6641 | 628.6878 | 531.7315 | 466.8565 | 412.9952 | 408.7381 | 413.5999 | 392.147 | 359.0498 | 285.1434 | 189.7536 | 234.4561 | 176.495 | 111.698 | 403.0561 |
| Comoros | 294.3848 | 461.3245 | 612.8572 | 638.0944 | 583.7185 | 495.4911 | 430.6285 | 382.5236 | 382.9315 | 388.1092 | 368.6333 | 335.0722 | 262.6931 | 186.8132 | 214.6927 | 161.7846 | 103.0216 | 354.6668 |
| Congo | 295.0607 | 467.5065 | 623.5391 | 650.4745 | 595.7486 | 494.2762 | 426.8498 | 375.6814 | 374.6237 | 383.7161 | 367.1615 | 337.6405 | 265.5536 | 196.8116 | 220.3629 | 166.9566 | 105.6852 | 344.6543 |
| Cook Islands | 362.7328 | 584.7117 | 773.1635 | 809.1485 | 736.8261 | 625.7135 | 540.2751 | 474.8411 | 458.7628 | 470.9496 | 438.7471 | 405.4351 | 319.263 | 226.8278 | 268.9642 | 197.96 | 127.7601 | 452.2147 |
| Costa Rica | 308.4882 | 493.5275 | 655.4773 | 687.9608 | 633.1431 | 532.8913 | 465.7654 | 411.4773 | 407.8335 | 411.7293 | 390.7397 | 356.6905 | 282.5689 | 188.0123 | 232.1939 | 176.3344 | 112.8044 | 412.134 |
| Côte d'Ivoire | 290.7474 | 463.0174 | 619.8822 | 642.727 | 583.2925 | 482.0544 | 419.2142 | 372.5156 | 370.7214 | 373.233 | 351.6457 | 324.571 | 258.3482 | 189.1799 | 214.8113 | 164.6664 | 106.2476 | 330.9227 |
| Croatia | 305.987 | 488.0118 | 644.647 | 675.0318 | 619.039 | 522.0759 | 457.6448 | 405.8543 | 402.7393 | 408.6149 | 390.9068 | 364.4423 | 293.9463 | 212.4952 | 244.7997 | 187.7081 | 121.4201 | 407.9409 |
| Cuba | 305.3058 | 485.7159 | 642.3007 | 673.3564 | 619.7209 | 524.0435 | 459.9735 | 407.2132 | 403.4283 | 408.4139 | 388.1368 | 355.6402 | 283.2181 | 188.9936 | 231.8295 | 175.1849 | 112.1228 | 407.0774 |
| Cyprus | 380.4212 | 621.9116 | 823.6383 | 840.3983 | 771.9928 | 668.0318 | 581.4198 | 518.3225 | 515.9202 | 513.9571 | 473.1308 | 421.9112 | 339.4504 | 245.5805 | 274.309 | 211.3445 | 134.0923 | 536.4138 |
| Czechia | 305.7931 | 487.4978 | 644.2795 | 673.1543 | 616.936 | 520.0643 | 454.6904 | 402.2133 | 399.0615 | 406.9368 | 390.6802 | 362.228 | 291.0597 | 202.9195 | 242.9216 | 186.2701 | 120.3046 | 405.5153 |
| Democratic People's Republic of Korea | 339.9831 | 541.2867 | 710.354 | 734.4022 | 669.1132 | 568.4583 | 504.7571 | 448.3798 | 446.2317 | 455.3324 | 435.0854 | 410.2802 | 337.9868 | 253.6922 | 282.8869 | 221.738 | 141.1768 | 457.8851 |
| Democratic Republic of the Congo | 293.2429 | 462.6767 | 614.6429 | 639.456 | 588.513 | 494.3515 | 431.9509 | 382.7943 | 381.4167 | 388.3853 | 371.0371 | 346.6079 | 276.7981 | 202.3299 | 230.2422 | 174.5439 | 110.8681 | 321.5452 |
| Denmark | 380.5071 | 621.3618 | 822.8778 | 839.9216 | 766.4718 | 655.5135 | 565.7407 | 505.4873 | 508.5958 | 516.7449 | 479.1149 | 425.8907 | 341.8266 | 228.4058 | 276.2514 | 213.653 | 134.6271 | 496.0288 |
| Djibouti | 284.7371 | 449.1571 | 607.6767 | 646.6043 | 590.2636 | 493.4664 | 422.3453 | 367.192 | 360.9805 | 361.5235 | 345.6307 | 313.3045 | 252.1146 | 188.1918 | 210.3938 | 161.4519 | 103.8654 | 352.4033 |
| Dominica | 305.3858 | 485.5 | 644.7695 | 677.8267 | 619.6535 | 519.5344 | 454.4648 | 399.5833 | 390.9132 | 391.7136 | 379.625 | 354.9996 | 280.0975 | 200.1859 | 231.5433 | 181.655 | 117.7166 | 395.7866 |
| Dominican Republic | 307.8738 | 490.7748 | 647.7401 | 675.1516 | 618.7575 | 521.8946 | 457.4239 | 404.9279 | 400.6192 | 405.3902 | 386.073 | 355.1405 | 282.1782 | 195.7339 | 231.7755 | 174.1263 | 109.8484 | 389.0913 |
| Ecuador | 307.1775 | 489.6993 | 649.3785 | 681.5044 | 627.2775 | 528.2488 | 462.1269 | 407.9246 | 402.9591 | 406.7468 | 386.2489 | 353.1113 | 280.2571 | 199.1098 | 230.5756 | 175.5385 | 111.4614 | 386.5922 |
| Egypt | 271.686 | 436.6041 | 581.6871 | 600.4785 | 550.4051 | 463.9952 | 406.1058 | 359.393 | 352.2632 | 353.2086 | 335.66 | 300.1464 | 235.3569 | 163.6445 | 188.6832 | 134.2504 | 77.32354 | 336.8038 |
| El Salvador | 307.9804 | 495.9377 | 661.0461 | 698.2073 | 646.9353 | 548.4373 | 480.932 | 425.1171 | 420.1795 | 422.6916 | 399.3444 | 363.939 | 288.4157 | 189.2234 | 235.4122 | 177.5276 | 114.1297 | 396.4798 |
| Equatorial Guinea | 278.4996 | 440.8022 | 593.1604 | 628.1731 | 589.7477 | 504.4179 | 446.8405 | 397.8164 | 397.2101 | 401.4284 | 377.6933 | 349.319 | 272.121 | 197.6781 | 223.9642 | 169.5297 | 108.4628 | 328.5846 |
| Eritrea | 293.4332 | 458.962 | 610.2909 | 636.0748 | 582.5576 | 494.8002 | 430.5848 | 384.3304 | 386.1958 | 393.2653 | 377.9156 | 347.2981 | 275.6443 | 209.0081 | 230.4887 | 175.5941 | 111.1407 | 335.6923 |
| Estonia | 306.063 | 487.6588 | 639.3899 | 670.0367 | 617.6394 | 521.6872 | 458.4166 | 408.2809 | 407.2169 | 417.138 | 403.2746 | 375.6058 | 304.285 | 215.6909 | 254.6296 | 194.672 | 126.0007 | 407.2349 |
| Eswatini | 292.6118 | 467.6304 | 628.0519 | 654.4175 | 596.7707 | 496.3888 | 435.6065 | 391.8139 | 394.2613 | 400.7076 | 381.0043 | 358.7267 | 287.8089 | 217.6249 | 240.2235 | 182.061 | 115.186 | 355.3006 |
| Ethiopia | 295.8362 | 466.1438 | 618.7218 | 650.6334 | 596.3853 | 503.0748 | 430.4661 | 380.4621 | 377.8695 | 379.3538 | 356.3996 | 318.6047 | 250.3185 | 179.9814 | 204.8092 | 154.513 | 98.31937 | 323.3024 |
| Fiji | 356.7082 | 569.4858 | 752.6997 | 786.3716 | 716.5311 | 606.0564 | 530.77 | 468.9335 | 463.7804 | 470.6303 | 450.8997 | 411.6957 | 332.5965 | 249.3114 | 276.0656 | 211.813 | 140.8565 | 448.6349 |
| Finland | 380.4768 | 621.4931 | 823.0655 | 839.6108 | 763.6509 | 651.6236 | 563.3976 | 506.0758 | 509.9707 | 519.8965 | 484.1584 | 433.0842 | 350.5398 | 233.1118 | 285.3775 | 220.0886 | 136.7076 | 496.6034 |
| France | 380.4939 | 620.9974 | 821.733 | 842.4098 | 770.2609 | 657.7188 | 567.9414 | 510.3186 | 515.8647 | 526.9408 | 488.3544 | 434.9981 | 349.5705 | 227.8786 | 284.2866 | 219.684 | 136.8267 | 488.8808 |
| Gabon | 297.72 | 471.8436 | 627.4333 | 651.3485 | 598.4491 | 502.6717 | 438.8843 | 384.4354 | 376.4576 | 377.7873 | 357.4647 | 337.3835 | 275.166 | 203.5103 | 232.5263 | 177.215 | 112.4883 | 360.2478 |
| Gambia | 295.9171 | 469.4484 | 626.2998 | 652.0235 | 598.7928 | 500.519 | 433.2664 | 379.3072 | 373.0699 | 378.4067 | 361.3066 | 331.3011 | 264.9683 | 197.5055 | 220.8581 | 168.1425 | 107.0772 | 330.8582 |
| Georgia | 301.9317 | 483.7132 | 645.3044 | 678.1657 | 625.0333 | 527.1367 | 462.2247 | 411.5046 | 410.0936 | 418.489 | 402.3581 | 371.9459 | 299.6716 | 204.4574 | 247.1799 | 188.0467 | 122.5445 | 402.8433 |
| Germany | 380.1836 | 623.5243 | 824.2503 | 838.9135 | 764.7457 | 653.8564 | 564.9938 | 505.1694 | 508.459 | 518.1995 | 485.2838 | 432.8182 | 348.5119 | 240.2303 | 281.3663 | 216.2698 | 136.7818 | 503.5619 |
| Ghana | 293.4004 | 469.0363 | 628.7722 | 654.3996 | 601.9314 | 504.0149 | 440.7999 | 392.7777 | 389.5688 | 392.4443 | 370.0446 | 341.828 | 272.5548 | 198.8929 | 225.3081 | 170.9901 | 109.7193 | 354.5985 |
| Greece | 380.5455 | 620.697 | 822.3873 | 841.4427 | 768.8058 | 657.5584 | 568.3112 | 512.8743 | 519.1525 | 525.7692 | 483.7249 | 434.7808 | 348.1278 | 231.8994 | 281.6783 | 210.8267 | 125.3608 | 500.2185 |
| Greenland | 449.8123 | 705.9168 | 943.0891 | 990.4165 | 944.5104 | 788.0076 | 697.9425 | 646.8507 | 652.0647 | 647.8828 | 596.9888 | 502.0536 | 456.5596 | 324.2219 | 371.9257 | 295.8286 | 174.6874 | 612.667 |
| Grenada | 303.2812 | 483.1585 | 641.4134 | 673.0232 | 618.1209 | 520.9833 | 455.0152 | 400.0847 | 393.5718 | 397.6709 | 381.5781 | 354.4624 | 285.3724 | 213.056 | 246.1931 | 196.3501 | 128.649 | 400.9876 |
| Guam | 354.8986 | 560.393 | 742.7831 | 777.9826 | 718.0105 | 610.4405 | 528.7362 | 460.4816 | 459.2341 | 465.4646 | 448.3256 | 401.0807 | 327.0355 | 220.8074 | 272.1929 | 207.893 | 130.8254 | 439.0402 |
| Guatemala | 306.9227 | 490.9509 | 653.1513 | 690.433 | 640.8068 | 543.5859 | 476.6851 | 419.43 | 412.679 | 414.7223 | 391.9746 | 357.4412 | 282.2754 | 210.2021 | 231.5325 | 177.8825 | 118.8282 | 382.0256 |
| Guinea | 296.5503 | 476.063 | 637.4635 | 664.9834 | 611.8348 | 510.9297 | 442.1382 | 384.3017 | 376.1479 | 374.987 | 351.9909 | 323.1812 | 255.246 | 181.7164 | 211.1219 | 164.5229 | 105.4914 | 318.3997 |
| Guinea-Bissau | 294.7923 | 468.7711 | 628.1907 | 657.0412 | 605.1888 | 506.2873 | 441.7374 | 389.1819 | 383.6362 | 387.3464 | 365.2613 | 340.2022 | 271.8861 | 201.7604 | 227.0136 | 172.5522 | 109.6064 | 334.8858 |
| Guyana | 306.9436 | 490.0448 | 651.8666 | 685.484 | 629.6594 | 529.1479 | 459.7631 | 405.6644 | 402.7201 | 409.2246 | 388.7881 | 357.3602 | 285.556 | 203.5496 | 236.5816 | 180.5719 | 115.308 | 393.5337 |
| Haiti | 309.0719 | 495.1884 | 659.3317 | 694.5089 | 639.8864 | 536.6887 | 466.1997 | 410.8837 | 406.3263 | 411.0766 | 389.6554 | 354.8652 | 280.77 | 200.8693 | 229.2783 | 169.9852 | 107.9661 | 380.5322 |
| Honduras | 308.6059 | 496.252 | 661.5939 | 695.7841 | 639.0672 | 536.1285 | 466.6462 | 411.2205 | 406.4123 | 409.758 | 389.3687 | 356.415 | 281.2482 | 200.8442 | 229.3337 | 171.3797 | 107.2683 | 377.1194 |
| Hungary | 305.7639 | 486.8566 | 644.8716 | 675.9723 | 622.0414 | 523.2828 | 458.0581 | 406.1905 | 405.5047 | 414.8851 | 398.5275 | 369.9693 | 298.9684 | 210.4614 | 250.859 | 190.6113 | 122.2157 | 412.321 |
| Iceland | 380.7308 | 621.7518 | 823.7127 | 838.651 | 762.3854 | 652.4746 | 563.6943 | 508.3421 | 508.7126 | 513.6668 | 469.757 | 418.2071 | 340.114 | 216.1701 | 270.9943 | 208.0894 | 132.0471 | 498.7516 |
| India | 271.5913 | 435.0174 | 574.5132 | 599.8263 | 551.3301 | 464.642 | 405.4875 | 358.8334 | 355.3181 | 360.2729 | 341.9842 | 311.614 | 248.8052 | 181.7927 | 206.7622 | 154.5772 | 96.03584 | 350.1447 |
| Indonesia | 385.3874 | 617.0007 | 810.2375 | 849.7819 | 772.5321 | 655.3951 | 572.8004 | 504.8739 | 498.4911 | 503.6985 | 477.0507 | 435.3584 | 352.2619 | 254.9359 | 287.9775 | 217.4997 | 137.4749 | 502.4854 |
| Iran (Islamic Republic of) | 275.8556 | 439.5084 | 582.7652 | 607.7732 | 556.8866 | 469.9746 | 410.5565 | 364.8194 | 356.411 | 363.7064 | 348.7307 | 316.9343 | 247.5728 | 170.0818 | 201.4235 | 148.6823 | 92.14342 | 374.4854 |
| Iraq | 284.7308 | 454.8031 | 602.6327 | 627.0964 | 575.1834 | 484.798 | 424.627 | 375.8104 | 370.4054 | 375.8579 | 358.8406 | 326.4493 | 258.4637 | 185.2428 | 215.0069 | 162.9928 | 103.7806 | 353.5706 |
| Ireland | 380.5618 | 620.83 | 821.9212 | 843.3679 | 772.5932 | 658.5316 | 567.7043 | 508.7808 | 512.205 | 516.5221 | 475.8623 | 421.2066 | 337.4277 | 229.3497 | 273.599 | 210.8362 | 134.0783 | 495.0781 |
| Israel | 380.5038 | 621.1245 | 822.4239 | 841.037 | 767.4493 | 655.9653 | 567.6701 | 509.6123 | 516.2853 | 528.5558 | 489.0875 | 434.7784 | 347.4399 | 227.4652 | 279.3322 | 210.6879 | 129.3981 | 470.5443 |
| Italy | 383.2974 | 626.9715 | 825.0418 | 843.885 | 770.2927 | 657.7581 | 571.1218 | 513.3817 | 516.9406 | 527.953 | 488.0256 | 434.3799 | 350.2905 | 234.1351 | 284.4593 | 218.3806 | 136.5273 | 501.664 |
| Jamaica | 306.5506 | 488.6599 | 649.7588 | 685.2693 | 632.8516 | 530.8518 | 459.4272 | 402.6515 | 398.4919 | 405.5599 | 382.8302 | 349.057 | 280.6322 | 183.5238 | 233.8663 | 178.9898 | 114.692 | 408.0256 |
| Japan | 397.707 | 638.6077 | 845.3859 | 876.6759 | 811.6967 | 694.5486 | 606.812 | 549.2564 | 552.3199 | 572.8522 | 549.6288 | 496.8973 | 398.0544 | 260.2619 | 324.763 | 250.7038 | 159.7413 | 526.5999 |
| Jordan | 271.277 | 427.594 | 564.8878 | 582.7777 | 539.6484 | 454.2469 | 398.0176 | 352.4334 | 348.7745 | 354.1665 | 340.0424 | 311.9254 | 242.7755 | 176.1919 | 200.1925 | 149.5234 | 88.61766 | 334.3993 |
| Kazakhstan | 307.1398 | 490.7057 | 650.7442 | 681.3973 | 625.8823 | 529.778 | 464.1657 | 411.5011 | 409.8124 | 419.2384 | 405.6075 | 377.756 | 306.0338 | 226.9581 | 253.6961 | 193.8484 | 122.9776 | 399.6506 |
| Kenya | 296.4441 | 467.6489 | 620.2519 | 649.4521 | 593.1127 | 502.9304 | 433.6369 | 382.9416 | 379.7642 | 381.801 | 361.735 | 330.0613 | 264.4128 | 195.3994 | 222.4134 | 170.1092 | 108.1071 | 342.3042 |
| Kiribati | 359.6554 | 575.4458 | 761.279 | 799.4424 | 731.2137 | 621.8148 | 544.8042 | 477.4527 | 470.6242 | 478.3498 | 470.8817 | 434.5013 | 342.7404 | 255.8021 | 281.186 | 213.6229 | 135.8854 | 435.3988 |
| Kuwait | 272.8283 | 435.5621 | 581.4445 | 604.1526 | 542.1286 | 451.1235 | 396.8936 | 344.7936 | 338.7891 | 337.4133 | 328.2123 | 283.6233 | 229.8711 | 150.3519 | 183.4315 | 143.0317 | 89.17056 | 397.7925 |
| Kyrgyzstan | 307.0231 | 489.469 | 647.3759 | 678.9598 | 624.4321 | 528.4368 | 463.4645 | 410.7974 | 408.6118 | 416.845 | 400.3674 | 370.8456 | 293.9968 | 210.5673 | 245.7044 | 186.0104 | 117.1917 | 384.183 |
| Lao People's Democratic Republic | 383.2467 | 613.1149 | 807.6568 | 844.3666 | 769.8212 | 652.7486 | 571.4708 | 505.3969 | 498.2071 | 500.7799 | 476.6268 | 429.1077 | 346.693 | 247.9876 | 282.8603 | 215.2608 | 138.2141 | 478.2049 |
| Latvia | 306.2093 | 486.1622 | 643.316 | 673.9109 | 621.0454 | 526.94 | 463.0733 | 411.3038 | 410.8387 | 419.7534 | 406.8615 | 379.5433 | 306.8406 | 217.8046 | 255.7743 | 195.3123 | 126.5572 | 408.0424 |
| Lebanon | 270.5031 | 431.4098 | 578.2739 | 608.2033 | 565.3952 | 482.2502 | 423.4846 | 374.5168 | 366.3482 | 371.9007 | 353.0916 | 319.8004 | 252.0992 | 179.4329 | 209.2512 | 156.6115 | 99.62904 | 360.21 |
| Lesotho | 293.195 | 463.4514 | 614.5538 | 633.97 | 581.7778 | 491.7857 | 435.1713 | 391.8555 | 394.2341 | 400.9431 | 380.0131 | 355.3711 | 284.4674 | 213.7836 | 239.135 | 182.0581 | 114.6052 | 365.8453 |
| Liberia | 294.1096 | 467.2295 | 623.0614 | 650.6127 | 593.6355 | 489.4081 | 424.6061 | 373.6356 | 372.2371 | 373.7692 | 355.7781 | 329.6402 | 260.6398 | 180.9826 | 211.8534 | 156.9032 | 99.2569 | 337.4185 |
| Libya | 273.4735 | 436.476 | 580.0834 | 596.1827 | 546.8071 | 461.5479 | 405.8561 | 360.3935 | 354.4565 | 359.1595 | 343.7462 | 309.8013 | 240.7958 | 167.2803 | 198.7812 | 152.5973 | 98.07347 | 375.525 |
| Lithuania | 305.7796 | 486.9715 | 642.9638 | 674.4242 | 622.3114 | 528.8361 | 464.5928 | 412.2158 | 410.4123 | 419.978 | 406.1141 | 379.2692 | 305.4772 | 216.5352 | 254.1225 | 193.4617 | 124.7568 | 409.1342 |
| Luxembourg | 380.5012 | 621.4244 | 822.5852 | 840.6927 | 766.6136 | 653.6249 | 561.9149 | 498.0579 | 500.3761 | 511.0562 | 472.3505 | 421.944 | 348.065 | 231.9435 | 283.2007 | 218.0118 | 137.3382 | 516.7423 |
| Madagascar | 294.5423 | 462.2235 | 615.6301 | 643.069 | 589.3082 | 500.406 | 434.9976 | 383.7674 | 377.4972 | 377.7614 | 359.3545 | 327.0874 | 259.4608 | 186.9554 | 213.6534 | 160.5032 | 101.1907 | 329.6364 |
| Malawi | 296.4916 | 468.1904 | 624.2357 | 651.2538 | 595.4025 | 497.4864 | 430.0567 | 384.4194 | 384.6166 | 387.1138 | 368.0345 | 336.3205 | 267.8368 | 195.3403 | 224.8719 | 173.2228 | 111.4476 | 324.4528 |
| Malaysia | 381.9217 | 609.0396 | 799.8606 | 834.1641 | 760.7033 | 647.1095 | 566.7997 | 501.2534 | 495.8861 | 500.4403 | 476.2184 | 426.1863 | 341.5113 | 240.8729 | 278.0933 | 210.1295 | 131.4449 | 502.4862 |
| Maldives | 378.1126 | 587.3929 | 754.4493 | 782.0684 | 716.9969 | 616.2873 | 543.6233 | 487.4172 | 487.385 | 493.392 | 466.5241 | 425.2221 | 339.9697 | 233.3667 | 272.1073 | 203.4968 | 129.1238 | 523.1466 |
| Mali | 294.1047 | 468.7211 | 630.0626 | 658.4121 | 602.7222 | 499.4155 | 430.6317 | 378.572 | 373.9612 | 376.4287 | 354.1219 | 324.5006 | 254.7361 | 180.3485 | 208.4702 | 155.4012 | 98.38044 | 304.6792 |
| Malta | 380.6405 | 621.8513 | 823.2471 | 839.0476 | 763.6746 | 650.6989 | 563.4666 | 506.2737 | 506.9917 | 515.8729 | 478.4515 | 428.3127 | 345.441 | 235.048 | 280.9822 | 220.3047 | 135.7867 | 510.2368 |
| Marshall Islands | 357.8179 | 569.4755 | 748.3541 | 781.7134 | 714.6286 | 608.8523 | 533.0912 | 470.2581 | 465.5139 | 466.6146 | 438.1893 | 393.9207 | 312.4056 | 226.1987 | 253.4579 | 189.3819 | 119.732 | 440.7451 |
| Mauritania | 296.0279 | 470.3528 | 625.491 | 650.6563 | 599.5215 | 504.7958 | 440.1428 | 386.3749 | 379.6325 | 380.3133 | 356.7841 | 326.8534 | 255.6842 | 178.7641 | 208.1337 | 155.132 | 98.20125 | 331.6679 |
| Mauritius | 383.172 | 612.756 | 807.8397 | 844.0445 | 769.2058 | 652.702 | 570.1074 | 503.8222 | 498.5024 | 503.8341 | 479.5587 | 438.3136 | 351.9411 | 239.8656 | 289.2811 | 219.289 | 139.5468 | 517.3415 |
| Mexico | 309.8282 | 495.8735 | 655.1633 | 687.3784 | 635.1815 | 536.8229 | 467.7472 | 413.8155 | 407.5734 | 412.6744 | 391.5382 | 358.8731 | 284.4587 | 196.4188 | 234.2266 | 174.9204 | 109.5853 | 400.7954 |
| Micronesia (Federated States of) | 356.1315 | 567.9772 | 749.6104 | 784.5572 | 716.4088 | 607.7107 | 533.8343 | 472.4407 | 464.5202 | 466.5688 | 448.5912 | 415.3073 | 338.7727 | 245.7906 | 279.8175 | 209.1201 | 130.5322 | 438.5332 |
| Monaco | 380.4674 | 621.3716 | 823.2403 | 841.0735 | 781.4346 | 661.4851 | 572.3693 | 508.7481 | 499.7544 | 512.5563 | 484.3419 | 431.0964 | 349.6383 | 218.3975 | 271.5014 | 211.2468 | 129.6186 | 487.8849 |
| Mongolia | 306.5669 | 489.0509 | 649.4499 | 680.0755 | 624.8327 | 527.046 | 462.2993 | 410.8575 | 409.0357 | 418.5546 | 402.6561 | 371.0104 | 291.2513 | 221.7459 | 240.8814 | 187.97 | 127.466 | 398.7223 |
| Montenegro | 304.8799 | 485.674 | 642.9587 | 675.4186 | 623.1977 | 526.983 | 461.0934 | 405.7583 | 402.1969 | 406.1058 | 390.3147 | 365.0427 | 292.3774 | 213.4542 | 240.3485 | 181.6308 | 116.7118 | 408.1447 |
| Morocco | 273.9822 | 438.8907 | 584.4398 | 605.717 | 557.933 | 471.0136 | 412.6124 | 364.0181 | 356.6216 | 358.7857 | 339.8967 | 312.0602 | 251.1032 | 178.2414 | 207.9799 | 153.9739 | 93.20808 | 356.879 |
| Mozambique | 298.9715 | 473.3729 | 627.8888 | 652.3924 | 597.4322 | 506.5228 | 440.8941 | 389.358 | 384.2001 | 384.053 | 365.1767 | 335.8857 | 269.3519 | 199.6832 | 227.2485 | 174.1153 | 110.4835 | 311.642 |
| Myanmar | 383.9418 | 615.8753 | 815.0887 | 854.1516 | 779.0971 | 661.1215 | 578.8385 | 511.5766 | 506.2992 | 511.4322 | 485.4139 | 441.8132 | 353.7346 | 248.0772 | 288.2907 | 219.4168 | 140.5043 | 491.4198 |
| Namibia | 294.6363 | 465.8494 | 620.8677 | 648.1135 | 597.1822 | 501.2897 | 438.9281 | 390.6184 | 391.0464 | 397.4598 | 376.3809 | 346.9031 | 274.8465 | 199.5385 | 229.2544 | 175.085 | 112.0703 | 355.2195 |
| Nauru | 356.3747 | 569.7396 | 754.749 | 791.9498 | 722.3932 | 612.5792 | 536.8881 | 473.7779 | 472.599 | 484.413 | 476.9582 | 434.5197 | 340.4565 | 202.789 | 227.0526 | 173.7006 | 126.5808 | 426.9081 |
| Nepal | 274.8351 | 445.4492 | 599.5859 | 632.6382 | 579.1759 | 484.8407 | 417.1937 | 363.3566 | 358.8083 | 362.0671 | 345.409 | 312.0488 | 245.5583 | 177.2099 | 200.6764 | 152.2764 | 96.7729 | 347.0918 |
| Netherlands | 380.241 | 620.4933 | 822.2129 | 840.7607 | 767.195 | 656.4421 | 567.4575 | 507.2196 | 507.5893 | 516.625 | 476.4826 | 424.3147 | 346.3739 | 236.1685 | 283.6124 | 219.7368 | 138.6234 | 497.7769 |
| New Zealand | 399.7018 | 642.6004 | 853.6066 | 892.4925 | 829.0997 | 715.5103 | 625.7154 | 566.7832 | 572.2541 | 582.3476 | 536.1078 | 469.9381 | 374.0269 | 242.1691 | 298.3613 | 225.1416 | 141.9817 | 526.084 |
| Nicaragua | 306.581 | 489.8553 | 649.4886 | 681.524 | 627.6996 | 530.8808 | 467.0541 | 414.3876 | 410.1418 | 414.3047 | 393.3728 | 360.9025 | 287.4276 | 206.1574 | 236.6769 | 179.3071 | 114.7768 | 388.0513 |
| Niger | 295.9308 | 468.8441 | 620.1142 | 642.1645 | 594.4166 | 505.7886 | 445.1328 | 389.7898 | 378.7856 | 380.0857 | 363.561 | 330.0888 | 259.715 | 187.5018 | 213.3557 | 159.4913 | 100.7178 | 287.6961 |
| Nigeria | 300.0461 | 476.723 | 633.1986 | 657.0248 | 603.4387 | 506.2 | 441.6066 | 395.3375 | 393.0593 | 396.0214 | 368.7845 | 326.8336 | 260.2576 | 180.4702 | 209.8308 | 155.5441 | 100.3449 | 324.4866 |
| Niue | 350.0937 | 570.9812 | 757.6373 | 792.5643 | 721.0517 | 609.8285 | 536.9158 | 467.0789 | 456.3181 | 463.0617 | 453.5155 | 424.5482 | 340.5552 | 234.7747 | 274.8975 | 209.3638 | 134.2409 | 445.5482 |
| North Macedonia | 305.2238 | 484.7458 | 640.6739 | 669.3597 | 615.9997 | 518.5545 | 455.2907 | 401.9784 | 397.6933 | 402.5863 | 386.2612 | 358.1022 | 285.6614 | 214.7324 | 236.2788 | 178.7518 | 113.1527 | 418.7267 |
| Northern Mariana Islands | 355.3169 | 567.817 | 757.004 | 771.592 | 682.6241 | 645.8191 | 483.0511 | 495.5378 | 436.3492 | 477.627 | 430.141 | 406.8249 | 312.9005 | 232.3816 | 272.2558 | 202.5615 | 127.3546 | 459.2779 |
| Norway | 383.3396 | 626.6087 | 825.0827 | 843.086 | 766.1773 | 652.2625 | 564.4354 | 504.3745 | 504.2489 | 514.765 | 475.2825 | 423.0728 | 341.2906 | 223.0637 | 279.2511 | 213.6138 | 133.4551 | 501.5351 |
| Oman | 274.0438 | 396.7851 | 502.5335 | 517.4362 | 480.9418 | 417.6275 | 364.1518 | 323.868 | 325.3445 | 343.6816 | 344.8321 | 306.0341 | 239.7941 | 180.0505 | 200.8135 | 161.8299 | 99.64422 | 364.8293 |
| Pakistan | 272.6685 | 436.7537 | 577.6857 | 603.9914 | 554.7432 | 469.9812 | 408.5872 | 354.3367 | 346.8613 | 352.9284 | 336.1529 | 305.538 | 242.9947 | 174.9367 | 198.9121 | 149.5859 | 94.23917 | 313.2189 |
| Palau | 353.6907 | 546.983 | 706.7751 | 741.4161 | 680.2715 | 586.6838 | 517.2699 | 461.0934 | 462.2666 | 465.7096 | 446.2922 | 406.2318 | 330.1494 | 231.9837 | 271.2444 | 200.2118 | 126.4507 | 465.8544 |
| Palestine | 273.548 | 436.3171 | 579.4401 | 603.0833 | 556.4153 | 468.2374 | 408.5063 | 359.5046 | 352.5859 | 359.9206 | 342.94 | 316.3568 | 255.7344 | 186.4669 | 215.1879 | 162.7329 | 107.2782 | 323.5504 |
| Panama | 306.283 | 488.3336 | 646.6717 | 676.662 | 623.1759 | 524.7642 | 458.346 | 404.6037 | 400.3654 | 404.5077 | 383.4805 | 350.8516 | 279.4027 | 186.8017 | 229.7822 | 174.57 | 111.8468 | 384.2736 |
| Papua New Guinea | 353.3291 | 566.4848 | 754.8081 | 794.6098 | 722.7491 | 611.4719 | 530.7012 | 463.2054 | 455.0883 | 454.6959 | 428.0875 | 394.6007 | 314.8803 | 228.2355 | 259.4514 | 199.4431 | 130.4736 | 419.7952 |
| Paraguay | 306.4605 | 488.6473 | 645.6106 | 674.7697 | 619.342 | 522.2823 | 457.9128 | 404.5773 | 399.5891 | 403.9421 | 384.3527 | 354.3636 | 284.1874 | 191.8462 | 234.1249 | 178.819 | 115.1979 | 389.8701 |
| Peru | 303.1869 | 486.2272 | 649.869 | 682.3191 | 628.4209 | 529.9714 | 463.1979 | 408.4893 | 403.2703 | 407.0395 | 385.8564 | 352.0086 | 278.4907 | 186.1038 | 228.453 | 172.4662 | 109.6752 | 392.4742 |
| Philippines | 385.0103 | 615.8442 | 808.5848 | 847.1186 | 770.411 | 653.513 | 571.6951 | 504.9294 | 499.0935 | 507.0329 | 481.9554 | 440.4537 | 356.9892 | 253.0172 | 293.0882 | 220.2903 | 134.0383 | 471.8155 |
| Poland | 307.8059 | 491.3173 | 647.4049 | 677.4245 | 624.9557 | 527.8586 | 460.2634 | 408.013 | 403.7107 | 412.192 | 396.7154 | 367.216 | 297.3148 | 208.8915 | 249.8455 | 189.1615 | 121.874 | 419.8955 |
| Portugal | 380.5286 | 620.9585 | 821.9007 | 842.3163 | 772.3106 | 662.0941 | 573.6545 | 517.4553 | 522.8223 | 532.3711 | 492.7675 | 442.9965 | 355.2303 | 238.4398 | 286.8119 | 219.6986 | 135.8549 | 508.9901 |
| Puerto Rico | 307.0029 | 491.6696 | 654.4071 | 687.2201 | 633.0264 | 532.1957 | 465.2421 | 412.2326 | 409.3239 | 414.0739 | 393.273 | 360.9486 | 286.0743 | 189.7142 | 235.0623 | 179.5104 | 115.4456 | 407.5992 |
| Qatar | 258.04 | 346.7017 | 465.6294 | 493.1564 | 467.5501 | 400.1216 | 343.5326 | 300.5293 | 288.9004 | 298.9173 | 290.4524 | 266.9498 | 220.2687 | 157.4498 | 170.5522 | 117.8974 | 74.80672 | 370.6333 |
| Republic of Korea | 393.8319 | 635.259 | 840.1387 | 866.9995 | 802.1463 | 683.4783 | 598.4129 | 542.2106 | 548.3645 | 565.8178 | 535.6553 | 485.6314 | 400.2363 | 289.5694 | 333.5722 | 260.4855 | 164.5744 | 558.2302 |
| Republic of Moldova | 305.8485 | 487.9335 | 645.4233 | 674.9249 | 619.8759 | 524.0819 | 460.7565 | 413.4301 | 413.0239 | 421.7383 | 404.3989 | 373.8376 | 298.6741 | 208.4662 | 246.0197 | 186.5047 | 120.4404 | 427.0974 |
| Romania | 305.7198 | 487.1007 | 643.3232 | 673.9625 | 619.5525 | 521.0076 | 455.0493 | 402.6339 | 402.1871 | 412.3445 | 396.0451 | 366.5546 | 295.2346 | 209.7619 | 245.1389 | 185.1052 | 117.2951 | 405.0167 |
| Russian Federation | 307.9061 | 491.5417 | 648.5003 | 679.8685 | 629.9565 | 535.2222 | 466.8727 | 415.7445 | 413.4885 | 424.6448 | 410.4422 | 380.1781 | 311.7337 | 222.7039 | 260.2958 | 194.8236 | 123.1789 | 418.6 |
| Rwanda | 295.8668 | 464.0542 | 615.7901 | 643.1392 | 590.5542 | 506.7737 | 444.9571 | 392.8543 | 390.3616 | 392.2843 | 371.7475 | 342.8193 | 271.747 | 197.6222 | 224.3006 | 170.215 | 108.2623 | 343.0367 |
| Saint Kitts and Nevis | 308.7557 | 493.0292 | 651.8959 | 678.8396 | 620.7538 | 520.3391 | 452.5375 | 399.49 | 398.3735 | 406.3406 | 383.7271 | 350.8468 | 278.9692 | 205.9308 | 236.4753 | 188.3442 | 126.9411 | 413.8592 |
| Saint Lucia | 306.6542 | 490.1806 | 646.3646 | 680.0731 | 625.4924 | 524.6964 | 460.9189 | 403.6612 | 400.3461 | 405.7136 | 386.424 | 350.1362 | 283.9399 | 201.8404 | 236.1135 | 181.9937 | 117.6756 | 415.8385 |
| Saint Vincent and the Grenadines | 305.5105 | 486.082 | 645.4307 | 679.5443 | 625.5946 | 523.9636 | 453.3085 | 397.7636 | 392.7054 | 396.9944 | 376.6599 | 342.8584 | 274.6377 | 198.195 | 226.3065 | 172.1853 | 114.4354 | 396.3901 |
| Samoa | 355.2209 | 567.4776 | 752.5845 | 789.4185 | 717.7148 | 604.6926 | 525.5101 | 462.1013 | 458.8028 | 464.4504 | 446.2227 | 408.6222 | 328.8572 | 230.5676 | 268.5459 | 202.7785 | 128.6473 | 416.4375 |
| San Marino | 380.7537 | 619.4413 | 820.4423 | 844.6937 | 778.3493 | 671.2147 | 581.106 | 517.5312 | 514.0512 | 507.0543 | 464.0279 | 416.263 | 340.7562 | 224.0782 | 277.212 | 211.6907 | 131.7345 | 499.871 |
| Sao Tome and Principe | 294.1378 | 463.8053 | 615.3766 | 639.9783 | 587.6217 | 492.8014 | 430.5396 | 382.2978 | 379.6877 | 383.2855 | 362.1832 | 334.8942 | 265.5711 | 188.9651 | 217.0153 | 163.0628 | 102.4111 | 345.7885 |
| Saudi Arabia | 265.7891 | 421.7596 | 556.4288 | 568.042 | 514.4253 | 431.9026 | 380.8764 | 341.0844 | 335.4055 | 339.4744 | 319.2527 | 285.7948 | 226.2541 | 164.2559 | 192.7027 | 151.4024 | 96.3912 | 381.7234 |
| Senegal | 290.2369 | 462.5766 | 622.8422 | 650.42 | 597.0784 | 502.3568 | 440.8412 | 388.2651 | 380.965 | 383.1176 | 361.8118 | 330.5149 | 262.1511 | 187.0368 | 215.4609 | 162.564 | 103.7363 | 330.2688 |
| Serbia | 304.6447 | 486.612 | 643.0059 | 676.8381 | 620.092 | 523.5123 | 457.2339 | 404.7165 | 403.2473 | 409.092 | 390.2743 | 359.5786 | 287.0644 | 217.7282 | 238.8713 | 184.2542 | 109.3437 | 405.4462 |
| Seychelles | 382.0012 | 604.3417 | 789.0201 | 820.6367 | 748.6118 | 639.3036 | 562.6622 | 499.7112 | 494.4899 | 498.7908 | 472.9772 | 430.7192 | 351.9343 | 248.5807 | 292.6192 | 223.5018 | 142.3516 | 503.0967 |
| Sierra Leone | 295.6265 | 469.4973 | 623.5129 | 648.7595 | 595.1874 | 496.0251 | 429.2524 | 377.5716 | 372.7009 | 376.3054 | 356.9275 | 329.5527 | 261.7797 | 189.2547 | 215.7137 | 161.9618 | 102.7856 | 333.3722 |
| Singapore | 391.7626 | 636.918 | 845.3923 | 874.7404 | 789.2863 | 703.3245 | 562.0767 | 544.4831 | 527.7393 | 552.9923 | 514.9198 | 469.987 | 394.012 | 260.6894 | 314.2409 | 240.182 | 153.0815 | 572.7418 |
| Slovakia | 305.9077 | 487.4602 | 644.7884 | 674.2687 | 617.6447 | 520.3179 | 456.6601 | 405.2459 | 402.3858 | 410.4685 | 394.5136 | 367.8818 | 298.2447 | 210.6869 | 249.0229 | 189.7722 | 122.1903 | 418.5431 |
| Slovenia | 305.3296 | 486.6858 | 642.3643 | 669.4992 | 613.4103 | 516.6896 | 453.0928 | 402.8896 | 398.5749 | 403.7072 | 385.21 | 358.469 | 288.8236 | 201.9532 | 241.8408 | 189.2844 | 125.0012 | 401.8803 |
| Solomon Islands | 355.5181 | 571.7811 | 759.8968 | 798.5841 | 724.8366 | 610.6731 | 532.721 | 468.0722 | 461.7044 | 463.3489 | 440.0724 | 401.8751 | 318.9392 | 233.8642 | 255.2335 | 189.7595 | 118.4556 | 410.4731 |
| Somalia | 291.6678 | 456.0655 | 608.3267 | 634.4879 | 581.0693 | 492.471 | 433.9544 | 387.6901 | 393.3867 | 393.5975 | 374.8111 | 341.3038 | 273.7432 | 206.1631 | 228.253 | 173.0923 | 109.2494 | 305.4246 |
| South Africa | 294.281 | 464.8994 | 617.7884 | 641.4883 | 591.0915 | 499.8574 | 440.9075 | 394.9898 | 391.4037 | 398.2588 | 376.8452 | 346.5415 | 277.5923 | 202.4495 | 233.9158 | 177.2668 | 111.7637 | 384.5854 |
| South Sudan | 291.4709 | 467.6585 | 629.6697 | 667.381 | 605.697 | 507.6899 | 434.02 | 378.6295 | 369.6081 | 368.5507 | 347.9844 | 316.0835 | 246.3216 | 172.9967 | 201.1763 | 151.8688 | 98.8108 | 309.2272 |
| Spain | 380.4817 | 621.258 | 822.3045 | 841.5578 | 767.2212 | 653.6276 | 564.7931 | 508.1476 | 513.2368 | 523.1194 | 485.8068 | 436.5439 | 351.236 | 231.4148 | 285.3216 | 217.9433 | 135.4552 | 504.009 |
| Sri Lanka | 383.5814 | 614.8459 | 814.1183 | 853.8915 | 777.1858 | 657.5211 | 574.5048 | 508.0914 | 502.9473 | 508.1571 | 482.5007 | 439.7615 | 352.1055 | 249.8203 | 287.8529 | 219.364 | 139.8073 | 497.4584 |
| Sudan | 272.7184 | 436.7757 | 587.333 | 616.6704 | 571.2956 | 476.4988 | 409.8785 | 357.7158 | 346.8918 | 349.4469 | 329.92 | 298.7088 | 235.5756 | 167.6681 | 195.5406 | 148.1728 | 95.46657 | 321.2947 |
| Suriname | 305.64 | 491.0381 | 654.6637 | 685.6701 | 626.094 | 526.2422 | 460.5994 | 406.3092 | 401.2722 | 407.7006 | 391.6846 | 362.0589 | 287.2498 | 199.2138 | 236.5974 | 180.7337 | 116.5036 | 393.3579 |
| Sweden | 383.2451 | 626.9071 | 825.2764 | 842.9987 | 767.2351 | 654.152 | 567.0662 | 507.1793 | 507.1475 | 516.1678 | 477.7835 | 424.3979 | 337.2131 | 223.098 | 274.9012 | 211.3724 | 131.1964 | 493.8039 |
| Switzerland | 380.3517 | 621.8786 | 822.8728 | 840.4538 | 765.37 | 653.8526 | 564.1246 | 504.8147 | 505.8573 | 513.5162 | 480.8267 | 429.7934 | 344.9768 | 225.9015 | 279.6157 | 215.4367 | 134.5888 | 508.3465 |
| Syrian Arab Republic | 275.3523 | 453.4748 | 616.0017 | 641.8143 | 577.1149 | 475.1634 | 412.6223 | 362.0004 | 353.7999 | 356.8964 | 340.592 | 310.794 | 244.2944 | 174.4529 | 197.3254 | 141.5376 | 76.94736 | 340.1584 |
| Taiwan (Province of China) | 336.1469 | 538.8052 | 712.9996 | 747.7214 | 692.1854 | 583.7447 | 511.6146 | 450.676 | 443.2484 | 448.0487 | 420.9258 | 382.7322 | 305.2632 | 204.3967 | 252.5063 | 188.2645 | 115.5475 | 467.9825 |
| Tajikistan | 305.8539 | 488.5522 | 646.7621 | 678.3295 | 620.9587 | 524.5556 | 460.7639 | 407.6382 | 402.0064 | 408.0381 | 388.3538 | 352.238 | 277.2571 | 210.9322 | 235.5377 | 184.2702 | 118.679 | 376.8167 |
| Thailand | 382.5604 | 612.5476 | 810.9244 | 848.6398 | 772.5369 | 655.0971 | 574.3087 | 507.2576 | 501.7051 | 506.3867 | 479.7168 | 436.2266 | 348.867 | 237.3222 | 285.2288 | 217.0451 | 137.5591 | 523.6159 |
| Timor-Leste | 382.0607 | 614.1933 | 814.2306 | 851.9129 | 774.4845 | 651.8443 | 564.7751 | 495.9838 | 490.6938 | 501.2257 | 478.8657 | 433.6851 | 339.8833 | 241.2363 | 272.9405 | 209.4091 | 131.2541 | 433.5574 |
| Togo | 290.7006 | 463.7045 | 627.7194 | 665.7917 | 608.785 | 503.6162 | 438.457 | 386.1395 | 384.5372 | 392.929 | 372.7816 | 356.6699 | 280.1601 | 203.4578 | 229.9341 | 176.2078 | 110.9613 | 339.7202 |
| Tokelau | 350.2095 | 577.437 | 723.9216 | 833.1094 | 673.421 | 642.1322 | 515.8325 | 473.1616 | 473.3002 | 447.812 | 448.1798 | 383.6233 | 332.1117 | 221.2909 | 257.3949 | 200.2725 | 125.0227 | 417.7688 |
| Tonga | 356.3232 | 572.5691 | 764.7676 | 808.7889 | 736.934 | 620.323 | 538.0338 | 470.197 | 465.1559 | 470.9783 | 449.4062 | 410.23 | 327.7497 | 230.4149 | 271.5037 | 209.8605 | 137.189 | 417.7404 |
| Trinidad and Tobago | 306.7961 | 488.9688 | 647.3668 | 676.9679 | 620.5223 | 520.7562 | 455.7287 | 403.5891 | 399.1617 | 404.4111 | 384.6443 | 352.3074 | 283.8793 | 195.1096 | 235.0133 | 179.3814 | 115.7792 | 411.088 |
| Tunisia | 273.1533 | 440.742 | 589.7898 | 610.4533 | 560.6972 | 472.5534 | 414.015 | 365.5675 | 357.2449 | 360.127 | 343.1661 | 314.0378 | 248.5007 | 177.1781 | 206.2448 | 156.2836 | 100.9209 | 366.6937 |
| Turkey | 264.9859 | 422.5016 | 560.0711 | 580.0002 | 537.2673 | 453.9682 | 399.3024 | 352.503 | 348.4888 | 354.7942 | 342.9124 | 307.5362 | 252.9294 | 171.8575 | 205.5337 | 154.0067 | 96.92288 | 364.4195 |
| Turkmenistan | 303.5446 | 481.3507 | 633.6042 | 661.6328 | 610.1799 | 518.5709 | 456.6796 | 409.9466 | 408.6976 | 416.2153 | 398.9362 | 366.4047 | 290.8995 | 206.8208 | 241.181 | 187.5521 | 124.1231 | 384.8187 |
| Tuvalu | 352.6058 | 563.2835 | 741.249 | 773.2692 | 704.253 | 599.8144 | 528.5693 | 469.579 | 468.0419 | 475.6563 | 454.6904 | 417.2299 | 331.2892 | 232.9741 | 268.4499 | 200.5442 | 125.4067 | 439.7224 |
| Uganda | 294.983 | 466.7597 | 624.8676 | 652.6308 | 597.1538 | 505.9835 | 437.7867 | 387.493 | 386.3221 | 388.0485 | 369.1972 | 337.8967 | 266.3953 | 194.9937 | 221.2615 | 170.9398 | 110.3407 | 310.4431 |
| Ukraine | 307.5124 | 491.0191 | 648.2156 | 680.2094 | 630.1375 | 535.4854 | 468.9892 | 418.4253 | 416.159 | 426.4009 | 411.827 | 381.8907 | 309.6058 | 224.0603 | 255.8102 | 192.0724 | 124.0085 | 427.0233 |
| United Arab Emirates | 272.3823 | 432.0298 | 521.9305 | 481.4995 | 481.1856 | 381.5487 | 333.7414 | 296.9336 | 298.7831 | 306.4312 | 292.504 | 280.6813 | 229.1637 | 163.2918 | 182.8109 | 128.32 | 77.13705 | 375.1823 |
| United Kingdom | 382.9667 | 624.9192 | 824.1021 | 843.6765 | 769.6833 | 656.7612 | 570.2763 | 512.3208 | 512.5904 | 520.7474 | 481.9541 | 428.511 | 343.1519 | 228.5303 | 276.7767 | 211.441 | 132.0541 | 502.5931 |
| United Republic of Tanzania | 299.8249 | 471.3574 | 628.2209 | 651.4819 | 594.9337 | 505.6061 | 437.834 | 384.4652 | 379.4824 | 378.0463 | 355.9878 | 325.9932 | 260.3779 | 188.4295 | 215.0847 | 164.1155 | 105.5822 | 322.734 |
| United States of America | 472.0656 | 749.3678 | 1013.786 | 1067.719 | 1013.518 | 882.1282 | 779.4955 | 724.1293 | 748.9071 | 779.8976 | 730.4604 | 648.7032 | 518.6554 | 328.6748 | 412.4183 | 315.0965 | 194.7054 | 671.8918 |
| United States Virgin Islands | 306.5993 | 493.3905 | 660.6461 | 697.2002 | 636.8261 | 533.9834 | 466.76 | 410.8165 | 407.1595 | 414.1124 | 393.1427 | 359.6121 | 288.1819 | 211.1151 | 240.0405 | 184.9142 | 119.9653 | 397.8468 |
| Uruguay | 397.4502 | 638.2097 | 850.3016 | 883.4944 | 821.1644 | 705.5025 | 616.7304 | 561.4103 | 573.0944 | 592.5359 | 554.9638 | 500.5281 | 403.9996 | 268.9581 | 329.4711 | 250.2821 | 152.6655 | 531.2737 |
| Uzbekistan | 306.2754 | 488.9858 | 647.7411 | 678.4362 | 624.8702 | 527.492 | 462.6644 | 409.3687 | 407.0062 | 414.0772 | 396.9714 | 368.0383 | 296.0681 | 234.9468 | 250.6576 | 179.6598 | 109.9591 | 392.5409 |
| Vanuatu | 358.4043 | 577.5864 | 766.4848 | 800.1311 | 723.1097 | 608.9484 | 529.9341 | 465.7451 | 459.1661 | 459.5629 | 435.933 | 395.0191 | 312.4865 | 231.2891 | 253.4773 | 191.9851 | 122.4077 | 417.9424 |
| Venezuela (Bolivarian Republic of) | 307.6199 | 495.6824 | 660.946 | 689.4768 | 627.4391 | 526.0847 | 459.331 | 406.3438 | 403.81 | 408.9211 | 389.6399 | 356.4259 | 283.4656 | 191.0521 | 234.7053 | 178.9104 | 114.7038 | 398.9585 |
| Viet Nam | 381.706 | 611.2545 | 806.071 | 844.6643 | 771.6726 | 653.4852 | 570.5369 | 505.585 | 502.4571 | 509.4916 | 485.7873 | 444.1497 | 358.7717 | 252.538 | 294.9863 | 224.4445 | 144.4498 | 518.2194 |
| Yemen | 273.5643 | 438.5628 | 585.5019 | 607.7858 | 556.9479 | 467.6986 | 410.5379 | 364.6198 | 359.2394 | 362.5582 | 343.1472 | 311.6864 | 246.1005 | 178.2681 | 202.8745 | 154.4674 | 99.44969 | 315.3711 |
| Zambia | 297.0064 | 467.416 | 621.2554 | 645.5685 | 587.6773 | 493.1325 | 424.373 | 376.9918 | 378.1908 | 382.8125 | 363.8191 | 331.5192 | 260.6563 | 191.1228 | 214.5915 | 162.0562 | 102.7803 | 326.5884 |
| Zimbabwe | 295.1827 | 470.5163 | 630.5419 | 657.1322 | 602.4871 | 501.2202 | 435.8763 | 391.2127 | 397.0244 | 403.5971 | 379.6451 | 348.9442 | 274.7867 | 200.3377 | 227.0824 | 172.5221 | 111.0279 | 339.4583 |

**Supplementary table 7. Age distribution of DALYs rate for alopecia areata in different countries in 2019.**

| **2019 DALY rate** | **15 to 19** | **20 to 24** | **25 to 29** | **30 to 34** | **35 to 39** | **40 to 44** | **45 to 49** | **50 to 54** | **55 to 59** | **60 to 64** | **65 to 69** | **70 to 74** | **75 to 79** | **80 plus** | **80-84** | **85-89** | **90-94** | **all ages** |
| --- | --- | --- | --- | --- | --- | --- | --- | --- | --- | --- | --- | --- | --- | --- | --- | --- | --- | --- |
| Afghanistan | 4.93529 | 7.670216 | 10.65485 | 11.12099 | 10.29458 | 8.684008 | 7.449435 | 6.679671 | 6.372473 | 6.689931 | 6.176762 | 5.652132 | 4.344078 | 3.031759 | 3.415393 | 2.558908 | 1.574245 | 5.377352 |
| Albania | 5.433521 | 8.648874 | 11.88313 | 12.61771 | 11.97948 | 10.29246 | 8.811658 | 7.80618 | 7.276324 | 7.403546 | 6.881746 | 6.445363 | 5.175587 | 3.62408 | 4.115049 | 3.255974 | 2.178749 | 7.511142 |
| Algeria | 5.011049 | 7.775872 | 10.77086 | 11.42363 | 10.64225 | 9.022971 | 7.709175 | 6.831116 | 6.421493 | 6.576531 | 6.060734 | 5.631944 | 4.352799 | 3.154297 | 3.513765 | 2.584561 | 1.450915 | 6.641897 |
| American Samoa | 6.397044 | 10.22189 | 14.02349 | 15.04474 | 13.82877 | 11.66845 | 9.990393 | 8.723656 | 8.327848 | 8.478133 | 7.804368 | 7.188974 | 5.653845 | 3.971162 | 4.55027 | 3.582013 | 2.301305 | 7.94461 |
| Andorra | 6.873751 | 11.03143 | 15.30772 | 15.78868 | 14.64496 | 12.4966 | 10.56996 | 9.377763 | 8.969565 | 9.054348 | 8.247155 | 7.300674 | 6.070445 | 3.744866 | 4.765695 | 3.683788 | 2.287 | 9.644081 |
| Angola | 5.307225 | 8.239222 | 11.59837 | 12.15965 | 11.44383 | 9.662031 | 8.279742 | 7.339048 | 6.953497 | 7.187889 | 6.612153 | 6.204548 | 4.856108 | 3.490975 | 3.901736 | 3.034571 | 1.938511 | 5.795464 |
| Antigua and Barbuda | 5.571199 | 8.671459 | 12.04671 | 12.99497 | 12.11688 | 10.13796 | 8.794554 | 7.728537 | 7.241678 | 7.484671 | 6.892058 | 6.443963 | 5.085332 | 3.53539 | 4.085319 | 3.288562 | 2.084019 | 7.774267 |
| Argentina | 7.173588 | 11.33075 | 15.73572 | 16.65969 | 15.60377 | 13.43989 | 11.5012 | 10.43168 | 10.25303 | 10.70828 | 10.02694 | 8.993558 | 7.266351 | 4.82041 | 5.753202 | 4.484245 | 2.724177 | 9.823942 |
| Armenia | 5.484628 | 8.723569 | 12.16918 | 12.91019 | 12.10871 | 10.21268 | 8.863253 | 7.87339 | 7.416274 | 7.645904 | 7.130167 | 6.717096 | 5.399223 | 3.780712 | 4.316287 | 3.281027 | 2.078817 | 7.778557 |
| Australia | 7.16002 | 11.26476 | 15.67088 | 16.58607 | 15.47353 | 13.36532 | 11.51403 | 10.41108 | 10.15019 | 10.55829 | 9.552869 | 8.414329 | 6.670234 | 4.249472 | 5.177688 | 3.997536 | 2.543604 | 9.87469 |
| Austria | 6.854713 | 11.0155 | 15.32401 | 15.887 | 14.63187 | 12.56844 | 10.66996 | 9.535126 | 9.222941 | 9.594828 | 8.824673 | 7.920141 | 6.413874 | 4.127476 | 4.987574 | 3.978245 | 2.441614 | 9.470417 |
| Azerbaijan | 5.52365 | 8.682419 | 12.0483 | 12.72609 | 11.89399 | 10.12272 | 8.774245 | 7.739948 | 7.335273 | 7.587186 | 7.067754 | 6.681665 | 5.317899 | 4.022826 | 4.306386 | 3.294014 | 2.019554 | 7.802452 |
| Bahamas | 5.532382 | 8.716446 | 12.10602 | 12.94264 | 12.03746 | 10.19872 | 8.668214 | 7.746058 | 7.34632 | 7.587508 | 7.050668 | 6.642656 | 5.273028 | 3.575191 | 4.206951 | 3.239624 | 2.14943 | 7.718746 |
| Bahrain | 4.979264 | 7.597964 | 10.43265 | 10.64309 | 9.297403 | 7.829038 | 6.891423 | 6.242082 | 5.773579 | 5.946699 | 5.454082 | 5.382141 | 4.331599 | 3.26302 | 3.564933 | 2.67082 | 1.677105 | 6.948816 |
| Bangladesh | 4.988901 | 7.817491 | 10.83111 | 11.40414 | 10.62225 | 9.026214 | 7.654093 | 6.735901 | 6.310028 | 6.414542 | 5.861646 | 5.427547 | 4.284943 | 3.002983 | 3.412219 | 2.598547 | 1.594667 | 6.485894 |
| Barbados | 5.564876 | 8.781709 | 12.11807 | 12.95609 | 12.06955 | 10.15837 | 8.804188 | 7.874264 | 7.30336 | 7.555287 | 6.993118 | 6.545574 | 5.166791 | 3.537178 | 4.186834 | 3.214375 | 2.048726 | 7.637826 |
| Belarus | 5.526667 | 8.716188 | 11.98169 | 12.77896 | 11.99068 | 10.12321 | 8.761142 | 7.829965 | 7.362231 | 7.743897 | 7.324685 | 6.846695 | 5.625608 | 3.886387 | 4.533153 | 3.5354 | 2.256184 | 7.759079 |
| Belgium | 6.807726 | 10.91486 | 15.1985 | 15.85213 | 14.6545 | 12.48591 | 10.6763 | 9.480309 | 9.178321 | 9.397778 | 8.689568 | 7.790444 | 6.352121 | 4.068645 | 4.954533 | 3.849078 | 2.397984 | 9.169899 |
| Belize | 5.598171 | 8.726033 | 12.0495 | 12.83292 | 12.04329 | 10.12424 | 8.647983 | 7.611642 | 7.158947 | 7.352115 | 6.739434 | 6.234319 | 4.914336 | 3.338105 | 3.958188 | 3.129356 | 2.018288 | 7.158735 |
| Benin | 5.344672 | 8.307792 | 11.54098 | 12.2608 | 11.45635 | 9.605082 | 8.127956 | 7.284755 | 6.827434 | 7.132454 | 6.51599 | 6.113364 | 4.822934 | 3.424328 | 3.849945 | 3.082272 | 1.94039 | 5.821251 |
| Bermuda | 5.512107 | 8.795778 | 12.10081 | 12.92407 | 11.97375 | 10.11898 | 8.591765 | 7.660849 | 7.311079 | 7.572038 | 7.040881 | 6.575977 | 5.232126 | 3.549478 | 4.228162 | 3.255673 | 2.193305 | 7.628595 |
| Bhutan | 4.906924 | 7.572332 | 10.49118 | 11.07086 | 10.3061 | 8.707501 | 7.476367 | 6.613093 | 6.2763 | 6.452883 | 5.970418 | 5.528005 | 4.353974 | 2.971036 | 3.409941 | 2.599021 | 1.66673 | 6.572971 |
| Bolivia (Plurinational State of) | 5.498755 | 8.760986 | 12.0528 | 12.78183 | 11.95928 | 10.1098 | 8.618624 | 7.648339 | 7.226736 | 7.420091 | 6.96842 | 6.473435 | 5.03422 | 3.555982 | 4.065708 | 3.129429 | 1.996913 | 6.956982 |
| Bosnia and Herzegovina | 5.553942 | 8.596498 | 11.96871 | 12.83701 | 11.94348 | 10.06416 | 8.626242 | 7.623592 | 7.207624 | 7.525333 | 6.946933 | 6.558243 | 5.243062 | 3.745605 | 4.209588 | 3.22282 | 2.096341 | 7.652025 |
| Botswana | 5.275106 | 8.204012 | 11.37501 | 12.00993 | 11.1526 | 9.274696 | 8.017039 | 7.212834 | 6.870425 | 7.102641 | 6.549704 | 6.226899 | 4.998114 | 3.628299 | 4.054789 | 3.16842 | 2.03002 | 6.938557 |
| Brazil | 5.512219 | 8.777173 | 12.05996 | 12.86284 | 12.0001 | 10.22399 | 8.698645 | 7.743242 | 7.301721 | 7.57639 | 7.032599 | 6.538272 | 5.202858 | 3.543194 | 4.234827 | 3.258436 | 2.045047 | 7.646868 |
| Brunei Darussalam | 7.1041 | 11.32077 | 15.70725 | 16.45883 | 15.19396 | 13.03068 | 11.29127 | 10.20422 | 9.840166 | 10.28267 | 9.496566 | 8.653614 | 7.058265 | 5.64644 | 5.968747 | 5.284784 | 3.184362 | 10.35539 |
| Bulgaria | 5.550208 | 8.672935 | 11.88406 | 12.71051 | 11.81672 | 9.944506 | 8.58129 | 7.638105 | 7.156498 | 7.517141 | 7.102494 | 6.686592 | 5.364294 | 3.790273 | 4.31964 | 3.366057 | 2.154274 | 7.582629 |
| Burkina Faso | 5.333652 | 8.34668 | 11.69552 | 12.45531 | 11.46945 | 9.726097 | 8.308466 | 7.420925 | 6.961727 | 7.22794 | 6.564238 | 6.157704 | 4.827061 | 3.423651 | 3.896093 | 3.006672 | 1.882407 | 5.821611 |
| Burundi | 5.314636 | 8.250764 | 11.37821 | 11.98217 | 10.99345 | 9.230193 | 7.948912 | 6.958135 | 6.609267 | 6.758767 | 6.270582 | 5.738642 | 4.5664 | 3.309397 | 3.71981 | 2.888123 | 1.86073 | 5.762331 |
| Cabo Verde | 5.321424 | 8.185862 | 11.2977 | 11.9718 | 11.14665 | 9.356992 | 8.056848 | 7.236164 | 6.953037 | 7.301299 | 6.759864 | 6.320058 | 4.98745 | 3.38817 | 4.03264 | 3.069083 | 1.918221 | 6.950077 |
| Cambodia | 6.884659 | 10.87035 | 14.99366 | 15.90889 | 14.85132 | 12.70747 | 10.88453 | 9.592949 | 9.181608 | 9.46432 | 8.772706 | 7.985603 | 6.318314 | 4.406329 | 4.992558 | 3.861607 | 2.445609 | 8.986708 |
| Cameroon | 5.31388 | 8.248333 | 11.54132 | 12.13081 | 11.34749 | 9.42045 | 8.087083 | 7.222681 | 6.833825 | 6.992512 | 6.433535 | 6.048107 | 4.764686 | 3.469969 | 3.909604 | 3.017344 | 1.901331 | 6.11342 |
| Canada | 8.118152 | 12.46346 | 17.58201 | 18.795 | 18.34774 | 15.9257 | 13.79832 | 12.80885 | 12.68238 | 13.4788 | 12.31502 | 10.87766 | 8.669311 | 5.431911 | 6.657188 | 5.186424 | 3.235517 | 11.79783 |
| Central African Republic | 5.278267 | 8.301964 | 11.59649 | 12.22212 | 11.32875 | 9.451962 | 7.983599 | 7.149395 | 6.703395 | 7.107623 | 6.599367 | 6.260793 | 5.000633 | 3.700943 | 4.072357 | 3.192781 | 1.959193 | 6.002028 |
| Chad | 5.318546 | 8.242147 | 11.60227 | 12.37455 | 11.45816 | 9.602029 | 8.159232 | 7.092723 | 6.618893 | 6.693285 | 6.155107 | 5.702557 | 4.442333 | 3.235701 | 3.654678 | 2.833129 | 1.843196 | 5.383046 |
| Chile | 7.134468 | 11.35027 | 15.71735 | 16.60937 | 15.51633 | 13.32351 | 11.58641 | 10.41721 | 10.29792 | 10.81554 | 9.840064 | 8.798094 | 7.063458 | 4.596118 | 5.513005 | 4.22716 | 2.534109 | 10.08992 |
| China | 6.054947 | 9.661286 | 13.42309 | 14.37338 | 13.2979 | 11.25353 | 9.644685 | 8.567525 | 8.01347 | 8.265164 | 7.576299 | 6.939289 | 5.520168 | 3.991975 | 4.430232 | 3.554908 | 2.425629 | 8.775755 |
| Colombia | 5.572846 | 8.786847 | 12.06215 | 12.82663 | 12.09674 | 10.19335 | 8.755077 | 7.823142 | 7.389763 | 7.589734 | 6.996435 | 6.557382 | 5.156007 | 3.397183 | 4.173153 | 3.176439 | 2.037517 | 7.494064 |
| Comoros | 5.32405 | 8.212678 | 11.35799 | 11.92856 | 11.10711 | 9.458348 | 8.118199 | 7.242148 | 6.89566 | 7.093661 | 6.603413 | 6.108798 | 4.729508 | 3.290522 | 3.757174 | 2.891495 | 1.844103 | 6.55922 |
| Congo | 5.327431 | 8.260253 | 11.49387 | 12.1679 | 11.29536 | 9.456729 | 7.923958 | 7.04673 | 6.776888 | 7.057182 | 6.559852 | 6.125133 | 4.763869 | 3.46479 | 3.869979 | 2.95711 | 1.872031 | 6.363156 |
| Cook Islands | 6.590459 | 10.37789 | 14.31553 | 15.16818 | 14.05788 | 12.0241 | 10.24608 | 8.891923 | 8.204796 | 8.563887 | 7.751527 | 7.283514 | 5.590032 | 3.906049 | 4.596366 | 3.460892 | 2.216264 | 8.350407 |
| Costa Rica | 5.582824 | 8.742791 | 12.15235 | 12.92786 | 12.10947 | 10.21443 | 8.738323 | 7.756823 | 7.327586 | 7.579884 | 7.036844 | 6.486013 | 5.043443 | 3.332584 | 4.078116 | 3.192756 | 1.980318 | 7.644511 |
| Côte d'Ivoire | 5.235032 | 8.213136 | 11.47319 | 12.05267 | 11.10774 | 9.161786 | 7.857232 | 7.014 | 6.604347 | 6.871439 | 6.281232 | 5.921292 | 4.645044 | 3.328023 | 3.742246 | 2.965743 | 1.89875 | 6.110645 |
| Croatia | 5.513921 | 8.690517 | 11.98701 | 12.75336 | 11.89148 | 10.0404 | 8.590299 | 7.63078 | 7.161023 | 7.448709 | 6.921206 | 6.557687 | 5.202007 | 3.707293 | 4.243558 | 3.33337 | 2.087586 | 7.538843 |
| Cuba | 5.51027 | 8.684798 | 11.87629 | 12.59616 | 11.80553 | 10.05658 | 8.6473 | 7.683032 | 7.269728 | 7.454476 | 6.969039 | 6.476444 | 5.090404 | 3.335356 | 4.077102 | 3.10685 | 1.989707 | 7.535996 |
| Cyprus | 6.85948 | 11.00677 | 15.42383 | 15.90963 | 14.77963 | 12.73403 | 10.95763 | 9.79535 | 9.364802 | 9.374788 | 8.563375 | 7.638794 | 6.12655 | 4.382283 | 4.880516 | 3.811077 | 2.353839 | 9.993536 |
| Czechia | 5.4662 | 8.663991 | 11.90284 | 12.6518 | 11.79655 | 9.995158 | 8.571867 | 7.540065 | 7.140486 | 7.442071 | 6.948832 | 6.492784 | 5.163175 | 3.546287 | 4.209552 | 3.292925 | 2.151436 | 7.503581 |
| Democratic People's Republic of Korea | 6.156331 | 9.568282 | 13.25765 | 13.93604 | 12.95423 | 10.95498 | 9.549036 | 8.521564 | 8.022247 | 8.402581 | 7.844567 | 7.356233 | 6.061156 | 4.498094 | 4.982996 | 4.008869 | 2.513781 | 8.535913 |
| Democratic Republic of the Congo | 5.264165 | 8.104084 | 11.27532 | 11.97575 | 11.14762 | 9.442316 | 8.000142 | 7.206004 | 6.76178 | 7.098101 | 6.547262 | 6.290949 | 4.974015 | 3.573778 | 4.034197 | 3.153686 | 1.965113 | 5.901762 |
| Denmark | 6.837728 | 11.08483 | 15.27342 | 15.77039 | 14.60933 | 12.49924 | 10.58111 | 9.432096 | 9.174165 | 9.352929 | 8.645208 | 7.696694 | 6.25555 | 4.070213 | 4.892917 | 3.854502 | 2.405408 | 9.15361 |
| Djibouti | 5.163133 | 7.957045 | 11.20101 | 12.15619 | 11.12889 | 9.462167 | 7.980877 | 6.914241 | 6.506376 | 6.674494 | 6.197804 | 5.705924 | 4.618965 | 3.341873 | 3.716805 | 2.901944 | 1.88946 | 6.538076 |
| Dominica | 5.489173 | 8.650586 | 11.96811 | 12.7752 | 11.84285 | 9.967458 | 8.571533 | 7.522459 | 6.965854 | 7.134421 | 6.801146 | 6.358919 | 5.037154 | 3.566073 | 4.098097 | 3.280178 | 2.106355 | 7.324957 |
| Dominican Republic | 5.562389 | 8.637525 | 12.00378 | 12.71808 | 11.83478 | 9.940671 | 8.60371 | 7.648551 | 7.259177 | 7.49892 | 6.963247 | 6.441711 | 5.159823 | 3.514546 | 4.141489 | 3.145219 | 2.016029 | 7.218413 |
| Ecuador | 5.528884 | 8.723537 | 12.03511 | 12.85875 | 11.98323 | 10.11243 | 8.745257 | 7.769738 | 7.243695 | 7.479259 | 6.925684 | 6.406511 | 5.07131 | 3.509026 | 4.034842 | 3.140921 | 1.983037 | 7.17528 |
| Egypt | 5.000671 | 7.710275 | 10.8522 | 11.27576 | 10.5252 | 8.959847 | 7.676309 | 6.720077 | 6.408038 | 6.481356 | 5.997206 | 5.415234 | 4.243532 | 2.860341 | 3.284451 | 2.367315 | 1.368294 | 6.267732 |
| El Salvador | 5.506746 | 8.865014 | 12.27492 | 13.09899 | 12.3582 | 10.45955 | 9.01622 | 7.981958 | 7.521499 | 7.735074 | 7.078016 | 6.58203 | 5.215907 | 3.367535 | 4.169401 | 3.198654 | 2.007913 | 7.33092 |
| Equatorial Guinea | 5.105538 | 7.781234 | 10.93156 | 11.7317 | 11.13982 | 9.614784 | 8.304316 | 7.472044 | 7.093292 | 7.391669 | 6.724872 | 6.339635 | 4.910441 | 3.46465 | 3.907808 | 3.020278 | 1.878611 | 6.051251 |
| Eritrea | 5.329643 | 8.118992 | 11.25805 | 11.92922 | 11.09094 | 9.348828 | 8.030602 | 7.222948 | 6.929974 | 7.28024 | 6.701897 | 6.311456 | 4.989495 | 3.69284 | 4.054937 | 3.148603 | 1.970533 | 6.196603 |
| Estonia | 5.522054 | 8.718971 | 11.91252 | 12.73502 | 11.86097 | 9.995662 | 8.595197 | 7.696716 | 7.319911 | 7.588273 | 7.17791 | 6.835264 | 5.575105 | 3.833739 | 4.490773 | 3.523597 | 2.226808 | 7.563364 |
| Eswatini | 5.287429 | 8.19046 | 11.60446 | 12.13178 | 11.16266 | 9.337795 | 8.040966 | 7.284356 | 6.950245 | 7.252214 | 6.702022 | 6.439527 | 5.134498 | 3.81404 | 4.183578 | 3.266032 | 2.024048 | 6.509066 |
| Ethiopia | 5.325629 | 8.309234 | 11.40799 | 12.23539 | 11.34566 | 9.613287 | 8.108574 | 7.126358 | 6.8607 | 6.984585 | 6.434607 | 5.773509 | 4.511092 | 3.171879 | 3.591325 | 2.765066 | 1.728705 | 5.968517 |
| Fiji | 6.417637 | 10.12829 | 13.898 | 14.80587 | 13.74351 | 11.53369 | 9.964776 | 8.727819 | 8.262565 | 8.506001 | 7.852877 | 7.257153 | 5.81611 | 4.259026 | 4.674171 | 3.710294 | 2.466408 | 8.290902 |
| Finland | 6.838286 | 10.86858 | 15.24825 | 15.84194 | 14.55636 | 12.43042 | 10.58283 | 9.443415 | 9.175032 | 9.469546 | 8.728371 | 7.819468 | 6.408358 | 4.135191 | 5.020032 | 3.971134 | 2.420784 | 9.160349 |
| France | 6.797115 | 10.94298 | 15.21741 | 15.89757 | 14.67606 | 12.61921 | 10.71856 | 9.548625 | 9.319843 | 9.687533 | 8.895245 | 7.920211 | 6.405093 | 4.093362 | 5.070106 | 4.007083 | 2.425224 | 9.051464 |
| Gabon | 5.363462 | 8.249618 | 11.52142 | 12.11708 | 11.33024 | 9.620901 | 8.180526 | 7.238208 | 6.735475 | 6.938529 | 6.393157 | 6.129197 | 4.953195 | 3.601152 | 4.107351 | 3.147613 | 1.994184 | 6.627507 |
| Gambia | 5.351053 | 8.300116 | 11.5911 | 12.31141 | 11.35276 | 9.536034 | 8.090037 | 7.174361 | 6.714282 | 6.948547 | 6.426454 | 5.972045 | 4.758259 | 3.481308 | 3.857762 | 3.044361 | 1.919172 | 6.098664 |
| Georgia | 5.444333 | 8.661316 | 11.9572 | 12.85005 | 12.01988 | 10.11612 | 8.662981 | 7.71975 | 7.343236 | 7.674929 | 7.140938 | 6.707078 | 5.434629 | 3.635513 | 4.351331 | 3.406452 | 2.208925 | 7.469551 |
| Germany | 6.822704 | 10.99802 | 15.4217 | 15.86784 | 14.54295 | 12.45695 | 10.61805 | 9.506906 | 9.135278 | 9.357753 | 8.709243 | 7.755793 | 6.311066 | 4.225246 | 4.932121 | 3.834878 | 2.404512 | 9.294905 |
| Ghana | 5.307457 | 8.330473 | 11.63274 | 12.35939 | 11.46396 | 9.674632 | 8.30014 | 7.45488 | 6.967685 | 7.221373 | 6.593369 | 6.272633 | 4.925973 | 3.526431 | 3.967158 | 3.077384 | 2.0029 | 6.567762 |
| Greece | 6.794679 | 10.94352 | 15.21584 | 15.95487 | 14.64921 | 12.59089 | 10.66278 | 9.622102 | 9.372422 | 9.583684 | 8.825268 | 7.925544 | 6.400725 | 4.136728 | 4.958702 | 3.852741 | 2.253524 | 9.26865 |
| Greenland | 8.179026 | 12.35792 | 17.42706 | 18.42627 | 17.907 | 14.98271 | 13.12008 | 12.02847 | 11.62981 | 11.72908 | 10.71758 | 8.983719 | 8.148086 | 5.61607 | 6.434548 | 5.155898 | 2.967281 | 11.26375 |
| Grenada | 5.471624 | 8.613599 | 11.90183 | 12.69572 | 11.83658 | 9.901155 | 8.530922 | 7.504028 | 7.006046 | 7.326806 | 6.814396 | 6.38333 | 5.164055 | 3.807902 | 4.365627 | 3.584815 | 2.299439 | 7.413016 |
| Guam | 6.393459 | 9.983198 | 13.74469 | 14.69928 | 13.78745 | 11.69482 | 10.05592 | 8.729026 | 8.375636 | 8.487249 | 8.122424 | 7.268142 | 5.863667 | 3.908053 | 4.791013 | 3.709556 | 2.337054 | 8.158956 |
| Guatemala | 5.510075 | 8.716014 | 12.0639 | 13.03283 | 12.24245 | 10.37409 | 8.857972 | 7.871192 | 7.342825 | 7.521436 | 6.973364 | 6.447086 | 5.02776 | 3.665722 | 4.013564 | 3.166098 | 2.073692 | 7.058657 |
| Guinea | 5.344936 | 8.445768 | 11.7268 | 12.44873 | 11.69661 | 9.744691 | 8.282639 | 7.271045 | 6.75757 | 6.905903 | 6.285564 | 5.858274 | 4.61173 | 3.212026 | 3.703327 | 2.960636 | 1.856608 | 5.872991 |
| Guinea-Bissau | 5.334641 | 8.218783 | 11.70584 | 12.35431 | 11.54017 | 9.610264 | 8.274913 | 7.389297 | 6.857215 | 7.126997 | 6.540918 | 6.163878 | 4.92978 | 3.573716 | 3.995515 | 3.11587 | 1.944941 | 6.190089 |
| Guyana | 5.493439 | 8.66507 | 12.04122 | 12.878 | 11.93877 | 10.05934 | 8.551347 | 7.590109 | 7.128463 | 7.392623 | 6.867583 | 6.391625 | 5.115079 | 3.610128 | 4.153588 | 3.279145 | 2.071312 | 7.233746 |
| Haiti | 5.501379 | 8.787542 | 12.13387 | 12.99675 | 12.10584 | 10.17637 | 8.690042 | 7.727702 | 7.247348 | 7.490653 | 6.874552 | 6.454899 | 5.015999 | 3.556818 | 4.029693 | 3.069206 | 1.945362 | 7.003811 |
| Honduras | 5.574698 | 8.844237 | 12.22079 | 13.09059 | 12.25379 | 10.24168 | 8.673158 | 7.713058 | 7.224566 | 7.479154 | 6.968917 | 6.468501 | 5.071775 | 3.548723 | 4.03617 | 3.055197 | 1.922753 | 6.980496 |
| Hungary | 5.520543 | 8.693256 | 11.98213 | 12.78599 | 11.95452 | 10.10683 | 8.663087 | 7.654234 | 7.223109 | 7.559431 | 7.090735 | 6.660021 | 5.327789 | 3.666948 | 4.320013 | 3.408426 | 2.126794 | 7.646281 |
| Iceland | 6.840503 | 10.98056 | 15.35871 | 15.91243 | 14.5637 | 12.52257 | 10.6934 | 9.531617 | 9.188849 | 9.416017 | 8.499695 | 7.588433 | 6.203752 | 3.847738 | 4.800048 | 3.743082 | 2.326104 | 9.259347 |
| India | 4.87237 | 7.702385 | 10.60093 | 11.24395 | 10.48029 | 8.801128 | 7.543985 | 6.65798 | 6.283869 | 6.457675 | 5.940696 | 5.474203 | 4.32263 | 3.095084 | 3.496397 | 2.680909 | 1.64925 | 6.433515 |
| Indonesia | 6.88192 | 11.03003 | 15.0213 | 16.04001 | 14.85157 | 12.55031 | 10.83821 | 9.519425 | 9.0337 | 9.194363 | 8.497726 | 7.775565 | 6.241499 | 4.388755 | 4.940395 | 3.779935 | 2.382814 | 9.344191 |
| Iran (Islamic Republic of) | 4.966344 | 7.828539 | 10.76683 | 11.432 | 10.59256 | 8.970466 | 7.680825 | 6.887177 | 6.403819 | 6.646273 | 6.196359 | 5.700551 | 4.418176 | 2.964651 | 3.483852 | 2.631759 | 1.614405 | 6.959605 |
| Iraq | 5.151504 | 8.029464 | 11.13072 | 11.77544 | 10.88348 | 9.307423 | 7.95027 | 7.037753 | 6.665537 | 6.908849 | 6.418535 | 5.842398 | 4.614253 | 3.255533 | 3.758832 | 2.908531 | 1.815008 | 6.53228 |
| Ireland | 6.841328 | 10.95831 | 15.2642 | 15.80561 | 14.66823 | 12.52256 | 10.71684 | 9.491997 | 9.281969 | 9.383464 | 8.653879 | 7.709573 | 6.116061 | 4.079715 | 4.82721 | 3.813018 | 2.380515 | 9.166952 |
| Israel | 6.797573 | 11.01664 | 15.33798 | 15.92434 | 14.61755 | 12.56523 | 10.72481 | 9.528405 | 9.37863 | 9.698525 | 8.819068 | 7.902888 | 6.333011 | 4.060205 | 4.960747 | 3.790814 | 2.328704 | 8.733467 |
| Italy | 6.869648 | 11.10994 | 15.32561 | 15.9508 | 14.67351 | 12.62199 | 10.76259 | 9.633454 | 9.326289 | 9.635334 | 8.862907 | 7.868724 | 6.376882 | 4.137889 | 4.999701 | 3.907627 | 2.395272 | 9.29183 |
| Jamaica | 5.547387 | 8.69058 | 12.0457 | 12.86824 | 12.09477 | 10.1959 | 8.7073 | 7.557155 | 7.125185 | 7.408022 | 6.863239 | 6.362186 | 5.105577 | 3.272188 | 4.138621 | 3.221621 | 2.06408 | 7.56392 |
| Japan | 7.162253 | 11.38343 | 15.71643 | 16.6417 | 15.52088 | 13.37349 | 11.44762 | 10.39428 | 10.03502 | 10.55609 | 9.991856 | 9.143164 | 7.306444 | 4.679945 | 5.803661 | 4.545631 | 2.878043 | 9.79325 |
| Jordan | 4.937482 | 7.585794 | 10.41146 | 11.01787 | 10.34255 | 8.720661 | 7.443282 | 6.675367 | 6.296468 | 6.560851 | 6.069444 | 5.672843 | 4.349839 | 3.124077 | 3.533807 | 2.685851 | 1.570639 | 6.211338 |
| Kazakhstan | 5.540578 | 8.688956 | 12.06035 | 12.84045 | 12.02167 | 10.10923 | 8.753067 | 7.713768 | 7.325941 | 7.585308 | 7.201651 | 6.808866 | 5.51114 | 4.042607 | 4.500695 | 3.498509 | 2.211309 | 7.418962 |
| Kenya | 5.339683 | 8.326525 | 11.4408 | 12.2067 | 11.28805 | 9.564749 | 8.152255 | 7.16613 | 6.836606 | 6.981798 | 6.470597 | 5.973239 | 4.762973 | 3.435536 | 3.881692 | 3.052233 | 1.910806 | 6.323498 |
| Kiribati | 6.515834 | 10.18408 | 14.08711 | 15.01244 | 13.97259 | 11.82194 | 10.19868 | 8.897006 | 8.353098 | 8.710327 | 8.3178 | 7.814618 | 6.092513 | 4.462995 | 4.876465 | 3.801002 | 2.418225 | 8.049304 |
| Kuwait | 4.936354 | 7.754684 | 10.83573 | 11.34946 | 10.34656 | 8.677789 | 7.434644 | 6.51353 | 6.151157 | 6.196977 | 5.846124 | 5.13904 | 4.124206 | 2.654147 | 3.200495 | 2.567004 | 1.571463 | 7.446221 |
| Kyrgyzstan | 5.557273 | 8.687996 | 12.03047 | 12.77907 | 12.0365 | 10.22102 | 8.728377 | 7.671486 | 7.386737 | 7.638689 | 7.167206 | 6.735701 | 5.376233 | 3.759263 | 4.348764 | 3.392018 | 2.099151 | 7.146716 |
| Lao People's Democratic Republic | 6.912343 | 10.94503 | 14.93394 | 15.93853 | 14.8044 | 12.46485 | 10.85965 | 9.560957 | 9.028538 | 9.211912 | 8.542737 | 7.787282 | 6.240363 | 4.3498 | 4.929609 | 3.839572 | 2.442305 | 8.893263 |
| Latvia | 5.527557 | 8.623816 | 12.00554 | 12.75394 | 11.90985 | 10.05251 | 8.7159 | 7.799752 | 7.367812 | 7.641871 | 7.187634 | 6.904536 | 5.484282 | 3.861028 | 4.508279 | 3.497889 | 2.270174 | 7.557907 |
| Lebanon | 4.95188 | 7.635783 | 10.7224 | 11.4631 | 10.76546 | 9.177107 | 7.941329 | 7.067576 | 6.599243 | 6.811159 | 6.27481 | 5.735393 | 4.492213 | 3.101675 | 3.575191 | 2.781221 | 1.73751 | 6.685976 |
| Lesotho | 5.29931 | 8.134438 | 11.37053 | 11.77663 | 10.8633 | 9.266772 | 8.008138 | 7.240743 | 6.956385 | 7.245291 | 6.654102 | 6.369111 | 5.08679 | 3.758474 | 4.186378 | 3.24397 | 2.021402 | 6.700499 |
| Liberia | 5.302769 | 8.299956 | 11.41982 | 12.18232 | 11.18358 | 9.377817 | 7.821252 | 6.965721 | 6.591599 | 6.757948 | 6.274231 | 5.8936 | 4.672658 | 3.175685 | 3.681064 | 2.818834 | 1.752375 | 6.206429 |
| Libya | 4.993639 | 7.674865 | 10.67653 | 11.13071 | 10.34539 | 8.799737 | 7.601486 | 6.776386 | 6.355499 | 6.567438 | 6.091254 | 5.609352 | 4.31014 | 2.915341 | 3.436205 | 2.705784 | 1.696673 | 6.950336 |
| Lithuania | 5.526441 | 8.700395 | 12.00458 | 12.76942 | 11.90875 | 10.10494 | 8.706635 | 7.764002 | 7.314779 | 7.616378 | 7.203261 | 6.83704 | 5.50881 | 3.846153 | 4.462453 | 3.509959 | 2.254447 | 7.561138 |
| Luxembourg | 6.843556 | 10.99117 | 15.21694 | 15.85097 | 14.63778 | 12.49547 | 10.59619 | 9.303242 | 9.012878 | 9.268672 | 8.542221 | 7.59307 | 6.370443 | 4.098829 | 4.977566 | 3.887051 | 2.437924 | 9.574829 |
| Madagascar | 5.363425 | 8.158304 | 11.37401 | 12.10842 | 11.22629 | 9.466557 | 8.159107 | 7.17141 | 6.831352 | 6.947664 | 6.469333 | 5.992633 | 4.689103 | 3.296698 | 3.735641 | 2.877545 | 1.852914 | 6.089118 |
| Malawi | 5.386549 | 8.332553 | 11.45663 | 12.27762 | 11.24748 | 9.43521 | 8.072895 | 7.157043 | 6.950319 | 7.113671 | 6.567403 | 6.160618 | 4.845931 | 3.472688 | 3.984064 | 3.108588 | 1.969639 | 5.974069 |
| Malaysia | 6.834105 | 10.83717 | 14.80761 | 15.73934 | 14.57739 | 12.47325 | 10.73732 | 9.46821 | 9.007237 | 9.202587 | 8.478436 | 7.708207 | 6.105353 | 4.231786 | 4.858645 | 3.753796 | 2.303001 | 9.338254 |
| Maldives | 6.846229 | 10.44572 | 14.09581 | 14.87907 | 13.83647 | 11.91953 | 10.30337 | 9.243782 | 8.862829 | 9.056016 | 8.436317 | 7.750672 | 6.122086 | 4.09026 | 4.740374 | 3.607014 | 2.299668 | 9.826462 |
| Mali | 5.278861 | 8.335909 | 11.62565 | 12.38037 | 11.4325 | 9.544076 | 8.063271 | 7.237579 | 6.666064 | 6.943756 | 6.332134 | 5.947777 | 4.579315 | 3.19034 | 3.671222 | 2.778142 | 1.747942 | 5.607325 |
| Malta | 6.883071 | 10.97053 | 15.23924 | 15.90698 | 14.53265 | 12.44532 | 10.68016 | 9.483275 | 9.151015 | 9.482571 | 8.684242 | 7.755198 | 6.294507 | 4.157799 | 4.932507 | 3.943416 | 2.428467 | 9.456249 |
| Marshall Islands | 6.402883 | 10.06166 | 13.83145 | 14.63628 | 13.65498 | 11.64049 | 9.996151 | 8.71516 | 8.339781 | 8.441415 | 7.669908 | 6.944523 | 5.524082 | 3.939579 | 4.389035 | 3.358093 | 2.105276 | 8.138231 |
| Mauritania | 5.35526 | 8.396848 | 11.64421 | 12.30702 | 11.47283 | 9.71199 | 8.262389 | 7.320042 | 6.830769 | 7.058912 | 6.415267 | 5.972013 | 4.673436 | 3.180266 | 3.670359 | 2.815666 | 1.751538 | 6.147787 |
| Mauritius | 6.85458 | 10.92914 | 14.9248 | 15.89388 | 14.72822 | 12.40572 | 10.72936 | 9.439999 | 9.024547 | 9.142016 | 8.431277 | 7.810645 | 6.254205 | 4.182928 | 5.023736 | 3.855072 | 2.437642 | 9.558788 |
| Mexico | 5.557114 | 8.850536 | 12.14248 | 12.97138 | 12.11851 | 10.27558 | 8.750493 | 7.778366 | 7.275692 | 7.506076 | 6.904916 | 6.425992 | 5.076652 | 3.444186 | 4.084428 | 3.10174 | 1.93402 | 7.424716 |
| Micronesia (Federated States of) | 6.394203 | 10.12782 | 13.8536 | 14.7821 | 13.67989 | 11.54797 | 10.02709 | 8.865162 | 8.29606 | 8.48239 | 7.882167 | 7.38548 | 6.020401 | 4.237463 | 4.783998 | 3.666525 | 2.335693 | 8.102504 |
| Monaco | 6.82589 | 10.99446 | 15.24192 | 15.86782 | 14.85857 | 12.67526 | 10.76279 | 9.54762 | 9.077702 | 9.320578 | 8.805931 | 7.822438 | 6.42316 | 3.892383 | 4.800171 | 3.821743 | 2.308983 | 9.020487 |
| Mongolia | 5.522874 | 8.649878 | 12.10875 | 12.87687 | 11.98629 | 10.11261 | 8.651789 | 7.732155 | 7.390381 | 7.675491 | 7.224943 | 6.786399 | 5.369421 | 4.00787 | 4.33314 | 3.450578 | 2.277957 | 7.435456 |
| Montenegro | 5.552315 | 8.627244 | 11.94659 | 12.77716 | 11.94616 | 10.10944 | 8.666949 | 7.602846 | 7.26176 | 7.370805 | 6.978591 | 6.626161 | 5.253575 | 3.769928 | 4.22431 | 3.261025 | 2.049044 | 7.564924 |
| Morocco | 4.961846 | 7.682124 | 10.87091 | 11.34192 | 10.61495 | 8.966986 | 7.784858 | 6.880963 | 6.409669 | 6.574373 | 6.028664 | 5.643011 | 4.512636 | 3.121182 | 3.613885 | 2.745093 | 1.65122 | 6.601285 |
| Mozambique | 5.382758 | 8.38606 | 11.5673 | 12.17753 | 11.27688 | 9.527151 | 8.192161 | 7.225981 | 6.822921 | 6.980681 | 6.459494 | 6.032221 | 4.795314 | 3.519385 | 3.987581 | 3.108975 | 1.948932 | 5.725141 |
| Myanmar | 6.917507 | 10.99111 | 15.06038 | 16.06942 | 14.98532 | 12.57922 | 10.97425 | 9.656621 | 9.138912 | 9.346366 | 8.670729 | 7.976516 | 6.323874 | 4.305575 | 4.990992 | 3.821971 | 2.460892 | 9.127063 |
| Namibia | 5.342108 | 8.25313 | 11.47987 | 11.95928 | 11.2502 | 9.439757 | 8.154556 | 7.289259 | 6.896788 | 7.250351 | 6.675756 | 6.251767 | 4.931565 | 3.529821 | 4.037304 | 3.143752 | 1.956126 | 6.531149 |
| Nauru | 6.413696 | 10.14267 | 13.89477 | 14.88214 | 13.84981 | 11.71817 | 10.20508 | 8.910563 | 8.449423 | 8.852166 | 8.423508 | 7.710264 | 6.020872 | 3.525852 | 3.924347 | 3.070398 | 2.22165 | 7.919214 |
| Nepal | 4.954981 | 7.862777 | 11.16433 | 11.89667 | 11.06169 | 9.219326 | 7.845316 | 6.722775 | 6.439358 | 6.54731 | 6.060105 | 5.579401 | 4.347968 | 3.108652 | 3.487338 | 2.732813 | 1.751841 | 6.402428 |
| Netherlands | 6.853654 | 10.97692 | 15.21598 | 15.88812 | 14.62747 | 12.63661 | 10.73744 | 9.499234 | 9.199553 | 9.466766 | 8.667195 | 7.738498 | 6.234402 | 4.222293 | 5.080519 | 3.933859 | 2.422659 | 9.220129 |
| New Zealand | 7.168225 | 11.29026 | 15.69246 | 16.75296 | 15.65142 | 13.56703 | 11.70583 | 10.54941 | 10.28462 | 10.62363 | 9.682993 | 8.467759 | 6.784263 | 4.256981 | 5.24891 | 3.963288 | 2.466937 | 9.672248 |
| Nicaragua | 5.541675 | 8.655566 | 12.06033 | 12.86373 | 11.99002 | 10.16979 | 8.706287 | 7.758652 | 7.302453 | 7.548291 | 7.011889 | 6.603785 | 5.192212 | 3.629098 | 4.133209 | 3.213542 | 2.057736 | 7.189047 |
| Niger | 5.384422 | 8.323654 | 11.48801 | 12.01833 | 11.36771 | 9.696438 | 8.339017 | 7.349716 | 6.754181 | 6.993368 | 6.524786 | 6.047658 | 4.735285 | 3.341193 | 3.770003 | 2.910055 | 1.828872 | 5.309061 |
| Nigeria | 5.382204 | 8.451528 | 11.67383 | 12.35183 | 11.45547 | 9.677507 | 8.245438 | 7.478267 | 7.046917 | 7.277824 | 6.594229 | 5.921395 | 4.682657 | 3.180213 | 3.670695 | 2.789885 | 1.785046 | 5.976738 |
| Niue | 6.28231 | 10.19243 | 14.09604 | 14.99569 | 13.78547 | 11.66844 | 10.08963 | 8.758548 | 8.128765 | 8.400503 | 8.060288 | 7.50633 | 5.976458 | 4.02822 | 4.686396 | 3.639258 | 2.318203 | 8.230315 |
| North Macedonia | 5.511705 | 8.589002 | 11.91438 | 12.69521 | 11.82025 | 10.01974 | 8.562758 | 7.535941 | 7.093841 | 7.358315 | 6.847353 | 6.461992 | 5.15333 | 3.792357 | 4.158102 | 3.191581 | 2.014604 | 7.773466 |
| Northern Mariana Islands | 6.417414 | 10.14724 | 14.08821 | 14.58211 | 13.14052 | 12.25647 | 9.205295 | 9.353963 | 7.905493 | 8.770672 | 7.677622 | 7.33203 | 5.560128 | 4.060578 | 4.724194 | 3.5971 | 2.23028 | 8.511406 |
| Norway | 6.851645 | 11.10706 | 15.26672 | 15.92218 | 14.65382 | 12.48273 | 10.60939 | 9.468268 | 9.078328 | 9.397363 | 8.582171 | 7.644286 | 6.166228 | 3.891993 | 4.856398 | 3.765668 | 2.300647 | 9.27958 |
| Oman | 4.969568 | 7.037869 | 9.373204 | 9.859392 | 9.261104 | 8.079432 | 6.877555 | 6.132933 | 5.864943 | 6.200015 | 6.11772 | 5.483184 | 4.261327 | 3.103416 | 3.449275 | 2.806538 | 1.752351 | 6.844269 |
| Pakistan | 4.920083 | 7.766177 | 10.68136 | 11.34167 | 10.5698 | 8.967888 | 7.642997 | 6.603843 | 6.167226 | 6.380637 | 5.921955 | 5.41196 | 4.312183 | 3.023221 | 3.409274 | 2.644346 | 1.643336 | 5.782468 |
| Palau | 6.363451 | 9.735146 | 13.05561 | 14.03401 | 13.0677 | 11.12366 | 9.768541 | 8.602091 | 8.293217 | 8.38336 | 7.827074 | 7.233399 | 5.78788 | 3.97794 | 4.613491 | 3.484407 | 2.219088 | 8.624999 |
| Palestine | 4.966282 | 7.695715 | 10.71378 | 11.31034 | 10.4996 | 8.881848 | 7.585114 | 6.73012 | 6.262519 | 6.596578 | 6.064528 | 5.698477 | 4.552647 | 3.260036 | 3.72602 | 2.90992 | 1.89455 | 5.970442 |
| Panama | 5.489536 | 8.672558 | 11.99567 | 12.75169 | 11.92305 | 10.04413 | 8.627873 | 7.656594 | 7.16616 | 7.378096 | 6.829894 | 6.4081 | 5.058287 | 3.304548 | 4.014057 | 3.162894 | 1.997069 | 7.12048 |
| Papua New Guinea | 6.348453 | 10.03288 | 13.96422 | 14.97409 | 13.75378 | 11.55615 | 10.021 | 8.627894 | 8.13917 | 8.234836 | 7.509043 | 6.976639 | 5.613487 | 3.920595 | 4.417855 | 3.503132 | 2.278254 | 7.762268 |
| Paraguay | 5.483111 | 8.666755 | 11.98943 | 12.65531 | 11.89502 | 9.955598 | 8.578642 | 7.622339 | 7.170919 | 7.37191 | 6.789902 | 6.496567 | 5.132959 | 3.404977 | 4.112336 | 3.225938 | 2.086599 | 7.214308 |
| Peru | 5.384635 | 8.621393 | 12.07507 | 12.88553 | 12.00927 | 10.15244 | 8.707069 | 7.669971 | 7.259636 | 7.48257 | 6.918699 | 6.431823 | 5.110441 | 3.290734 | 4.014518 | 3.090115 | 1.91747 | 7.281439 |
| Philippines | 6.884883 | 11.00206 | 14.98968 | 15.98794 | 14.7708 | 12.49025 | 10.81437 | 9.493265 | 9.019708 | 9.274687 | 8.616956 | 7.907796 | 6.372163 | 4.429597 | 5.104105 | 3.908089 | 2.362946 | 8.754217 |
| Poland | 5.542364 | 8.761342 | 12.00719 | 12.8052 | 11.94655 | 10.14281 | 8.662692 | 7.718057 | 7.265117 | 7.543136 | 7.074773 | 6.670793 | 5.360108 | 3.678298 | 4.36497 | 3.381091 | 2.153919 | 7.797122 |
| Portugal | 6.79874 | 11.02323 | 15.22091 | 15.94622 | 14.69419 | 12.60036 | 10.79286 | 9.717978 | 9.428427 | 9.673122 | 8.902516 | 7.98824 | 6.485779 | 4.20187 | 5.036752 | 3.885863 | 2.407183 | 9.407556 |
| Puerto Rico | 5.501228 | 8.74226 | 12.12018 | 12.95979 | 12.12397 | 10.16047 | 8.676529 | 7.780203 | 7.335878 | 7.562588 | 7.003125 | 6.515972 | 5.177567 | 3.370992 | 4.118596 | 3.264201 | 2.093569 | 7.521881 |
| Qatar | 4.668007 | 6.176431 | 8.726393 | 9.267147 | 8.986288 | 7.701742 | 6.475676 | 5.647453 | 5.152585 | 5.378513 | 5.073491 | 4.730462 | 3.872348 | 2.652824 | 2.86602 | 2.010078 | 1.295308 | 6.945452 |
| Republic of Korea | 7.103901 | 11.25541 | 15.64831 | 16.39403 | 15.37305 | 13.1946 | 11.3406 | 10.24456 | 9.933058 | 10.41733 | 9.716927 | 8.890773 | 7.33159 | 5.151393 | 5.912522 | 4.674763 | 2.919474 | 10.39685 |
| Republic of Moldova | 5.495591 | 8.726319 | 11.99177 | 12.76973 | 11.86287 | 10.0334 | 8.641422 | 7.813169 | 7.380314 | 7.681496 | 7.16021 | 6.802401 | 5.415855 | 3.698013 | 4.330716 | 3.380019 | 2.123175 | 7.927645 |
| Romania | 5.472778 | 8.677627 | 11.97138 | 12.6986 | 11.93771 | 10.03896 | 8.568203 | 7.632074 | 7.222629 | 7.591863 | 7.095623 | 6.68594 | 5.31494 | 3.761524 | 4.370003 | 3.359832 | 2.120244 | 7.527 |
| Russian Federation | 5.54454 | 8.776523 | 12.02424 | 12.83447 | 12.00317 | 10.25547 | 8.746729 | 7.810519 | 7.395933 | 7.732555 | 7.273206 | 6.862243 | 5.583252 | 3.916821 | 4.551976 | 3.477517 | 2.178113 | 7.755744 |
| Rwanda | 5.365956 | 8.255591 | 11.28624 | 11.98865 | 11.12577 | 9.609844 | 8.359862 | 7.348088 | 6.932802 | 7.180483 | 6.655318 | 6.240635 | 4.844861 | 3.488727 | 3.948182 | 3.025633 | 1.923103 | 6.314919 |
| Saint Kitts and Nevis | 5.552764 | 8.704765 | 12.06442 | 12.7919 | 11.90567 | 9.879535 | 8.548848 | 7.472127 | 7.107387 | 7.462131 | 6.830989 | 6.364775 | 5.057651 | 3.636513 | 4.137173 | 3.389968 | 2.280127 | 7.664179 |
| Saint Lucia | 5.512735 | 8.650483 | 11.93085 | 12.83283 | 11.96851 | 10.0389 | 8.619548 | 7.611449 | 7.097061 | 7.365159 | 6.881901 | 6.307871 | 5.101487 | 3.571033 | 4.145639 | 3.2697 | 2.103777 | 7.686492 |
| Saint Vincent and the Grenadines | 5.4689 | 8.625579 | 11.97739 | 12.85374 | 11.96368 | 9.930719 | 8.502209 | 7.457708 | 7.001436 | 7.306583 | 6.666692 | 6.213931 | 4.903589 | 3.543668 | 4.013834 | 3.129845 | 2.093017 | 7.32867 |
| Samoa | 6.344749 | 10.16128 | 13.96487 | 14.88876 | 13.73235 | 11.54743 | 9.933482 | 8.719659 | 8.231691 | 8.47432 | 7.900706 | 7.294821 | 5.855135 | 4.001668 | 4.645327 | 3.537735 | 2.263128 | 7.705671 |
| San Marino | 6.819649 | 10.95036 | 15.12061 | 15.94596 | 14.86689 | 12.78367 | 10.90862 | 9.741945 | 9.275336 | 9.288362 | 8.421446 | 7.504437 | 6.217973 | 3.999142 | 4.899908 | 3.848201 | 2.337384 | 9.247874 |
| Sao Tome and Principe | 5.357048 | 8.233883 | 11.37255 | 12.03899 | 11.17026 | 9.521673 | 8.049683 | 7.249422 | 6.847575 | 7.053291 | 6.482613 | 6.067915 | 4.796218 | 3.325333 | 3.812316 | 2.872671 | 1.82462 | 6.413493 |
| Saudi Arabia | 4.82162 | 7.481821 | 10.27157 | 10.71288 | 9.880365 | 8.314356 | 7.108213 | 6.467063 | 5.994614 | 6.185659 | 5.587651 | 5.086971 | 3.9997 | 2.815378 | 3.275077 | 2.628676 | 1.681769 | 7.119931 |
| Senegal | 5.196271 | 8.196048 | 11.52911 | 12.24801 | 11.33822 | 9.615707 | 8.242162 | 7.321841 | 6.842645 | 7.091935 | 6.484233 | 6.020897 | 4.717427 | 3.330534 | 3.811092 | 2.924973 | 1.915911 | 6.08997 |
| Serbia | 5.470769 | 8.676209 | 11.89961 | 12.7819 | 11.91487 | 10.03138 | 8.547544 | 7.640514 | 7.249238 | 7.476244 | 6.974324 | 6.521957 | 5.169279 | 3.812329 | 4.142791 | 3.326566 | 1.962652 | 7.504966 |
| Seychelles | 6.845503 | 10.75001 | 14.71693 | 15.60106 | 14.49853 | 12.21879 | 10.66625 | 9.416495 | 8.901427 | 9.146702 | 8.39364 | 7.680425 | 6.244554 | 4.304275 | 5.03318 | 3.917092 | 2.486624 | 9.379216 |
| Sierra Leone | 5.328311 | 8.264644 | 11.53664 | 12.24371 | 11.28777 | 9.444809 | 8.071978 | 7.174952 | 6.686728 | 6.932946 | 6.465298 | 5.994029 | 4.72616 | 3.347387 | 3.78231 | 2.931527 | 1.852022 | 6.144922 |
| Singapore | 7.125377 | 11.38647 | 15.80471 | 16.61635 | 15.2596 | 13.51792 | 10.6649 | 10.3205 | 9.547102 | 10.2387 | 9.442912 | 8.658452 | 7.306171 | 4.675347 | 5.590032 | 4.362122 | 2.774477 | 10.75969 |
| Slovakia | 5.547658 | 8.639707 | 11.93243 | 12.77092 | 11.80004 | 9.990228 | 8.611981 | 7.621333 | 7.230106 | 7.502946 | 6.992166 | 6.666978 | 5.357946 | 3.732196 | 4.368539 | 3.418279 | 2.212426 | 7.770209 |
| Slovenia | 5.522919 | 8.60048 | 11.9498 | 12.62604 | 11.68642 | 9.90818 | 8.536183 | 7.607243 | 7.165621 | 7.387724 | 6.87278 | 6.482984 | 5.20751 | 3.535809 | 4.200594 | 3.368985 | 2.186839 | 7.445189 |
| Solomon Islands | 6.415977 | 10.25311 | 14.13268 | 15.08878 | 13.81923 | 11.61765 | 9.991556 | 8.789814 | 8.278785 | 8.403216 | 7.821822 | 7.246567 | 5.639277 | 4.083912 | 4.447226 | 3.341081 | 2.085852 | 7.612282 |
| Somalia | 5.263057 | 8.030195 | 11.19365 | 11.90451 | 10.97963 | 9.302657 | 8.184849 | 7.227925 | 7.044371 | 7.124539 | 6.683926 | 6.203462 | 4.964806 | 3.625889 | 3.978933 | 3.139923 | 1.943407 | 5.622053 |
| South Africa | 5.300301 | 8.202533 | 11.34466 | 11.96892 | 11.09479 | 9.453251 | 8.127177 | 7.330729 | 6.927692 | 7.243835 | 6.670241 | 6.199672 | 4.956684 | 3.538678 | 4.061449 | 3.150815 | 1.950422 | 7.065389 |
| South Sudan | 5.221029 | 8.236821 | 11.62452 | 12.46536 | 11.43737 | 9.563694 | 8.081895 | 7.028527 | 6.550935 | 6.641418 | 6.077444 | 5.604577 | 4.296958 | 2.990585 | 3.447652 | 2.687474 | 1.71179 | 5.661098 |
| Spain | 6.840763 | 10.95386 | 15.2769 | 15.9725 | 14.68104 | 12.52792 | 10.8043 | 9.464518 | 9.30861 | 9.573789 | 8.8169 | 7.902365 | 6.416603 | 4.117668 | 5.027367 | 3.929952 | 2.440808 | 9.374507 |
| Sri Lanka | 6.886385 | 10.99769 | 15.09022 | 16.12196 | 14.98547 | 12.57083 | 10.91821 | 9.575487 | 9.110725 | 9.296759 | 8.555631 | 7.819967 | 6.179826 | 4.284152 | 4.916328 | 3.792763 | 2.427201 | 9.226951 |
| Sudan | 4.924526 | 7.726918 | 10.90497 | 11.56988 | 10.85833 | 9.081605 | 7.712432 | 6.715316 | 6.253799 | 6.428005 | 5.845636 | 5.353267 | 4.268099 | 2.959566 | 3.435672 | 2.652485 | 1.664922 | 5.940284 |
| Suriname | 5.501437 | 8.660302 | 12.07963 | 12.91627 | 11.87919 | 10.04005 | 8.582833 | 7.602634 | 7.156176 | 7.477024 | 6.936725 | 6.555519 | 5.168418 | 3.52508 | 4.152881 | 3.249812 | 2.089045 | 7.257396 |
| Sweden | 6.855429 | 11.17905 | 15.37705 | 15.87211 | 14.60391 | 12.55074 | 10.66239 | 9.516538 | 9.229109 | 9.472242 | 8.687142 | 7.754199 | 6.096813 | 3.966533 | 4.861416 | 3.812693 | 2.305708 | 9.15863 |
| Switzerland | 6.817388 | 11.00774 | 15.28712 | 15.98446 | 14.62303 | 12.49894 | 10.62836 | 9.481429 | 9.087742 | 9.42122 | 8.709776 | 7.827502 | 6.304957 | 4.013619 | 4.938519 | 3.866071 | 2.403116 | 9.43219 |
| Syrian Arab Republic | 4.988024 | 7.973602 | 11.32966 | 12.02451 | 10.94411 | 9.07734 | 7.732068 | 6.8302 | 6.381149 | 6.496663 | 6.113885 | 5.619982 | 4.331946 | 3.018065 | 3.404679 | 2.464828 | 1.340193 | 6.269256 |
| Taiwan (Province of China) | 6.065457 | 9.594654 | 13.38294 | 14.20224 | 13.36034 | 11.25752 | 9.648604 | 8.532781 | 8.083848 | 8.295904 | 7.640161 | 6.979323 | 5.569906 | 3.650805 | 4.475506 | 3.410732 | 2.071692 | 8.749096 |
| Tajikistan | 5.503301 | 8.679293 | 12.0348 | 12.78572 | 11.98276 | 10.06958 | 8.680666 | 7.690474 | 7.257191 | 7.522223 | 7.018245 | 6.426263 | 5.054207 | 3.783116 | 4.209719 | 3.346917 | 2.125774 | 7.007039 |
| Thailand | 6.928477 | 10.96974 | 15.01867 | 16.09725 | 14.91644 | 12.55748 | 10.91631 | 9.550145 | 9.164228 | 9.265642 | 8.633455 | 7.91242 | 6.23901 | 4.173371 | 4.986415 | 3.860565 | 2.430173 | 9.762542 |
| Timor-Leste | 6.874651 | 10.86626 | 15.11782 | 16.08165 | 14.78362 | 12.46688 | 10.62249 | 9.367689 | 8.893896 | 9.192294 | 8.505148 | 7.818593 | 6.019898 | 4.202928 | 4.740636 | 3.668144 | 2.318259 | 8.015327 |
| Togo | 5.264444 | 8.23171 | 11.57646 | 12.48569 | 11.61768 | 9.520251 | 8.183512 | 7.319896 | 6.867508 | 7.257914 | 6.681303 | 6.471842 | 5.0642 | 3.60345 | 4.032059 | 3.205922 | 2.000293 | 6.268336 |
| Tokelau | 6.340066 | 10.26084 | 13.50136 | 15.67872 | 12.92044 | 12.31344 | 9.754824 | 8.8507 | 8.487478 | 8.158636 | 7.868634 | 6.854071 | 5.874569 | 3.852409 | 4.460845 | 3.546771 | 2.178766 | 7.73506 |
| Tonga | 6.403269 | 10.19461 | 14.20884 | 15.27004 | 14.08677 | 11.85421 | 10.12774 | 8.854101 | 8.323819 | 8.56804 | 7.991135 | 7.374066 | 5.946535 | 4.036032 | 4.706443 | 3.740921 | 2.455222 | 7.738836 |
| Trinidad and Tobago | 5.544538 | 8.672307 | 11.94237 | 12.7264 | 11.83244 | 10.00941 | 8.532323 | 7.575278 | 7.070055 | 7.371733 | 6.855958 | 6.381639 | 5.096569 | 3.490746 | 4.165839 | 3.255883 | 2.112156 | 7.611203 |
| Tunisia | 4.959153 | 7.796057 | 10.90257 | 11.53078 | 10.76081 | 9.016765 | 7.778234 | 6.937001 | 6.404402 | 6.578779 | 6.134135 | 5.737504 | 4.435763 | 3.096421 | 3.570839 | 2.784918 | 1.784055 | 6.816255 |
| Turkey | 4.80437 | 7.464554 | 10.381 | 10.8874 | 10.25817 | 8.622796 | 7.484142 | 6.658863 | 6.264123 | 6.489469 | 6.132005 | 5.548674 | 4.514021 | 3.00372 | 3.5717 | 2.717383 | 1.709758 | 6.75083 |
| Turkmenistan | 5.499313 | 8.598209 | 11.79766 | 12.53468 | 11.68711 | 9.993913 | 8.639243 | 7.747379 | 7.363825 | 7.623625 | 7.11896 | 6.700237 | 5.275204 | 3.732907 | 4.331663 | 3.405646 | 2.286909 | 7.173439 |
| Tuvalu | 6.371302 | 10.04235 | 13.73515 | 14.61426 | 13.59966 | 11.44803 | 9.962084 | 8.781485 | 8.368421 | 8.640115 | 8.079981 | 7.423313 | 5.876283 | 4.01001 | 4.595049 | 3.49059 | 2.200642 | 8.142627 |
| Uganda | 5.356979 | 8.263075 | 11.53644 | 12.28418 | 11.36803 | 9.701581 | 8.161156 | 7.223559 | 6.940837 | 7.052773 | 6.563459 | 6.109485 | 4.844645 | 3.496647 | 3.953308 | 3.101388 | 1.967194 | 5.721079 |
| Ukraine | 5.557912 | 8.78144 | 11.97977 | 12.84285 | 12.12966 | 10.24145 | 8.854151 | 7.855139 | 7.444587 | 7.787043 | 7.287787 | 6.893358 | 5.626003 | 3.980468 | 4.526462 | 3.457504 | 2.206794 | 7.925124 |
| United Arab Emirates | 4.918916 | 7.652188 | 9.728151 | 9.102641 | 9.195259 | 7.376312 | 6.26271 | 5.659384 | 5.402772 | 5.552391 | 5.145311 | 5.040708 | 4.094035 | 2.80442 | 3.130479 | 2.215574 | 1.377175 | 7.085882 |
| United Kingdom | 6.839434 | 11.04336 | 15.21958 | 15.88001 | 14.59477 | 12.49366 | 10.6864 | 9.545536 | 9.189904 | 9.469422 | 8.675799 | 7.711782 | 6.19594 | 4.029074 | 4.844363 | 3.778804 | 2.332849 | 9.264531 |
| United Republic of Tanzania | 5.461588 | 8.347397 | 11.57518 | 12.27273 | 11.35482 | 9.624152 | 8.202358 | 7.155373 | 6.819082 | 6.967124 | 6.378828 | 5.9217 | 4.723274 | 3.327728 | 3.766115 | 2.958714 | 1.899353 | 5.95898 |
| United States of America | 8.548831 | 13.12698 | 18.62342 | 19.93442 | 19.05999 | 16.67546 | 14.4431 | 13.34519 | 13.24693 | 13.93243 | 12.90692 | 11.39233 | 9.092141 | 5.612552 | 6.991578 | 5.432727 | 3.346331 | 12.2428 |
| United States Virgin Islands | 5.519456 | 8.762606 | 12.2789 | 13.16105 | 12.19601 | 10.19773 | 8.740883 | 7.70992 | 7.328968 | 7.567867 | 7.110437 | 6.529647 | 5.263637 | 3.766033 | 4.25754 | 3.343363 | 2.175543 | 7.36384 |
| Uruguay | 7.14364 | 11.30872 | 15.8136 | 16.67213 | 15.63161 | 13.47616 | 11.60161 | 10.51079 | 10.29162 | 10.90046 | 10.01175 | 9.120203 | 7.335174 | 4.803898 | 5.820862 | 4.553497 | 2.752738 | 9.831663 |
| Uzbekistan | 5.52051 | 8.715652 | 12.00446 | 12.82373 | 11.93939 | 10.13614 | 8.671387 | 7.732912 | 7.320073 | 7.60219 | 7.109457 | 6.689853 | 5.399227 | 4.171864 | 4.43108 | 3.278065 | 2.002624 | 7.289738 |
| Vanuatu | 6.466301 | 10.21403 | 14.11862 | 15.10178 | 13.79965 | 11.54959 | 9.891172 | 8.726666 | 8.230622 | 8.351484 | 7.716167 | 7.121263 | 5.544472 | 4.057022 | 4.434705 | 3.398665 | 2.156515 | 7.719279 |
| Venezuela (Bolivarian Republic of) | 5.569725 | 8.862857 | 12.24236 | 12.98081 | 12.07715 | 10.04443 | 8.668472 | 7.643542 | 7.241459 | 7.452834 | 6.995612 | 6.509032 | 5.096476 | 3.382647 | 4.110619 | 3.221865 | 2.063667 | 7.415612 |
| Viet Nam | 6.914381 | 10.89663 | 15.0164 | 16.06781 | 14.95664 | 12.60371 | 10.91328 | 9.470955 | 9.152084 | 9.454305 | 8.768872 | 8.044811 | 6.412216 | 4.423743 | 5.137473 | 3.978593 | 2.538522 | 9.705717 |
| Yemen | 4.907765 | 7.711428 | 10.70608 | 11.30549 | 10.57506 | 8.885421 | 7.673521 | 6.825725 | 6.459327 | 6.622088 | 6.101615 | 5.585043 | 4.402789 | 3.118085 | 3.536235 | 2.718033 | 1.76972 | 5.798093 |
| Zambia | 5.34087 | 8.22375 | 11.42966 | 12.05969 | 11.16127 | 9.416896 | 7.889208 | 6.940114 | 6.790839 | 7.009395 | 6.502337 | 5.998481 | 4.737256 | 3.362073 | 3.752209 | 2.895806 | 1.844284 | 6.001926 |
| Zimbabwe | 5.391519 | 8.32483 | 11.61075 | 12.26788 | 11.4176 | 9.481694 | 8.057555 | 7.263067 | 7.078841 | 7.384429 | 6.740883 | 6.318504 | 4.92606 | 3.492914 | 3.925408 | 3.089277 | 1.93111 | 6.248761 |
